# The Form of the HOMA Geometric Aromaticity Index Is Universal and Allows the Design of Electronic and Magnetic Descriptors

**DOI:** 10.1021/acs.joc.5c00740

**Published:** 2025-06-17

**Authors:** Jan Cz. Dobrowolski, Sławomir Ostrowski

**Affiliations:** 86904 Institute of Nuclear Chemistry and Technology, 16 Dorodna Street, 03-195 Warsaw, Poland

## Abstract

The HOMA geometrical aromaticity index is unique. It is simple, based on observable bond distances, and can be defined using experimental or computational bond values. Moreover, its mathematical form expresses geometric similarity to the archetypal aromatic benzene. Here, we show that HOMA is simply a kind of mean of the errors squared (MSE). This is why the index is such a good measure of aromaticity. Thus, only a slight modification grounds the HOMA index on electronic or magnetic properties, producing an electronic or magnetic molecular measure expressing electronic or magnetic similarity/dissimilarity to benzene. Based on an analysis of over 70 neutral or charged carbocyclic rings, we compare the HOMA indices based on bond distances, selected electron density properties in bond or ring critical points, chemical shifts, and spin–spin coupling constants. We conclude that using electronic and magnetic variables discloses separate trends that are invisible if CC bond lengths are used. Such new HOMA indices can be used to study different facets of aromaticity and as more general molecular structure descriptors.

## Graphical abstract

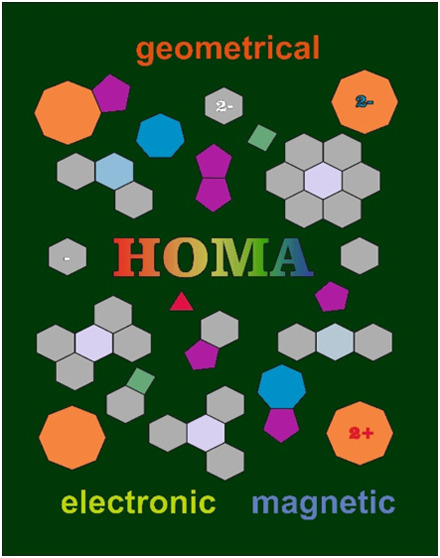

## 1 Introduction

Aromaticity is nonobservable, but it is recognizable in myriad physicochemical properties of molecules and must be evaluated.^1^ Therefore, it is described by a bunch of authoritarian indices expressing its different aspects: geometrical, magnetic, energetic, electronic, topological, etc.^1^ Nevertheless, from the philosophical empiricism point of view,^2^ aromaticity does not exist and, as such, is nothing but what is provided by those indices. On the other hand, from the point of view of philosophical realism, using the aromaticity indices, we are approaching the essence of aromaticity. Regardless of which philosophical position you are closer to, aromaticity indices must be understood in depth.

The indices are various:^1^ some have, and others have no upper and/or lower limits. Some are based on measurable properties, and others are grounded on again nonobservable quantities. Some are based on properties of the part of the rings, while the others express properties of the whole rings or even whole molecules.

The HOMA geometrical aromaticity index^3–6^ is unique. Its formula is simple. It is based on observable bond distances, and its primary version has been defined using experimental values plus a condition referring to the nonexisting cyclohexatriene molecule. Still, an existing molecule can replace the reference cyclohexatriene with only a cosmetic change of the HOMA values obtained previously.^7^ Thus, although HOMA is as arbitrary as any other aromaticity index, it can be robustly defined based only on observable distances. Even more, the HOMA mathematical formula satisfies axioms of a similarity function between the examined and benzene ring.^8^ Hence, the index has a simple and precise meaning: it expresses a geometric similarity to the archetypal aromatic benzene. A molecule geometrically similar to benzene is also chemically similar to benzene and thus is aromatic.

On the other hand, the nucleus-independent chemical shift (NICS) group of aromaticity indices^9–12^ are probably the most popular. There are, however, serious reasons to have reservations about such aromaticity measures. First, NICS indices are determined at points without atoms using the property adequate for atoms.^9^ As a result, the nonobservable aromaticity is measured with a nonobservable quantity. Second, since the early days of the NICS index, successive modifications have required new, gradually more complicated assumptions.^12–15^ Only the part of the shielding tensor is now considered. The shielding tensor axes should be explicitly oriented, and it is hoped that the ring’s σ and π electron structures are always perfectly separated despite the ring deformations. Even if there is a procedure for tying the integral NICS (INICS) index^15–17^ to Ampère-Maxwell’s law,^18,19^ the above drawbacks dictate that one should keep a reserve against such a defined measure.

Still, the interpretation of standard ^1^H and ^13^C NMR spectra starts from identifying “aromatic” and “aliphatic” NMR signals. In ^13^C NMR, the aromatic carbon atoms absorb in the range of 120–150 ppm. Thus, why not define the aromaticity index based on this easily observable and well-computable quantity? Why not insert the ring’s ^13^C NMR chemical shifts or isotropic parts of shielding tensors into the HOMA index expression?

Indeed, the HOMA mathematical formula permits us to replace distances with another quantity depicting bonds in a ring. Using HOMA­(Rho) for various carbocyclic structures, where Rho is the electron density in a bond critical point, we demonstrated an excellent quadratic correlation with commonly defined HOMA­(*R*), where *R* denotes bond distance.^20,21^ Still, the HOMA­(Rho) indices of benzynes, cyclopropanes, and some other compounds deviated from the perfect correlation. The irregularities occur because bond distances *R* and the electron density in bond critical point Rho express different properties of bonds: the former geometric and the latter electronic.

Other examples are the *I*_5_ and *I*_6_ indices of Bird,^22,23^ and the Bond Order Index of Aromaticity (BOIA) of Bultinck et al.,^24,25^ which are kind of the HOMA­(BO) indices, where BO stands for bond order. The bond order was introduced as the difference in the electron population of bonding and antibonding molecular orbitals.^26^ Now, IUPAC describes it as the “*degree of bonding between two atoms relative to that of a single bond*”.^27^ Although the bond order and bond length correlate exponentially,^28^ the former property is electronic while the latter is geometric. Hence, they describe diverse facets of molecular structure, and there are deviations from the correlations between HOMA­(*R*) and HOMA­(BO) (*I*_5_, *I*_6_, BOIA).^24,25,29,30^

If so, one can simultaneously define HOMA on ring parameters corresponding to the ring’s geometric, electronic, and magnetic properties to express various aspects of aromaticity with indices designed precisely in the same way. Such an approach would release the aromaticity analysis from the bias originating from comparing indices differing not only by a physical basis but also by mathematical properties of the index, like limitlessness and possessing a one-sided limit. All HOMA indices would show similarity to benzene in terms of the ring’s geometric, electronic, and magnetic properties. We already know that according to electron density at the bond critical point, bond order, and bond distance some rings are dissimilar. However, whether the magnetic parameters convoluted with the HOMA equation will provide an analogous or complementary picture of aromaticity remains unknown. Additionally, we consider HOMA-similarity indices constructed based on the atom, bond, and ring parameters and discuss the consequences of such different definitions.

## 2 Results and Discussion

### 2.1 Definition of the Universal HOMA Index

For hydrocarbon rings, the universal HOMA equation looks like the standard one, but it is defined using arbitrary variables *X* instead of the bond distances *R*:

1 $$HOMA\left(X\right)=1-\frac{\alpha_{X}}{n}\sum_{i=1}^{n}{\left(X_{i}-X_{B}\right)}^{2}$$

where *X_i_* and *X*_B_ stand for the *i*th variable related to the analyzed *n*-membered hydrocarbon ring and the reference variable in the benzene ring (B), and HOMA­(*X*_B_) ≡ 1 for benzene. α*_X_* normalizes the index to be unitless. The second assumption requires HOMA­(*X*_C_) ≡ β*_X_* < 1 for declared ring C ≠ B. In the standard approach, C = CHT, i.e., nonexisting cyclohexatriene ring, and HOMA­(*X*_CHT_) ≡ β_CHT_ ≡ 0. Hereafter, C is assumed to be the chair cyclohexane ring for which HOMA­(*X*_C_) ≡ β_C_ ≡ −4. Note that α*_X_* and β*_X_* constants are method-dependent and must be reestablished to satisfy HOMA­(*X*_B_) ≡ 1 and HOMA­(*X*_C_) ≡ β*_X_* conditions whenever the *X* variable estimation method is modified.

We remark that for HOMA­(*R*) with β_C_ ≡ −4, where C denotes chair cyclohexane, HOMA­(*R*_CHT_) ≈ 0 and such HOMA­(*R*) produces figures very similar to those of the standard HOMA index. Still, using the existing chair cyclohexane instead of hypothetical cyclohexatriene helps avoid referring to hypothetical cyclohexatriene and solidly grounds the index on measurable value foundations.

The equivalent definition of the universal HOMA is as follows:^8^

2 $$HOMA\left(X\right)=1-EN\left(X\right)-GEO\left(X\right)=1-\alpha_{X}\cdot \left[{\left(\mathrm{X̅}-\mathrm{Y̅}\right)}^{2}+\mathrm{Var}\left(X_{i}\right)\right]$$

where

$${EN\left(X\right)=\alpha }_{X}\cdot {\left(\mathrm{X̅}-\mathrm{Y̅}\right)}^{2},{GEO\left(X\right)=\alpha }_{X}\cdot \mathrm{Var}\left(X_{i}\right)$$

in which $\mathrm{X̅}=\frac{1}{n}\cdot \sum_{i=1}^{n}X_{i}$ , $\mathrm{Y̅}\equiv X_{B}$ , and $Var\left(X_{i}\right)=\frac{1}{n}\cdot \sum_{i=1}^{n}{\left(X_{i}-\mathrm{X̅}\right)}^{2}$ .

So, the appropriate arithmetical means of the variables in the examined and reference rings and the variation of *X* in the single examined ring are needed to calculate the universal HOMA­(*X*) index. The summation operations contain information on the ring size.

However, eq 2 means that HOMA is a linear function of a scaled mean squared error (MSE):^31^

3 $$MSE\left(\mathrm{\chi ̂}\right)=E{\left(\chi -\mathrm{\chi ̂}\right)}^{2}=E{\left(\left[\mathrm{\chi ̂}\right]-\chi \right)}^{2}+Var\left(\mathrm{\chi ̂}\right)=bias+variance$$

where χ̂ is an estimator of the parameter χ and MSE is the mathematical expectation *E*(·) of this value. As a result:

4 $$HOMA\left(X\right)=1-\alpha_{X}\cdot MSE\left(X\right)$$

Thus, HOMA is a linear function of the MSE, where the slope α_*X*_ is the MSE scaling coefficient, EN­(*X*) is the *bias*, and GEO­(*X*) is the *variance*. This is why the index is such a good measure of aromaticity despite being derived from early quantum chemical theory.

### 2.2 Types of the Universal HOMA Index

The index can differ by (i) the variable *X*’s physical origin and (ii) the part of the ring it comes from.

(i) **The Physical Origin of X.** The bond distances in the standard HOMA index, in a generalized equation, can be replaced by a variety of parameters expressing electronic or magnetic properties besides geometrical properties such as bond orders, electron density parameters in the bond critical points (BCP), nucleus-independent chemical shifts in the middle of bonds, or the spin–spin coupling constants through one bond. In that way, the universal HOMA index can express various physical properties of rings.
(ii) **The Part of the Ring the Universal HOMA Index Comes From.**
  - *Atoms.* Equation 1 allows us to ground the index on the atom properties: a six-membered ring has six bonds between six atoms. Thus, the HOMA indices using the ring atoms’ partial charges, isotropic part of the shielding tensors, or atom electron contribution to the ring π orbitals can be determined like standard HOMA with modified α*_X_* and β*_X_* constants.
  - *Bonds.* As pointed out above, diverse bond properties can be used to define various kinds of the universal HOMA index and can express diverse physical aspects of the ring’s aromaticity.
  - *Angles.* Similarly, the universal HOMA index can also be constructed based on angles and dihedral angles, spin–spin coupling constants through two or more bonds, etc.
  - *Whole Rings.* The universal HOMA index can be based on a single property of the entire ring. In such a case, *Var*(*X*_*i*_) ≡ 0, but the remaining term (*X̅* – *Y̅*)^2^ can sufficiently describe HOMA­(*X*). The properties of the whole ring can be geometrical (*e.g*., ring surface area), electronic (*e.g*., electron density parameter in the ring critical point RCP), or magnetic, like NICS..

Unified mathematical expressions for aromaticity indices defined on distinct physical quantities, in terms of linear functions of MSE, filter out errors by comparing heterogeneous index types with and without upper and/or lower limits. Yet, the utility of a universal HOMA index based on a given variable *X* matters. Forecasting the index’s usefulness needs the analysis below.

### 2.3 The Electronic HOMA Indices

The electron density, which depicts the probability of an electron’s presence near a given point, is observable. It can be measured, for example, with X-ray diffraction scanning, transmission electron microscopy, scanning tunneling microscopy, and atomic force microscopy. The electron density allows for the direct calculation of any electronic molecular property. It can be well characterized by properties in its critical (stationary) points, CPs, where the gradient of the density function vanishes. Hessian, the matrix of second derivatives, shows the CPs kind, which can be a local density maximum, a minimum only along the bond, a maximum only along the normal to the ring plane, or a local minimum, defining the nuclear (NCP), bond (BCP), ring (RCP), or molecular cage (CCP) critical point, respectively.

In the late 1990s, Howard and Krygowski first demonstrated that in the RCP of rings in benzenoid hydrocarbons, the charge density, Laplacian, and especially that Hessian eigenvalue, which describes charge density curvature in the direction normal to the ring’s plane, linearly correlate with the HOMA index.^32^ They also established that the charge density, Laplacian, and ellipticity in CC bond BCPs significantly correlate with the CC bond lengths. Soon thereafter, O’Brien and Popelier made evident that although for homogeneous sets of molecules, the correlations exist, consideration of over 700 diverse BCPs proved that, in general, the “*BCP properties cannot be trivially recovered or even predicted by knowledge of bond length alone*”.^33^ The total, potential, and kinetic electron energy densities at the RCP were then correlated with the HOMA and various NICS indices by Palusiak and Krygowski, which convinced that those parameters might serve as quantitative characteristics of π-electron delocalization.^34^ Thus, the observable-based HOMA aromaticity index correlates well with RCP’s observable-based electron density parameters.^32,34^

In the mid-2010s, we revealed that the HOMA(·) function defined on variables such as the electron density, potential, and kinetic energy component at BCPs perfectly correlates nonlinearly with the standard HOMA index.^20^ Moreover, the indices also work for acyclic hydrocarbons and even for alkanes; thus, they are not only the aromaticity indices but also express a general property of the molecules.^7,20,21^ Although the correlation is excellent for most carbocyclic systems, separate trends exist for the strained cyclic systems, linear allenes, and polyynes.^21^ We finally found that the mathematical form of the standard HOMA index expresses geometrical similarity to the archetypal aromatic benzene.^8^ Analogously, if the HOMA index operates on a nongeometrical variable, it expresses similarity to benzene according to this very variable.

#### 2.3.1 HOMA Based on the Electron Density Parameters at BCPs

Here, the AIM parameters in BCPs and RCPs were obtained for all considered structures (Figure 1) with different functionals (Tables S1–S3). They were inserted into the HOMA equation (eq 1). Comparison of the same types of HOMA­(AIM/BCP) calculated with different functionals revealed deviations (Figure S1). The aberrations occur for molecules that are strained (53, 54), charged (8, 11, 19, 21), or contain a triple bond (32, 41, 44, 56, 57, 65, 73). They are likely caused by functional differences and their technical defects. Therefore, the HOMA­(AIM/BCP) vs HOMA­(*R*) correlations were analyzed using only the most popular B3LYP functional (Figure 2).

**Figure 1:**
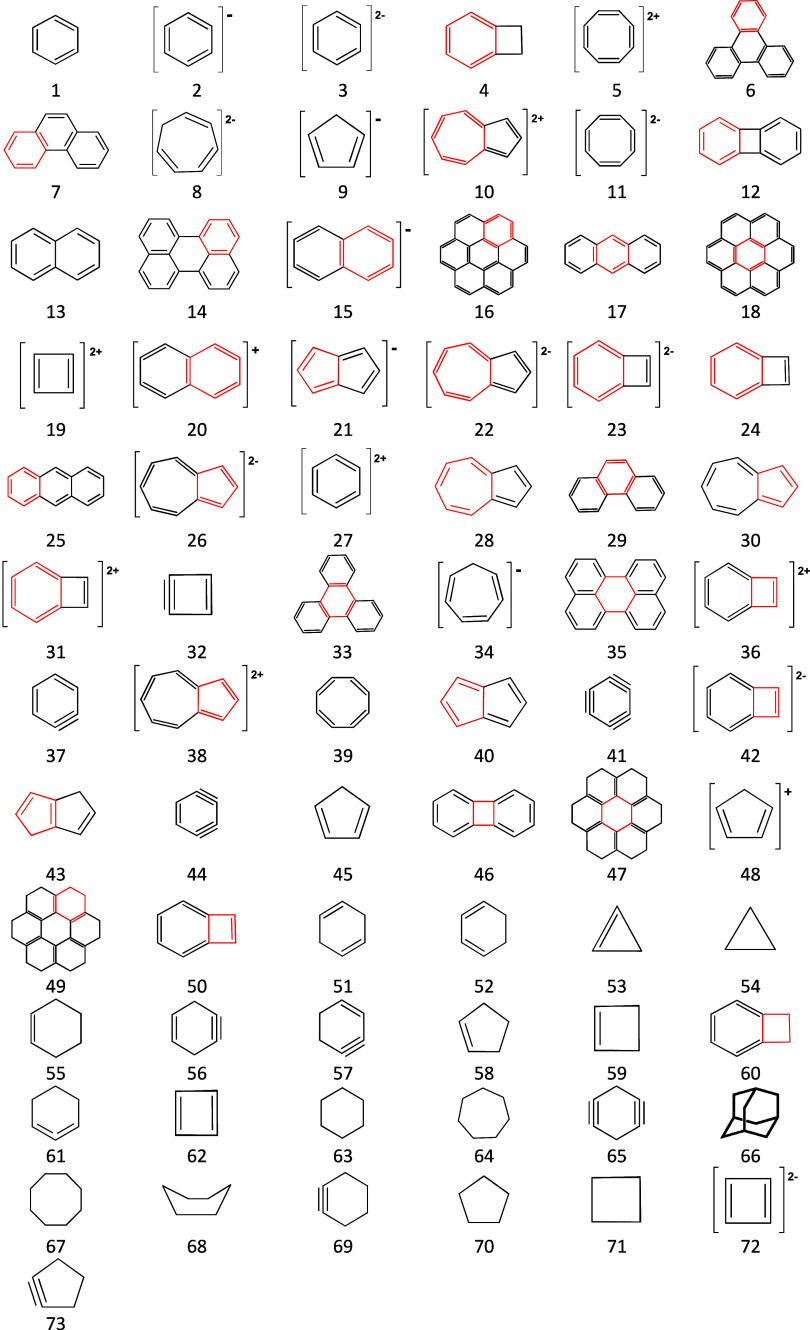
Structural formulas of the considered carbocyclic structures ordered according to decreasing HOMA­(*R*) index calculated at the B3LYP/D3/aug-cc-pVTZ level. In multiring systems, the ring of interest is shown in red.

**Figure 2:**
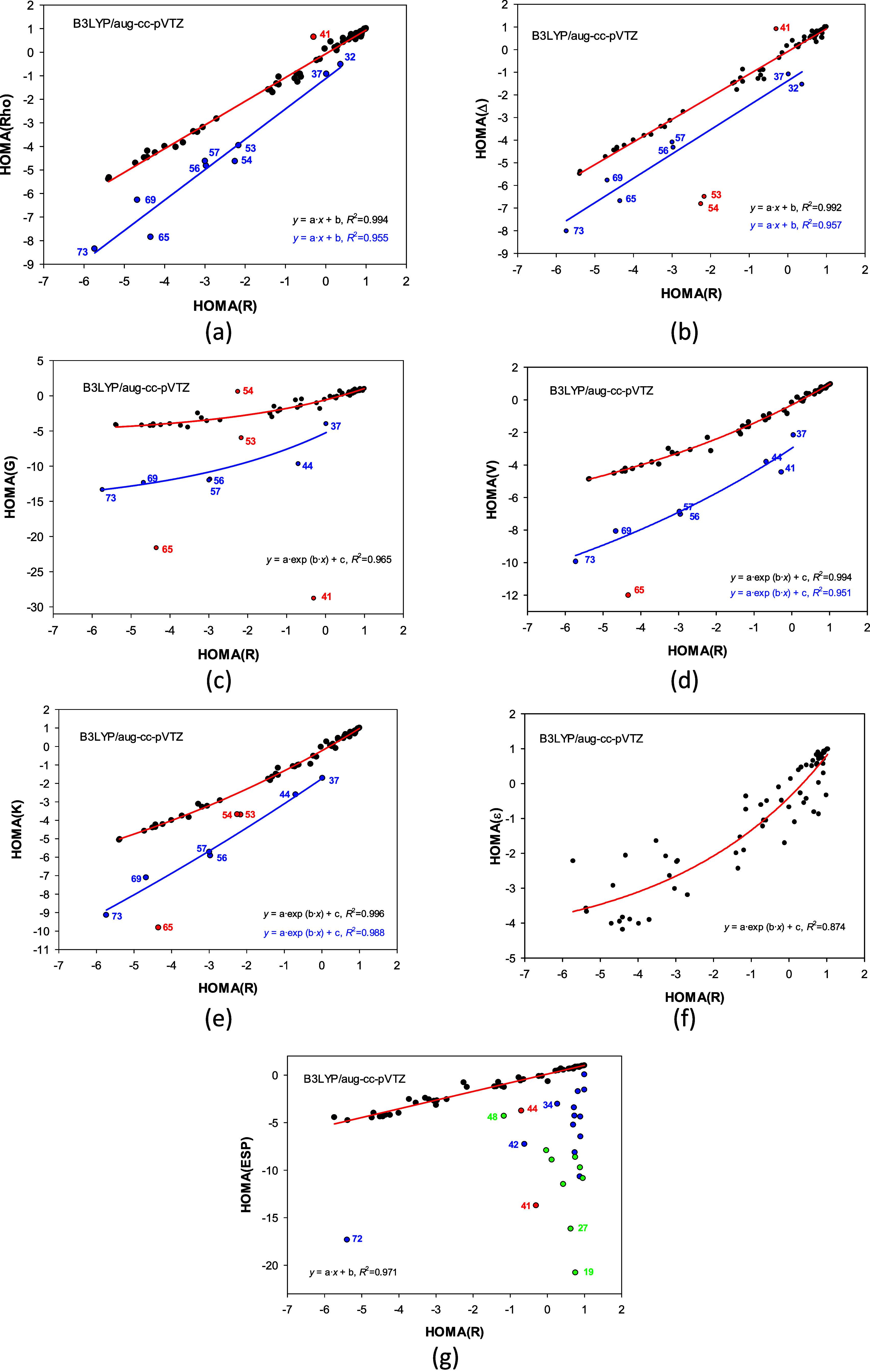
Correlations between the HOMA­(*R*) and HOMA­(AIM/BCP) indices: (a) Rho electron density, (b) Δ Laplacian of Rho, (c) *G* Lagrangian form of kinetic energy density, (d) *V* potential energy density, (e) *K* Hamiltonian form of kinetic energy density, (f) ε ellipticity (molecules 53 and 54 were omitted), and (g) ESP total electrostatic potential. Black and blue points form separate trends in (a–e). Points in red were excluded from the correlations. In (g), blue and green points denote anions and cations, respectively.

In showing the correlation charts below, we first tested linear correlation model unless data distribution suggested a nonlinear relationship. The slot of the linear regression correlation shows whether the dependent variable is more or less sensitive to changes than the independent one. When the data distribution suggested a nonlinear relationship, we first tested the simplest nonlinear model, *i.e*., minimizing the number of fitting parameters and simultaneously maximizing the correlation coefficient. Considering a dozen correlations of the HOMA quadratic-type function defined on different variables, we did not know what regressions one should expect, although a deep physical analysis would probably lead to some guesses. Therefore, we used either linear or the simplest (effective) nonlinear model without judging what this correlation is in its physical essence. We treat the claims about the linearity/nonlinearity of the correlation pragmatically in the context of whether a parameter can be used to measure aromaticity in the sense of HOMA and whether it provides new information compared to HOMA­(*R*). The fit parameters for all correlations presented in the manuscript are in Table S5.

Most of the HOMA­(AIM/BCP) vs HOMA­(*R*) plots (Figure 2) share three features: (i) there is a strong correlation between the most points; (ii) about ten points deviate from the correlation, and (iii) these points are mostly the same as those for which the three functionals disagree. There are also some differences: (i) the correlations are sometimes linear (Figure 2a, b, and g) and sometimes not (Figure 2c, d, e, and f), (ii) the spread pattern around the correlation line varies with AIM parameter and individual points may strongly deviate but may also almost fit the trend (e.g., 41 in Figure 2a and c), (iii) correlation with ellipticity is not strong and the points are much more scattered (Figure 2f), and for total electrostatic potential most points deviate from the correlation (Figure 2g).

The most troubling feature in common is that the outliers are mostly the same as those for which the three functionals disagree (Figures 2 and S1). Indeed, a comparison of the functionals’ work may suggest that the outliers should be discarded, but the HOMA­(AIM) and HOMA­(*R*) correlations reveal that they show specificities of some compounds. Yet, both factors can appear simultaneously: The structures for which the functionals do not work correctly are the same, for which the AIM parameters signal their peculiarity.

Among the outliers (32, 37, 41, 53, 54, 56, 57, 65, 69, and 73, Figure 2a–e), only structures 53 and 54 lack a triple bond but have a 3-membered ring. If they and structures 41 and 65 were omitted, the remaining carbocyclic-ynes cluster around separate correlation lines (Figure 2a–e). We had already observed such behavior when we first noticed that the HOMA/HOMA­(AIM) indices also work for acyclic compounds.^20,21^ We then recognized separate trends for oligoallenes, polyynes, and various geometric isomers of polyenes. Here, we consider a not-large set of primarily single or doubly condensed carbocyclic rings. Therefore, it is not easy to find a homogeneous series of structures and demonstrate the existence of correlations. However, the situation is entirely analogous.

Ellipticity at BCP, ε = (λ_1_/λ_2_ – 1), where λ_1_ < λ_2_ < 0 are negative eigenvalues of the Hessian, shows the electron density distortion in directions 1 and 2 perpendicular to the bond axis 3. Large ε denotes significant π-double bond character, while small shows cylindrical charge distribution typical for a single or a triple bond. Ellipticities are much larger at benzene’s CC BCPs than at cycloalkane or carbocyclic-yne ones but smaller than at double bonds of cycloalkenes. Then, the HOMA­(*R*) vs HOMA­(ε) correlation has a clear physical meaning (Figure 2f); it is significant, has no outliers, yet is weaker than those of separate trends detected in Figure 2a–e.

The HOMA­(*R*) vs HOMA­(ESP) correlation, where ESP denotes total electrostatic potential in BCP, is quite satisfactory (*R* ^2^ = 0.971), but the number of outliers is significant (Figure 2g). Nevertheless, all deviating points, except 41 and 44, correspond to charged molecules. Hence, it is apparent why their HOMA­(ESP) values differ from those of neutral molecules. With more of these types of molecules, it could be possible to separate additional individual trends; still, such a task goes beyond this project.

However, since HOMA­(*X*) is a linear function of the mean squared errors, MSE­(X), which have the bias, EN­(*X*), and variance, GEO­(*X*), components, it could be essential to show not only how the overall MSE correlates but also how the bias and variance factors do. To this aim, for all correlations presented in Figure 2a–g, we plotted graphs reflecting how EN­(*X*) and GEO­(*X*) change with the change of EN­(*R*) and GEO­(*R*) and how EN­(*X*) and GEO­(*X*) contribute to HOMA­(*X*) (Figure S7). Figure S7 shows that for the large and diversified set of rings, neither of the two components alone determines HOMA­(*X*).

Nevertheless, for some rings, the variance of *X*, GEO­(*X*), varies stronger than the variance of bond distance, GEO­(*R*), while biases vary pretty much the same (*e.g*., Figures S7b4 and S7b4). In these cases, the GEO­(*X*) is responsible for the side trends in Figure 2a. The more negative HOMA­(*R*) is, the more the variance component skews away from the straight line, while the bias maintains the primary trend. This means that a significant variance in bond length causes an even larger variance in the electrical parameters of AIM in BCP. Plots in Figure S7 showing the EN­(*X*) and GEO­(*X*) contribution to HOMA­(*X*) look very similar at first glance; however, a closer look reveals their relatively different spread. Thus, different variables disclose diverse aspects of the ring’s aromaticity in terms of HOMA similarity to the benzene ring. Still, the sole decomposition into dependence on bias and variance contributions of the relationships without further structural ring analysis does not help us understand the HOMA­(*X*) vs HOMA­(*R*) relationships.

#### 2.3.2 HOMA Based on the Electron Density Parameters at RCPs

Knowing excellent correlations between HOMA and RCP parameters described so far,^32,34^ it is surprising that the relationship between HOMA­(*R*) and Rho/RCP (Table S3) shown in Figure 3a is chaotic. However, here, an inhomogeneous set of rings is studied: aromatic, nonaromatic, and antiaromatic; aliphatic, containing double and triple bonds; neutral, anionic, and cationic (Figure 1). In contrast, only aromatic rings were considered previously.^32,34^ Juxtaposition of HOMA­(*R*) with HOMA­(Rho/RCP) (greater than – 4500) reveals that points cluster into sets of the same n-membered rings (Figure 3b). Moreover, they disclose significant separate correlations for different ring sizes (Figure 3c–f). Unexpectedly, for the 6-membered rings, the relationship splits into three nearly linear, increasing trends for three kinds of rings (Figure 3c). For the 5-membered rings, it is almost linear but decreasing (Figure 3d), whereas, for the 4-membered rings, it is also decreasing but strongly nonlinear (Figure 3e). Hence, when the HOMA­(*R*) index is used as an arbitrary ring descriptor instead of the aromaticity index, its correlations with the HOMA­(Rho/RCP) expose a series of separate trends mainly diversified by the ring size, but then modified by more subtle ring features. For example, 17, 18, 29, 33, 35, and 47 are central rings in anthracene, coronene, phenanthrene, triphenylbenzene, perylene, and dodecahydrocoronene, respectively (blue line, Figure 3c). After all, different trends for different ring sizes could also be recognized in Figure 3a.

**Figure 3:**
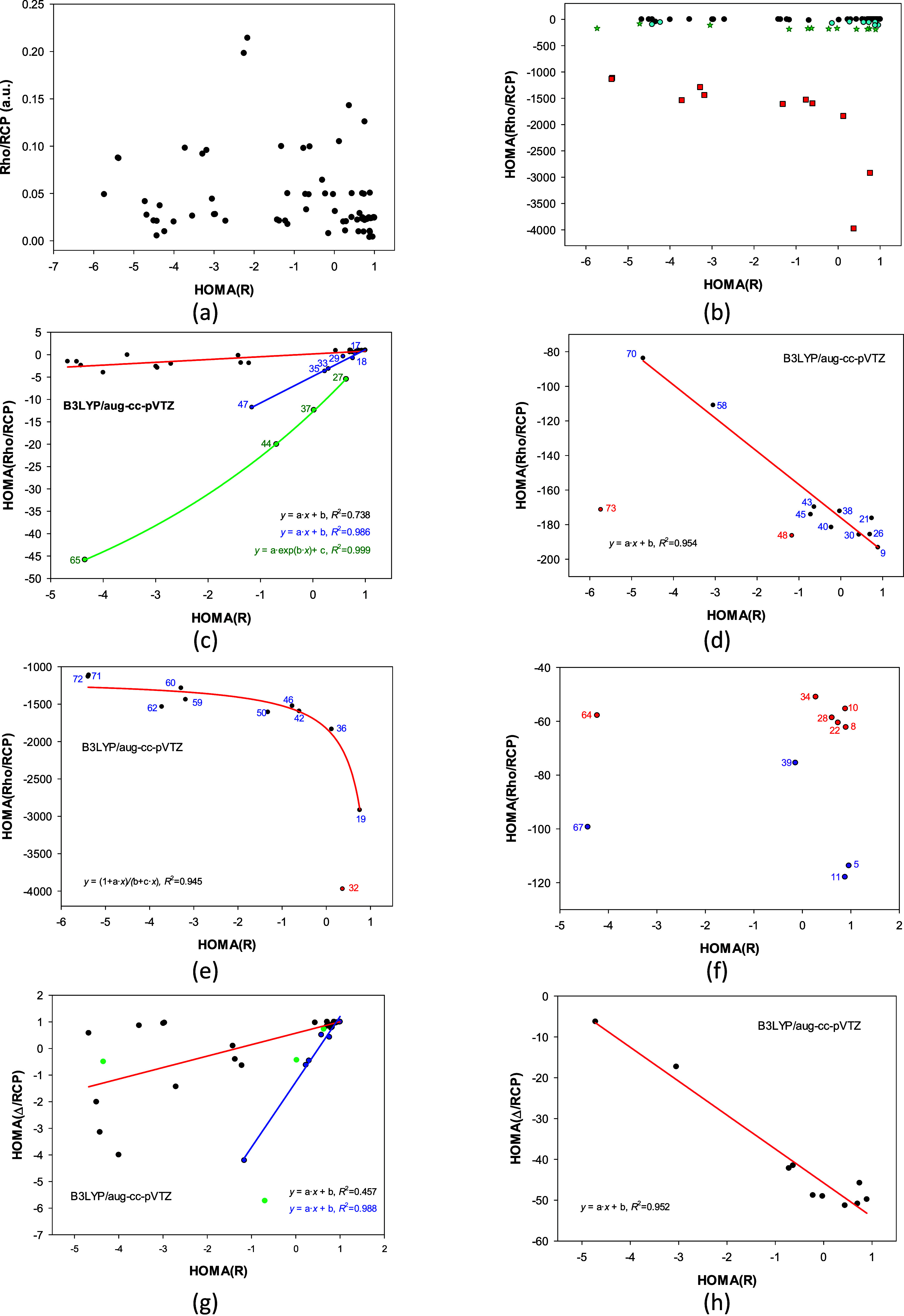
Plot of the HOMA­(*R*) relationship for all structures: (a) vs Rho/RCP and (b) vs HOMA­(Rho/RCP) greater than −4500; black, green, red, and light blue points denote 6-, 5-, 4-, and more than 6-membered rings, respectively. Correlations for: (c) 6-membered rings—different colors represent trends for rings in specific arrangements (see point’s labels), (d) 5-membered rings, (e) 4-membered rings, (f) 7-membered rings (red) and 8-membered rings (blue-violet). Plot of the HOMA­(*R*) relationship with (g) HOMA­(Δ/RCP) for 6-membered rings, and (h) for 5-membered rings.

An analogous performance exhibits the Laplacian Δ at RCPs. No general correlation exists between the HOMA­(*R*) and HOMA­(Δ/RCP) indices. However, a series of separate trends diversified by the ring size, which are modified by some other ring features, can be found (Figure 3g and h). Still, the trends are not identical to those for HOMA­(Rho/RCP), indicating that each RCP AIM parameter can best unveil a specific ring feature. Here, we are investigating the behavior of the universal HOMA index rather than AIM ring properties. Therefore, we do not discuss using the other AIM parameters.

In the case of the AIM parameters in RCP, there is a colossal difference in the pictures showing decompositions into the bias, EN­(*X*), and variance, GEO­(*X*) (Figure S7j and k). This is because these parameters characterize the entire ring with one value: they have no variance (no GEO components). Therefore, all the above statements about correlations tell about the 1-EN­(X/RCP) component.

### 2.4 The Magnetic HOMA Indices

#### 2.4.1 HOMA Based on the ^13^C Shielding Constants

For over 70 carbocyclic rings (Figure 1) optimized with the B3LYP, ωB97XD, and TPSSh functional and the aug-cc-pVTZ basis set, the isotropic σ­(^13^C) shielding constants (chemical shifts) were calculated in the single point runs using the optimized geometries, GIAO approximation, and pcSeg-2 basis set (Table S3). The CC bond-length-based HOMA­(*R*) indices obtained with different functionals show good linear correlations with squared correlation coefficients *R* ^2^ around 0.96 (Figure S2), from which only five rings are distorted from the straight line: benzene dication (4), four-membered ring in benzocyclobutadiene dianion (42), cyclohex-1-en-3,5-diyne (44), cyclohex-1,4-diyne (65), and cyclopent-1-yne (73) (Figure S2). They contain either a triple bond or are significantly strained, and their geometries substantially differ when calculated with the three functionals. The differences are probably caused by technical disparities/defects of functionals rather than by the physical nature of the rings; hence, the five rings were excluded from the correlations. Thus, the three functionals provide a reasonably consistent picture of molecular geometry in terms of the HOMA­(*R*) index. Therefore, it is sufficient to analyze the problem using only one functional. For convenience, we chose the most popular B3LYP functional.

Similarly, the σ­(^13^C) shielding-constants-based HOMA­(σ­(^13^C)) indices obtained with three different functionals display very strong linear correlations (*R* ^2^ ≥ 0.998, Figure S3), from which some rings are outlying: benzene anion (2), benzene dianion (4), cycloheptatriene dianion (9), naphthalene dianion (16), six- and four-membered ring in benzocyclobutadiene dianion (24) and (42), cyclobuta-1-en-3-yne (32), and cyclopropene (54). In the outlying points, the rings are either ions or are significantly strained.

Again, the discrepancy is attributable to the functionals’ incompatibility rather than the rings’ physical nature (Figure S3). Hence, the three functionals yield a congruent picture of magnetic properties through the HOMA­(σ­(^13^C)) index. Analogous reasoning can be given for the indirect ^1^J­(CC) spin–spin coupling constants through the one CC bond. Thus, analyzing the issue using only one B3LYP functional is sufficient.

The HOMA­(*R*) and HOMA­(σ­(^13^C)) indices vary from 1.0 to ca. −6.0 (Table S4). There is no correlation for all structures regardless of the ring size (Figure S5). However, limiting the consideration to only common neutral rings reveals a strong nonlinear correlation between the HOMA indices (*R* ^2^ = 0.939, Figure 4a and b). Moreover, the nonlinear correlation remains significant after adding rings with triple bonds to the common rings (*R* ^2^ = 0.853, Figure 4b). Yet, anionic, cationic, and strained rings substantially deviate from the regression line (Figure S5b).

**Figure 4:**
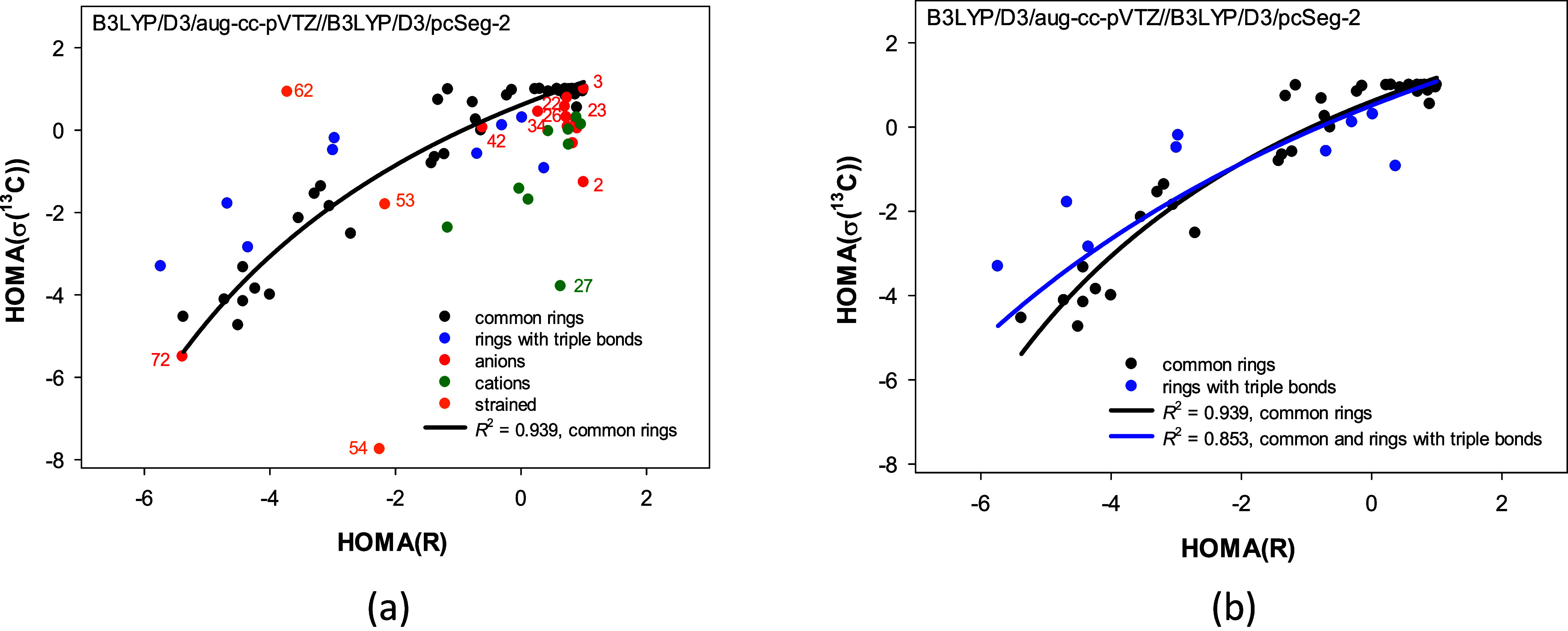
Correlations between the HOMA­(*R*) and HOMA­(σ­(^13^C)) indices calculated with B3LYP functional depend on types of the carbocyclic rings considered: (a) for common neutral carbocyclic rings (black points), (b) for common neutral and rings with triple bonds (black and dark blue points).

The correlations (Figure 4) show that the lower the HOMA­(*R*) index, the lower the HOMA­(σ­(^13^C)) one. Thus, for common neutral carbocyclic rings, the HOMA­(*R*) geometrical similarity of a molecule to benzene is in line with the analogous HOMA­(σ­(^13^C)) magnetic similarity to benzene. This is unsurprising as it agrees with typical ^13^C NMR spectra readings regarding “aromatic” and “aliphatic” signals.

Moreover, it is clear why ions and strained molecules deviate from the correlation (Figure 4a). Chemical shift comprises three factors: dia- and paramagnetic shielding, plus intra- and intermolecular influences. The first shielding is mainly a function of the atom orbitals occupied in the ground state, while the paramagnetic one also depends on the excited electronic states and is essential when *p* or *d* orbitals form the bond. This term roughly depends on the HOMO–LUMO gap, the bond orders, and the average radius of the 2*p* orbitals. Often, it changes dramatically for charged and strained rings, which throws out the corresponding points from the regression line (Figures 4a and S5).

Finally, it should be noted that decomposing the HOMA­(*R*) vs HOMA­(σ­(^13^C)) relationship into the bias and variance components (Figure S7l) does not help us understand it more deeply. Further analysis of the ring types would be needed, but it would hardly provide something more than that done for HOMA alone.

#### 2.4.2 HOMA Based on the ^13^C–^13^C Spin–Spin Coupling Constants through One Bond

The indirect ^13^C–^13^C spin–spin coupling constants are the CC bonds’ vital magnetic attributes. Indeed, the ^1^J­(^13^C–^13^C) in ethyne, ethene, benzene, and ethane equals 174.8, 67.5, 56.0, and 34.5 Hz, respectively, providing a perfect distinction of the CC bond types.^35^ Four contributions determine the spin–spin coupling constant of the CC bonds. The Fermi-contact (FC) term, dominant at short ranges, represents interaction at the nucleus. The paramagnetic and diamagnetic spin–orbit terms, (PSO) and (DSO), represent electrons’ interaction in the nuclei’s potential and often are small and cancel for large internuclear separations. The spin-dipolar (SD) terms become meaningful at a distance.^36^ Thus, ^1^J­(CC)­s are usually controlled by short-range Fermi contact, which requires basis sets with a fair description of the electron density at the nuclei to be correctly reproduced. Thus, two magnetic indices can be formulated (HOMA­(σ­(^13^C)) and HOMA­(^1^J­(CC))); however, the former typifies nuclei while the latter bonds.

The presence of the correlations between HOMA­(*R*), HOMA­(σ­(^13^C)), and HOMA­(^1^J­(CC)) for common neutral carbocyclic rings (Figure 5) shows that the geometrical similarity of a molecule to benzene is in agreement with its magnetic similarity to benzene be it monitored using chemical shifts of ^13^C atoms or indirect spin–spin coupling constants through one CC bond. This also aligns with our daily observations and eliminates the ambiguity of comparing the standard HOMA and NICS-type indices. Interestingly, correlations between HOMA indices based on bond properties, *R* and ^1^J­(CC), are slightly more significant than those of bond and atom attributes: *R* and σ­(^13^C) or ^1^J­(CC) and σ­(^13^C) (Figure 5). As before, decomposing the HOMA­(*R*) vs HOMA­(^1^J­(CC)) relationship into the bias and variance components (Figure S7m) does not help us understand it more deeply without further analysis.

**Figure 5:**
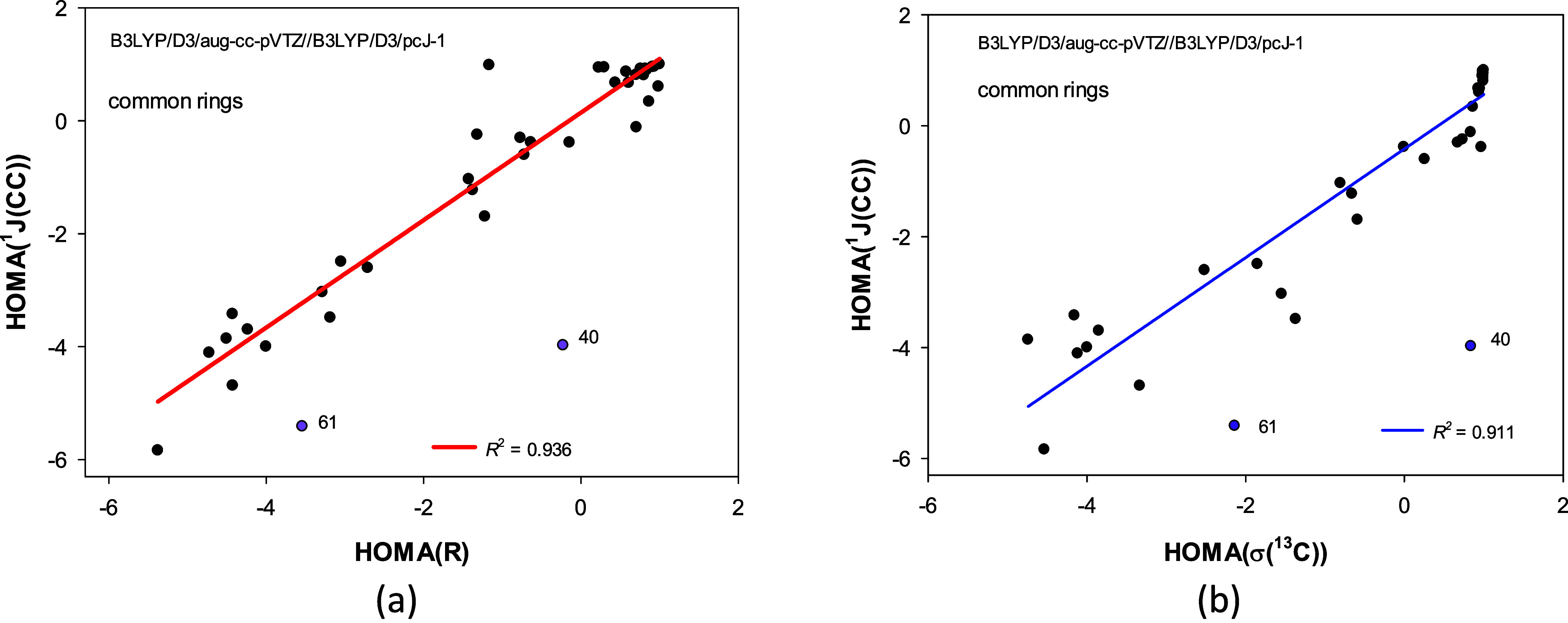
Correlations of the HOMA­(^1^J­(CC)) index with the (a) HOMA­(*R*) and (b) HOMA­(σ­(^13^C)) indices for common neutral carbocyclic rings. Structures of pentalene (40) and cyclohexa-1,2-diene (61) were excluded from the correlations.

Now, consider a similar comparison between the HOMA­(*R*) and HOMA­(σ­(^13^C)) indices and a NICS-type magnetic aromaticity index (Figures 6 and S4). It has long been known that the correlation between the HOMA and NICS-type indices is either nonexistent or weak unless the analysis is restricted to a very regular series of compounds.^37^ The NICS parameter was not designed to analyze compounds without or feeble π-electron structure containing single multiple bond or several π bonds separated by alkyl moieties. Nevertheless, for a while, let us treat the INICS index as a general chemical descriptor, not just one with which only aromaticity is tested. Figure 6a compares the HOMA­(*R*) and INICS values for all the diversified rings studied that are not highly strained or charged.

**Figure 6:**
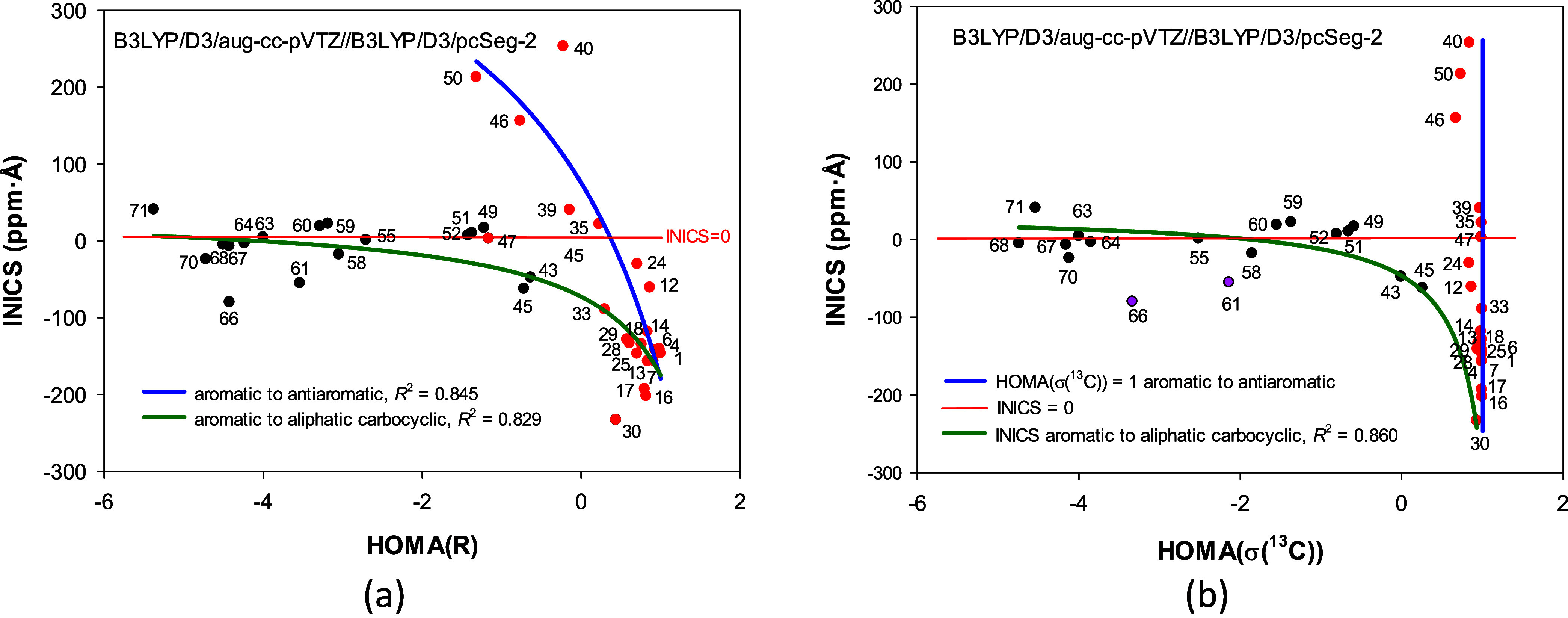
Correlations of the integral INICS index with the (a) HOMA­(*R*) and (b) HOMA­(σ­(^13^C)) indices for common neutral carbocyclic rings representing aromatic, aliphatic, and antiaromatic rings. Structures of azulene (30) and adamantane (66) were omitted in the correlations in Panel a and adamantane (66) and cyclohexa-1,2-diene (61) were excluded from the correlation shown in Panel b.

At first glance, there is no correlation between HOMA­(*R*) and INICS indices (Figures 6, S4, and S6). However, a closer analysis of the points distribution allows us to distinguish two trends. One links alicyclic rings (INICS ≈ 0) to strictly aromatic ones (INICS ≪ 0), and the other links antiaromatic rings (INICS ≫ 0) to purely aromatic ones (INICS ≪ 0, Figure 6a). If one could suppose that these correlations have been manipulated to obtain substantial trends, the graph in Figure 6b makes these trends plausible. In the INICS plot against HOMA­(σ­(^13^C)), the former trend has a smaller scatter of points, and the latter organizes the points around the line vertical to the OX axis at HOMA­(σ­(^13^C)) = 1.

Figure 6b confirms that INICS (and NICS-type)^16,17^ indices are unsuitable for analyzing systems without a delocalized π structure. For them, NICSs approach zero, but, at the same time, HOMA­(*R*) or HOMA­(σ­(^13^C)) are very diversified. The vertical line groups around the π-delocalized structures, and the horizontal divides them into aromatic, nonaromatic, and antiaromatic. For HOMA values from one to zero, some points belong to both trends.

### 2.5 The Universal HOMA Indices as the Aromaticity Descriptors

The universal HOMA indices are primarily concerned with aromaticity rather than the general structural characterization of molecules. Therefore, analyzing the structural, electronic, and magnetic HOMA indices within the range that determines aromaticity is crucial. Rings are aromatic when HOMA indicates their similarity to benzene one, *i.e*., it has a value from 1 to about 0.5. Therefore, further on, we show correlations between the HOMA­(*R*) index and HOMA indices defined on Laplacians Δ in BCPs, ellipticities ε in BCPs, σ­(^13^C) chemical shifts, and ^13^C–^13^C spin–spin coupling constants through one bond ^1^J­(CC) for HOMA­(*R*) limited to the 1 to about 0.0 range (Figure 7).

**Figure 7:**
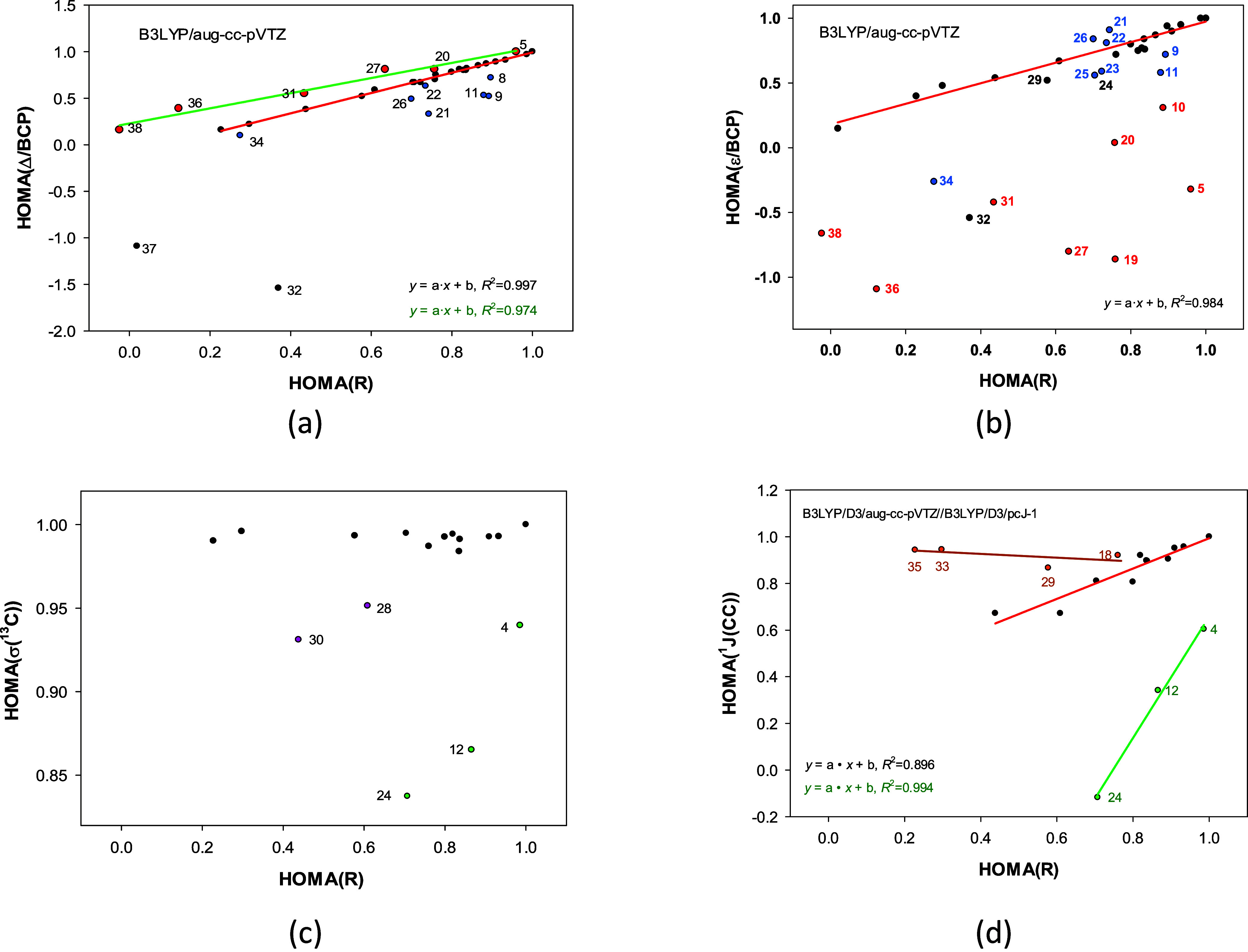
Plot of the HOMA­(*R*) relationship with HOMA indices defined on (a) Laplacians Δ in BCPs, (b) ellipticities ε in BCPs, (c) σ­(^13^C) chemical shifts, and (d) ^13^C–^13^C spin–spin coupling constants through one bond ^1^ *J*(CC). Negatively and positively charged molecules are in blue or red, respectively.

The relationship between HOMA­(*R*) and the HOMA­(Δ/BCP) index defined on Laplacians in BCPs (Figure 7a) reveals an excellent linear correlation (*R* ^2^ = 0.997) where the majority of uncharged rings with HOMA­(*R*) greater than 0.0 belong to. It is accompanied by a very strong linear correlation with a smaller slope, corresponding to positively charged rings (*R* ^2^ = 0.974). The points representing the negatively charged rings are more spread, but if they could been more colinear, the resulting correlation would have a steeper slope. Two strained rings with one triple bond, 32 and 37, are definitely outlying. Thus, HOMA­(*R*) and HOMA­(Δ/BCP) indices can be used to analyze geometric and electronic aspects of aromaticity. However, the latter index differentiates the rings concerning their charge.

The HOMA­(*R*) vs HOMA­(ε/BCP) relationship, where the second index is defined on ellipticities in BCPs (Figure 7b), shows a strong linear correlation (*R* ^2^ = 0.962) for the uncharged rings with HOMA­(*R*) > 0.0. The points representing negatively and positively charged molecules (in blue or red, respectively, Figure 7b) are erratically spread, although the former are positioned at higher HOMA­(ε/BCP) values than the latter. Thus, the HOMA­(ε/BCP) can be used to analyze electronic aspects of the aromaticity of uncharged rings.

The HOMA­(σ­(^13^C)) index is rather useless for evaluating their magnetic facet of aromaticity as most of the neutral molecules with HOMA­(*R*) > 0.0 have HOMA­(σ­(^13^C)) close to 1.0 (Figure 7c). Nevertheless, the HOMA­(^1^ *J*(CC)) index is very promising as it is likely to reveal separate trends for standard carbocyclic rings, rings in the middle of large fused systems (18, 29, 33, 35), and rings fused with 4-membered ring (Figure 7d).

Figure 7 shows that HOMA indices based on the electronic or magnetic properties of the bonds in carbocyclic rings can be successfully used to analyze different aspects of aromaticity. Moreover, electronic and magnetic properties disclose separate trends that are invisible if standard CC bond lengths are used.

### 2.6 A Few Final Remarks

With the help of anonymous reviewer of this paper, we show that HOMA is a kind of mean squared error measure. HOMA connection to MSE is the origin of the index quality, stability, and unshakable robustness of such a measure of aromaticity. This also permits and justifies testing and using HOMA for any physical quantity we think may be related to aromaticity, π-electron delocalization, or a molecular moiety of interesting properties. This also provokes using other statistical measures, like mean absolute error (MAE), etc., to construct modified HOMA indices.

In this work, we have studied only carbocyclic systems, but the task is entirely analogous for heterocyclic systems. However, it requires rationally selecting heterocyclic molecules (or their fragments), allowing the parametrization for chosen heteroatoms. Nevertheless, reference systems may have to be chosen accordingly for each electrical or magnetic variable. Still, this step forward is not trivial and requires careful new research.

The HOMA index is by far the most important in studying aromatic systems. However, we show that the index defined by electrical or magnetic variables can also be used as a general structural descriptor considering the geometry, electronic, or magnetic properties. At present, it is difficult to determine the usefulness range of such studies.

The strength of HOMA-type indices is that they can be defined on observables. This embeds the study of hard-to-define aromaticity in strong experimental principles.

## 3 Conclusions

The HOMA index is one of the most widely used aromaticity descriptors. Now, we know the primary origin of the index quality, stability, and unshakable robustness as a measure of aromaticity: it is a linear function of the mean squared errors (MSE). Its physical meaning is simple: it expresses similarity to the archetypal aromatic benzene ring. The greater the similarity to benzene, the closer the index approaches one, and the lower the similarity, the lower the HOMA value. This structural index is based on observable quantities—bond lengths. In this paper, based on over 70 diversified carbocyclic rings, aromatic, nonaromatic, saturated, differently unsaturated, charged negatively, positively, and neutral, we show that it can be a universal index, determined using many observable physical properties of the ring under study. HOMA connection to MSE strips the index of the mystery it was shrouded in but also permits and justifies testing and using it for any physical quantity we think may be related to aromaticity, π-electron delocalization, or a molecular moiety of interesting properties.

The universal HOMA equation looks like the standard one. However, as a linear function of MSE, it can be defined using arbitrary variables *X* instead of the bond distances *R*. We considered electronic and magnetic atom and bond observables and compared universal HOMA indices constructed using them with the standard HOMA values. The universal HOMA index expresses similarity to the benzene molecule concerning its electronic or magnetic features described by the used property. The strength of HOMA-type indices is that they can be defined using electronic or magnetic observables instead of ephemeric computational quantities like chemical shifts taken at nonstationary points around the molecule. This embeds the study of hard-to-define aromaticity in strong experimental principles.

The universal HOMA definition authorizes using as a variable the rings’ atom, bond, angle, or whole ring properties, such as, e.g., chemical shift, AIM parameter in BCP, spin–spin coupling constant through two bonds, or AIM parameter in RCP, respectively. Here, the following observables were used: the AIM parameters at bond or ring critical points and chemical shifts or spin–spin coupling constants through one bond instead of bond lengths. Correlations between the standard HOMA index and the indices containing these observables show an underlying trend with several deviating points. However, the outliers are not random points but correspond to structures sharing joint features. So, they reveal unique features of specific rings rather than reduce the correlations’ quality.

The vital features were different in the case of electronic and magnetic observables. In the former case, structural factors, such as the presence of a triple bond (BCPs) or the size of the ring (RCPs), come to the fore. As for the standard HOMA index, the molecular charge is of secondary importance for the HOMA­(AIM) indices. On the other hand, in HOMA indices based on magnetic properties, the molecular charge mainly matters. Nevertheless, if analysis can be limited to only neutral species, the HOMA­(^1^J­(CC)) index looks very promising as it discloses separate trends for standard carbocyclic rings, rings in the middle of a large fused system or being fused with the 4-membered ring. On the contrary, the HOMA­(σ­(^13^C)) index is rather useless for evaluating a magnetic facet of aromaticity as for most of the neutral molecules with HOMA­(*R*) > 0.0 it is close to 1.0.

As a side result of the study, we unequivocally demonstrated that there is no correlation between standard HOMA­(*R*) and NICS or HOMA­(*R*) and the AIM parameter in RCP if descriptors are used for inhomogeneous sets of rings, i.e., if the indices are used for a sufficiently diversified set of rings.

## 4 Computations

The calculations were performed by using the Gaussian 16 suite of programs.^38^ The geometries of over 70 carbocyclic rings (Figure 1) were optimized following the DFT^39^ and unrestricted DFT^40^ approaches using three different functionals that perform well for the NMR properties of carbocyclic compounds: the utmost used B3LYP^41–44^ functional with the D3 Grimme’s correction for dispersion forces;^45^ the long-range and empirical atom–atom dispersion corrected ωB97XD^46^ functional; and the local kinetic energy τ-dependent gradient-corrected correlation TPSSh^47–49^ functional. The aug-cc-pVTZ^50,51^ correlation consistent basis set was used because it performs very well for geometry and most standard properties of organic molecules.^52,53^ All optimized geometries exhibited only positive harmonic frequencies, ascertaining that the structures are at true minima. In a few cases, obtaining all positive frequencies required lowering the symmetry of a molecule.

The NMR parameters were determined based on frozen geometries from optimizations with the aug-cc-pVTZ basis set. The NMR shielding constants were calculated using the pcSeg-2 ^54–56^ basis set, specially optimized for good shielding constants reproduction, and the Gauge-Independent Atomic Orbital (GIAO) method.^57,58^ In contrast, the ^13^C–^13^C spin–spin coupling constants^59,60^ were estimated with the pcJ-1 ^61^ basis set optimized for calculations of indirect nuclear spin–spin coupling constants using DFT methods. The pcSeg-2 and pcJ-1 bases were accessed from the Basis Set Exchange software.^62^

The Atoms-in-Molecule (AIM)^63,64^ electron density parameters in bond and ring critical points, BCP and RCP, respectively, were calculated using AIMAll Software.^65^ Here, we consider the Rho (Electron Density), Laplacian of Rho (Trace of Hessian of Rho), *V* (Virial Field = Potential Energy Density), *G* (Lagrangian Form of Kinetic Energy Density), *K* (Hamiltonian Form of Kinetic Energy Density), *H* (*H* = *G* + *V*, Total Energy), and ESP (Total Electrostatic Potential) at CC BCPs and RCPs of all (over 70) carbocyclic rings (Figure 1) optimized with the three functionals and the aug-cc-pVTZ basis set.

Calculations of the HOMA indices and their components were done using the commercial Microsoft Excel program, while correlations were performed using the commercial SigmaPlot for Windows ver. 14.^66^

## Supplementary Material



## Data Availability

The data underlying this study are available in the published article and its Supporting Information.

## Notes

*XYZ* coordinates of all molecules used, AIM parameters in ring and bond critical points, and HOMA values defined on them and ^13^C and ^1^J­(CC) spin–spin coupling constants (PDF)
