# Supplementary material for: The Form of the HOMA Geometric Aromaticity Index Is Universal and Allows the Design of Electronic and Magnetic Descriptors
Source: J Org Chem. 2025 Jun 17;90(25):8674–86. doi: 10.1021/acs.joc.5c00740 (PMC12210268; doi:10.1021/acs.joc.5c00740)
Supplement: Supplementary file 1 [file jo5c00740_si_001.pdf]

Supplementary Information file to

The Form of the HOMA Geometric Aromaticity Index is Universal and Allows the Design of Electronic and Magnetic Descriptors

Jan Cz. Dobrowolski* and Sławomir Ostrowski

Institute of Nuclear Chemistry and Technology, 16 Dorodna Street, 03-195 Warsaw, Poland

Table of contents:

#	Title	page
1	**Table S1.** The normalization parameters α_X and the reference variable in the benzene ring XB for the HOMA(X) values under assumption that HOMA(XB) ≡ 1 and HOMA(XC) ≡ -4, where B and C stand for benzene and chair cyclohexane molecules, calculated with different functionals: B3LYP, ωB97-XD, and TPSSh, D3 correction for dispersion forces, and the aug-cc-pVTZ basis set.	S3
2	**Figure S1**. Intercorrelations of the HOMA(AIM/BCP) indices calculated in the bond critical points for all considered molecules using the B3LYP, ωB97XD, TPSSH functionals and the aug-cc-pVTZ basis set: (a) ellipticity, (b) Rho electron density, (c) Laplacian of Rho, (d) G Lagrangian Form of Kinetic Energy Density, (e) V potential energy, (f) K Hamiltonian Form of Kinetic Energy Density, (g) ESP Total Electrostatic Potential.	S4
3	**Table S2a.** The AIM parameters in BCPs were obtained for all considered structures (Figure 1) with different functionals: B3LYP, ωB97-XD, and TPSSh and the aug-cc-pVTZ basis set.	S5
4	**Table S2b.** The HOMA (AIM) parameters in BCPs were obtained for all considered structures (Figure 1) with different functionals: B3LYP, ωB97-XD, and TPSSh and the aug-cc-pVTZ basis set.	S14
5	**Table S3.** The HOMA(AIM) parameters in RCPs were obtained for all considered structures (Figure 1) with B3LYP functional.	S18
6	**Table S4**. The HOMA(R) and HOMA($\sigma(^{13}C)$) indices for 73 rings (Figure 1) calculated with the B3LYP/D3, ωB97-XD, and TPSSh functionals based on geometries optimiized with the aug-cc-pVTZ basis set, and the isotropic $\sigma(^{13}C)$ shielding constants calculated with the pcSeg-2 basis set.	S20
7	**Table S5.** The fit parameters for all correlations presented in the manuscript. R2 is reflecting the percentage of the dependent variable variation that the used model explains, coefficient value,σ standard error, t Student's t-test value, P values indicate whether the for the coefficients in the elationships are statistically significant.	S22
8	**Figure S2.** Linear correlations between the HOMA(R) indices calculated using the three DFT functions. The outlying points were excluded: four-membered ring in benzocyclo-butadiene dianion (42), cyclohex-1-en-3,5-diyne (44), cyclohex-1,4-diyne (65), and cyclopent-1-yne (73).	S23
9	**Figure S3.** HOMA($\sigma(^{13}C)$) indices obtained with different functionals display linear correlations. The outlying points were excluded: benzene anion (2), benzene dianion (27), cycloheptatriene dianion (8), naphthalene dianion (15), six- and four-membered ring in benzocyclobutadiene dianion (23) and (42), cyclobuta-1-en-3-yne (32), and cyclopropene (54).	S23
10	**Figure S4.** Correlations between the HOMA(R) and HOMA($\sigma(^{13}C)$) and NICS(-1) indices, a) and b), respectively calculated with B3LYP functional depend on types of the carbocyclic rings considered for common neutral carbocyclic rings devided to aromatic, aliphatic, and antiaromaetic rings.	S24
11	**Figure S5.** Correlations between the HOMA(R) and HOMA($\sigma(^{13}C)$) indices calculated with B3LYP functional depend on types of the carbocyclic rings considered: a) rings of different size, b) anionic, cationic, and strained rings, and the correlation line for the common rings and rings with triple bonds.	S24

12	**Figure S6.** Correlations between the HOMA(R) and HOMA(σ(^{13}C)) and NICS(-1) indices, a) and b) respectively, calculated with B3LYP functional depend on types of the carbocyclic rings considered for all types of rings considered	S26
13	**Illustration of the role of the bias (EN) and variance (GEO) components in the correlations presented in the manuscript**	S27
14	**Figure S7.** The relationships between the HOMA(R), 1-EN(R), and 1-GEO(R) and analogous parameters of X variables (see Table S4) for the optimized molecules from Figure 1 calculated using B3LYP functional and the aug-cc-pVTZ, (optimization and AIM parameters) the pcSeg−2 (^{13}CNMR)and pcJ−1 (^{1}J(CC)) basis and sets.	S28
14	**Table S6.** The HOMA, EN, and GEO values for the optimized molecules from Figure 1 calculated using B3LYP functional and the aug-cc-pVTZ, (optimization and AIM parameters) the pcSeg−2 (13CNMR)and pcJ−1 (1J(CC)) basis and sets.	S34
15	**Table S7.** Cartesian xyz coordinates for optimized molecules from Figure 1 calculated using B3LYP, ωB97-XD, and TPSSh methods and the aug-cc-pVTZ basis set.	S39

Table S1. The normalization parameters α_X and the reference variable in the benzene ring X_B for the HOMA(X) values under assumption that HOMA(X_B) ≡ 1 and HOMA(X_C) ≡ -4, where B and C stand for benzene and chair cyclohexane molecules, calculated with different functionals: B3LYP, ωB97-XD, and TPSSh, D3 correction for dispersion forces, and the aug-cc-pVTZ basis set.

HOMA parameter	Computational method					
	B3LYP/D3/aug-cc-pVTZ		ωB97XD/D3/aug-cc-pVTZ		TPSSh/D3/aug-cc-pVTZ	
	α_X	X_B	α_X	X_B	α_X	X_B
**HOMA(R)**	250.808000	1.3913E+00	254.217564	1.3872E+00	253.253500	1.3920E+00
**HOMA(Rho/BCP)**	845.2360	3.2296E-01	863.1581	3.2371E-01	899.3950	3.2037E-01
**HOMA(Δ/BCP)**	23.4829	-1.0699E+00	23.9586	-1.0721E+00	25.6180	-1.0502E+00
**HOMA(V/BCP)**	111.2030	-4.7877E-01	110.5982	-4.8333E-01	115.8832	-4.7597E-01
**HOMA(G/BCP)**	2139.4550	1.0565E-01	2064.8699	1.0766E-01	2113.8000	1.0671E-01
**HOMA(K/BCP)**	186.5810	3.7312E-01	187.2339	3.7567E-01	197.5715	3.6926E-01
**HOMA(L/BCP)**	375.7265	2.6746E-01	383.3404	2.6801E-01	409.8880	2.6255E-01
**HOMA(ε/BCP)**	144.7867	1.8976E-01	139.3438	1.9309E-01	128.0570	2.0070E-01
**HOMA(ESP/BCP)**	74.4044	8.6197E-01	72.5802	8.6974E-01	73.9683	8.6280E-01
**HOMA(ESPe/BCP)**	14.4253	-1.5609E+01	13.9057	-1.5647E+01	13.8815	-1.5597E+01
**HOMA(ESPn/BCP)**	46.0506	1.6471E+01	43.9821	1.6517E+01	43.2098	1.6460E+01
**HOMA(Rho/RCP)**	282370	2.4246E-02				
**HOMA(Δ/RCP)**	2756.995	1.51809- 01				
**HOMA(^{13}C NMR)**	0.000451	4.5705E+01	0.000422	4.9726E+01	0.000489	5.3997E+01
**HOMA(SPIN)**	0.006300	6.3090E+01	0.007278	5.6818E+01	0.007279	5.6707E+01

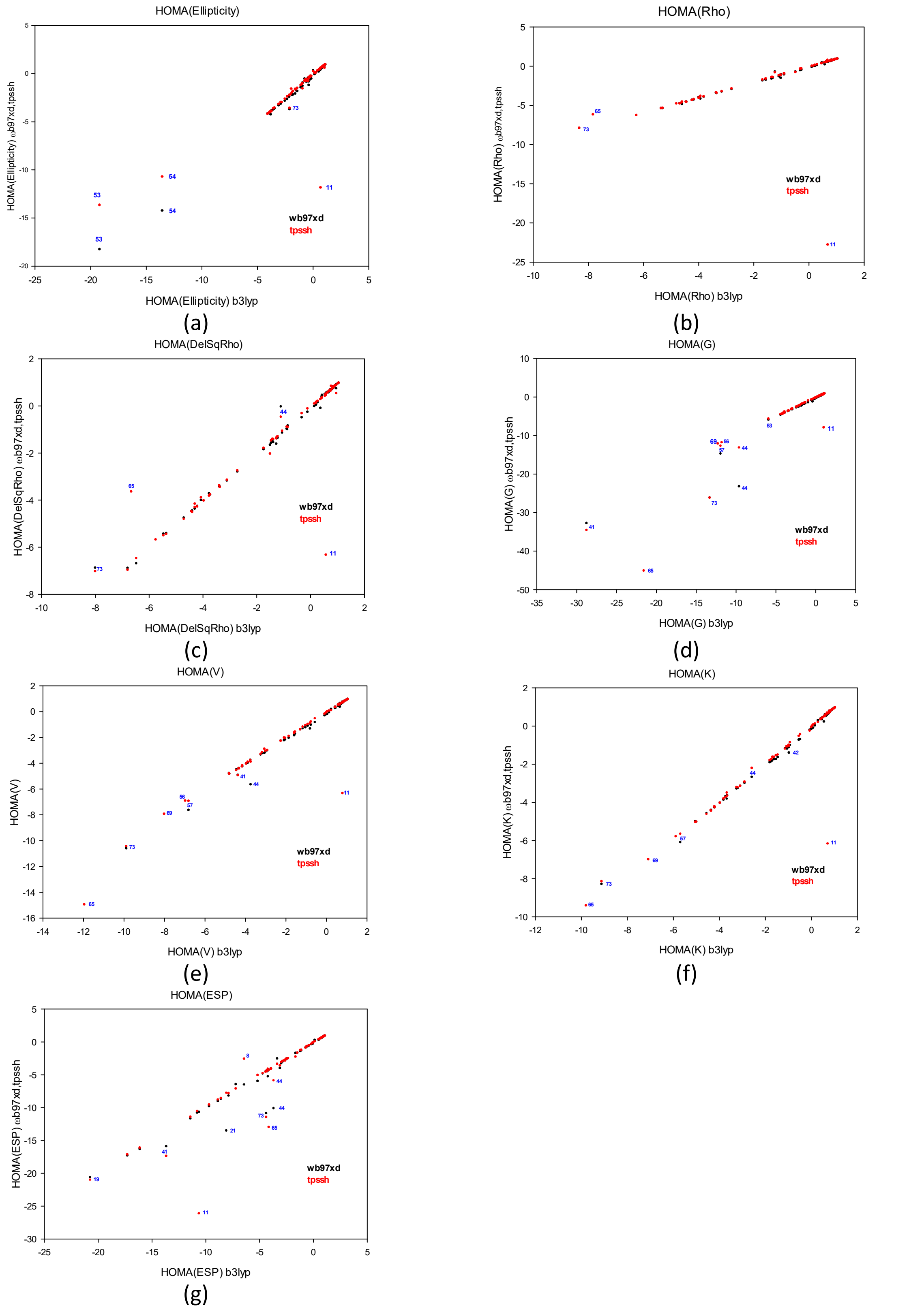

Figure S1. Intercorrelations of the HOMA(AIM/BCP) indices calculated in the bond critical points for all considered molecules using the B3LYP, ωB97XD, TPSSH functionals and the aug-cc-pVTZ basis set: (a) ellipticity, (b) Rho electron density, (c) Laplacian of Rho, (d) G Lagrangian Form of Kinetic Energy Density, (e) V potential energy, (f) K Hamiltonian Form of Kinetic Energy Density, (g) ESP Total Electrostatic Potential.

Table S2a. The AIM parameters in BCPs were obtained for all considered structures (Figure 1) with different functionals: B3LYP, ωB97-XD, and TPSSh and the aug-cc-pVTZ basis set.

B3LYP																
	Ellipticity								Rho							
#	BCP1	BCP2	BCP3	BCP4	BCP5	BCP6	BCP7	BCP8	BCP1	BCP2	BCP3	BCP4	BCP5	BCP6	BCP7	BCP8
1	1.898E-01	1.898E-01	1.898E-01	1.898E-01	1.898E-01	1.898E-01			3.230E-01	3.230E-01	3.230E-01	3.230E-01	3.230E-01	3.230E-01		
2	1.864E-01	1.864E-01	1.864E-01	1.864E-01	1.864E-01	1.864E-01			3.231E-01	3.231E-01	3.232E-01	3.231E-01	3.232E-01	3.231E-01		
3	1.863E-01	1.863E-01	1.863E-01	1.902E-01	1.863E-01	1.902E-01			3.226E-01	3.226E-01	3.226E-01	3.227E-01	3.226E-01	3.227E-01		
4	1.933E-01	1.949E-01	1.927E-01	1.975E-01	1.927E-01	1.949E-01			3.322E-01	3.266E-01	3.167E-01	3.184E-01	3.167E-01	3.266E-01		
5	9.437E-02	9.440E-02	9.438E-02	9.440E-02	9.438E-02	9.440E-02	9.440E-02	9.438E-02	3.181E-01	3.181E-01	3.181E-01	3.181E-01	3.181E-01	3.181E-01	3.181E-01	3.181E-01
6	1.713E-01	1.739E-01	2.091E-01	1.693E-01	2.091E-01	1.713E-01			3.126E-01	3.101E-01	3.318E-01	3.206E-01	3.318E-01	3.126E-01		
7	1.597E-01	2.159E-01	1.622E-01	1.654E-01	1.673E-01	2.144E-01			3.067E-01	3.333E-01	3.129E-01	3.163E-01	3.120E-01	3.315E-01		
8	2.100E-01	2.093E-01	2.108E-01	2.103E-01	2.100E-01	2.093E-01	2.103E-01		3.066E-01	3.069E-01	3.060E-01	3.063E-01	3.066E-01	3.068E-01	3.062E-01	
9	2.334E-01	2.334E-01	2.334E-01	2.334E-01	2.334E-01				3.047E-01	3.047E-01	3.047E-01	3.047E-01	3.047E-01			
10	1.266E-01	8.689E-02	1.266E-01	1.798E-01	8.689E-02	1.262E-01	1.798E-01		3.238E-01	3.093E-01	3.238E-01	3.378E-01	3.093E-01	3.109E-01	3.378E-01	
11	2.434E-01	2.434E-01	2.434E-01	2.434E-01	2.434E-01	2.434E-01	2.434E-01	2.434E-01	3.027E-01	3.027E-01	3.027E-01	3.027E-01	3.027E-01	3.027E-01	3.027E-01	3.027E-01
12	2.201E-01	1.593E-01	1.669E-01	2.252E-01	1.669E-01	2.252E-01			3.265E-01	3.153E-01	3.064E-01	3.355E-01	3.064E-01	3.355E-01		
13	2.287E-01	1.421E-01	1.498E-01	1.541E-01	1.498E-01	2.287E-01			3.358E-01	3.029E-01	3.090E-01	3.102E-01	3.090E-01	3.358E-01		
14	1.503E-01	1.473E-01	2.205E-01	1.511E-01	1.669E-01	2.115E-01			3.028E-01	3.011E-01	3.369E-01	3.112E-01	3.166E-01	3.264E-01		
15	2.259E-01	1.434E-01	1.512E-01	1.476E-01	1.512E-01	2.259E-01			3.352E-01	3.025E-01	3.075E-01	3.103E-01	3.075E-01	3.352E-01		
16	1.455E-01	1.454E-01	1.572E-01	2.385E-01	1.572E-01	1.454E-01			3.063E-01	3.070E-01	3.102E-01	3.383E-01	3.102E-01	3.071E-01		
17	1.251E-01	1.860E-01	1.860E-01	1.864E-01	1.250E-01	1.864E-01			2.961E-01	3.215E-01	3.215E-01	3.218E-01	2.960E-01	3.218E-01		
18	1.455E-01	1.455E-01	1.455E-01	1.455E-01	1.455E-01	1.455E-01			3.063E-01	3.063E-01	3.063E-01	3.063E-01	3.063E-01	3.063E-01		
19	7.632E-02	7.638E-02	7.638E-02	7.631E-02					3.073E-01	3.074E-01	3.074E-01	3.073E-01				
20	6.808E-02	1.370E-01	1.295E-01	1.808E-01	1.295E-01	6.808E-02			3.024E-01	3.050E-01	3.202E-01	3.413E-01	3.202E-01	3.024E-01		
21	2.212E-01	2.334E-01	1.866E-01	1.759E-01	1.858E-01				3.011E-01	3.054E-01	3.038E-01	2.933E-01	2.971E-01			
22	1.881E-01	2.289E-01	1.881E-01	1.533E-01	2.289E-01	1.289E-01	1.533E-01		3.106E-01	3.260E-01	3.106E-01	3.041E-01	3.260E-01	2.806E-01	3.041E-01	
23	1.190E-01	2.527E-01	1.677E-01	2.527E-01	2.437E-01	1.677E-01			3.014E-01	3.347E-01	2.973E-01	3.347E-01	3.249E-01	2.973E-01		
24	1.420E-01	2.556E-01	1.466E-01	2.556E-01	2.485E-01	1.466E-01			3.146E-01	3.416E-01	2.966E-01	3.416E-01	3.331E-01	2.966E-01		
25	1.359E-01	1.251E-01	2.434E-01	1.394E-01	2.434E-01	1.359E-01			3.037E-01	2.961E-01	3.401E-01	3.046E-01	3.401E-01	3.037E-01		
26	1.289E-01	2.151E-01	2.088E-01	2.151E-01	2.088E-01				2.806E-01	3.130E-01	3.103E-01	3.130E-01	3.103E-01			
27	1.649E-01	1.649E-01	6.600E-05	1.649E-01	1.649E-01	2.800E-05			3.202E-01	3.203E-01	2.933E-01	3.202E-01	3.202E-01	2.932E-01		
28	1.855E-01	1.821E-01	1.855E-01	1.772E-01	1.821E-01	6.428E-02	1.772E-01		3.211E-01	3.214E-01	3.211E-01	3.266E-01	3.214E-01	2.657E-01	3.266E-01	
29	1.597E-01	1.214E-01	1.247E-01	2.590E-01	1.247E-01	1.597E-01			3.067E-01	2.879E-01	3.008E-01	3.471E-01	3.008E-01	3.067E-01		
30	6.428E-02	1.959E-01	1.969E-01	1.959E-01	1.969E-01				2.657E-01	3.172E-01	3.166E-01	3.172E-01	3.166E-01			
31	2.313E-02	1.278E-01	1.428E-01	1.278E-01	5.244E-02	1.428E-01			2.791E-01	3.279E-01	3.252E-01	3.279E-01	2.919E-01	3.252E-01		
32	1.978E-01	8.483E-02	1.291E-02	1.978E-01					2.903E-01	3.056E-01	2.541E-01	2.903E-01				
33	1.069E-01	1.739E-01	1.739E-01	1.069E-01	1.069E-01	1.739E-01			2.830E-01	3.101E-01	3.101E-01	2.830E-01	2.830E-01	3.101E-01		
34	2.566E-01	1.499E-01	2.563E-01	3.242E-01	1.502E-01	7.646E-02	3.241E-01		3.163E-01	2.831E-01	3.162E-01	3.474E-01	2.832E-01	2.658E-01	3.473E-01	
35	1.503E-01	9.342E-02	1.503E-01	1.503E-01	9.342E-02	1.503E-01			3.028E-01	2.773E-01	3.028E-01	3.028E-01	2.773E-01	3.028E-01		
36	8.092E-02	9.457E-02	2.313E-02	9.457E-02					3.027E-01	3.106E-01	2.791E-01	3.106E-01				
37	1.658E-02	1.471E-01	2.193E-01	1.469E-01	2.193E-01	1.950E-01			4.362E-01	3.086E-01	3.088E-01	3.085E-01	3.088E-01	3.160E-01		
38	1.262E-01	3.168E-02	1.482E-01	3.168E-02	1.482E-01				3.109E-01	2.737E-01	3.306E-01	2.737E-01	3.306E-01			
39	5.765E-02	3.302E-01	5.766E-02	5.765E-02	3.302E-01	3.302E-01	3.302E-01	5.765E-02	2.782E-01	3.558E-01	2.782E-01	2.782E-01	3.558E-01	3.558E-01	3.558E-01	2.782E-01
40	8.809E-02	3.111E-01	1.107E-01	7.476E-02	2.677E-01				2.642E-01	3.481E-01	2.914E-01	2.743E-01	3.467E-01			
41	1.031E-01	1.031E-01	1.031E-01	1.031E-01	1.031E-01	1.031E-01			3.437E-01	3.437E-01	3.437E-01	3.437E-01	3.437E-01	3.437E-01		
**42**	2.994E-01	8.082E-02	1.190E-01	8.082E-02					3.275E-01	2.551E-01	3.014E-01	2.551E-01				
43	4.698E-02	3.071E-01	2.749E-01	1.134E-01	3.692E-02				2.543E-01	3.473E-01	3.515E-01	2.839E-01	2.618E-01			
44	3.127E-02	3.720E-02	3.127E-02	2.425E-01	1.256E-01	1.256E-01			4.051E-01	3.364E-01	4.051E-01	2.819E-01	3.045E-01	3.045E-01		
45	4.458E-02	3.058E-01	1.052E-01	4.458E-02	3.058E-01				2.608E-01	3.521E-01	2.789E-01	2.608E-01	3.521E-01			
46	1.593E-01	4.407E-02	4.407E-02	1.593E-01					3.153E-01	2.525E-01	2.525E-01	3.153E-01				
47	9.337E-02	9.337E-02	9.341E-02	9.337E-02	9.341E-02	9.337E-02			2.699E-01	2.699E-01	2.699E-01	2.699E-01	2.699E-01	2.699E-01		
48	2.822E-02	2.704E-01	2.704E-01	8.811E-02	8.808E-02				2.327E-01	3.571E-01	3.571E-01	2.944E-01	2.944E-01			
49	3.226E-01	9.337E-02	1.359E-03	3.225E-01	4.825E-02	4.822E-02			3.483E-01	2.699E-01	2.516E-01	3.483E-01	2.619E-01	2.619E-01		
50	3.316E-01	3.601E-02	1.420E-01	3.601E-02					3.516E-01	2.457E-01	3.146E-01	2.457E-01				
51	3.487E-01	3.487E-01	3.885E-02	3.885E-02	3.885E-02	3.885E-02			3.615E-01	3.615E-01	2.607E-01	2.607E-01	2.607E-01	2.607E-01		
52	8.957E-02	3.138E-01	3.138E-01	3.700E-02	5.800E-05	3.700E-02			2.811E-01	3.566E-01	3.566E-01	2.584E-01	2.452E-01	2.584E-01		
53	6.475E-01	6.480E-01	1.864E-01						2.355E-01	2.355E-01	3.708E-01					
54	5.076E-01	5.076E-01	5.076E-01						2.412E-01	2.412E-01	2.412E-01					
55	3.344E-03	5.822E-03	3.853E-02	3.446E-01	3.332E-03	3.851E-02			2.459E-01	2.470E-01	2.591E-01	3.600E-01	2.458E-01	2.590E-01		
56	1.792E-02	1.479E-01	3.953E-02	1.792E-02	3.953E-02	3.528E-01			2.583E-01	4.547E-01	2.381E-01	2.583E-01	2.381E-01	3.581E-01		
57	1.246E-01	3.968E-02	3.427E-01	1.401E-02	4.682E-02	9.683E-03			4.517E-01	2.710E-01	3.463E-01	2.561E-01	2.534E-01	2.190E-01		
58	4.474E-03	4.473E-03	3.662E-02	3.662E-02	3.398E-01				2.379E-01	2.379E-01	2.568E-01	2.568E-01	3.622E-01			
59	2.989E-02	2.989E-02	3.268E-02	3.462E-01					2.517E-01	2.517E-01	2.263E-01	3.585E-01				
60	1.933E-01	2.193E-02	2.193E-02	2.104E-02					3.322E-01	2.499E-01	2.499E-01	2.234E-01				
61	7.177E-03	4.556E-02	1.395E-02	6.043E-02	2.534E-01	2.454E-01			2.295E-01	2.401E-01	2.286E-01	2.440E-01	3.650E-01	3.659E-01		
62	9.253E-03	9.253E-03	3.768E-01	3.768E-01					2.209E-01	2.209E-01	3.617E-01	3.617E-01				
63	3.931E-03	3.929E-03	3.929E-03	3.931E-03	3.929E-03	3.929E-03			2.460E-01	2.461E-01	2.461E-01	2.460E-01	2.460E-01	2.460E-01		
64	4.415E-03	5.402E-03	2.762E-03	4.415E-03	1.158E-02	2.762E-03	1.158E-02		2.445E-01	2.464E-01	2.437E-01	2.445E-01	2.428E-01	2.437E-01	2.428E-01	
65	1.465E-01	1.465E-01	1.455E-02	1.463E-02	1.463E-02	1.455E-02			4.537E-01	4.537E-01	2.383E-01	2.383E-01	2.383E-01	2.383E-01		
66	8.530E-04	8.530E-04	8.530E-04	8.530E-04	8.530E-04	8.530E-04			2.445E-01	2.445E-01	2.445E-01	2.445E-01	2.445E-01	2.445E-01		
67	2.081E-03	1.801E-03	1.432E-02	1.121E-02	2.092E-03	1.121E-02	1.518E-03	1.434E-02	2.426E-01	2.400E-01	2.436E-01	2.437E-01	2.426E-01	2.437E-01	2.407E-01	2.435E-01
68	4.369E-03	4.369E-03	4.369E-03	6.571E-03	4.369E-03	6.571E-03			2.403E-01	2.403E-01	2.403E-01	2.470E-01	2.402E-01	2.470E-01		
69	1.416E-01	1.505E-02	1.505E-02	8.176E-03	8.175E-03	9.361E-03			4.525E-01	2.567E-01	2.567E-01	2.241E-01	2.241E-01	2.418E-01		
70	3.811E-03	1.739E-03	3.555E-03	5.587E-03	5.240E-03				2.429E-01	2.458E-01	2.439E-01	2.353E-01	2.366E-01			
71	1.042E-02	1.042E-02	1.042E-02	1.042E-02					2.365E-01	2.365E-01	2.365E-01	2.365E-01				
72	1.237E-02	1.237E-02	1.237E-02	1.237E-02					2.360E-01	2.360E-01	2.360E-01	2.360E-01				
73	3.101E-02	3.097E-02	1.685E-02	1.679E-02	1.647E-01				2.217E-01	2.217E-01	2.357E-01	2.358E-01	4.628E-01			
	DelSqRho								V							
#	BCP1	BCP2	BCP3	BCP4	BCP5	BCP6	BCP7	BCP8	BCP1	BCP2	BCP3	BCP4	BCP5	BCP6	BCP7	BCP8
1	-1.070E+00	-1.070E+00	-1.070E+00	-1.070E+00	-1.070E+00	-1.070E+00			-4.788E-01	-4.788E-01	-4.788E-01	-4.788E-01	-4.788E-01	-4.788E-01		
2	-1.071E+00	-1.071E+00	-1.071E+00	-1.071E+00	-1.071E+00	-1.071E+00			-4.794E-01	-4.794E-01	-4.796E-01	-4.794E-01	-4.796E-01	-4.794E-01		
3	-1.067E+00	-1.067E+00	-1.067E+00	-1.069E+00	-1.067E+00	-1.069E+00			-4.777E-01	-4.777E-01	-4.777E-01	-4.782E-01	-4.777E-01	-4.783E-01		
4	-1.114E+00	-1.094E+00	-1.024E+00	-1.035E+00	-1.024E+00	-1.094E+00			-4.986E-01	-4.945E-01	-4.623E-01	-4.668E-01	-4.623E-01	-4.945E-01		
5	-1.065E+00	-1.065E+00	-1.065E+00	-1.065E+00	-1.065E+00	-1.065E+00	-1.065E+00	-1.065E+00	-4.545E-01	-4.545E-01	-4.545E-01	-4.545E-01	-4.545E-01	-4.545E-01	-4.545E-01	-4.545E-01
6	-1.001E+00	-9.796E-01	-1.125E+00	-1.063E+00	-1.125E+00	-1.001E+00			-4.468E-01	-4.378E-01	-5.076E-01	-4.693E-01	-5.076E-01	-4.468E-01		
7	-9.611E-01	-1.134E+00	-1.009E+00	-1.034E+00	-9.994E-01	-1.120E+00			-4.269E-01	-5.124E-01	-4.449E-01	-4.568E-01	-4.445E-01	-5.073E-01		
8	-9.607E-01	-9.626E-01	-9.569E-01	-9.589E-01	-9.608E-01	-9.625E-01	-9.587E-01		-4.361E-01	-4.369E-01	-4.343E-01	-4.352E-01	-4.361E-01	-4.368E-01	-4.351E-01	
9	-9.267E-01	-9.267E-01	-9.267E-01	-9.267E-01	-9.267E-01				-4.368E-01	-4.368E-01	-4.368E-01	-4.368E-01	-4.368E-01			
10	-1.096E+00	-1.009E+00	-1.096E+00	-1.180E+00	-1.009E+00	-9.971E-01	-1.180E+00		-4.742E-01	-4.263E-01	-4.742E-01	-5.218E-01	-4.263E-01	-4.333E-01	-5.218E-01	
11	-9.288E-01	-9.288E-01	-9.288E-01	-9.288E-01	-9.288E-01	-9.288E-01	-9.288E-01	-9.288E-01	-4.308E-01	-4.308E-01	-4.308E-01	-4.308E-01	-4.308E-01	-4.308E-01	-4.308E-01	-4.308E-01
12	-1.082E+00	-1.004E+00	-9.635E-01	-1.149E+00	-9.635E-01	-1.149E+00			-4.930E-01	-4.460E-01	-4.303E-01	-5.270E-01	-4.303E-01	-5.270E-01		
13	-1.145E+00	-9.420E-01	-9.870E-01	-9.959E-01	-9.870E-01	-1.145E+00			-5.214E-01	-4.148E-01	-4.326E-01	-4.384E-01	-4.326E-01	-5.214E-01		
14	-9.404E-01	-9.280E-01	-1.158E+00	-1.002E+00	-1.036E+00	-1.081E+00			-4.147E-01	-4.103E-01	-5.234E-01	-4.387E-01	-4.584E-01	-4.914E-01		
15	-1.141E+00	-9.386E-01	-9.764E-01	-9.967E-01	-9.764E-01	-1.141E+00			-5.197E-01	-4.138E-01	-4.288E-01	-4.386E-01	-4.288E-01	-5.197E-01		
16	-9.665E-01	-9.764E-01	-9.876E-01	-1.160E+00	-9.875E-01	-9.764E-01			-4.237E-01	-4.269E-01	-4.363E-01	-5.300E-01	-4.362E-01	-4.269E-01		
17	-9.032E-01	-1.060E+00	-1.060E+00	-1.062E+00	-9.027E-01	-1.062E+00			-3.945E-01	-4.723E-01	-4.723E-01	-4.731E-01	-3.943E-01	-4.731E-01		
18	-9.665E-01	-9.665E-01	-9.666E-01	-9.665E-01	-9.666E-01	-9.665E-01			-4.237E-01	-4.237E-01	-4.237E-01	-4.237E-01	-4.237E-01	-4.237E-01		
19	-9.569E-01	-9.578E-01	-9.578E-01	-9.568E-01					-4.374E-01	-4.377E-01	-4.377E-01	-4.373E-01				
20	-9.664E-01	-9.635E-01	-1.067E+00	-1.195E+00	-1.067E+00	-9.664E-01			-4.074E-01	-4.171E-01	-4.609E-01	-5.293E-01	-4.609E-01	-4.074E-01		
21	-9.068E-01	-9.317E-01	-9.119E-01	-8.689E-01	-8.903E-01				-4.235E-01	-4.369E-01	-4.238E-01	-4.035E-01	-4.150E-01			
22	-9.923E-01	-1.083E+00	-9.923E-01	-9.643E-01	-1.083E+00	-7.970E-01	-9.643E-01		-4.447E-01	-4.931E-01	-4.447E-01	-4.214E-01	-4.931E-01	-3.578E-01	-4.214E-01	
23	-9.209E-01	-1.132E+00	-9.041E-01	-1.132E+00	-1.065E+00	-9.041E-01			-4.082E-01	-5.297E-01	-4.058E-01	-5.297E-01	-4.920E-01	-4.058E-01		
24	-1.006E+00	-1.180E+00	-9.053E-01	-1.180E+00	-1.118E+00	-9.053E-01			-4.439E-01	-5.521E-01	-4.015E-01	-5.521E-01	-5.161E-01	-4.015E-01		
25	-9.567E-01	-9.032E-01	-1.170E+00	-9.632E-01	-1.170E+00	-9.567E-01			-4.163E-01	-3.945E-01	-5.362E-01	-4.211E-01	-5.362E-01	-4.163E-01		
26	-7.970E-01	-9.826E-01	-9.711E-01	-9.826E-01	-9.711E-01				-3.578E-01	-4.540E-01	-4.481E-01	-4.540E-01	-4.481E-01			
27	-1.056E+00	-1.056E+00	-9.171E-01	-1.056E+00	-1.056E+00	-9.167E-01			-4.913E-01	-4.913E-01	-3.696E-01	-4.913E-01	-4.913E-01	-3.695E-01		
28	-1.064E+00	-1.068E+00	-1.064E+00	-1.107E+00	-1.068E+00	-7.237E-01	-1.107E+00		-4.738E-01	-4.741E-01	-4.738E-01	-4.877E-01	-4.741E-01	-3.124E-01	-4.877E-01	
29	-9.611E-01	-8.550E-01	-9.415E-01	-1.215E+00	-9.415E-01	-9.611E-01			-4.269E-01	-3.711E-01	-4.070E-01	-5.595E-01	-4.070E-01	-4.269E-01		
30	-7.237E-01	-1.017E+00	-1.016E+00	-1.017E+00	-1.016E+00				-3.124E-01	-4.643E-01	-4.637E-01	-4.642E-01	-4.637E-01			
31	-7.955E-01	-1.133E+00	-1.092E+00	-1.133E+00	-8.928E-01	-1.092E+00			-3.364E-01	-5.063E-01	-4.785E-01	-5.063E-01	-3.744E-01	-4.785E-01		
32	-8.061E-01	-8.882E-01	-5.588E-01	-8.061E-01					-4.348E-01	-4.731E-01	-3.460E-01	-4.348E-01				
33	-8.292E-01	-9.796E-01	-9.796E-01	-8.292E-01	-8.292E-01	-9.796E-01			-3.568E-01	-4.378E-01	-4.378E-01	-3.568E-01	-3.568E-01	-4.378E-01		
34	-1.014E+00	-8.289E-01	-1.013E+00	-1.201E+00	-8.294E-01	-7.350E-01	-1.201E+00		-4.732E-01	-3.696E-01	-4.728E-01	-5.699E-01	-3.698E-01	-3.154E-01	-5.697E-01	
35	-9.404E-01	-7.970E-01	-9.404E-01	-9.404E-01	-7.970E-01	-9.404E-01			-4.147E-01	-3.410E-01	-4.147E-01	-4.147E-01	-3.410E-01	-4.147E-01		
36	-9.398E-01	-9.916E-01	-7.955E-01	-9.916E-01					-4.058E-01	-4.341E-01	-3.364E-01	-4.341E-01				
37	-1.769E+00	-9.686E-01	-9.624E-01	-9.677E-01	-9.628E-01	-1.018E+00			-8.875E-01	-4.796E-01	-4.466E-01	-4.792E-01	-4.467E-01	-4.585E-01		
38	-9.971E-01	-7.799E-01	-1.126E+00	-7.799E-01	-1.126E+00				-4.333E-01	-3.246E-01	-5.065E-01	-3.246E-01	-5.065E-01			
39	-8.174E-01	-1.255E+00	-8.174E-01	-8.174E-01	-1.255E+00	-1.255E+00	-1.255E+00	-8.174E-01	-3.430E-01	-5.987E-01	-3.431E-01	-3.431E-01	-5.987E-01	-5.987E-01	-5.987E-01	-3.430E-01
40	-7.096E-01	-1.193E+00	-8.549E-01	-7.794E-01	-1.195E+00				-3.126E-01	-5.700E-01	-3.800E-01	-3.409E-01	-5.683E-01			
41	-1.008E+00	-1.008E+00	-1.008E+00	-1.008E+00	-1.008E+00	-1.008E+00			-6.993E-01	-6.993E-01	-6.993E-01	-6.993E-01	-6.993E-01	-6.994E-01		
**42**	-1.053E+00	-6.393E-01	-9.209E-01	-6.393E-01					-5.075E-01	-3.039E-01	-4.082E-01	-3.039E-01				
43	-6.516E-01	-1.188E+00	-1.211E+00	-8.271E-01	-7.003E-01				-2.881E-01	-5.680E-01	-5.694E-01	-3.693E-01	-3.085E-01			

44	-1.525E+00	-1.078E+00	-1.525E+00	-7.731E-01	-9.206E-01	-9.206E-01			-8.211E-01	-5.976E-01	-8.211E-01	-3.833E-01	-4.682E-01	-4.682E-01		
45	-6.910E-01	-1.223E+00	-7.978E-01	-6.910E-01	-1.223E+00				-3.038E-01	-5.838E-01	-3.513E-01	-3.038E-01	-5.838E-01			
46	-1.004E+00	-6.335E-01	-6.335E-01	-1.004E+00					-4.460E-01	-2.934E-01	-2.934E-01	-4.460E-01				
47	-7.488E-01	-7.488E-01	-7.487E-01	-7.488E-01	-7.487E-01	-7.488E-01			-3.246E-01	-3.246E-01	-3.246E-01	-3.246E-01	-3.246E-01	-3.246E-01		
48	-5.517E-01	-1.266E+00	-1.266E+00	-9.055E-01	-9.054E-01				-2.277E-01	-6.035E-01	-6.035E-01	-4.039E-01	-4.038E-01			
49	-1.188E+00	-7.488E-01	-6.491E-01	-1.188E+00	-7.024E-01	-7.023E-01			-5.719E-01	-3.246E-01	-2.786E-01	-5.718E-01	-3.038E-01	-3.038E-01		
50	-1.209E+00	-5.951E-01	-1.006E+00	-5.951E-01					-5.827E-01	-2.769E-01	-4.439E-01	-2.769E-01				
51	-1.283E+00	-1.283E+00	-7.041E-01	-7.041E-01	-7.041E-01	-7.041E-01			-6.187E-01	-6.187E-01	-3.006E-01	-3.006E-01	-3.006E-01	-3.006E-01		
52	-8.255E-01	-1.258E+00	-1.258E+00	-6.866E-01	-6.080E-01	-6.866E-01			-3.531E-01	-5.977E-01	-5.977E-01	-2.954E-01	-2.646E-01	-2.954E-01		
53	-3.866E-01	-3.863E-01	-1.225E+00						-3.134E-01	-3.133E-01	-7.156E-01					
54	-4.929E-01	-4.928E-01	-4.929E-01						-3.064E-01	-3.064E-01	-3.064E-01					
55	-6.100E-01	-6.141E-01	-6.912E-01	-1.272E+00	-6.100E-01	-6.910E-01			-2.662E-01	-2.692E-01	-2.975E-01	-6.136E-01	-2.662E-01	-2.974E-01		
56	-6.970E-01	-1.805E+00	-5.653E-01	-6.970E-01	-5.653E-01	-1.251E+00			-3.236E-01	-9.939E-01	-2.536E-01	-3.236E-01	-2.536E-01	-6.100E-01		
57	-1.780E+00	-7.667E-01	-1.180E+00	-6.829E-01	-6.536E-01	-4.597E-01			-9.955E-01	-3.586E-01	-5.706E-01	-3.207E-01	-2.828E-01	-2.137E-01		
58	-5.609E-01	-5.609E-01	-6.716E-01	-6.716E-01	-1.284E+00				-2.498E-01	-2.498E-01	-2.941E-01	-2.941E-01	-6.199E-01			
59	-6.308E-01	-6.308E-01	-4.933E-01	-1.256E+00					-2.874E-01	-2.874E-01	-2.297E-01	-6.076E-01				
60	-1.114E+00	-6.247E-01	-6.247E-01	-4.769E-01					-4.986E-01	-2.831E-01	-2.831E-01	-2.223E-01				
61	-5.143E-01	-5.718E-01	-5.061E-01	-5.940E-01	-1.304E+00	-1.314E+00			-2.316E-01	-2.644E-01	-2.331E-01	-2.731E-01	-6.471E-01	-6.467E-01		
62	-4.627E-01	-4.627E-01	-1.269E+00	-1.269E+00					-2.216E-01	-2.216E-01	-6.210E-01	-6.210E-01				
63	-6.084E-01	-6.084E-01	-6.084E-01	-6.084E-01	-6.084E-01	-6.084E-01			-2.667E-01	-2.667E-01	-2.667E-01	-2.667E-01	-2.667E-01	-2.667E-01		
64	-5.994E-01	-6.114E-01	-5.955E-01	-5.994E-01	-5.909E-01	-5.955E-01	-5.909E-01		-2.634E-01	-2.675E-01	-2.617E-01	-2.634E-01	-2.596E-01	-2.617E-01	-2.596E-01	
65	-1.784E+00	-1.784E+00	-5.842E-01	-5.843E-01	-5.842E-01	-5.842E-01			-9.918E-01	-9.918E-01	-2.702E-01	-2.702E-01	-2.702E-01	-2.702E-01		
66	-5.935E-01	-5.935E-01	-5.935E-01	-5.935E-01	-5.935E-01	-5.935E-01			-2.627E-01	-2.627E-01	-2.627E-01	-2.627E-01	-2.627E-01	-2.627E-01		
67	-5.893E-01	-5.733E-01	-5.952E-01	-5.951E-01	-5.894E-01	-5.949E-01	-5.794E-01	-5.951E-01	-2.593E-01	-2.540E-01	-2.540E-01	-2.618E-01	-2.593E-01	-2.617E-01	-2.550E-01	-2.618E-01
68	-5.758E-01	-5.758E-01	-5.758E-01	-6.138E-01	-5.758E-01	-6.138E-01			-2.540E-01	-2.540E-01	-2.540E-01	-2.694E-01	-2.540E-01	-2.694E-01		
69	-1.788E+00	-6.867E-01	-6.867E-01	-4.847E-01	-4.847E-01	-5.820E-01			-9.878E-01	-3.213E-01	-3.214E-01	-2.238E-01	-2.238E-01	-2.562E-01		
70	-5.870E-01	-6.027E-01	-5.925E-01	-5.452E-01	-5.520E-01				-2.613E-01	-2.678E-01	-2.635E-01	-2.445E-01	-2.472E-01			
71	-5.482E-01	-5.482E-01	-5.482E-01	-5.482E-01					-2.497E-01	-2.497E-01	-2.497E-01	-2.497E-01				
72	-5.443E-01	-5.443E-01	-5.443E-01	-5.443E-01					-2.495E-01	-2.495E-01	-2.495E-01	-2.495E-01				
73	-4.737E-01	-4.737E-01	-5.613E-01	-5.616E-01	-1.902E+00				-2.132E-01	-2.132E-01	-2.779E-01	-2.780E-01	-9.974E-01			

	G								K							
#	BCP1	BCP2	BCP3	BCP4	BCP5	BCP6	BCP7	BCP8	BCP1	BCP2	BCP3	BCP4	BCP5	BCP6	BCP7	BCP8
1	1.057E-01	1.057E-01	1.057E-01	1.057E-01	1.057E-01	1.057E-01			3.731E-01	3.731E-01	3.731E-01	3.731E-01	3.731E-01	3.731E-01		
2	1.059E-01	1.059E-01	1.059E-01	1.059E-01	1.059E-01	1.059E-01			3.735E-01	3.735E-01	3.737E-01	3.735E-01	3.737E-01	3.735E-01		
3	1.055E-01	1.055E-01	1.055E-01	1.056E-01	1.055E-01	1.056E-01			3.722E-01	3.722E-01	3.723E-01	3.727E-01	3.722E-01	3.727E-01		
4	1.101E-01	1.105E-01	1.031E-01	1.040E-01	1.031E-01	1.105E-01			3.886E-01	3.840E-01	3.592E-01	3.628E-01	3.592E-01	3.840E-01		
5	9.409E-02	9.409E-02	9.409E-02	9.409E-02	9.409E-02	9.409E-02	9.409E-02	9.409E-02	3.604E-01	3.604E-01	3.604E-01	3.604E-01	3.604E-01	3.604E-01	3.604E-01	3.604E-01
6	9.826E-02	9.645E-02	1.132E-01	1.018E-01	1.132E-01	9.826E-02			3.485E-01	3.414E-01	3.944E-01	3.675E-01	3.944E-01	3.485E-01		
7	9.329E-02	1.145E-01	9.639E-02	9.914E-02	9.731E-02	1.136E-01			3.336E-01	3.979E-01	3.485E-01	3.577E-01	3.472E-01	3.936E-01		
8	9.793E-02	9.812E-02	9.756E-02	9.773E-02	9.794E-02	9.811E-02	9.772E-02		3.381E-01	3.388E-01	3.368E-01	3.374E-01	3.382E-01	3.387E-01	3.374E-01	
9	1.026E-01	1.026E-01	1.026E-01	1.026E-01	1.026E-01				3.343E-01	3.343E-01	3.343E-01	3.343E-01	3.343E-01			
10	1.001E-01	8.707E-02	1.001E-01	1.134E-01	8.707E-02	9.199E-02	1.134E-01		3.740E-01	3.392E-01	3.740E-01	4.084E-01	3.392E-01	3.413E-01	4.084E-01	
11	9.930E-02	9.930E-02	9.930E-02	9.930E-02	9.930E-02	9.930E-02	9.930E-02	9.930E-02	3.315E-01	3.315E-01	3.315E-01	3.315E-01	3.315E-01	3.315E-01	3.315E-01	3.315E-01
12	1.112E-01	9.752E-02	9.471E-02	1.200E-01	9.471E-02	1.200E-01			3.818E-01	3.485E-01	3.356E-01	4.071E-01	3.356E-01	4.071E-01		
13	1.175E-01	8.962E-02	9.292E-02	9.470E-02	9.292E-02	1.175E-01			4.039E-01	3.251E-01	3.397E-01	3.437E-01	3.397E-01	4.039E-01		
14	8.982E-02	8.913E-02	1.170E-01	9.411E-02	9.972E-02	1.106E-01			3.249E-01	3.211E-01	4.064E-01	3.446E-01	3.586E-01	3.808E-01		
15	1.172E-01	8.957E-02	9.233E-02	9.472E-02	9.233E-02	1.172E-01			4.025E-01	3.242E-01	3.364E-01	3.439E-01	3.364E-01	4.025E-01		
16	9.104E-02	9.141E-02	9.469E-02	1.200E-01	9.468E-02	9.141E-02			3.327E-01	3.355E-01	3.416E-01	4.099E-01	3.416E-01	3.355E-01		
17	8.437E-02	1.037E-01	1.037E-01	1.038E-01	8.432E-02	1.038E-01			3.102E-01	3.687E-01	3.687E-01	3.692E-01	3.100E-01	3.692E-01		
18	9.104E-02	9.104E-02	9.104E-02	9.104E-02	9.104E-02	9.104E-02			3.327E-01	3.327E-01	3.327E-01	3.327E-01	3.327E-01	3.327E-01		
19	9.906E-02	9.912E-02	9.912E-02	9.906E-02					3.383E-01	3.386E-01	3.386E-01	3.383E-01				
20	8.289E-02	8.810E-02	9.700E-02	1.152E-01	9.700E-02	8.289E-02			3.245E-01	3.290E-01	3.639E-01	4.140E-01	3.639E-01	3.245E-01		
21	9.840E-02	1.020E-01	9.793E-02	9.316E-02	9.622E-02				3.251E-01	3.349E-01	3.259E-01	3.104E-01	3.188E-01			
22	9.833E-02	1.112E-01	9.833E-02	9.016E-02	1.112E-01	7.930E-02	9.016E-02		3.464E-01	3.820E-01	3.464E-01	3.312E-01	3.820E-01	2.785E-01	3.312E-01	
23	8.900E-02	1.233E-01	8.988E-02	1.233E-01	1.129E-01	8.988E-02			3.192E-01	4.064E-01	3.159E-01	4.064E-01	3.791E-01	3.159E-01		
24	9.621E-02	1.286E-01	8.758E-02	1.286E-01	1.183E-01	8.758E-02			3.477E-01	4.235E-01	3.139E-01	4.235E-01	3.978E-01	3.139E-01		
25	8.857E-02	8.437E-02	1.219E-01	9.012E-02	1.219E-01	8.857E-02			3.278E-01	3.102E-01	4.144E-01	3.309E-01	4.144E-01	3.278E-01		
26	7.930E-02	1.042E-01	1.027E-01	1.042E-01	1.027E-01				2.785E-01	3.498E-01	3.455E-01	3.498E-01	3.455E-01			
27	1.137E-01	1.137E-01	7.016E-02	1.137E-01	1.137E-01	7.014E-02			3.776E-01	3.776E-01	2.994E-01	3.776E-01	3.776E-01	2.993E-01		
28	1.039E-01	1.036E-01	1.039E-01	1.055E-01	1.036E-01	6.574E-02	1.055E-01		3.699E-01	3.705E-01	3.699E-01	3.822E-01	3.705E-01	2.467E-01	3.822E-01	
29	9.329E-02	7.866E-02	8.581E-02	1.278E-01	8.581E-02	9.329E-02			3.336E-01	2.924E-01	3.212E-01	4.317E-01	3.212E-01	3.336E-01		
30	6.574E-02	1.050E-01	1.048E-01	1.050E-01	1.049E-01				2.467E-01	3.592E-01	3.588E-01	3.592E-01	3.589E-01			
31	6.878E-02	1.115E-01	1.028E-01	1.115E-01	7.559E-02	1.028E-01			2.677E-01	3.948E-01	3.757E-01	3.948E-01	2.988E-01	3.757E-01		
32	1.166E-01	1.255E-01	1.031E-01	1.166E-01					3.182E-01	3.476E-01	2.428E-01	3.182E-01				
33	7.473E-02	9.645E-02	9.644E-02	7.473E-02	7.473E-02	9.644E-02			2.820E-01	3.414E-01	3.413E-01	2.820E-01	2.820E-01	3.413E-01		
34	1.098E-01	8.117E-02	1.097E-01	1.348E-01	8.125E-02	6.583E-02	1.347E-01		3.633E-01	2.884E-01	3.631E-01	4.351E-01	2.886E-01	2.496E-01	4.349E-01	
35	8.982E-02	7.088E-02	8.982E-02	8.982E-02	7.088E-02	8.982E-02			3.249E-01	2.701E-01	3.249E-01	3.249E-01	2.701E-01	3.249E-01		
36	8.542E-02	9.311E-02	6.878E-02	9.311E-02					3.204E-01	3.410E-01	2.677E-01	3.410E-01				
37	2.226E-01	1.187E-01	1.030E-01	1.186E-01	1.030E-01	1.020E-01			6.648E-01	3.609E-01	3.436E-01	3.605E-01	3.437E-01	3.565E-01		
38	9.199E-02	6.479E-02	1.126E-01	6.479E-02	1.126E-01				3.413E-01	2.598E-01	3.940E-01	2.598E-01	3.940E-01			
39	6.935E-02	1.424E-01	6.935E-02	6.935E-02	1.424E-01	1.424E-01	1.424E-01	6.935E-02	2.737E-01	4.563E-01	2.737E-01	2.737E-01	4.563E-01	4.563E-01	4.563E-01	2.737E-01
40	6.760E-02	1.359E-01	8.313E-02	7.305E-02	1.348E-01				2.450E-01	4.341E-01	2.969E-01	2.679E-01	4.335E-01			
41	2.237E-01	2.237E-01	2.237E-01	2.237E-01	2.237E-01	2.237E-01			4.756E-01	4.756E-01	4.756E-01	4.756E-01	4.756E-01	4.757E-01		
**42**	1.221E-01	7.203E-02	8.900E-02	7.203E-02					3.854E-01	2.319E-01	3.192E-01	2.319E-01				
43	6.261E-02	1.355E-01	1.333E-01	8.127E-02	6.672E-02				2.255E-01	4.325E-01	4.361E-01	2.880E-01	2.418E-01			
44	2.199E-01	1.640E-01	2.199E-01	9.504E-02	1.190E-01	1.190E-01			6.012E-01	4.336E-01	6.012E-01	2.883E-01	3.492E-01	3.492E-01		
45	6.550E-02	1.391E-01	7.594E-02	6.550E-02	1.391E-01				2.383E-01	4.447E-01	2.754E-01	2.383E-01	4.447E-01			
46	9.752E-02	6.750E-02	6.750E-02	9.752E-02					3.485E-01	2.259E-01	2.259E-01	3.485E-01				
47	6.870E-02	6.870E-02	6.870E-02	6.870E-02	6.870E-02	6.870E-02			2.559E-01	2.559E-01	2.559E-01	2.559E-01	2.559E-01	2.559E-01		
48	4.488E-02	1.435E-01	1.436E-01	8.874E-02	8.872E-02				1.828E-01	4.599E-01	4.600E-01	3.151E-01	3.151E-01			
49	1.374E-01	6.870E-02	5.816E-02	1.374E-01	6.412E-02	6.412E-02			4.345E-01	2.559E-01	2.204E-01	4.344E-01	2.397E-01	2.397E-01		
50	1.403E-01	6.407E-02	9.621E-02	6.407E-02					4.424E-01	2.129E-01	3.477E-01	2.129E-01				
51	1.490E-01	1.490E-01	6.228E-02	6.228E-02	6.228E-02	6.228E-02			4.697E-01	4.697E-01	2.383E-01	2.383E-01	2.383E-01	2.383E-01		
52	7.338E-02	1.416E-01	1.416E-01	6.187E-02	5.629E-02	6.187E-02			2.797E-01	4.561E-01	4.561E-01	2.335E-01	2.083E-01	2.335E-01		
53	1.084E-01	1.084E-01	2.047E-01						2.050E-01	2.049E-01	5.109E-01					
54	9.161E-02	9.160E-02	9.161E-02						2.148E-01	2.148E-01	2.148E-01					
55	5.686E-02	5.782E-02	6.234E-02	1.478E-01	5.685E-02	6.233E-02			2.094E-01	2.113E-01	2.351E-01	4.658E-01	2.093E-01	2.351E-01		
56	7.469E-02	2.713E-01	5.614E-02	7.469E-02	5.614E-02	1.487E-01			2.489E-01	7.226E-01	1.975E-01	2.489E-01	1.975E-01	4.614E-01		
57	2.753E-01	8.346E-02	1.378E-01	7.501E-02	5.972E-02	4.941E-02			7.202E-01	2.751E-01	4.328E-01	2.457E-01	2.231E-01	1.643E-01		
58	5.477E-02	5.477E-02	6.311E-02	6.311E-02	1.494E-01				1.950E-01	1.950E-01	2.310E-01	2.310E-01	4.705E-01			
59	6.488E-02	6.488E-02	5.318E-02	1.468E-01					2.226E-01	2.226E-01	1.765E-01	4.608E-01				
60	1.101E-01	6.346E-02	6.346E-02	5.155E-02					3.886E-01	2.196E-01	2.196E-01	1.708E-01				
61	5.153E-02	6.074E-02	5.328E-02	6.230E-02	1.606E-01	1.590E-01			1.801E-01	2.037E-01	1.798E-01	2.108E-01	4.865E-01	4.877E-01		
62	5.297E-02	5.297E-02	1.519E-01	1.519E-01					1.687E-01	1.687E-01	4.692E-01	4.692E-01				
63	5.731E-02	5.731E-02	5.731E-02	5.731E-02	5.731E-02	5.731E-02			2.094E-01	2.094E-01	2.094E-01	2.094E-01	2.094E-01	2.094E-01		
64	5.678E-02	5.730E-02	5.644E-02	5.678E-02	5.595E-02	5.644E-02	5.595E-02		2.066E-01	2.102E-01	2.053E-01	2.066E-01	2.037E-01	2.053E-01	2.037E-01	
65	2.729E-01	2.729E-01	6.207E-02	6.208E-02	6.207E-02	6.208E-02			7.189E-01	7.189E-01	2.081E-01	2.081E-01	2.081E-01	2.081E-01		
66	5.715E-02	5.715E-02	5.715E-02	5.715E-02	5.715E-02	5.715E-02			2.055E-01	2.055E-01	2.055E-01	2.055E-01	2.055E-01	2.055E-01		
67	5.600E-02	5.534E-02	5.651E-02	5.651E-02	5.600E-02	5.650E-02	5.508E-02	5.650E-02	2.033E-01	1.987E-01	2.053E-01	2.053E-01	2.033E-01	2.052E-01	1.999E-01	2.053E-01
68	5.502E-02	5.501E-02	5.502E-02	5.798E-02	5.501E-02	5.798E-02			1.990E-01	1.990E-01	1.990E-01	2.114E-01	1.990E-01	2.114E-01		
69	2.704E-01	7.484E-02	7.484E-02	5.130E-02	5.130E-02	5.537E-02			7.174E-01	2.465E-01	2.465E-01	1.725E-01	1.725E-01	2.009E-01		
70	5.726E-02	5.855E-02	5.769E-02	5.411E-02	5.461E-02				2.040E-01	2.092E-01	2.058E-01	1.904E-01	1.926E-01			
71	5.634E-02	5.634E-02	5.634E-02	5.634E-02					1.934E-01	1.934E-01	1.934E-01	1.934E-01				
72	5.669E-02	5.669E-02	5.669E-02	5.669E-02					1.928E-01	1.928E-01	1.928E-01	1.928E-01				
73	4.738E-02	4.738E-02	6.880E-02	6.881E-02	2.609E-01				1.658E-01	1.658E-01	2.091E-01	2.092E-01	7.365E-01			

	L								ESP							
#	BCP1	BCP2	BCP3	BCP4	BCP5	BCP6	BCP7	BCP8	BCP1	BCP2	BCP3	BCP4	BCP5	BCP6	BCP7	BCP8
1	2.675E-01	2.675E-01	2.675E-01	2.675E-01	2.675E-01	2.675E-01			8.620E-01	8.620E-01	8.620E-01	8.620E-01	8.620E-01	8.620E-01		
2	2.676E-01	2.676E-01	2.678E-01	2.676E-01	2.678E-01	2.676E-01			7.491E-01	7.491E-01	7.494E-01	7.491E-01	7.494E-01	7.491E-01		
3	2.667E-01	2.668E-01	2.668E-01	2.671E-01	2.667E-01	2.672E-01			6.758E-01	6.759E-01	6.759E-01	6.786E-01	6.758E-01	6.787E-01		
4	2.785E-01	2.736E-01	2.560E-01	2.588E-01	2.560E-01	2.736E-01			8.475E-01	8.790E-01	8.435E-01	8.457E-01	8.435E-01	8.790E-01		
5	2.663E-01	2.663E-01	2.663E-01	2.663E-01	2.663E-01	2.663E-01	2.663E-01	2.663E-01	1.261E+00	1.261E+00	1.261E+00	1.261E+00	1.261E+00	1.261E+00	1.261E+00	1.261E+00
6	2.503E-01	2.449E-01	2.812E-01	2.657E-01	2.812E-01	2.503E-01			8.363E-01	8.257E-01	8.906E-01	8.553E-01	8.906E-01	8.363E-01		
7	2.403E-01	2.834E-01	2.521E-01	2.585E-01	2.499E-01	2.800E-01			8.140E-01	8.951E-01	8.325E-01	8.437E-01	8.340E-01	8.903E-01		
8	2.402E-01	2.406E-01	2.392E-01	2.397E-01	2.402E-01	2.406E-01	2.397E-01		5.452E-01	5.452E-01	5.446E-01	5.447E-01	5.453E-01	5.452E-01	5.447E-01	
9	2.317E-01	2.317E-01	2.317E-01	2.317E-01	2.317E-01				5.927E-01	5.927E-01	5.927E-01	5.927E-01	5.927E-01			
10	2.739E-01	2.522E-01	2.739E-01	2.950E-01	2.522E-01	2.493E-01	2.950E-01		1.237E+00	1.197E+00	1.237E+00	1.299E+00	1.197E+00	1.209E+00	1.299E+00	
11	2.322E-01	2.322E-01	2.322E-01	2.322E-01	2.322E-01	2.322E-01	2.322E-01	2.322E-01	4.657E-01	4.657E-01	4.657E-01	4.657E-01	4.657E-01	4.657E-01	4.657E-01	4.657E-01
12	2.706E-01	2.510E-01	2.409E-01	2.871E-01	2.409E-01	2.871E-01			8.772E-01	8.103E-01	8.211E-01	9.251E-01	8.211E-01	9.251E-01		
13	2.863E-01	2.355E-01	2.467E-01	2.490E-01	2.467E-01	2.863E-01			9.028E-01	8.009E-01	8.207E-01	8.262E-01	8.206E-01	9.028E-01		
14	2.351E-01	2.320E-01	2.894E-01	2.505E-01	2.589E-01	2.703E-01			8.052E-01	7.997E-01	9.056E-01	8.271E-01	8.467E-01	8.784E-01		
15	2.852E-01	2.346E-01	2.441E-01	2.492E-01	2.441E-01	2.852E-01			7.323E-01	6.327E-01	6.493E-01	6.559E-01	6.493E-01	7.323E-01		
16	2.416E-01	2.441E-01	2.469E-01	2.899E-01	2.469E-01	2.441E-01			8.165E-01	8.182E-01	8.263E-01	9.138E-01	8.263E-01	8.182E-01		
17	2.258E-01	2.650E-01	2.650E-01	2.654E-01	2.257E-01	2.654E-01			7.830E-01	8.603E-01	8.603E-01	8.610E-01	7.827E-01	8.610E-01		
18	2.416E-01	2.416E-01	2.416E-01	2.416E-01	2.416E-01	2.416E-01			8.165E-01	8.165E-01	8.165E-01	8.165E-01	8.165E-01	8.165E-01		
19	2.392E-01	2.395E-01	2.395E-01	2.392E-01					1.403E+00	1.403E+00	1.403E+00	1.403E+00				
20	2.416E-01	2.409E-01	2.669E-01	2.988E-01	2.669E-01	2.416E-01			1.186E+00	1.185E+00	1.241E+00	1.281E+00	1.241E+00	1.186E+00		
21	2.267E-01	2.329E-01	2.280E-01	2.172E-01	2.226E-01				5.211E-01	5.340E-01	4.832E-01	5.048E-01	5.165E-01			
22	2.481E-01	2.708E-01	2.481E-01	2.411E-01	2.708E-01	1.992E-01	2.411E-01		6.081E-01	6.559E-01	6.081E-01	5.850E-01	6.559E-01	5.012E-01	5.850E-01	

23	2.302E-01	2.831E-01	2.260E-01	2.831E-01	2.662E-01	2.260E-01			5.509E-01	7.039E-01	5.742E-01	7.039E-01	6.517E-01	5.742E-01		
24	2.515E-01	2.949E-01	2.263E-01	2.949E-01	2.795E-01	2.263E-01			8.071E-01	9.456E-01	7.869E-01	9.456E-01	8.911E-01	7.869E-01		
25	2.392E-01	2.258E-01	2.925E-01	2.408E-01	2.925E-01	2.392E-01			8.062E-01	7.830E-01	9.166E-01	8.104E-01	9.166E-01	8.062E-01		
26	1.992E-01	2.457E-01	2.428E-01	2.457E-01	2.428E-01				5.012E-01	5.941E-01	5.923E-01	5.941E-01	5.923E-01			
27	2.640E-01	2.640E-01	2.293E-01	2.640E-01	2.640E-01	2.292E-01			1.385E+00	1.385E+00	1.243E+00	1.385E+00	1.385E+00	1.243E+00		
28	2.660E-01	2.669E-01	2.660E-01	2.767E-01	2.669E-01	1.809E-01	2.767E-01		8.729E-01	8.745E-01	8.730E-01	8.867E-01	8.745E-01	6.904E-01	8.867E-01	
29	2.403E-01	2.137E-01	2.354E-01	3.039E-01	2.354E-01	2.403E-01			8.140E-01	7.628E-01	7.969E-01	9.383E-01	7.969E-01	8.140E-01		
30	1.809E-01	2.542E-01	2.540E-01	2.542E-01	2.540E-01				6.904E-01	8.392E-01	8.366E-01	8.392E-01	8.366E-01			
31	1.989E-01	2.833E-01	2.729E-01	2.833E-01	2.232E-01	2.729E-01			1.136E+00	1.355E+00	1.278E+00	1.355E+00	1.178E+00	1.278E+00		
32	2.015E-01	2.220E-01	1.397E-01	2.015E-01					9.128E-01	9.588E-01	8.052E-01	9.128E-01				
33	2.073E-01	2.449E-01	2.449E-01	2.073E-01	2.073E-01	2.449E-01			7.485E-01	8.257E-01	8.257E-01	7.485E-01	7.485E-01	8.257E-01		
34	2.535E-01	2.072E-01	2.533E-01	3.003E-01	2.074E-01	1.837E-01	3.002E-01		6.733E-01	5.717E-01	6.730E-01	7.641E-01	5.720E-01	5.139E-01	7.639E-01	
35	2.351E-01	1.992E-01	2.351E-01	2.351E-01	1.992E-01	2.351E-01			8.052E-01	7.334E-01	8.052E-01	8.052E-01	7.334E-01	8.052E-01		
36	2.349E-01	2.479E-01	1.989E-01	2.479E-01					1.229E+00	1.264E+00	1.136E+00	1.264E+00				
37	4.422E-01	2.422E-01	2.406E-01	2.419E-01	2.407E-01	2.544E-01			1.205E+00	9.533E-01	8.553E-01	9.528E-01	8.554E-01	8.570E-01		
38	2.493E-01	1.950E-01	2.814E-01	1.950E-01	2.814E-01				1.209E+00	1.099E+00	1.291E+00	1.099E+00	1.291E+00			
39	2.043E-01	3.139E-01	2.043E-01	2.043E-01	3.139E-01	3.139E-01	3.139E-01	2.043E-01	7.317E-01	9.752E-01	7.317E-01	7.317E-01	9.752E-01	9.752E-01	9.752E-01	7.317E-01
40	1.774E-01	2.982E-01	2.137E-01	1.949E-01	2.987E-01				6.935E-01	9.402E-01	7.352E-01	7.306E-01	9.529E-01			
41	2.519E-01	2.519E-01	2.519E-01	2.519E-01	2.519E-01	2.519E-01			1.307E+00	1.307E+00	1.307E+00	1.307E+00	1.307E+00	1.307E+00		
**42**	2.633E-01	1.598E-01	2.302E-01	1.598E-01					6.650E-01	4.689E-01	5.509E-01	4.689E-01				
43	1.629E-01	2.971E-01	3.028E-01	2.068E-01	1.751E-01				6.487E-01	9.312E-01	9.056E-01	7.503E-01	6.798E-01			
44	3.812E-01	2.695E-01	3.812E-01	1.933E-01	2.302E-01	2.302E-01			1.252E+00	1.117E+00	1.252E+00	8.161E-01	9.424E-01	9.424E-01		
45	1.727E-01	3.056E-01	1.995E-01	1.727E-01	3.056E-01				6.687E-01	9.457E-01	7.254E-01	6.687E-01	9.457E-01			
46	2.510E-01	1.584E-01	1.584E-01	2.510E-01					8.103E-01	6.854E-01	6.854E-01	8.103E-01				
47	1.872E-01	1.872E-01	1.872E-01	1.872E-01	1.872E-01	1.872E-01			6.871E-01	6.871E-01	6.871E-01	6.871E-01	6.871E-01	6.871E-01		
48	1.379E-01	3.164E-01	3.164E-01	2.264E-01	2.263E-01				8.362E-01	1.231E+00	1.231E+00	1.066E+00	1.066E+00			
49	2.971E-01	1.872E-01	1.623E-01	2.971E-01	1.756E-01	1.756E-01			9.249E-01	6.871E-01	6.275E-01	9.249E-01	6.587E-01	6.587E-01		
50	3.021E-01	1.488E-01	2.515E-01	1.488E-01					9.614E-01	6.615E-01	8.071E-01	6.615E-01				
51	3.206E-01	3.206E-01	1.760E-01	1.760E-01	1.760E-01	1.760E-01			9.780E-01	9.780E-01	6.677E-01	6.677E-01	6.677E-01	6.677E-01		
52	2.064E-01	3.146E-01	3.146E-01	1.717E-01	1.520E-01	1.717E-01			7.318E-01	9.601E-01	9.601E-01	6.600E-01	6.151E-01	6.600E-01		
53	9.665E-02	9.658E-02	3.061E-01						7.369E-01	7.368E-01	1.108E+00					
54	1.232E-01	1.232E-01	1.232E-01						7.067E-01	7.067E-01	7.067E-01					
55	1.525E-01	1.535E-01	1.728E-01	3.180E-01	1.525E-01	1.727E-01			6.092E-01	6.106E-01	6.565E-01	9.681E-01	6.092E-01	6.564E-01		
56	1.743E-01	4.513E-01	1.413E-01	1.743E-01	1.413E-01	3.127E-01			7.715E-01	1.264E+00	6.340E-01	7.715E-01	6.340E-01	9.889E-01		
57	4.449E-01	1.917E-01	2.949E-01	1.707E-01	1.634E-01	1.149E-01			1.303E+00	8.118E-01	9.595E-01	7.742E-01	6.615E-01	5.760E-01		
58	1.402E-01	1.402E-01	1.679E-01	1.679E-01	3.210E-01				5.883E-01	5.883E-01	6.514E-01	6.514E-01	9.721E-01			
59	1.577E-01	1.577E-01	1.233E-01	3.140E-01					6.435E-01	6.435E-01	5.669E-01	9.611E-01				
60	2.785E-01	1.562E-01	1.562E-01	1.192E-01					8.475E-01	6.474E-01	6.474E-01	5.598E-01				
61	1.286E-01	1.429E-01	1.265E-01	1.485E-01	3.259E-01	3.286E-01			5.751E-01	6.407E-01	5.806E-01	6.512E-01	1.039E+00	1.036E+00		
62	1.157E-01	1.157E-01	3.173E-01	3.173E-01					5.792E-01	5.792E-01	9.863E-01	9.863E-01				
63	1.521E-01	1.521E-01	1.521E-01	1.521E-01	1.521E-01	1.521E-01			6.027E-01	6.028E-01	6.028E-01	6.027E-01	6.027E-01	6.027E-01		
64	1.499E-01	1.529E-01	1.489E-01	1.499E-01	1.477E-01	1.489E-01	1.477E-01		5.985E-01	6.034E-01	5.966E-01	5.985E-01	5.939E-01	5.966E-01	5.939E-01	
65	4.461E-01	4.461E-01	1.460E-01	1.461E-01	1.461E-01	1.460E-01			1.273E+00	1.273E+00	7.198E-01	7.198E-01	7.198E-01	7.198E-01		
66	1.484E-01	1.484E-01	1.484E-01	1.484E-01	1.484E-01	1.484E-01			5.934E-01	5.934E-01	5.934E-01	5.934E-01	5.934E-01	5.934E-01		
67	1.473E-01	1.433E-01	1.488E-01	1.488E-01	1.473E-01	1.487E-01	1.449E-01	1.488E-01	5.937E-01	5.874E-01	5.963E-01	5.962E-01	5.938E-01	5.961E-01	5.873E-01	5.962E-01
68	1.440E-01	1.440E-01	1.440E-01	1.535E-01	1.440E-01	1.535E-01			5.866E-01	5.866E-01	5.866E-01	6.067E-01	5.866E-01	6.067E-01		
69	4.470E-01	1.717E-01	1.717E-01	1.212E-01	1.212E-01	1.455E-01			1.256E+00	7.628E-01	7.628E-01	5.763E-01	5.763E-01	6.084E-01		
70	1.467E-01	1.507E-01	1.481E-01	1.363E-01	1.380E-01				5.963E-01	6.047E-01	5.992E-01	5.747E-01	5.782E-01			
71	1.371E-01	1.371E-01	1.371E-01	1.371E-01					5.835E-01	5.835E-01	5.835E-01	5.835E-01				
72	1.361E-01	1.361E-01	1.361E-01	1.361E-01					3.654E-01	3.654E-01	3.654E-01	3.654E-01				
73	1.184E-01	1.184E-01	1.403E-01	1.404E-01	4.756E-01				5.585E-01	5.585E-01	7.237E-01	7.238E-01	1.241E+00			

	ESPe								ESPn							
**#**	**BCP1**	**BCP2**	**BCP3**	**BCP4**	**BCP5**	**BCP6**	**BCP7**	**BCP8**	**BCP1**	**BCP2**	**BCP3**	**BCP4**	**BCP5**	**BCP6**	**BCP7**	**BCP8**
1	-1.561E+01	-1.561E+01	-1.561E+01	-1.561E+01	-1.561E+01	-1.561E+01			1.647E+01	1.647E+01	1.647E+01	1.647E+01	1.647E+01	1.647E+01		
2	-1.572E+01	-1.572E+01	-1.572E+01	-1.572E+01	-1.572E+01	-1.572E+01			1.647E+01	1.647E+01	1.647E+01	1.647E+01	1.647E+01	1.647E+01		
3	-1.579E+01	-1.579E+01	-1.579E+01	-1.578E+01	-1.579E+01	-1.578E+01			1.647E+01	1.647E+01	1.647E+01	1.646E+01	1.647E+01	1.646E+01		
4	-1.955E+01	-1.839E+01	-1.754E+01	-1.737E+01	-1.754E+01	-1.839E+01			2.040E+01	1.927E+01	1.839E+01	1.822E+01	1.839E+01	1.927E+01		
5	-1.666E+01	-1.666E+01	-1.666E+01	-1.666E+01	-1.666E+01	-1.666E+01	-1.666E+01	-1.666E+01	1.792E+01	1.792E+01	1.792E+01	1.792E+01	1.792E+01	1.792E+01	1.792E+01	1.792E+01
6	-2.630E+01	-2.789E+01	-2.405E+01	-2.318E+01	-2.405E+01	-2.630E+01			2.714E+01	2.872E+01	2.494E+01	2.403E+01	2.494E+01	2.714E+01		
7	-2.460E+01	-2.127E+01	-2.281E+01	-2.085E+01	-2.366E+01	-2.177E+01			2.541E+01	2.217E+01	2.364E+01	2.170E+01	2.449E+01	2.266E+01		
8	-1.654E+01	-1.654E+01	-1.654E+01	-1.654E+01	-1.654E+01	-1.654E+01	-1.654E+01		1.708E+01	1.708E+01	1.708E+01	1.708E+01	1.708E+01	1.708E+01	1.708E+01	
9	-1.463E+01	-1.463E+01	-1.463E+01	-1.463E+01	-1.463E+01				1.522E+01	1.522E+01	1.522E+01	1.522E+01	1.522E+01			
10	-1.816E+01	-1.847E+01	-1.816E+01	-1.974E+01	-1.847E+01	-2.067E+01	-1.974E+01		1.940E+01	1.967E+01	1.940E+01	2.104E+01	1.967E+01	2.188E+01	2.104E+01	
11	-1.731E+01	-1.731E+01	-1.731E+01	-1.731E+01	-1.731E+01	-1.731E+01	-1.731E+01	-1.731E+01	1.778E+01	1.778E+01	1.778E+01	1.778E+01	1.778E+01	1.778E+01	1.778E+01	1.778E+01
12	-1.952E+01	-2.285E+01	-1.977E+01	-2.130E+01	-1.977E+01	-2.130E+01			2.040E+01	2.366E+01	2.059E+01	2.222E+01	2.059E+01	2.222E+01		
13	-1.904E+01	-2.127E+01	-2.016E+01	-1.854E+01	-2.016E+01	-1.904E+01			1.995E+01	2.207E+01	2.098E+01	1.937E+01	2.098E+01	1.995E+01		
14	-2.952E+01	-2.863E+01	-2.484E+01	-2.618E+01	-2.531E+01	-2.779E+01			3.032E+01	2.943E+01	2.575E+01	2.701E+01	2.616E+01	2.866E+01		
15	-1.920E+01	-2.142E+01	-2.030E+01	-1.870E+01	-2.030E+01	-1.920E+01			1.993E+01	2.205E+01	2.095E+01	1.936E+01	2.095E+01	1.993E+01		
16	-3.287E+01	-2.874E+01	-3.160E+01	-2.757E+01	-3.160E+01	-2.874E+01			3.368E+01	2.956E+01	3.243E+01	2.848E+01	3.243E+01	2.956E+01		
17	-2.422E+01	-2.361E+01	-2.361E+01	-2.361E+01	-2.422E+01	-2.361E+01			2.501E+01	2.447E+01	2.447E+01	2.447E+01	2.500E+01	2.447E+01		
18	-3.287E+01	-3.287E+01	-3.287E+01	-3.287E+01	-3.287E+01	-3.287E+01			3.368E+01	3.368E+01	3.368E+01	3.368E+01	3.368E+01	3.368E+01		
19	-1.257E+01	-1.257E+01	-1.257E+01	-1.257E+01					1.397E+01	1.397E+01	1.397E+01	1.397E+01				
20	-1.833E+01	-2.085E+01	-1.980E+01	-1.823E+01	-1.980E+01	-1.833E+01			1.952E+01	2.204E+01	2.104E+01	1.952E+01	2.104E+01	1.952E+01		
21	-1.730E+01	-1.733E+01	-1.955E+01	-1.819E+01	-1.822E+01				1.782E+01	1.786E+01	2.003E+01	1.870E+01	1.874E+01			
22	-1.870E+01	-1.919E+01	-1.870E+01	-2.016E+01	-1.919E+01	-2.114E+01	-2.016E+01		1.931E+01	1.984E+01	1.931E+01	2.075E+01	1.984E+01	2.165E+01	2.075E+01	
23	-1.919E+01	-1.826E+01	-1.734E+01	-1.826E+01	-1.735E+01	-1.734E+01			1.974E+01	1.896E+01	1.792E+01	1.896E+01	1.800E+01	1.792E+01		
24	-1.907E+01	-1.810E+01	-1.715E+01	-1.810E+01	-1.721E+01	-1.715E+01			1.988E+01	1.905E+01	1.793E+01	1.905E+01	1.810E+01	1.793E+01		
25	-2.272E+01	-2.422E+01	-2.120E+01	-2.048E+01	-2.120E+01	-2.272E+01			2.353E+01	2.501E+01	2.211E+01	2.129E+01	2.211E+01	2.353E+01		
26	-2.114E+01	-2.016E+01	-1.887E+01	-2.016E+01	-1.887E+01				2.165E+01	2.076E+01	1.946E+01	2.076E+01	1.946E+01			
27	-1.508E+01	-1.508E+01	-1.492E+01	-1.508E+01	-1.508E+01	-1.492E+01			1.647E+01	1.647E+01	1.617E+01	1.647E+01	1.647E+01	1.617E+01		
28	-1.858E+01	-1.897E+01	-1.858E+01	-2.011E+01	-1.897E+01	-2.083E+01	-2.011E+01		1.945E+01	1.984E+01	1.945E+01	2.100E+01	1.984E+01	2.153E+01	2.100E+01	
29	-2.460E+01	-2.455E+01	-2.311E+01	-2.246E+01	-2.311E+01	-2.460E+01			2.541E+01	2.532E+01	2.391E+01	2.340E+01	2.391E+01	2.541E+01		
30	-2.083E+01	-1.996E+01	-1.872E+01	-1.996E+01	-1.872E+01				2.153E+01	2.080E+01	1.956E+01	2.080E+01	1.956E+01			
31	-1.840E+01	-1.749E+01	-1.683E+01	-1.749E+01	-1.650E+01	-1.683E+01			1.954E+01	1.885E+01	1.811E+01	1.885E+01	1.768E+01	1.811E+01		
32	-1.262E+01	-1.283E+01	-1.236E+01	-1.262E+01					1.353E+01	1.379E+01	1.316E+01	1.353E+01				
33	-2.749E+01	-2.789E+01	-2.789E+01	-2.749E+01	-2.749E+01	-2.789E+01			2.824E+01	2.872E+01	2.872E+01	2.824E+01	2.824E+01	2.872E+01		
34	-1.660E+01	-1.638E+01	-1.660E+01	-1.675E+01	-1.638E+01	-1.624E+01	-1.675E+01		1.728E+01	1.695E+01	1.728E+01	1.752E+01	1.695E+01	1.675E+01	1.752E+01	
35	-2.952E+01	-2.890E+01	-2.952E+01	-2.952E+01	-2.890E+01	-2.952E+01			3.032E+01	2.963E+01	3.032E+01	3.032E+01	2.963E+01	3.032E+01		
36	-1.636E+01	-1.723E+01	-1.840E+01	-1.723E+01					1.759E+01	1.849E+01	1.954E+01	1.849E+01				
37	-1.592E+01	-1.517E+01	-1.523E+01	-1.517E+01	-1.523E+01	-1.534E+01			1.712E+01	1.612E+01	1.609E+01	1.612E+01	1.609E+01	1.620E+01		
38	-2.067E+01	-1.923E+01	-1.824E+01	-1.923E+01	-1.824E+01				2.188E+01	2.033E+01	1.953E+01	2.033E+01	1.953E+01			
39	-1.721E+01	-1.767E+01	-1.721E+01	-1.721E+01	-1.767E+01	-1.767E+01	-1.767E+01	-1.721E+01	1.794E+01	1.865E+01	1.794E+01	1.794E+01	1.865E+01	1.865E+01	1.865E+01	1.794E+01
40	-1.682E+01	-1.728E+01	-1.918E+01	-1.775E+01	-1.826E+01				1.752E+01	1.822E+01	1.992E+01	1.848E+01	1.921E+01			
41	-1.507E+01	-1.507E+01	-1.507E+01	-1.507E+01	-1.507E+01	-1.507E+01			1.638E+01	1.638E+01	1.638E+01	1.638E+01	1.638E+01	1.638E+01		
**42**	-1.711E+01	-1.753E+01	-1.919E+01	-1.753E+01					1.778E+01	1.800E+01	1.974E+01	1.800E+01				
43	-1.706E+01	-1.759E+01	-1.985E+01	-1.819E+01	-1.804E+01				1.771E+01	1.852E+01	2.075E+01	1.894E+01	1.872E+01			
44	-1.547E+01	-1.509E+01	-1.547E+01	-1.483E+01	-1.489E+01	-1.489E+01			1.672E+01	1.620E+01	1.672E+01	1.564E+01	1.584E+01	1.584E+01		
45	-1.434E+01	-1.483E+01	-1.441E+01	-1.434E+01	-1.483E+01				1.501E+01	1.578E+01	1.514E+01	1.501E+01	1.578E+01			
46	-2.285E+01	-2.193E+01	-2.193E+01	-2.285E+01					2.366E+01	2.262E+01	2.262E+01	2.366E+01				
47	-3.362E+01	-3.362E+01	-3.362E+01	-3.362E+01	-3.362E+01	-3.362E+01			3.430E+01	3.430E+01	3.430E+01	3.430E+01	3.430E+01	3.430E+01		
48	-1.355E+01	-1.435E+01	-1.435E+01	-1.402E+01	-1.402E+01				1.438E+01	1.558E+01	1.558E+01	1.508E+01	1.508E+01			
49	-3.298E+01	-3.362E+01	-2.808E+01	-3.298E+01	-2.964E+01	-2.964E+01			3.391E+01	3.430E+01	2.871E+01	3.391E+01	3.029E+01	3.029E+01		
50	-1.702E+01	-1.727E+01	-1.907E+01	-1.727E+01					1.798E+01	1.793E+01	1.988E+01	1.793E+01				
51	-1.609E+01	-1.609E+01	-1.546E+01	-1.546E+01	-1.546E+01	-1.546E+01			1.706E+01	1.706E+01	1.613E+01	1.613E+01	1.613E+01	1.613E+01		
52	-1.562E+01	-1.608E+01	-1.608E+01	-1.554E+01	-1.551E+01	-1.554E+01			1.635E+01	1.704E+01	1.704E+01	1.620E+01	1.612E+01	1.620E+01		
53	-1.149E+01	-1.149E+01	-1.206E+01						1.223E+01	1.223E+01	1.317E+01					
54	-1.193E+01	-1.193E+01	-1.193E+01						1.264E+01	1.264E+01	1.264E+01					
55	-1.584E+01	-1.591E+01	-1.584E+01	-1.643E+01	-1.584E+01	-1.584E+01			1.645E+01	1.652E+01	1.649E+01	1.740E+01	1.645E+01	1.649E+01		
56	-1.506E+01	-1.630E+01	-1.501E+01	-1.506E+01	-1.501E+01	-1.583E+01			1.583E+01	1.756E+01	1.564E+01	1.583E+01	1.564E+01	1.681E+01		
57	-1.629E+01	-1.515E+01	-1.567E+01	-1.510E+01	-1.522E+01	-1.496E+01			1.759E+01	1.596E+01	1.663E+01	1.587E+01	1.588E+01	1.554E+01		
58	-1.452E+01	-1.452E+01	-1.460E+01	-1.460E+01	-1.520E+01				1.511E+01	1.511E+01	1.525E+01	1.525E+01	1.618E+01			
59	-1.313E+01	-1.313E+01	-1.301E+01	-1.371E+01					1.378E+01	1.378E+01	1.358E+01	1.467E+01				
60	-1.955E+01	-1.768E+01	-1.768E+01	-1.672E+01					2.040E+01	1.833E+01	1.833E+01	1.728E+01				
61	-1.541E+01	-1.541E+01	-1.548E+01	-1.550E+01	-1.618E+01	-1.620E+01			1.599E+01	1.605E+01	1.606E+01	1.615E+01	1.722E+01	1.724E+01		
62	-1.246E+01	-1.246E+01	-1.325E+01	-1.325E+01					1.304E+01	1.304E+01	1.424E+01	1.424E+01				
63	-1.620E+01	-1.620E+01	-1.620E+01	-1.620E+01	-1.620E+01	-1.620E+01			1.680E+01	1.680E+01	1.680E+01	1.680E+01	1.680E+01	1.680E+01		
64	-1.728E+01	-1.721E+01	-1.735E+01	-1.728E+01	-1.720E+01	-1.735E+01	-1.720E+01		1.788E+01	1.781E+01	1.794E+01	1.788E+01	1.780E+01	1.794E+01	1.780E+01	
65	-1.601E+01	-1.601E+01	-1.458E+01	-1.458E+01	-1.458E+01	-1.458E+01			1.728E+01	1.728E+01	1.530E+01	1.530E+01	1.530E+01	1.530E+01		
66	-2.169E+01	-2.169E+01	-2.169E+01	-2.169E+01	-2.169E+01	-2.169E+01			2.228E+01	2.228E+01	2.228E+01	2.228E+01	2.228E+01	2.228E+01		
67	-1.831E+01	-1.828E+01	-1.829E+01	-1.822E+01	-1.831E+01	-1.822E+01	-1.853E+01	-1.829E+01	1.890E+01	1.887E+01	1.889E+01	1.882E+01	1.890E+01	1.882E+01	1.912E+01	1.889E+01
68	-1.617E+01	-1.617E+01	-1.617E+01	-1.628E+01	-1.617E+01	-1.628E+01			1.675E+01	1.675E+01	1.675E+01	1.688E+01	1.675E+01	1.688E+01		
69	-1.664E+01	-1.541E+01	-1.541E+01	-1.534E+01	-1.534E+01	-1.558E+01			1.789E+01	1.617E+01	1.617E+01	1.592E+01	1.592E+01	1.619E+01		
70	-1.492E+01	-1.497E+01	-1.494E+01	-1.484E+01	-1.485E+01				1.552E+01	1.557E+01	1.553E+01	1.541E+01	1.543E+01			
71	-1.344E+01	-1.344E+01	-1.344E+01	-1.344E+01					1.402E+01	1.402E+01	1.402E+01	1.402E+01				
72	-1.366E+01	-1.366E+01	-1.366E+01	-1.366E+01					1.403E+01	1.403E+01	1.403E+01	1.403E+01				
73	-1.401E+01	-1.401E+01	-1.404E+01	-1.404E+01	-1.525E+01				1.457E+01	1.457E+01	1.477E+01	1.477E+01	1.649E+01			

ωB97-XD

	Ellipticity	Rho

#	BCP1	BCP2	BCP3	BCP4	BCP5	BCP6	BCP7	BCP8	BCP1	BCP2	BCP3	BCP4	BCP5	BCP6	BCP7	BCP8
1	1.931E-01	1.931E-01	1.931E-01	1.931E-01	1.931E-01	1.931E-01			3.237E-01	3.237E-01	3.237E-01	3.237E-01	3.237E-01	3.237E-01		
2	1.902E-01	1.929E-01	1.922E-01	1.904E-01	1.924E-01	1.927E-01			3.235E-01	3.237E-01	3.239E-01	3.236E-01	3.241E-01	3.236E-01		
3	1.889E-01	1.890E-01	1.890E-01	1.934E-01	1.890E-01	1.934E-01			3.236E-01	3.236E-01	3.236E-01	3.236E-01	3.235E-01	3.236E-01		
4	2.001E-01	2.002E-01	1.966E-01	2.006E-01	1.966E-01	2.002E-01			3.336E-01	3.271E-01	3.179E-01	3.194E-01	3.179E-01	3.271E-01		
5	9.660E-02	9.665E-02	9.660E-02	9.666E-02	9.660E-02	9.666E-02	9.666E-02	9.660E-02	3.192E-01	3.192E-01	3.192E-01	3.192E-01	3.192E-01	3.192E-01	3.192E-01	3.192E-01
6	1.735E-01	1.839E-01	2.135E-01	1.722E-01	2.135E-01	1.735E-01			3.138E-01	3.144E-01	3.325E-01	3.212E-01	3.325E-01	3.138E-01		
7	1.699E-01	2.223E-01	1.629E-01	1.663E-01	1.671E-01	2.211E-01			3.115E-01	3.344E-01	3.134E-01	3.165E-01	3.123E-01	3.328E-01		
8	2.144E-01	2.164E-01	2.124E-01	2.170E-01	2.124E-01	2.162E-01	2.146E-01		3.086E-01	3.079E-01	3.079E-01	3.067E-01	3.069E-01	3.073E-01	3.074E-01	
9	2.401E-01	2.402E-01	2.401E-01	2.402E-01	2.402E-01				3.057E-01	3.056E-01	3.057E-01	3.057E-01	3.056E-01			
10	1.299E-01	8.819E-02	1.299E-01	1.891E-01	8.819E-02	1.315E-01	1.891E-01		3.249E-01	3.111E-01	3.249E-01	3.395E-01	3.111E-01	3.133E-01	3.395E-01	
11	2.507E-01	2.507E-01	2.507E-01	2.507E-01	2.507E-01	2.507E-01	2.507E-01	2.507E-01	3.035E-01	3.035E-01	3.035E-01	3.035E-01	3.035E-01	3.035E-01	3.035E-01	3.035E-01
12	2.261E-01	1.660E-01	1.683E-01	2.342E-01	1.683E-01	2.342E-01			3.278E-01	3.179E-01	3.076E-01	3.366E-01	3.076E-01	3.366E-01		
13	2.384E-01	1.509E-01	1.467E-01	1.519E-01	1.467E-01	2.384E-01			3.376E-01	3.076E-01	3.085E-01	3.099E-01	3.085E-01	3.376E-01		
14	1.486E-01	1.554E-01	2.320E-01	1.480E-01	1.617E-01	2.258E-01			3.032E-01	3.059E-01	3.393E-01	3.106E-01	3.149E-01	3.304E-01		
15	2.356E-01	1.521E-01	1.481E-01	1.453E-01	1.481E-01	2.356E-01			3.374E-01	3.073E-01	3.075E-01	3.098E-01	3.075E-01	3.374E-01		
16	1.410E-01	1.410E-01	1.691E-01	2.506E-01	1.691E-01	1.410E-01			3.058E-01	3.063E-01	3.149E-01	3.408E-01	3.148E-01	3.063E-01		
17	1.319E-01	1.895E-01	1.895E-01	1.896E-01	1.319E-01	1.896E-01			3.008E-01	3.225E-01	3.225E-01	3.226E-01	3.007E-01	3.226E-01		
18	1.410E-01	1.410E-01	1.410E-01	1.410E-01	1.410E-01	1.410E-01			3.058E-01	3.058E-01	3.059E-01	3.058E-01	3.059E-01	3.058E-01		
19	8.960E-02	8.962E-02	8.962E-02	8.960E-02					3.085E-01	3.085E-01	3.085E-01	3.085E-01				
20	6.179E-02	1.356E-01	1.368E-01	1.960E-01	1.368E-01	6.178E-02			3.010E-01	3.049E-01	3.230E-01	3.440E-01	3.230E-01	3.010E-01		
21	2.537E-01	2.537E-01	1.843E-01	2.054E-01	2.054E-01				2.995E-01	2.995E-01	2.975E-01	2.891E-01	2.891E-01			
22	1.859E-01	2.483E-01	1.859E-01	1.407E-01	2.482E-01	1.526E-01	1.407E-01		3.083E-01	3.302E-01	3.083E-01	2.976E-01	3.302E-01	2.880E-01	2.976E-01	
23	1.289E-01	2.654E-01	1.567E-01	2.654E-01	2.510E-01	1.567E-01			3.091E-01	3.392E-01	2.976E-01	3.392E-01	3.301E-01	2.976E-01		
24	1.434E-01	2.676E-01	1.464E-01	2.676E-01	2.564E-01	1.464E-01			3.156E-01	3.429E-01	2.975E-01	3.429E-01	3.344E-01	2.975E-01		
25	1.288E-01	1.319E-01	2.571E-01	1.338E-01	2.571E-01	1.288E-01			3.018E-01	3.008E-01	3.428E-01	3.029E-01	3.428E-01	3.018E-01		
26	1.526E-01	2.283E-01	2.213E-01	2.282E-01	2.213E-01				2.880E-01	3.127E-01	3.090E-01	3.126E-01	3.091E-01			
27	1.736E-01	1.736E-01	4.967E-03	1.736E-01	1.736E-01	4.965E-03			3.218E-01	3.218E-01	2.921E-01	3.218E-01	3.218E-01	2.921E-01		
28	1.886E-01	1.861E-01	1.886E-01	1.774E-01	1.861E-01	6.177E-02	1.774E-01		3.222E-01	3.231E-01	3.222E-01	3.266E-01	3.230E-01	2.683E-01	3.266E-01	
29	1.699E-01	1.137E-01	1.166E-01	2.749E-01	1.166E-01	1.699E-01			3.115E-01	2.866E-01	2.985E-01	3.499E-01	2.985E-01	3.115E-01		
30	6.177E-02	2.026E-01	2.051E-01	2.026E-01	2.051E-01				2.683E-01	3.180E-01	3.177E-01	3.180E-01	3.177E-01			
31	2.130E-02	1.306E-01	1.528E-01	1.306E-01	4.872E-02	1.528E-01			2.822E-01	3.273E-01	3.277E-01	3.273E-01	2.922E-01	3.277E-01		
32	2.096E-01	1.089E-01	1.021E-02	2.096E-01					2.923E-01	3.085E-01	2.496E-01	2.923E-01				
33	9.712E-02	1.839E-01	1.840E-01	9.712E-02	9.712E-02	1.840E-01			2.809E-01	3.144E-01	3.143E-01	2.809E-01	2.809E-01	3.143E-01		
34	2.715E-01	1.519E-01	2.718E-01	3.416E-01	1.515E-01	7.207E-02	3.417E-01		3.167E-01	2.815E-01	3.169E-01	3.492E-01	2.814E-01	2.642E-01	3.493E-01	
35	1.486E-01	8.163E-02	1.486E-01	1.486E-01	8.163E-02	1.486E-01			3.032E-01	2.747E-01	3.032E-01	3.032E-01	2.747E-01	3.032E-01		
36	8.770E-02	9.752E-02	2.130E-02	9.752E-02					3.062E-01	3.118E-01	2.822E-01	3.118E-01				
37	1.639E-02	1.518E-01	2.272E-01	1.516E-01	2.273E-01	1.951E-01			4.373E-01	3.081E-01	3.109E-01	3.079E-01	3.110E-01	3.157E-01		
38	1.315E-01	2.201E-02	1.589E-01	2.201E-02	1.589E-01				3.133E-01	2.719E-01	3.320E-01	2.719E-01	3.320E-01			
39	5.126E-02	3.460E-01	5.126E-02	5.126E-02	3.460E-01	3.460E-01	3.460E-01	5.126E-02	2.771E-01	3.571E-01	2.771E-01	2.771E-01	3.571E-01	3.572E-01	3.571E-01	2.771E-01
40	8.214E-02	3.279E-01	1.027E-01	6.809E-02	2.915E-01				2.638E-01	3.505E-01	2.905E-01	2.732E-01	3.498E-01			
41	1.256E-01	1.255E-01	1.255E-01	1.254E-01	1.256E-01	1.254E-01			3.417E-01	3.417E-01	3.417E-01	3.417E-01	3.417E-01	3.417E-01		
42	3.324E-01	5.545E-02	1.289E-01	5.545E-02					3.418E-01	2.501E-01	3.091E-01	2.501E-01				
43	4.532E-02	3.231E-01	2.933E-01	1.083E-01	3.748E-02				2.559E-01	3.494E-01	3.544E-01	2.818E-01	2.627E-01			
44	5.275E-02	8.704E-02	3.709E-02	2.411E-01	3.837E-02	2.128E-01			3.892E-01	3.190E-01	3.933E-01	2.971E-01	2.832E-01	3.162E-01		
45	4.322E-02	3.197E-01	1.014E-01	4.322E-02	3.197E-01				2.619E-01	3.533E-01	2.782E-01	2.619E-01	3.533E-01			
46	1.660E-01	3.814E-02	3.814E-02	1.660E-01					3.179E-01	2.518E-01	2.518E-01	3.179E-01				
47	8.721E-02	8.721E-02	8.719E-02	8.721E-02	8.719E-02	8.721E-02			2.696E-01	2.696E-01	2.696E-01	2.696E-01	2.696E-01	2.696E-01		
48	3.347E-02	2.863E-01	2.863E-01	9.030E-02	9.017E-02				2.358E-01	3.577E-01	3.578E-01	2.950E-01	2.950E-01			
49	3.405E-01	8.721E-02	1.919E-03	3.405E-01	4.553E-02	4.552E-02			3.512E-01	2.696E-01	2.528E-01	3.512E-01	2.622E-01	2.622E-01		
50	3.516E-01	3.223E-02	1.434E-01	3.223E-02					3.537E-01	2.464E-01	3.156E-01	2.464E-01				
51	3.608E-01	3.608E-01	3.602E-02	3.602E-02	3.603E-02	3.603E-02			3.618E-01	3.618E-01	2.613E-01	2.613E-01	2.613E-01	2.613E-01		
52	8.190E-02	3.278E-01	3.278E-01	3.430E-02	1.800E-05	3.430E-02			2.794E-01	3.578E-01	3.578E-01	2.590E-01	2.471E-01	2.590E-01		
53	6.478E-01	6.476E-01	1.949E-01						2.362E-01	2.362E-01	3.721E-01					
54	5.231E-01	5.231E-01	5.231E-01						2.431E-01	2.431E-01	2.431E-01					
55	3.077E-03	5.536E-03	3.571E-02	3.563E-01	3.083E-03	3.566E-02			2.476E-01	2.484E-01	2.595E-01	3.603E-01	2.475E-01	2.594E-01		
56	ND	ND	ND	ND	ND	ND			ND	ND	ND	ND	ND	ND		
57	1.302E-01	3.300E-02	3.592E-01	1.288E-02	4.487E-02	1.133E-02			4.526E-01	2.681E-01	3.483E-01	2.561E-01	2.535E-01	2.221E-01		
58	4.108E-03	4.107E-03	3.473E-02	3.473E-02	3.527E-01				2.399E-01	2.399E-01	2.573E-01	2.573E-01	3.625E-01			
59	3.025E-02	3.025E-02	3.116E-02	3.621E-01					2.527E-01	2.527E-01	2.293E-01	3.588E-01				
60	2.001E-01	2.147E-02	2.147E-02	2.042E-02					3.336E-01	2.506E-01	2.506E-01	2.262E-01				
61	8.041E-03	4.377E-02	1.485E-02	5.860E-02	2.661E-01	2.578E-01			2.311E-01	2.414E-01	2.309E-01	2.448E-01	3.649E-01	3.657E-01		
62	7.159E-03	7.159E-03	3.931E-01	3.931E-01					2.239E-01	2.239E-01	3.619E-01	3.619E-01				
63	3.670E-03	3.655E-03	3.655E-03	3.670E-03	3.670E-03	3.670E-03			2.476E-01	2.476E-01	2.476E-01	2.476E-01	2.476E-01	2.476E-01		
64	4.526E-03	5.575E-03	2.764E-03	4.525E-03	1.216E-02	2.765E-03	1.216E-02		2.460E-01	2.478E-01	2.452E-01	2.460E-01	2.445E-01	2.452E-01	2.445E-01	
65	ND	ND	ND	ND	ND	ND			ND	ND	ND	ND	ND	ND		
66	1.622E-03	1.622E-03	1.622E-03	1.622E-03	1.622E-03	1.622E-03			2.460E-01	2.460E-01	2.460E-01	2.460E-01	2.460E-01	2.460E-01		
67	2.385E-03	2.066E-03	1.476E-02	1.224E-02	2.389E-03	1.220E-02	2.052E-03	1.475E-02	2.439E-01	2.413E-01	2.455E-01	2.453E-01	2.440E-01	2.452E-01	2.424E-01	2.454E-01
68	4.478E-03	4.478E-03	4.478E-03	6.392E-03	4.478E-03	6.392E-03			2.420E-01	2.420E-01	2.420E-01	2.484E-01	2.420E-01	2.484E-01		
69	ND	ND	ND	ND	ND	ND			ND	ND	ND	ND	ND	ND		
70	4.595E-03	3.110E-03	2.840E-03	5.815E-03	4.417E-03				2.413E-01	2.467E-01	2.471E-01	2.360E-01	2.422E-01			
71	9.593E-03	9.593E-03	9.593E-03	9.593E-03					2.382E-01	2.382E-01	2.382E-01	2.382E-01				
72	1.164E-02	1.165E-02	1.164E-02	1.165E-02					2.383E-01	2.383E-01	2.383E-01	2.383E-01				
73	2.110E-02	3.255E-02	4.396E-01	7.204E-03	6.535E-02				2.191E-01	2.285E-01	1.751E-01	2.694E-01	4.024E-01			

	DelSqRho								V							
#	BCP1	BCP2	BCP3	BCP4	BCP5	BCP6	BCP7	BCP8	BCP1	BCP2	BCP3	BCP4	BCP5	BCP6	BCP7	BCP8
1	-1.072E+00	-1.072E+00	-1.072E+00	-1.072E+00	-1.072E+00	-1.072E+00			-4.833E-01	-4.834E-01	-4.833E-01	-4.833E-01	-4.833E-01	-4.834E-01		
2	-1.071E+00	-1.072E+00	-1.073E+00	-1.072E+00	-1.074E+00	-1.071E+00			-4.828E-01	-4.834E-01	-4.838E-01	-4.833E-01	-4.843E-01	-4.829E-01		
3	-1.071E+00	-1.071E+00	-1.071E+00	-1.072E+00	-1.071E+00	-1.072E+00			-4.829E-01	-4.829E-01	-4.830E-01	-4.831E-01	-4.828E-01	-4.832E-01		
4	-1.122E+00	-1.095E+00	-1.029E+00	-1.039E+00	-1.029E+00	-1.095E+00			-5.049E-01	-4.983E-01	-4.678E-01	-4.717E-01	-4.678E-01	-4.983E-01		
5	-1.072E+00	-1.071E+00	-1.072E+00	-1.071E+00	-1.071E+00	-1.071E+00	-1.071E+00	-1.072E+00	-4.596E-01	-4.596E-01	-4.596E-01	-4.596E-01	-4.596E-01	-4.596E-01	-4.596E-01	-4.596E-01
6	-1.007E+00	-1.004E+00	-1.126E+00	-1.064E+00	-1.126E+00	-1.007E+00			-4.520E-01	-4.526E-01	-5.122E-01	-4.733E-01	-5.122E-01	-4.520E-01		
7	-9.890E-01	-1.137E+00	-1.010E+00	-1.033E+00	-1.000E+00	-1.125E+00			-4.430E-01	-5.186E-01	-4.480E-01	-4.592E-01	-4.469E-01	-5.140E-01		
8	-9.726E-01	-9.676E-01	-9.670E-01	-9.588E-01	-9.599E-01	-9.633E-01	-9.640E-01		-4.435E-01	-4.419E-01	-4.419E-01	-4.388E-01	-4.391E-01	-4.400E-01	-4.401E-01	
9	-9.301E-01	-9.297E-01	-9.301E-01	-9.301E-01	-9.297E-01				-4.420E-01	-4.419E-01	-4.420E-01	-4.420E-01	-4.419E-01			
10	-1.101E+00	-1.020E+00	-1.101E+00	-1.189E+00	-1.020E+00	-1.013E+00	-1.189E+00		-4.800E-01	-4.331E-01	-4.800E-01	-5.313E-01	-4.331E-01	-4.418E-01	-5.313E-01	
11	-9.308E-01	-9.308E-01	-9.308E-01	-9.308E-01	-9.308E-01	-9.308E-01	-9.308E-01	-9.308E-01	-4.347E-01	-4.347E-01	-4.347E-01	-4.347E-01	-4.347E-01	-4.347E-01	-4.347E-01	-4.347E-01
12	-1.087E+00	-1.021E+00	-9.690E-01	-1.152E+00	-9.690E-01	-1.152E+00			-4.994E-01	-4.554E-01	-4.354E-01	-5.334E-01	-4.354E-01	-5.334E-01		
13	-1.153E+00	-9.687E-01	-9.830E-01	-9.927E-01	-9.830E-01	-1.153E+00			-5.303E-01	-4.299E-01	-4.322E-01	-4.390E-01	-4.322E-01	-5.303E-01		
14	-9.427E-01	-9.560E-01	-1.168E+00	-9.972E-01	-1.024E+00	-1.103E+00			-4.171E-01	-4.257E-01	-5.345E-01	-4.382E-01	-4.547E-01	-5.071E-01		
15	-1.152E+00	-9.664E-01	-9.755E-01	-9.926E-01	-9.755E-01	-1.152E+00			-5.298E-01	-4.294E-01	-4.296E-01	-4.386E-01	-4.296E-01	-5.298E-01		
16	-9.640E-01	-9.712E-01	-1.014E+00	-1.171E+00	-1.014E+00	-9.712E-01			-4.234E-01	-4.257E-01	-4.524E-01	-5.413E-01	-4.523E-01	-4.257E-01		
17	-9.304E-01	-1.064E+00	-1.064E+00	-1.064E+00	-9.301E-01	-1.064E+00			-4.091E-01	-4.771E-01	-4.771E-01	-4.775E-01	-4.090E-01	-4.775E-01		
18	-9.640E-01	-9.640E-01	-9.641E-01	-9.640E-01	-9.641E-01	-9.640E-01			-4.234E-01	-4.234E-01	-4.234E-01	-4.234E-01	-4.234E-01	-4.234E-01		
19	-9.636E-01	-9.640E-01	-9.640E-01	-9.636E-01					-4.449E-01	-4.451E-01	-4.451E-01	-4.449E-01				
20	-9.583E-01	-9.622E-01	-1.084E+00	-1.207E+00	-1.084E+00	-9.583E-01			-4.055E-01	-4.180E-01	-4.726E-01	-5.420E-01	-4.726E-01	-4.055E-01		
21	-8.891E-01	-8.891E-01	-8.692E-01	-8.329E-01	-8.329E-01				-4.253E-01	-4.253E-01	-4.084E-01	-3.969E-01	-3.969E-01			
22	-9.771E-01	-1.105E+00	-9.769E-01	-9.246E-01	-1.105E+00	-8.372E-01	-9.248E-01		-4.400E-01	-5.098E-01	-4.399E-01	-4.032E-01	-5.098E-01	-3.808E-01	-4.033E-01	
23	-9.717E-01	-1.158E+00	-9.068E-01	-1.158E+00	-1.095E+00	-9.068E-01			-4.298E-01	-5.470E-01	-4.069E-01	-5.470E-01	-5.103E-01	-4.069E-01		
24	-1.014E+00	-1.184E+00	-9.090E-01	-1.184E+00	-1.123E+00	-9.090E-01			-4.480E-01	-5.600E-01	-4.052E-01	-5.600E-01	-5.231E-01	-4.052E-01		
25	-9.447E-01	-9.304E-01	-1.182E+00	-9.520E-01	-1.182E+00	-9.447E-01			-4.116E-01	-4.091E-01	-5.486E-01	-4.175E-01	-5.486E-01	-4.116E-01		
26	-8.372E-01	-9.760E-01	-9.587E-01	-9.758E-01	-9.590E-01				-3.808E-01	-4.564E-01	-4.483E-01	-4.564E-01	-4.485E-01			
27	-1.063E+00	-1.062E+00	-9.107E-01	-1.062E+00	-1.063E+00	-9.107E-01			-5.018E-01	-5.018E-01	-3.671E-01	-5.018E-01	-5.018E-01	-3.671E-01		
28	-1.070E+00	-1.076E+00	-1.070E+00	-1.107E+00	-1.076E+00	-7.402E-01	-1.107E+00		-4.793E-01	-4.812E-01	-4.793E-01	-4.894E-01	-4.811E-01	-3.191E-01	-4.895E-01	
29	-9.890E-01	-8.482E-01	-9.272E-01	-1.227E+00	-9.272E-01	-9.890E-01			-4.430E-01	-3.680E-01	-4.012E-01	-5.723E-01	-4.012E-01	-4.430E-01		
30	-7.402E-01	-1.020E+00	-1.020E+00	-1.020E+00	-1.020E+00				-3.191E-01	-4.697E-01	-4.698E-01	-4.696E-01	-4.699E-01			
31	-8.176E-01	-1.128E+00	-1.104E+00	-1.128E+00	-8.944E-01	-1.104E+00			-3.442E-01	-5.123E-01	-4.896E-01	-5.123E-01	-3.761E-01	-4.896E-01		
32	-8.248E-01	-9.077E-01	-5.336E-01	-8.248E-01					-4.446E-01	-4.845E-01	-3.369E-01	-4.446E-01				
33	-8.175E-01	-1.004E+00	-1.004E+00	-8.170E-01	-8.175E-01	-1.004E+00			-3.513E-01	-4.526E-01	-4.525E-01	-3.511E-01	-3.513E-01	-4.525E-01		
34	-1.013E+00	-8.180E-01	-1.014E+00	-1.207E+00	-8.175E-01	-7.260E-01	-1.208E+00		-4.793E-01	-3.676E-01	-4.798E-01	-5.797E-01	-3.673E-01	-3.117E-01	-5.800E-01	
35	-9.427E-01	-7.826E-01	-9.427E-01	-9.427E-01	-7.827E-01	-9.427E-01			-4.171E-01	-3.341E-01	-4.171E-01	-4.171E-01	-3.341E-01	-4.171E-01		
36	-9.617E-01	-1.001E+00	-8.176E-01	-1.001E+00					-4.170E-01	-4.386E-01	-3.442E-01	-4.386E-01				
37	-1.769E+00	-9.651E-01	-9.736E-01	-9.641E-01	-9.740E-01	-1.014E+00			-8.970E-01	-4.824E-01	-4.547E-01	-4.819E-01	-4.548E-01	-4.594E-01		
38	-1.013E+00	-7.711E-01	-1.132E+00	-7.711E-01	-1.132E+00				-4.418E-01	-3.203E-01	-5.187E-01	-3.203E-01	-5.187E-01			
39	-8.107E-01	-1.259E+00	-8.107E-01	-8.107E-01	-1.259E+00	-1.259E+00	-1.259E+00	-8.107E-01	-3.403E-01	-6.071E-01	-3.403E-01	-3.403E-01	-6.071E-01	-6.071E-01	-6.071E-01	-3.403E-01
40	-7.072E-01	-1.204E+00	-8.509E-01	-7.739E-01	-1.209E+00				-3.120E-01	-5.820E-01	-3.776E-01	-3.387E-01	-5.826E-01			
41	-9.724E-01	-9.723E-01	-9.724E-01	-9.724E-01	-9.724E-01	-9.723E-01			-7.139E-01	-7.138E-01	-7.138E-01	-7.138E-01	-7.139E-01	-7.137E-01		
42	-1.143E+00	-6.152E-01	-9.717E-01	-6.152E-01					-5.546E-01	-2.903E-01	-4.298E-01	-2.903E-01				
43	-6.596E-01	-1.197E+00	-1.227E+00	-8.150E-01	-7.043E-01				-2.926E-01	-5.786E-01	-5.826E-01	-3.646E-01	-3.117E-01			
44	-1.260E+00	-9.131E-01	-1.309E+00	-8.719E-01	-7.701E-01	-1.005E+00			-8.752E-01	-5.774E-01	-9.095E-01	-4.273E-01	-4.101E-01	-5.600E-01		
45	-6.966E-01	-1.226E+00	-7.928E-01	-6.966E-01	-1.226E+00				-3.075E-01	-5.913E-01	-3.505E-01	-3.075E-01	-5.913E-01			
46	-1.021E+00	-6.308E-01	-6.308E-01	-1.021E+00					-4.554E-01	-2.918E-01	-2.918E-01	-4.554E-01				
47	-7.469E-01	-7.469E-01	-7.472E-01	-7.469E-01	-7.472E-01	-7.469E-01			-3.239E-01	-3.239E-01	-3.240E-01	-3.239E-01	-3.240E-01	-3.239E-01		
48	-5.688E-01	-1.265E+00	-1.265E+00	-9.098E-01	-9.095E-01				-2.341E-01	-6.117E-01	-6.118E-01	-4.109E-01	-4.108E-01			
49	-1.203E+00	-7.469E-01	-6.540E-01	-1.203E+00	-7.036E-01	-7.037E-01			-5.852E-01	-3.239E-01	-2.820E-01	-5.852E-01	-3.050E-01	-3.050E-01		
50	-1.218E+00	-5.996E-01	-1.014E+00	-5.996E-01					-5.934E-01	-2.789E-01	-4.480E-01	-2.789E-01				
51	-1.279E+00	-1.279E+00	-7.068E-01	-7.068E-01	-7.068E-01	-7.068E-01			-6.233E-01	-6.233E-01	-3.028E-01	-3.028E-01	-3.027E-01	-3.027E-01		
52	-8.153E-01	-1.260E+00	-1.260E+00	-6.893E-01	-6.163E-01	-6.893E-01			-3.494E-01	-6.056E-01	-6.056E-01	-2.976E-01	-2.693E-01	-2.976E-01		
53	-3.895E-01	-3.895E-01	-1.236E+00						-3.164E-01	-3.165E-01	-7.235E-01					

54	-4.995E-01	-4.995E-01	-4.995E-01						-3.127E-01	-3.127E-01	-3.127E-01					
55	-6.178E-01	-6.198E-01	-6.924E-01	-1.269E+00	-6.176E-01	-6.923E-01			-2.706E-01	-2.728E-01	-2.991E-01	-6.182E-01	-2.706E-01	-2.990E-01		
56	ND	ND	ND	ND	ND	ND			ND	ND	ND	ND	ND	ND		
57	-1.740E+00	-7.502E-01	-1.189E+00	-6.835E-01	-6.532E-01	-4.737E-01			-1.035E+00	-3.529E-01	-5.802E-01	-3.231E-01	-2.837E-01	-2.198E-01		
58	-5.702E-01	-5.702E-01	-6.733E-01	-6.733E-01	-1.282E+00				-2.548E-01	-2.548E-01	-2.961E-01	-2.961E-01	-6.246E-01			
59	-6.355E-01	-6.355E-01	-5.078E-01	-1.255E+00					-2.909E-01	-2.909E-01	-2.364E-01	-6.126E-01				
60	-1.122E+00	-6.281E-01	-6.281E-01	-4.898E-01					-5.049E-01	-2.857E-01	-2.857E-01	-2.286E-01				
61	-5.210E-01	-5.773E-01	-5.166E-01	-5.968E-01	-1.298E+00	-1.309E+00			-2.355E-01	-2.678E-01	-2.382E-01	-2.756E-01	-6.521E-01	-6.521E-01		
62	-4.773E-01	-4.773E-01	-1.268E+00	-1.268E+00					-2.282E-01	-2.282E-01	-6.258E-01	-6.258E-01				
63	-6.152E-01	-6.153E-01	-6.153E-01	-6.152E-01	-6.152E-01	-6.152E-01			-2.707E-01	-2.707E-01	-2.707E-01	-2.707E-01	-2.707E-01	-2.707E-01		
64	-6.060E-01	-6.178E-01	-6.016E-01	-6.060E-01	-5.986E-01	-6.016E-01	-5.986E-01		-2.672E-01	-2.711E-01	-2.654E-01	-2.672E-01	-2.639E-01	-2.654E-01	-2.638E-01	
65	ND	ND	ND	ND	ND	ND			ND	ND	ND	ND	ND	ND		
66	-6.001E-01	-6.001E-01	-6.001E-01	-6.001E-01	-6.001E-01	-6.001E-01			-2.664E-01	-2.664E-01	-2.664E-01	-2.664E-01	-2.664E-01	-2.664E-01		
67	-5.952E-01	-5.790E-01	-6.040E-01	-6.021E-01	-5.953E-01	-6.019E-01	-5.871E-01	-6.037E-01	-2.628E-01	-2.574E-01	-2.665E-01	-2.656E-01	-2.628E-01	-2.656E-01	-2.593E-01	-2.663E-01
68	-5.832E-01	-5.832E-01	-5.832E-01	-6.196E-01	-5.832E-01	-6.196E-01			-2.582E-01	-2.582E-01	-2.582E-01	-2.731E-01	-2.582E-01	-2.731E-01		
69	ND	ND	ND	ND	ND	ND			ND	ND	ND	ND	ND	ND		
70	-5.760E-01	-6.061E-01	-6.084E-01	-5.469E-01	-5.808E-01				-2.582E-01	-2.705E-01	-2.714E-01	-2.466E-01	-2.602E-01			
71	-5.557E-01	-5.557E-01	-5.557E-01	-5.557E-01					-2.544E-01	-2.544E-01	-2.544E-01	-2.544E-01				
72	-5.549E-01	-5.549E-01	-5.549E-01	-5.549E-01					-2.554E-01	-2.554E-01	-2.554E-01	-2.554E-01				
73	-4.552E-01	-5.056E-01	-1.750E-01	-7.698E-01	-1.273E+00				-2.098E-01	-2.382E-01	-1.741E-01	-3.642E-01	-1.011E+00			
	G								K							
#	BCP1	BCP2	BCP3	BCP4	BCP5	BCP6	BCP7	BCP8	BCP1	BCP2	BCP3	BCP4	BCP5	BCP6	BCP7	BCP8
1	1.077E-01	1.077E-01	1.077E-01	1.077E-01	1.077E-01	1.077E-01			3.757E-01	3.757E-01	3.757E-01	3.757E-01	3.757E-01	3.757E-01		
2	1.076E-01	1.077E-01	1.077E-01	1.077E-01	1.078E-01	1.076E-01			3.752E-01	3.757E-01	3.761E-01	3.756E-01	3.764E-01	3.753E-01		
3	1.076E-01	1.076E-01	1.076E-01	1.076E-01	1.075E-01	1.076E-01			3.753E-01	3.753E-01	3.754E-01	3.755E-01	3.753E-01	3.756E-01		
4	1.122E-01	1.122E-01	1.053E-01	1.060E-01	1.053E-01	1.122E-01			3.927E-01	3.861E-01	3.625E-01	3.657E-01	3.625E-01	3.861E-01		
5	9.587E-02	9.588E-02	9.587E-02	9.588E-02	9.587E-02	9.588E-02	9.588E-02	9.587E-02	3.637E-01	3.637E-01	3.637E-01	3.637E-01	3.637E-01	3.637E-01	3.637E-01	3.637E-01
6	1.002E-01	1.008E-01	1.154E-01	1.037E-01	1.154E-01	1.002E-01			3.518E-01	3.518E-01	3.968E-01	3.696E-01	3.968E-01	3.518E-01		
7	9.789E-02	1.172E-01	9.774E-02	1.005E-01	9.844E-02	1.164E-01			3.451E-01	4.014E-01	3.503E-01	3.587E-01	3.485E-01	3.976E-01		
8	1.002E-01	9.998E-02	1.001E-01	9.956E-02	9.957E-02	9.959E-02	9.954E-02		3.433E-01	3.419E-01	3.418E-01	3.393E-01	3.395E-01	3.404E-01	3.405E-01	
9	1.048E-01	1.047E-01	1.048E-01	1.048E-01	1.047E-01				3.373E-01	3.372E-01	3.373E-01	3.373E-01	3.372E-01			
10	1.024E-01	8.910E-02	1.024E-01	1.171E-01	8.910E-02	9.434E-02	1.171E-01		3.777E-01	3.440E-01	3.777E-01	4.142E-01	3.440E-01	3.475E-01	4.142E-01	
11	1.010E-01	1.010E-01	1.010E-01	1.010E-01	1.010E-01	1.010E-01	1.010E-01	1.010E-01	3.337E-01	3.337E-01	3.337E-01	3.337E-01	3.337E-01	3.337E-01	3.337E-01	3.337E-01
12	1.138E-01	1.001E-01	9.657E-02	1.226E-01	9.657E-02	1.226E-01			3.856E-01	3.553E-01	3.388E-01	4.108E-01	3.388E-01	4.108E-01		
13	1.211E-01	9.388E-02	9.324E-02	9.541E-02	9.324E-02	1.211E-01			4.092E-01	3.361E-01	3.390E-01	3.436E-01	3.390E-01	4.092E-01		
14	9.070E-02	9.336E-02	1.212E-01	9.443E-02	9.939E-02	1.157E-01			3.264E-01	3.324E-01	4.133E-01	3.437E-01	3.553E-01	3.914E-01		
15	1.210E-01	9.392E-02	9.285E-02	9.525E-02	9.285E-02	1.210E-01			4.089E-01	3.355E-01	3.367E-01	3.434E-01	3.367E-01	4.089E-01		
16	9.119E-02	9.146E-02	9.949E-02	1.243E-01	9.947E-02	9.146E-02			3.322E-01	3.342E-01	3.529E-01	4.170E-01	3.529E-01	3.343E-01		
17	8.827E-02	1.056E-01	1.056E-01	1.057E-01	8.824E-02	1.057E-01			3.209E-01	3.715E-01	3.715E-01	3.718E-01	3.208E-01	3.718E-01		
18	9.119E-02	9.119E-02	9.119E-02	9.119E-02	9.119E-02	9.119E-02			3.322E-01	3.322E-01	3.322E-01	3.322E-01	3.322E-01	3.322E-01		
19	1.020E-01	1.020E-01	1.020E-01	1.020E-01					3.429E-01	3.430E-01	3.430E-01	3.429E-01				
20	8.295E-02	8.874E-02	1.008E-01	1.201E-01	1.008E-01	8.295E-02			3.225E-01	3.293E-01	3.718E-01	4.219E-01	3.718E-01	3.225E-01		
21	1.015E-01	1.015E-01	9.556E-02	9.435E-02	9.435E-02				3.238E-01	3.238E-01	3.129E-01	3.026E-01	3.026E-01			
22	9.786E-02	1.168E-01	9.785E-02	8.604E-02	1.168E-01	8.573E-02	8.605E-02		3.421E-01	3.930E-01	3.421E-01	3.172E-01	3.930E-01	2.950E-01	3.172E-01	
23	9.342E-02	1.288E-01	9.009E-02	1.288E-01	1.182E-01	9.009E-02			3.364E-01	4.182E-01	3.168E-01	4.182E-01	3.920E-01	3.168E-01		
24	9.729E-02	1.320E-01	8.898E-02	1.320E-01	1.212E-01	8.898E-02			3.507E-01	4.280E-01	3.162E-01	4.280E-01	4.019E-01	3.162E-01		
25	8.770E-02	8.827E-02	1.265E-01	8.973E-02	1.265E-01	8.770E-02			3.239E-01	3.209E-01	4.221E-01	3.277E-01	4.221E-01	3.239E-01		
26	8.573E-02	1.062E-01	1.043E-01	1.062E-01	1.044E-01				2.950E-01	3.502E-01	3.440E-01	3.502E-01	3.441E-01			
27	1.181E-01	1.181E-01	6.970E-02	1.181E-01	1.181E-01	6.970E-02			3.837E-01	3.837E-01	2.974E-01	3.837E-01	3.837E-01	2.974E-01		
28	1.059E-01	1.061E-01	1.059E-01	1.064E-01	1.061E-01	6.704E-02	1.064E-01		3.733E-01	3.751E-01	3.734E-01	3.831E-01	3.750E-01	2.521E-01	3.831E-01	
29	9.789E-02	7.799E-02	8.468E-02	1.328E-01	8.468E-02	9.789E-02			3.451E-01	2.900E-01	3.165E-01	4.395E-01	3.165E-01	3.451E-01		
30	6.704E-02	1.073E-01	1.075E-01	1.073E-01	1.075E-01				2.521E-01	3.623E-01	3.624E-01	3.623E-01	3.624E-01			
31	6.989E-02	1.151E-01	1.067E-01	1.151E-01	7.627E-02	1.067E-01			2.743E-01	3.972E-01	3.828E-01	3.972E-01	2.999E-01	3.828E-01		
32	1.192E-01	1.288E-01	1.017E-01	1.192E-01					3.254E-01	3.557E-01	2.351E-01	3.254E-01				
33	7.347E-02	1.008E-01	1.008E-01	7.344E-02	7.347E-02	1.008E-01			2.778E-01	3.518E-01	3.517E-01	2.777E-01	2.778E-01	3.517E-01		
34	1.131E-01	8.158E-02	1.132E-01	1.389E-01	8.148E-02	6.509E-02	1.390E-01		3.663E-01	2.861E-01	3.666E-01	4.408E-01	2.858E-01	2.466E-01	4.410E-01	
35	9.070E-02	6.920E-02	9.070E-02	9.070E-02	6.920E-02	9.070E-02			3.264E-01	2.649E-01	3.264E-01	3.264E-01	2.649E-01	3.264E-01		
36	8.828E-02	9.422E-02	6.989E-02	9.422E-02					3.287E-01	3.444E-01	2.743E-01	3.444E-01				
37	2.274E-01	1.206E-01	1.057E-01	1.205E-01	1.057E-01	1.030E-01			6.696E-01	3.618E-01	3.491E-01	3.615E-01	3.492E-01	3.565E-01		
38	9.434E-02	6.376E-02	1.179E-01	6.376E-02	1.179E-01				3.475E-01	2.565E-01	4.009E-01	2.565E-01	4.009E-01			
39	6.883E-02	1.462E-01	6.883E-02	6.883E-02	1.462E-01	1.462E-01	1.462E-01	6.883E-02	2.715E-01	4.609E-01	2.715E-01	2.715E-01	4.609E-01	4.609E-01	4.609E-01	2.715E-01
40	6.759E-02	1.405E-01	8.243E-02	7.260E-02	1.401E-01				2.444E-01	4.415E-01	2.952E-01	2.661E-01	4.424E-01			
41	2.354E-01	2.354E-01	2.354E-01	2.353E-01	2.354E-01	2.353E-01			4.785E-01	4.784E-01	4.784E-01	4.785E-01	4.785E-01	4.784E-01		
42	1.344E-01	6.823E-02	9.342E-02	6.823E-02					4.201E-01	2.220E-01	3.364E-01	2.220E-01				
43	6.386E-02	1.397E-01	1.380E-01	8.043E-02	6.781E-02				2.287E-01	4.390E-01	4.446E-01	2.842E-01	2.439E-01			
44	2.801E-01	1.746E-01	2.911E-01	1.047E-01	1.088E-01	1.544E-01			5.951E-01	4.028E-01	6.184E-01	3.226E-01	3.013E-01	4.056E-01		
45	6.668E-02	1.425E-01	7.613E-02	6.668E-02	1.425E-01				2.408E-01	4.489E-01	2.743E-01	2.408E-01	4.489E-01			
46	1.001E-01	6.703E-02	6.703E-02	1.001E-01					3.553E-01	2.247E-01	2.247E-01	3.553E-01				
47	6.858E-02	6.858E-02	6.859E-02	6.858E-02	6.859E-02	6.858E-02			2.553E-01	2.553E-01	2.554E-01	2.553E-01	2.554E-01	2.553E-01		
48	4.597E-02	1.477E-01	1.478E-01	9.173E-02	9.169E-02				1.882E-01	4.640E-01	4.641E-01	3.192E-01	3.191E-01			
49	1.422E-01	6.858E-02	5.923E-02	1.422E-01	6.455E-02	6.456E-02			4.430E-01	2.553E-01	2.227E-01	4.430E-01	2.405E-01	2.405E-01		
50	1.444E-01	6.448E-02	9.729E-02	6.448E-02					4.490E-01	2.144E-01	3.507E-01	2.144E-01				
51	1.518E-01	1.518E-01	6.302E-02	6.302E-02	6.302E-02	6.302E-02			4.715E-01	4.715E-01	2.397E-01	2.397E-01	2.397E-01	2.397E-01		
52	7.277E-02	1.453E-01	1.453E-01	6.262E-02	5.761E-02	6.262E-02			2.766E-01	4.604E-01	4.604E-01	2.350E-01	2.117E-01	2.350E-01		
53	1.095E-01	1.095E-01	2.072E-01						2.069E-01	2.069E-01	5.163E-01					
54	9.390E-02	9.390E-02	9.390E-02						2.188E-01	2.188E-01	2.188E-01					
55	5.810E-02	5.891E-02	6.298E-02	1.505E-01	5.809E-02	6.297E-02			2.125E-01	2.139E-01	2.361E-01	4.677E-01	2.125E-01	2.361E-01		
56	ND	ND	ND	ND	ND	ND			ND	ND	ND	ND	ND	ND		
57	3.001E-01	8.266E-02	1.415E-01	7.609E-02	6.018E-02	5.069E-02			7.351E-01	2.702E-01	4.387E-01	2.470E-01	2.235E-01	1.691E-01		
58	5.613E-02	5.613E-02	6.388E-02	6.388E-02	1.521E-01				1.987E-01	1.987E-01	2.322E-01	2.322E-01	4.725E-01			
59	6.602E-02	6.602E-02	5.472E-02	1.494E-01					2.249E-01	2.249E-01	1.817E-01	4.632E-01				
60	1.122E-01	6.434E-02	6.434E-02	5.307E-02					3.927E-01	2.214E-01	2.214E-01	1.755E-01				
61	5.265E-02	6.174E-02	5.453E-02	6.320E-02	1.638E-01	1.625E-01			1.829E-01	2.061E-01	1.837E-01	2.124E-01	4.883E-01	4.897E-01		
62	5.443E-02	5.443E-02	1.544E-01	1.544E-01					1.738E-01	1.738E-01	4.714E-01	4.714E-01				
63	5.845E-02	5.846E-02	5.846E-02	5.845E-02	5.845E-02	5.845E-02			2.122E-01	2.123E-01	2.123E-01	2.122E-01	2.123E-01	2.123E-01		
64	5.785E-02	5.833E-02	5.748E-02	5.785E-02	5.709E-02	5.748E-02	5.709E-02		2.093E-01	2.128E-01	2.079E-01	2.093E-01	2.068E-01	2.079E-01	2.068E-01	
65	ND	ND	ND	ND	ND	ND			ND	ND	ND	ND	ND	ND		
66	5.818E-02	5.818E-02	5.818E-02	5.818E-02	5.818E-02	5.818E-02			2.082E-01	2.082E-01	2.082E-01	2.082E-01	2.082E-01	2.082E-01		
67	5.699E-02	5.631E-02	5.774E-02	5.754E-02	5.700E-02	5.753E-02	5.624E-02	5.771E-02	2.058E-01	2.011E-01	2.088E-01	2.081E-01	2.058E-01	2.080E-01	2.030E-01	2.086E-01
68	5.621E-02	5.621E-02	5.621E-02	5.911E-02	5.621E-02	5.911E-02			2.020E-01	2.020E-01	2.020E-01	2.140E-01	2.020E-01	2.140E-01		
69	ND	ND	ND	ND	ND	ND			ND	ND	ND	ND	ND	ND		
70	5.710E-02	5.948E-02	5.965E-02	5.493E-02	5.747E-02				2.011E-01	2.110E-01	2.118E-01	1.917E-01	2.027E-01			
71	5.776E-02	5.776E-02	5.776E-02	5.776E-02					1.967E-01	1.967E-01	1.967E-01	1.967E-01				
72	5.835E-02	5.835E-02	5.835E-02	5.835E-02					1.971E-01	1.971E-01	1.971E-01	1.971E-01				
73	4.800E-02	5.591E-02	6.517E-02	8.589E-02	3.462E-01				1.618E-01	1.823E-01	1.089E-01	2.783E-01	6.645E-01			
	L								ESP							
#	BCP1	BCP2	BCP3	BCP4	BCP5	BCP6	BCP7	BCP8	BCP1	BCP2	BCP3	BCP4	BCP5	BCP6	BCP7	BCP8
1	2.680E-01	2.680E-01	2.680E-01	2.680E-01	2.680E-01	2.680E-01			8.697E-01	8.698E-01	8.697E-01	8.697E-01	8.697E-01	8.698E-01		
2	2.676E-01	2.680E-01	2.683E-01	2.679E-01	2.686E-01	2.678E-01			7.726E-01	7.739E-01	7.735E-01	7.731E-01	7.740E-01	7.734E-01		
3	2.678E-01	2.678E-01	2.678E-01	2.679E-01	2.677E-01	2.679E-01			6.792E-01	6.792E-01	6.793E-01	6.816E-01	6.791E-01	6.817E-01		
4	2.805E-01	2.738E-01	2.572E-01	2.598E-01	2.572E-01	2.738E-01			8.559E-01	8.863E-01	8.520E-01	8.537E-01	8.520E-01	8.863E-01		
5	2.679E-01	2.679E-01	2.679E-01	2.679E-01	2.679E-01	2.679E-01	2.679E-01	2.679E-01	1.271E+00	1.271E+00	1.271E+00	1.271E+00	1.271E+00	1.271E+00	1.271E+00	1.271E+00
6	2.516E-01	2.510E-01	2.814E-01	2.660E-01	2.814E-01	2.516E-01			8.453E-01	8.439E-01	8.993E-01	8.631E-01	8.993E-01	8.453E-01		
7	2.473E-01	2.842E-01	2.525E-01	2.582E-01	2.500E-01	2.812E-01			8.332E-01	9.051E-01	8.391E-01	8.497E-01	8.401E-01	9.009E-01		
8	2.432E-01	2.419E-01	2.418E-01	2.397E-01	2.400E-01	2.408E-01	2.410E-01		5.521E-01	5.529E-01	5.486E-01	5.501E-01	5.458E-01	5.505E-01	5.488E-01	
9	2.325E-01	2.324E-01	2.325E-01	2.325E-01	2.324E-01				5.990E-01	5.988E-01	5.990E-01	5.990E-01	5.988E-01			
10	2.753E-01	2.549E-01	2.753E-01	2.971E-01	2.549E-01	2.531E-01	2.971E-01		1.247E+00	1.207E+00	1.247E+00	1.315E+00	1.207E+00	1.222E+00	1.315E+00	
11	2.327E-01	2.327E-01	2.327E-01	2.327E-01	2.327E-01	2.327E-01	2.327E-01	2.327E-01	4.700E-01	4.700E-01	4.700E-01	4.700E-01	4.700E-01	4.700E-01	4.700E-01	4.700E-01
12	2.718E-01	2.552E-01	2.423E-01	2.881E-01	2.423E-01	2.881E-01			8.869E-01	8.222E-01	8.293E-01	9.357E-01	8.293E-01	9.357E-01		
13	2.882E-01	2.422E-01	2.457E-01	2.482E-01	2.457E-01	2.882E-01			9.151E-01	8.187E-01	8.237E-01	8.303E-01	8.237E-01	9.151E-01		
14	2.357E-01	2.390E-01	2.921E-01	2.493E-01	2.559E-01	2.756E-01			8.114E-01	8.184E-01	9.202E-01	8.304E-01	8.473E-01	8.975E-01		
15	2.879E-01	2.416E-01	2.439E-01	2.481E-01	2.439E-01	2.879E-01			7.449E-01	6.500E-01	6.525E-01	6.583E-01	6.525E-01	7.449E-01		
16	2.410E-01	2.428E-01	2.535E-01	2.927E-01	2.534E-01	2.428E-01			8.202E-01	8.210E-01	8.455E-01	9.289E-01	8.455E-01	8.210E-01		
17	2.326E-01	2.659E-01	2.659E-01	2.661E-01	2.325E-01	2.661E-01			8.006E-01	8.687E-01	8.687E-01	8.691E-01	8.005E-01	8.691E-01		
18	2.410E-01	2.410E-01	2.410E-01	2.410E-01	2.410E-01	2.410E-01			8.202E-01	8.202E-01	8.202E-01	8.202E-01	8.202E-01	8.202E-01		
19	2.409E-01	2.410E-01	2.410E-01	2.409E-01					1.415E+00	1.415E+00	1.415E+00	1.415E+00				
20	2.396E-01	2.406E-01	2.710E-01	3.018E-01	2.710E-01	2.396E-01			1.190E+00	1.189E+00	1.259E+00	1.297E+00	1.259E+00	1.190E+00		
21	2.223E-01	2.223E-01	2.173E-01	2.082E-01	2.082E-01				4.486E-01	4.486E-01	3.917E-01	4.170E-01	4.170E-01			
22	2.443E-01	2.762E-01	2.442E-01	2.311E-01	2.762E-01	2.093E-01	2.312E-01		5.850E-01	6.540E-01	5.849E-01	5.479E-01	6.539E-01	5.039E-01	5.480E-01	
23	2.429E-01	2.895E-01	2.267E-01	2.895E-01	2.738E-01	2.267E-01			5.954E-01	7.449E-01	5.967E-01	7.449E-01	6.904E-01	5.967E-01		
24	2.534E-01	2.959E-01	2.272E-01	2.959E-01	2.807E-01	2.272E-01			8.138E-01	9.580E-01	7.930E-01	9.580E-01	9.010E-01	7.930E-01		
25	2.362E-01	2.326E-01	2.956E-01	2.380E-01	2.956E-01	2.362E-01			8.048E-01	8.006E-01	9.323E-01	8.103E-01	9.323E-01	8.048E-01		
26	2.093E-01	2.440E-01	2.397E-01	2.439E-01	2.398E-01				5.039E-01	5.776E-01	5.766E-01	5.775E-01	5.768E-01			
27	2.656E-01	2.656E-01	2.277E-01	2.656E-01	2.656E-01	2.277E-01			1.404E+00	1.404E+00	1.245E+00	1.404E+00	1.404E+00	1.245E+00		
28	2.674E-01	2.690E-01	2.675E-01	2.767E-01	2.690E-01	1.850E-01	2.767E-01		8.839E-01	8.870E-01	8.840E-01	8.940E-01	8.870E-01	6.998E-01	8.940E-01	
29	2.473E-01	2.120E-01	2.318E-01	3.067E-01	2.318E-01	2.473E-01			8.332E-01	7.627E-01	7.943E-01	9.545E-01	7.943E-01	8.332E-01		
30	1.850E-01	2.550E-01	2.549E-01	2.550E-01	2.550E-01				6.998E-01	8.472E-01	8.439E-01	8.472E-01	8.439E-01			
31	2.044E-01	2.820E-01	2.761E-01	2.820E-01	2.236E-01	2.761E-01			1.149E+00	1.375E+00	1.291E+00	1.375E+00	1.182E+00	1.291E+00		
32	2.062E-01	2.269E-01	1.334E-01	2.062E-01					9.274E-01	9.707E-01	7.949E-01	9.274E-01				

33	2.044E-01	2.510E-01	2.509E-01	2.043E-01	2.044E-01	2.509E-01			7.458E-01	8.439E-01	8.438E-01	7.456E-01	7.458E-01	8.438E-01		
34	2.532E-01	2.045E-01	2.534E-01	3.018E-01	2.044E-01	1.815E-01	3.020E-01		6.797E-01	5.682E-01	6.802E-01	7.740E-01	5.679E-01	5.074E-01	7.743E-01	
35	2.357E-01	1.957E-01	2.357E-01	2.357E-01	1.957E-01	2.357E-01			8.114E-01	7.288E-01	8.114E-01	8.114E-01	7.288E-01	8.114E-01		
36	2.404E-01	2.501E-01	2.044E-01	2.501E-01					1.246E+00	1.275E+00	1.149E+00	1.275E+00				
37	4.422E-01	2.413E-01	2.434E-01	2.410E-01	2.435E-01	2.535E-01			1.218E+00	9.645E-01	8.655E-01	9.639E-01	8.655E-01	8.611E-01		
38	2.531E-01	1.928E-01	2.830E-01	1.928E-01	2.830E-01				1.222E+00	1.100E+00	1.316E+00	1.100E+00	1.316E+00			
39	2.027E-01	3.146E-01	2.027E-01	2.027E-01	3.146E-01	3.146E-01	3.146E-01	2.027E-01	7.300E-01	9.865E-01	7.300E-01	7.300E-01	9.865E-01	9.865E-01	9.865E-01	7.300E-01
40	1.768E-01	3.010E-01	2.127E-01	1.935E-01	3.023E-01				6.943E-01	9.552E-01	7.352E-01	7.308E-01	9.689E-01			
41	2.431E-01	2.431E-01	2.431E-01	2.431E-01	2.431E-01	2.431E-01			1.351E+00	1.351E+00	1.351E+00	1.351E+00	1.351E+00	1.351E+00		
42	2.857E-01	1.538E-01	2.429E-01	1.538E-01					7.319E-01	4.742E-01	5.954E-01	4.742E-01				
43	1.649E-01	2.993E-01	3.067E-01	2.037E-01	1.761E-01				6.548E-01	9.443E-01	9.203E-01	7.475E-01	6.845E-01			
44	3.150E-01	2.283E-01	3.273E-01	2.180E-01	1.925E-01	2.512E-01			1.459E+00	1.139E+00	1.502E+00	8.755E-01	8.655E-01	1.173E+00		
45	1.742E-01	3.064E-01	1.982E-01	1.742E-01	3.064E-01				6.738E-01	9.562E-01	7.260E-01	6.738E-01	9.562E-01			
46	2.552E-01	1.577E-01	1.577E-01	2.552E-01					8.222E-01	6.849E-01	6.849E-01	8.222E-01				
47	1.867E-01	1.867E-01	1.868E-01	1.867E-01	1.868E-01	1.867E-01			6.881E-01	6.881E-01	6.882E-01	6.881E-01	6.882E-01	6.881E-01		
48	1.422E-01	3.163E-01	3.163E-01	2.274E-01	2.274E-01				8.454E-01	1.246E+00	1.246E+00	1.082E+00	1.082E+00			
49	3.008E-01	1.867E-01	1.635E-01	3.008E-01	1.759E-01	1.759E-01			9.405E-01	6.881E-01	6.323E-01	9.405E-01	6.609E-01	6.610E-01		
50	3.046E-01	1.499E-01	2.534E-01	1.499E-01					9.746E-01	6.644E-01	8.138E-01	6.644E-01				
51	3.198E-01	3.198E-01	1.767E-01	1.767E-01	1.767E-01	1.767E-01			9.863E-01	9.863E-01	6.710E-01	6.710E-01	6.710E-01	6.710E-01		
52	2.038E-01	3.151E-01	3.151E-01	1.723E-01	1.541E-01	1.723E-01			7.294E-01	9.711E-01	9.711E-01	6.632E-01	6.208E-01	6.632E-01		
53	9.737E-02	9.739E-02	3.091E-01						7.391E-01	7.390E-01	1.114E+00					
54	1.249E-01	1.249E-01	1.249E-01						7.122E-01	7.122E-01	7.122E-01					
55	1.544E-01	1.550E-01	1.731E-01	3.172E-01	1.544E-01	1.731E-01			6.144E-01	6.147E-01	6.591E-01	9.763E-01	6.144E-01	6.590E-01		
56	ND	ND	ND	ND	ND	ND			ND	ND	ND	ND	ND	ND		
57	4.351E-01	1.875E-01	2.972E-01	1.709E-01	1.633E-01	1.184E-01			1.382E+00	8.098E-01	9.710E-01	7.817E-01	6.631E-01	5.836E-01		
58	1.426E-01	1.426E-01	1.683E-01	1.683E-01	3.204E-01				5.944E-01	5.944E-01	6.542E-01	6.542E-01	9.801E-01			
59	1.589E-01	1.589E-01	1.269E-01	3.138E-01					6.469E-01	6.469E-01	5.739E-01	9.685E-01				
60	2.805E-01	1.570E-01	1.570E-01	1.225E-01					8.559E-01	6.509E-01	6.509E-01	5.669E-01				
61	1.302E-01	1.443E-01	1.292E-01	1.492E-01	3.246E-01	3.272E-01			5.791E-01	6.448E-01	5.859E-01	6.543E-01	1.052E+00	1.050E+00		
62	1.193E-01	1.193E-01	3.170E-01	3.170E-01					5.856E-01	5.856E-01	9.937E-01	9.937E-01				
63	1.538E-01	1.538E-01	1.538E-01	1.538E-01	1.538E-01	1.538E-01			6.073E-01	6.073E-01	6.073E-01	6.073E-01	6.073E-01	6.073E-01		
64	1.515E-01	1.545E-01	1.504E-01	1.515E-01	1.497E-01	1.504E-01	1.497E-01		6.027E-01	6.075E-01	6.006E-01	6.027E-01	5.987E-01	6.006E-01	5.987E-01	
65	ND	ND	ND	ND	ND	ND			ND	ND	ND	ND	ND	ND		
66	1.500E-01	1.500E-01	1.500E-01	1.500E-01	1.500E-01	1.500E-01			5.976E-01	5.976E-01	5.976E-01	5.976E-01	5.976E-01	5.976E-01		
67	1.488E-01	1.447E-01	1.510E-01	1.505E-01	1.488E-01	1.505E-01	1.468E-01	1.509E-01	5.978E-01	5.911E-01	6.017E-01	6.005E-01	5.978E-01	6.004E-01	5.921E-01	6.015E-01
68	1.458E-01	1.458E-01	1.458E-01	1.549E-01	1.458E-01	1.549E-01			5.914E-01	5.914E-01	5.914E-01	6.109E-01	5.914E-01	6.109E-01		
69	ND	ND	ND	ND	ND	ND			ND	ND	ND	ND	ND	ND		
70	1.440E-01	1.515E-01	1.521E-01	1.367E-01	1.452E-01				5.917E-01	6.075E-01	6.087E-01	5.767E-01	5.942E-01			
71	1.389E-01	1.389E-01	1.389E-01	1.389E-01					5.882E-01	5.882E-01	5.882E-01	5.882E-01				
72	1.387E-01	1.387E-01	1.387E-01	1.387E-01					3.686E-01	3.686E-01	3.686E-01	3.686E-01				
73	1.138E-01	1.264E-01	4.375E-02	1.924E-01	3.183E-01				5.658E-01	6.305E-01	5.580E-01	8.627E-01	1.622E+00			

	ESPe								ESPn							
#	BCP1	BCP2	BCP3	BCP4	BCP5	BCP6	BCP7	BCP8	BCP1	BCP2	BCP3	BCP4	BCP5	BCP6	BCP7	BCP8
1	-1.565E+01	-1.565E+01	-1.565E+01	-1.565E+01	-1.565E+01	-1.565E+01			1.652E+01	1.652E+01	1.652E+01	1.652E+01	1.652E+01	1.652E+01		
2	-1.574E+01	-1.574E+01	-1.574E+01	-1.575E+01	-1.574E+01	-1.574E+01			1.652E+01	1.652E+01	1.652E+01	1.652E+01	1.652E+01	1.652E+01		
3	-1.584E+01	-1.584E+01	-1.584E+01	-1.583E+01	-1.584E+01	-1.583E+01			1.652E+01	1.652E+01	1.652E+01	1.651E+01	1.652E+01	1.651E+01		
4	-1.961E+01	-1.843E+01	-1.759E+01	-1.742E+01	-1.759E+01	-1.843E+01			2.046E+01	1.932E+01	1.844E+01	1.827E+01	1.844E+01	1.932E+01		
5	-1.671E+01	-1.671E+01	-1.671E+01	-1.671E+01	-1.671E+01	-1.671E+01	-1.671E+01	-1.671E+01	1.799E+01	1.799E+01	1.799E+01	1.799E+01	1.799E+01	1.799E+01	1.799E+01	1.799E+01
6	-2.637E+01	-2.799E+01	-2.411E+01	-2.323E+01	-2.411E+01	-2.637E+01			2.722E+01	2.883E+01	2.501E+01	2.410E+01	2.501E+01	2.722E+01		
7	-2.469E+01	-2.133E+01	-2.287E+01	-2.090E+01	-2.372E+01	-2.183E+01			2.552E+01	2.224E+01	2.371E+01	2.175E+01	2.456E+01	2.273E+01		
8	-1.659E+01	-1.659E+01	-1.659E+01	-1.658E+01	-1.659E+01	-1.658E+01	-1.658E+01		1.714E+01	1.714E+01	1.714E+01	1.713E+01	1.713E+01	1.713E+01	1.713E+01	
9	-1.467E+01	-1.467E+01	-1.467E+01	-1.467E+01	-1.467E+01				1.527E+01	1.527E+01	1.527E+01	1.527E+01	1.527E+01			
10	-1.822E+01	-1.853E+01	-1.822E+01	-1.980E+01	-1.853E+01	-2.073E+01	-1.980E+01		1.946E+01	1.974E+01	1.946E+01	2.111E+01	1.974E+01	2.196E+01	2.111E+01	
11	-1.736E+01	-1.736E+01	-1.736E+01	-1.736E+01	-1.736E+01	-1.736E+01	-1.736E+01	-1.736E+01	1.783E+01	1.783E+01	1.783E+01	1.783E+01	1.783E+01	1.783E+01	1.783E+01	1.783E+01
12	-1.958E+01	-2.292E+01	-1.982E+01	-2.135E+01	-1.982E+01	-2.135E+01			2.046E+01	2.374E+01	2.065E+01	2.229E+01	2.065E+01	2.229E+01		
13	-1.910E+01	-2.135E+01	-2.020E+01	-1.858E+01	-2.020E+01	-1.910E+01			2.001E+01	2.217E+01	2.103E+01	1.941E+01	2.103E+01	2.001E+01		
14	-2.959E+01	-2.873E+01	-2.491E+01	-2.625E+01	-2.536E+01	-2.788E+01			3.040E+01	2.955E+01	2.583E+01	2.708E+01	2.621E+01	2.878E+01		
15	-1.926E+01	-2.151E+01	-2.035E+01	-1.875E+01	-2.035E+01	-1.926E+01			2.000E+01	2.216E+01	2.101E+01	1.941E+01	2.101E+01	2.000E+01		
16	-3.295E+01	-2.881E+01	-3.172E+01	-2.765E+01	-3.172E+01	-2.881E+01			3.377E+01	2.963E+01	3.257E+01	2.858E+01	3.256E+01	2.963E+01		
17	-2.431E+01	-2.367E+01	-2.367E+01	-2.367E+01	-2.431E+01	-2.367E+01			2.511E+01	2.454E+01	2.454E+01	2.454E+01	2.511E+01	2.454E+01		
18	-3.295E+01	-3.295E+01	-3.295E+01	-3.295E+01	-3.295E+01	-3.295E+01			3.377E+01	3.377E+01	3.377E+01	3.377E+01	3.377E+01	3.377E+01		
19	-1.262E+01	-1.262E+01	-1.262E+01	-1.262E+01					1.403E+01	1.403E+01	1.403E+01	1.403E+01				
20	-1.837E+01	-2.091E+01	-1.988E+01	-1.830E+01	-1.988E+01	-1.837E+01			1.956E+01	2.210E+01	2.114E+01	1.960E+01	2.114E+01	1.956E+01		
21	-1.738E+01	-1.738E+01	-1.957E+01	-1.823E+01	-1.823E+01				1.783E+01	1.783E+01	1.996E+01	1.865E+01	1.865E+01			
22	-1.874E+01	-1.927E+01	-1.874E+01	-2.018E+01	-1.927E+01	-2.126E+01	-2.018E+01		1.932E+01	1.992E+01	1.932E+01	2.073E+01	1.992E+01	2.176E+01	2.073E+01	
23	-1.926E+01	-1.831E+01	-1.737E+01	-1.831E+01	-1.741E+01	-1.737E+01			1.986E+01	1.906E+01	1.797E+01	1.906E+01	1.810E+01	1.797E+01		
24	-1.912E+01	-1.815E+01	-1.719E+01	-1.815E+01	-1.726E+01	-1.719E+01			1.993E+01	1.911E+01	1.798E+01	1.911E+01	1.816E+01	1.798E+01		
25	-2.276E+01	-2.431E+01	-2.126E+01	-2.052E+01	-2.126E+01	-2.276E+01			2.357E+01	2.511E+01	2.219E+01	2.133E+01	2.219E+01	2.357E+01		
26	-2.126E+01	-2.023E+01	-1.891E+01	-2.023E+01	-1.891E+01				2.176E+01	2.081E+01	1.949E+01	2.081E+01	1.949E+01			
27	-1.513E+01	-1.513E+01	-1.496E+01	-1.513E+01	-1.513E+01	-1.496E+01			1.654E+01	1.654E+01	1.621E+01	1.654E+01	1.654E+01	1.621E+01		
28	-1.863E+01	-1.902E+01	-1.863E+01	-2.016E+01	-1.902E+01	-2.090E+01	-2.016E+01		1.951E+01	1.991E+01	1.951E+01	2.106E+01	1.991E+01	2.160E+01	2.106E+01	
29	-2.469E+01	-2.461E+01	-2.315E+01	-2.253E+01	-2.315E+01	-2.469E+01			2.552E+01	2.537E+01	2.394E+01	2.348E+01	2.394E+01	2.552E+01		
30	-2.090E+01	-2.001E+01	-1.878E+01	-2.001E+01	-1.878E+01				2.160E+01	2.086E+01	1.962E+01	2.086E+01	1.962E+01			
31	-1.846E+01	-1.753E+01	-1.690E+01	-1.753E+01	-1.655E+01	-1.690E+01			1.961E+01	1.891E+01	1.819E+01	1.891E+01	1.773E+01	1.819E+01		
32	-1.265E+01	-1.287E+01	-1.235E+01	-1.265E+01					1.357E+01	1.384E+01	1.315E+01	1.357E+01				
33	-2.754E+01	-2.799E+01	-2.799E+01	-2.754E+01	-2.754E+01	-2.799E+01			2.829E+01	2.883E+01	2.883E+01	2.829E+01	2.829E+01	2.883E+01		
34	-1.665E+01	-1.640E+01	-1.665E+01	-1.679E+01	-1.640E+01	-1.626E+01	-1.680E+01		1.733E+01	1.697E+01	1.733E+01	1.757E+01	1.697E+01	1.676E+01	1.757E+01	
35	-2.959E+01	-2.895E+01	-2.959E+01	-2.959E+01	-2.895E+01	-2.959E+01			3.040E+01	2.968E+01	3.040E+01	3.040E+01	2.968E+01	3.040E+01		
36	-1.641E+01	-1.727E+01	-1.846E+01	-1.727E+01					1.766E+01	1.855E+01	1.961E+01	1.855E+01				
37	-1.597E+01	-1.519E+01	-1.528E+01	-1.519E+01	-1.528E+01	-1.537E+01			1.719E+01	1.616E+01	1.614E+01	1.616E+01	1.614E+01	1.623E+01		
38	-2.073E+01	-1.926E+01	-1.828E+01	-1.926E+01	-1.828E+01				2.196E+01	2.036E+01	1.960E+01	2.036E+01	1.960E+01			
39	-1.726E+01	-1.774E+01	-1.726E+01	-1.726E+01	-1.774E+01	-1.774E+01	-1.774E+01	-1.726E+01	1.799E+01	1.872E+01	1.799E+01	1.799E+01	1.872E+01	1.872E+01	1.872E+01	1.799E+01
40	-1.686E+01	-1.733E+01	-1.922E+01	-1.777E+01	-1.831E+01				1.755E+01	1.828E+01	1.995E+01	1.850E+01	1.928E+01			
41	-1.511E+01	-1.511E+01	-1.511E+01	-1.511E+01	-1.511E+01	-1.511E+01			1.647E+01	1.646E+01	1.646E+01	1.646E+01	1.647E+01	1.646E+01		
42	-1.720E+01	-1.751E+01	-1.926E+01	-1.751E+01					1.793E+01	1.799E+01	1.986E+01	1.799E+01				
43	-1.711E+01	-1.764E+01	-1.991E+01	-1.821E+01	-1.808E+01				1.777E+01	1.858E+01	2.083E+01	1.896E+01	1.877E+01			
44	-1.549E+01	-1.509E+01	-1.562E+01	-1.495E+01	-1.483E+01	-1.512E+01			1.695E+01	1.623E+01	1.712E+01	1.583E+01	1.569E+01	1.629E+01		
45	-1.438E+01	-1.487E+01	-1.443E+01	-1.438E+01	-1.487E+01				1.505E+01	1.583E+01	1.516E+01	1.505E+01	1.583E+01			
46	-2.292E+01	-2.197E+01	-2.197E+01	-2.292E+01					2.374E+01	2.266E+01	2.266E+01	2.374E+01				
47	-3.368E+01	-3.368E+01	-3.368E+01	-3.368E+01	-3.368E+01	-3.368E+01			3.437E+01	3.437E+01	3.437E+01	3.437E+01	3.437E+01	3.437E+01		
48	-1.360E+01	-1.439E+01	-1.439E+01	-1.406E+01	-1.406E+01				1.444E+01	1.563E+01	1.564E+01	1.514E+01	1.514E+01			
49	-3.307E+01	-3.368E+01	-2.814E+01	-3.307E+01	-2.970E+01	-2.970E+01			3.401E+01	3.437E+01	2.878E+01	3.401E+01	3.036E+01	3.036E+01		
50	-1.706E+01	-1.731E+01	-1.912E+01	-1.731E+01					1.804E+01	1.797E+01	1.993E+01	1.797E+01				
51	-1.612E+01	-1.612E+01	-1.550E+01	-1.550E+01	-1.550E+01	-1.550E+01			1.711E+01	1.711E+01	1.617E+01	1.617E+01	1.617E+01	1.617E+01		
52	-1.564E+01	-1.612E+01	-1.612E+01	-1.557E+01	-1.555E+01	-1.557E+01			1.637E+01	1.709E+01	1.709E+01	1.624E+01	1.617E+01	1.624E+01		
53	-1.151E+01	-1.151E+01	-1.209E+01						1.225E+01	1.225E+01	1.320E+01					
54	-1.197E+01	-1.197E+01	-1.197E+01						1.268E+01	1.268E+01	1.268E+01					
55	-1.589E+01	-1.596E+01	-1.587E+01	-1.647E+01	-1.589E+01	-1.587E+01			1.650E+01	1.657E+01	1.653E+01	1.744E+01	1.650E+01	1.653E+01		
56	ND	ND	ND	ND	ND	ND			ND	ND	ND	ND	ND	ND		
57	-1.635E+01	-1.515E+01	-1.571E+01	-1.512E+01	-1.525E+01	-1.501E+01			1.774E+01	1.596E+01	1.668E+01	1.590E+01	1.592E+01	1.559E+01		
58	-1.457E+01	-1.457E+01	-1.463E+01	-1.463E+01	-1.524E+01				1.516E+01	1.516E+01	1.529E+01	1.529E+01	1.622E+01			
59	-1.317E+01	-1.317E+01	-1.306E+01	-1.374E+01					1.381E+01	1.381E+01	1.363E+01	1.471E+01				
60	-1.961E+01	-1.773E+01	-1.773E+01	-1.678E+01					2.046E+01	1.838E+01	1.838E+01	1.734E+01				
61	-1.546E+01	-1.545E+01	-1.554E+01	-1.554E+01	-1.622E+01	-1.624E+01			1.604E+01	1.610E+01	1.612E+01	1.620E+01	1.727E+01	1.729E+01		
62	-1.251E+01	-1.251E+01	-1.329E+01	-1.329E+01					1.309E+01	1.309E+01	1.428E+01	1.428E+01				
63	-1.625E+01	-1.625E+01	-1.625E+01	-1.625E+01	-1.625E+01	-1.625E+01			1.685E+01	1.685E+01	1.685E+01	1.685E+01	1.685E+01	1.685E+01		
64	-1.734E+01	-1.726E+01	-1.741E+01	-1.734E+01	-1.726E+01	-1.741E+01	-1.726E+01		1.794E+01	1.787E+01	1.801E+01	1.794E+01	1.786E+01	1.801E+01	1.786E+01	
65	ND	ND	ND	ND	ND	ND			ND	ND	ND	ND	ND	ND		
66	-2.175E+01	-2.175E+01	-2.175E+01	-2.175E+01	-2.175E+01	-2.175E+01			2.235E+01	2.235E+01	2.235E+01	2.235E+01	2.235E+01	2.235E+01		
67	-1.837E+01	-1.835E+01	-1.836E+01	-1.828E+01	-1.837E+01	-1.828E+01	-1.861E+01	-1.836E+01	1.897E+01	1.894E+01	1.896E+01	1.888E+01	1.897E+01	1.888E+01	1.920E+01	1.896E+01
68	-1.622E+01	-1.622E+01	-1.622E+01	-1.633E+01	-1.622E+01	-1.633E+01			1.681E+01	1.681E+01	1.681E+01	1.694E+01	1.681E+01	1.694E+01		
69	ND	ND	ND	ND	ND	ND			ND	ND	ND	ND	ND	ND		
70	-1.493E+01	-1.500E+01	-1.501E+01	-1.487E+01	-1.494E+01				1.552E+01	1.561E+01	1.562E+01	1.545E+01	1.553E+01			
71	-1.348E+01	-1.348E+01	-1.348E+01	-1.348E+01					1.407E+01	1.407E+01	1.407E+01	1.407E+01				
72	-1.372E+01	-1.372E+01	-1.372E+01	-1.372E+01					1.409E+01	1.409E+01	1.409E+01	1.409E+01				
73	-1.405E+01	-1.407E+01	-1.384E+01	-1.434E+01	-1.497E+01				1.462E+01	1.470E+01	1.439E+01	1.520E+01	1.659E+01			

TPSSh

	Ellipticity								Rho							
#	BCP1	BCP2	BCP3	BCP4	BCP5	BCP6	BCP7	BCP8	BCP1	BCP2	BCP3	BCP4	BCP5	BCP6	BCP7	BCP8
1	2.007E-01	2.007E-01	2.007E-01	2.007E-01	2.007E-01	2.007E-01			3.204E-01	3.204E-01	3.204E-01	3.204E-01	3.204E-01	3.204E-01		
2	1.985E-01	1.985E-01	1.985E-01	1.985E-01	1.985E-01	1.985E-01			3.202E-01	3.202E-01	3.203E-01	3.202E-01	3.203E-01	3.202E-01		
3	1.972E-01	1.972E-01	1.972E-01	2.011E-01	1.972E-01	2.011E-01			3.198E-01	3.198E-01	3.198E-01	3.198E-01	3.198E-01	3.198E-01		
4	2.042E-01	2.025E-01	2.041E-01	2.079E-01	2.041E-01	2.025E-01			3.283E-01	3.229E-01	3.142E-01	3.155E-01	3.142E-01	3.229E-01		
5	1.048E-01	1.048E-01	1.048E-01	1.048E-01	1.048E-01	1.048E-01	1.048E-01	1.048E-01	3.145E-01	3.145E-01	3.145E-01	3.145E-01	3.145E-01	3.145E-01	3.145E-01	3.145E-01
6	1.804E-01	1.798E-01	2.201E-01	1.798E-01	2.201E-01	1.804E-01			3.105E-01	3.074E-01	3.281E-01	3.176E-01	3.281E-01	3.105E-01		
7	1.659E-01	2.270E-01	1.711E-01	1.757E-01	1.764E-01	2.256E-01			3.045E-01	3.296E-01	3.102E-01	3.135E-01	3.097E-01	3.278E-01		
8	2.308E-01	1.269E-01	1.762E-01	2.645E-01	1.717E-01	1.244E-01	2.351E-01		3.145E-01	3.009E-01	3.064E-01	3.192E-01	3.056E-01	3.012E-01	3.150E-01	
9	2.441E-01	2.441E-01	2.441E-01	2.441E-01	2.441E-01				3.020E-01	3.020E-01	3.020E-01	3.020E-01	3.020E-01			

10	1.370E-01	9.706E-02	1.370E-01	1.869E-01	9.706E-02	1.342E-01	1.869E-01		3.205E-01	3.068E-01	3.205E-01	3.329E-01	3.068E-01	3.082E-01	3.329E-01	
11	2.848E-01	1.807E-01	2.834E-01	1.810E-01	2.834E-01	1.810E-01	1.807E-01	2.848E-01	3.189E-01	2.899E-01	3.199E-01	2.897E-01	3.199E-01	2.897E-01	2.899E-01	3.189E-01
12	2.308E-01	1.676E-01	1.779E-01	2.332E-01	1.779E-01	2.332E-01			3.229E-01	3.125E-01	3.046E-01	3.312E-01	3.046E-01	3.312E-01		
13	2.399E-01	1.486E-01	1.589E-01	1.644E-01	1.589E-01	2.399E-01			3.321E-01	3.009E-01	3.065E-01	3.078E-01	3.065E-01	3.321E-01		
14	1.579E-01	1.533E-01	2.306E-01	1.602E-01	1.784E-01	2.188E-01			3.010E-01	2.988E-01	3.326E-01	3.087E-01	3.141E-01	3.227E-01		
15	2.340E-01	1.461E-01	1.667E-01	1.723E-01	1.667E-01	2.340E-01			3.275E-01	2.982E-01	3.051E-01	3.102E-01	3.051E-01	3.275E-01		
16	1.533E-01	1.543E-01	1.628E-01	2.491E-01	1.628E-01	1.543E-01			3.039E-01	3.048E-01	3.070E-01	3.342E-01	3.070E-01	3.048E-01		
17	1.310E-01	1.950E-01	1.950E-01	1.950E-01	1.310E-01	1.950E-01			2.939E-01	3.182E-01	3.182E-01	3.182E-01	2.939E-01	3.182E-01		
18	1.533E-01	1.533E-01	1.533E-01	1.533E-01	1.533E-01	1.533E-01			3.039E-01	3.039E-01	3.039E-01	3.039E-01	3.039E-01	3.039E-01		
19	8.457E-02	8.459E-02	8.459E-02	8.457E-02					3.048E-01	3.048E-01	3.048E-01	3.048E-01				
20	7.924E-02	1.450E-01	1.368E-01	1.899E-01	1.368E-01	7.924E-02			3.008E-01	3.040E-01	3.166E-01	3.369E-01	3.166E-01	3.008E-01		
21	2.362E-01	2.362E-01	1.946E-01	1.868E-01	1.868E-01				3.012E-01	3.012E-01	3.018E-01	2.930E-01	2.930E-01			
22	1.981E-01	2.367E-01	1.981E-01	1.637E-01	2.367E-01	1.299E-01	1.637E-01		3.089E-01	3.219E-01	3.089E-01	3.030E-01	3.219E-01	2.778E-01	3.030E-01	
23	1.277E-01	2.624E-01	1.765E-01	2.624E-01	2.554E-01	1.765E-01			2.998E-01	3.308E-01	2.955E-01	3.308E-01	3.223E-01	2.955E-01		
24	1.510E-01	2.649E-01	1.568E-01	2.649E-01	2.606E-01	1.568E-01			3.124E-01	3.370E-01	2.953E-01	3.370E-01	3.293E-01	2.953E-01		
25	1.454E-01	1.310E-01	2.541E-01	1.499E-01	2.541E-01	1.454E-01			3.019E-01	2.939E-01	3.359E-01	3.028E-01	3.359E-01	3.019E-01		
26	1.299E-01	2.224E-01	2.169E-01	2.224E-01	2.169E-01				2.778E-01	3.099E-01	3.081E-01	3.099E-01	3.081E-01			
27	1.774E-01	1.774E-01	1.046E-02	1.774E-01	1.774E-01	1.046E-02			3.158E-01	3.158E-01	2.940E-01	3.158E-01	3.158E-01	2.941E-01		
28	1.961E-01	1.926E-01	1.961E-01	1.864E-01	1.926E-01	6.973E-02	1.865E-01		3.181E-01	3.184E-01	3.181E-01	3.228E-01	3.184E-01	2.650E-01	3.228E-01	
29	1.659E-01	1.283E-01	1.336E-01	2.701E-01	1.336E-01	1.659E-01			3.045E-01	2.863E-01	2.986E-01	3.425E-01	2.986E-01	3.045E-01		
30	6.973E-02	2.055E-01	2.057E-01	2.055E-01	2.058E-01				2.650E-01	3.141E-01	3.136E-01	3.141E-01	3.136E-01			
31	2.777E-02	1.366E-01	1.532E-01	1.366E-01	6.223E-02	1.532E-01			2.775E-01	3.245E-01	3.220E-01	3.245E-01	2.900E-01	3.220E-01		
32	2.041E-01	8.129E-02	3.265E-03	2.041E-01					2.846E-01	3.015E-01	2.516E-01	2.846E-01				
33	1.138E-01	1.798E-01	1.798E-01	1.138E-01	1.138E-01	1.798E-01			2.820E-01	3.074E-01	3.074E-01	2.821E-01	2.820E-01	3.074E-01		
34	2.686E-01	1.581E-01	2.683E-01	3.404E-01	1.584E-01	8.078E-02	3.403E-01		3.131E-01	2.818E-01	3.130E-01	3.429E-01	2.819E-01	2.650E-01	3.429E-01	
35	1.579E-01	1.010E-01	1.579E-01	1.579E-01	1.010E-01	1.579E-01			3.010E-01	2.770E-01	3.010E-01	3.010E-01	2.770E-01	3.010E-01		
36	8.626E-02	1.018E-01	2.777E-02	1.018E-01					3.006E-01	3.078E-01	2.775E-01	3.078E-01				
37	1.596E-02	1.577E-01	2.309E-01	1.575E-01	2.310E-01	2.085E-01			4.279E-01	3.054E-01	3.055E-01	3.053E-01	3.056E-01	3.139E-01		
38	1.342E-01	3.919E-02	1.577E-01	3.919E-02	1.577E-01				3.082E-01	2.724E-01	3.270E-01	2.724E-01	3.270E-01			
39	6.363E-02	3.455E-01	6.363E-02	6.363E-02	3.455E-01	3.455E-01	3.455E-01	6.363E-02	2.774E-01	3.508E-01	2.774E-01	2.774E-01	3.508E-01	3.508E-01	3.508E-01	2.774E-01
40	9.650E-02	3.235E-01	1.219E-01	8.190E-02	2.740E-01				2.650E-01	3.430E-01	2.915E-01	2.732E-01	3.404E-01			
41	1.274E-01	1.274E-01	1.274E-01	1.274E-01	1.274E-01	1.274E-01			3.342E-01	3.342E-01	3.342E-01	3.342E-01	3.342E-01	3.342E-01		
42	3.116E-01	8.146E-02	1.277E-01	8.146E-02					3.254E-01	2.542E-01	2.998E-01	2.542E-01				
43	4.961E-02	3.214E-01	2.855E-01	1.203E-01	3.793E-02				2.543E-01	3.427E-01	3.462E-01	2.821E-01	2.607E-01			
44	2.855E-02	1.243E-02	1.461E-02	2.524E-01	8.004E-02	1.818E-01			3.912E-01	3.337E-01	3.822E-01	2.793E-01	2.921E-01	3.091E-01		
45	4.728E-02	3.207E-01	1.123E-01	4.728E-02	3.207E-01				2.605E-01	3.473E-01	2.781E-01	2.605E-01	3.473E-01			
46	1.676E-01	4.778E-02	4.778E-02	1.676E-01					3.125E-01	2.524E-01	2.524E-01	3.125E-01				
47	9.945E-02	9.945E-02	9.950E-02	9.945E-02	9.950E-02	9.945E-02			2.693E-01	2.693E-01	2.693E-01	2.693E-01	2.693E-01	2.693E-01		
48	2.534E-02	2.848E-01	2.848E-01	9.621E-02	9.618E-02				2.322E-01	3.527E-01	3.527E-01	2.923E-01	2.923E-01			
49	3.326E-01	9.945E-02	5.800E-05	3.325E-01	5.082E-02	5.079E-02			3.430E-01	2.693E-01	2.505E-01	3.430E-01	2.605E-01	2.605E-01		
50	3.452E-01	3.789E-02	1.510E-01	3.789E-02					3.469E-01	2.460E-01	3.124E-01	2.460E-01				
51	3.664E-01	3.664E-01	4.145E-02	4.145E-02	4.145E-02	4.145E-02			3.566E-01	3.566E-01	2.605E-01	2.605E-01	2.605E-01	2.605E-01		
52	9.777E-02	3.292E-01	3.292E-01	3.962E-02	1.352E-03	3.962E-02			2.805E-01	3.517E-01	3.517E-01	2.578E-01	2.452E-01	2.578E-01		
53	6.145E-01	6.142E-01	2.040E-01						2.367E-01	2.367E-01	3.640E-01					
54	5.024E-01	5.025E-01	5.024E-01						2.416E-01	2.416E-01	2.416E-01					
55	2.896E-03	5.485E-03	4.124E-02	3.619E-01	2.892E-03	4.122E-02			2.457E-01	2.466E-01	2.586E-01	3.550E-01	2.458E-01	2.586E-01		
56	1.762E-02	1.532E-01	4.190E-02	1.762E-02	3.719E-01	4.190E-02			2.575E-01	4.452E-01	2.382E-01	2.575E-01	3.529E-01	2.382E-01		
57	1.265E-01	4.603E-02	3.598E-01	1.464E-02	5.016E-02	7.676E-03			4.416E-01	2.695E-01	3.413E-01	2.555E-01	2.534E-01	2.191E-01		
58	5.516E-03	5.515E-03	3.882E-02	3.882E-02	3.571E-01				2.380E-01	2.380E-01	2.564E-01	2.564E-01	3.570E-01			
59	3.104E-02	3.104E-02	3.400E-02	3.627E-01					2.519E-01	2.519E-01	2.278E-01	3.532E-01				
60	2.042E-01	2.344E-02	2.344E-02	2.228E-02					3.283E-01	2.492E-01	2.492E-01	2.245E-01				
61	5.230E-03	4.930E-02	1.345E-02	6.426E-02	2.657E-01	2.565E-01			2.300E-01	2.397E-01	2.287E-01	2.435E-01	3.591E-01	3.602E-01		
62	7.260E-03	7.260E-03	3.966E-01	3.966E-01					2.215E-01	2.215E-01	3.568E-01	3.568E-01				
63	3.106E-03	3.103E-03	3.103E-03	3.106E-03	3.105E-03	3.105E-03			2.458E-01	2.458E-01	2.458E-01	2.458E-01	2.458E-01	2.458E-01		
64	4.054E-03	4.197E-03	2.762E-03	4.053E-03	9.457E-03	2.763E-03	9.457E-03		2.442E-01	2.459E-01	2.434E-01	2.442E-01	2.425E-01	2.434E-01	2.425E-01	
65	1.174E-01	1.174E-01	3.732E-02	4.039E-02	4.036E-02	3.731E-02			4.214E-01	4.214E-01	2.340E-01	2.426E-01	2.426E-01	2.340E-01		
66	5.090E-04	5.090E-04	5.090E-04	5.090E-04	5.090E-04	5.090E-04			2.446E-01	2.446E-01	2.446E-01	2.446E-01	2.446E-01	2.446E-01		
67	1.860E-03	2.134E-03	1.246E-02	9.089E-03	1.874E-03	9.076E-03	8.100E-05	1.248E-02	2.417E-01	2.395E-01	2.435E-01	2.432E-01	2.417E-01	2.432E-01	2.404E-01	2.435E-01
68	2.708E-03	2.708E-03	2.708E-03	6.384E-03	2.708E-03	6.384E-03			2.403E-01	2.403E-01	2.403E-01	2.466E-01	2.403E-01	2.466E-01		
69	1.489E-02	1.488E-02	7.295E-03	7.292E-03	9.225E-03	1.459E-01			2.554E-01	2.554E-01	2.245E-01	2.245E-01	2.416E-01	4.428E-01		
70	3.845E-03	9.700E-04	3.445E-03	6.177E-03	5.797E-03				2.427E-01	2.454E-01	2.437E-01	2.354E-01	2.366E-01			
71	1.167E-02	1.167E-02	1.167E-02	1.167E-02					2.368E-01	2.368E-01	2.368E-01	2.368E-01				
72	1.336E-02	1.336E-02	1.336E-02	1.336E-02					2.366E-01	2.366E-01	2.366E-01	2.366E-01				
73	3.628E-02	1.521E-02	5.708E-03	4.481E-01	7.355E-02				2.270E-01	2.154E-01	2.682E-01	1.731E-01	3.903E-01			
	DelSqRho								V							
#	BCP1	BCP2	BCP3	BCP4	BCP5	BCP6	BCP7	BCP8	BCP1	BCP2	BCP3	BCP4	BCP5	BCP6	BCP7	BCP8
1	-1.050E+00	-1.050E+00	-1.050E+00	-1.050E+00	-1.050E+00	-1.050E+00			-4.760E-01	-4.760E-01	-4.760E-01	-4.760E-01	-4.760E-01	-4.760E-01		
2	-1.049E+00	-1.049E+00	-1.050E+00	-1.049E+00	-1.050E+00	-1.049E+00			-4.757E-01	-4.757E-01	-4.760E-01	-4.757E-01	-4.760E-01	-4.757E-01		
3	-1.046E+00	-1.046E+00	-1.046E+00	-1.047E+00	-1.046E+00	-1.047E+00			-4.743E-01	-4.743E-01	-4.744E-01	-4.746E-01	-4.743E-01	-4.746E-01		
4	-1.086E+00	-1.067E+00	-1.005E+00	-1.014E+00	-1.005E+00	-1.067E+00			-4.927E-01	-4.885E-01	-4.598E-01	-4.630E-01	-4.598E-01	-4.885E-01		
5	-1.039E+00	-1.039E+00	-1.039E+00	-1.039E+00	-1.039E+00	-1.039E+00	-1.039E+00	-1.039E+00	-4.481E-01	-4.481E-01	-4.481E-01	-4.481E-01	-4.481E-01	-4.481E-01	-4.481E-01	-4.481E-01
6	-9.850E-01	-9.617E-01	-1.097E+00	-1.040E+00	-1.097E+00	-9.850E-01			-4.452E-01	-4.346E-01	-5.015E-01	-4.654E-01	-5.015E-01	-4.452E-01		
7	-9.474E-01	-1.105E+00	-9.891E-01	-1.013E+00	-9.822E-01	-1.092E+00			-4.253E-01	-5.063E-01	-4.420E-01	-4.531E-01	-4.425E-01	-5.013E-01		
8	-9.995E-01	-9.523E-01	-9.685E-01	-1.018E+00	-9.651E-01	-9.554E-01	-1.001E+00		-4.652E-01	-4.133E-01	-4.348E-01	-4.812E-01	-4.320E-01	-4.136E-01	-4.670E-01	
9	-9.063E-01	-9.063E-01	-9.063E-01	-9.063E-01	-9.063E-01				-4.342E-01	-4.342E-01	-4.342E-01	-4.342E-01	-4.342E-01			
10	-1.071E+00	-9.904E-01	-1.071E+00	-1.145E+00	-9.904E-01	-9.782E-01	-1.145E+00		-4.687E-01	-4.232E-01	-4.687E-01	-5.121E-01	-4.232E-01	-4.300E-01	-5.121E-01	
11	-1.021E+00	-8.627E-01	-1.028E+00	-8.614E-01	-1.028E+00	-8.614E-01	-8.627E-01	-1.021E+00	-4.852E-01	-3.918E-01	-4.882E-01	-3.913E-01	-4.882E-01	-3.913E-01	-3.918E-01	-4.852E-01
12	-1.056E+00	-9.839E-01	-9.492E-01	-1.117E+00	-9.492E-01	-1.117E+00			-4.873E-01	-4.430E-01	-4.296E-01	-5.194E-01	-4.296E-01	-5.194E-01		
13	-1.117E+00	-9.290E-01	-9.685E-01	-9.776E-01	-9.685E-01	-1.117E+00			-5.153E-01	-4.133E-01	-4.301E-01	-4.359E-01	-4.301E-01	-5.153E-01		
14	-9.277E-01	-9.135E-01	-1.126E+00	-9.835E-01	-1.016E+00	-1.055E+00			-4.142E-01	-4.084E-01	-5.155E-01	-4.363E-01	-4.560E-01	-4.851E-01		
15	-1.088E+00	-9.122E-01	-9.569E-01	-9.901E-01	-9.569E-01	-1.088E+00			-5.014E-01	-4.061E-01	-4.276E-01	-4.444E-01	-4.277E-01	-5.014E-01		
16	-9.499E-01	-9.599E-01	-9.666E-01	-1.129E+00	-9.665E-01	-9.599E-01			-4.219E-01	-4.252E-01	-4.318E-01	-5.227E-01	-4.318E-01	-4.252E-01		
17	-8.891E-01	-1.036E+00	-1.036E+00	-1.036E+00	-8.891E-01	-1.036E+00			-3.925E-01	-4.677E-01	-4.677E-01	-4.677E-01	-3.925E-01	-4.677E-01		
18	-9.499E-01	-9.499E-01	-9.500E-01	-9.499E-01	-9.500E-01	-9.499E-01			-4.219E-01	-4.219E-01	-4.220E-01	-4.219E-01	-4.220E-01	-4.219E-01		
19	-9.336E-01	-9.339E-01	-9.338E-01	-9.337E-01					-4.363E-01	-4.363E-01	-4.363E-01	-4.363E-01				
20	-9.542E-01	-9.564E-01	-1.042E+00	-1.164E+00	-1.042E+00	-9.542E-01			-4.066E-01	-4.185E-01	-4.546E-01	-5.204E-01	-4.546E-01	-4.066E-01		
21	-9.039E-01	-9.039E-01	-8.975E-01	-8.634E-01	-8.634E-01				-4.291E-01	-4.291E-01	-4.232E-01	-4.076E-01	-4.075E-01			
22	-9.791E-01	-1.054E+00	-9.791E-01	-9.540E-01	-1.054E+00	-7.804E-01	-9.540E-01		-4.445E-01	-4.858E-01	-4.445E-01	-4.231E-01	-4.858E-01	-3.540E-01	-4.231E-01	
23	-9.088E-01	-1.103E+00	-8.909E-01	-1.103E+00	-1.045E+00	-8.909E-01			-4.085E-01	-5.235E-01	-4.050E-01	-5.235E-01	-4.893E-01	-4.050E-01		
24	-9.900E-01	-1.146E+00	-8.949E-01	-1.146E+00	-1.090E+00	-8.949E-01			-4.428E-01	-5.436E-01	-4.020E-01	-5.436E-01	-5.099E-01	-4.020E-01		
25	-9.428E-01	-8.891E-01	-1.139E+00	-9.496E-01	-1.139E+00	-9.428E-01			-4.157E-01	-3.925E-01	-5.287E-01	-4.204E-01	-5.287E-01	-4.157E-01		
26	-7.804E-01	-9.608E-01	-9.543E-01	-9.608E-01	-9.543E-01				-3.540E-01	-4.501E-01	-4.464E-01	-4.501E-01	-4.464E-01			
27	-1.023E+00	-1.023E+00	-9.205E-01	-1.023E+00	-1.023E+00	-9.205E-01			-4.836E-01	-4.836E-01	-3.755E-01	-4.836E-01	-4.836E-01	-3.755E-01		
28	-1.042E+00	-1.044E+00	-1.042E+00	-1.079E+00	-1.044E+00	-7.191E-01	-1.079E+00		-4.698E-01	-4.697E-01	-4.698E-01	-4.817E-01	-4.697E-01	-3.138E-01	-4.817E-01	
29	-9.474E-01	-8.440E-01	-9.253E-01	-1.181E+00	-9.253E-01	-9.474E-01			-4.253E-01	-3.712E-01	-4.053E-01	-5.506E-01	-4.053E-01	-4.253E-01		
30	-7.191E-01	-9.938E-01	-9.929E-01	-9.938E-01	-9.929E-01				-3.138E-01	-4.602E-01	-4.594E-01	-4.601E-01	-4.595E-01			
31	-7.853E-01	-1.110E+00	-1.069E+00	-1.110E+00	-8.797E-01	-1.069E+00			-3.356E-01	-5.017E-01	-4.734E-01	-5.017E-01	-3.726E-01	-4.734E-01		
32	-7.623E-01	-8.542E-01	-5.352E-01	-7.623E-01					-4.270E-01	-4.673E-01	-3.471E-01	-4.270E-01				
33	-8.221E-01	-9.617E-01	-9.616E-01	-8.222E-01	-8.221E-01	-9.616E-01			-3.583E-01	-4.346E-01	-4.346E-01	-3.584E-01	-3.583E-01	-4.346E-01		
34	-9.903E-01	-8.191E-01	-9.897E-01	-1.167E+00	-8.196E-01	-7.299E-01	-1.167E+00		-4.689E-01	-3.700E-01	-4.686E-01	-5.619E-01	-3.703E-01	-3.168E-01	-5.617E-01	
35	-9.277E-01	-7.938E-01	-9.277E-01	-9.277E-01	-7.938E-01	-9.277E-01			-4.142E-01	-3.442E-01	-4.142E-01	-4.142E-01	-3.442E-01	-4.142E-01		
36	-9.237E-01	-9.708E-01	-7.853E-01	-9.708E-01					-4.029E-01	-4.300E-01	-3.356E-01	-4.300E-01				
37	-1.704E+00	-9.440E-01	-9.388E-01	-9.430E-01	-9.392E-01	-1.001E+00			-8.662E-01	-4.772E-01	-4.424E-01	-4.767E-01	-4.425E-01	-4.574E-01		
38	-9.782E-01	-7.707E-01	-1.100E+00	-7.707E-01	-1.100E+00				-4.300E-01	-3.243E-01	-4.998E-01	-3.243E-01	-4.998E-01			
39	-8.113E-01	-1.218E+00	-8.113E-01	-8.113E-01	-1.218E+00	-1.218E+00	-1.218E+00	-8.113E-01	-3.441E-01	-5.889E-01	-3.442E-01	-3.441E-01	-5.889E-01	-5.889E-01	-5.889E-01	-3.441E-01
40	-7.124E-01	-1.155E+00	-8.537E-01	-7.708E-01	-1.149E+00				-3.177E-01	-5.596E-01	-3.850E-01	-3.418E-01	-5.541E-01			
41	-9.169E-01	-9.169E-01	-9.170E-01	-9.169E-01	-9.169E-01	-9.170E-01			-7.016E-01	-7.016E-01	-7.016E-01	-7.015E-01	-7.015E-01	-7.016E-01		
42	-1.034E+00	-6.326E-01	-9.088E-01	-6.326E-01					-5.062E-01	-3.046E-01	-4.085E-01	-3.046E-01				
43	-6.512E-01	-1.153E+00	-1.174E+00	-8.139E-01	-6.936E-01				-2.905E-01	-5.594E-01	-5.588E-01	-3.689E-01	-3.086E-01			
44	-1.416E+00	-1.032E+00	-1.289E+00	-7.556E-01	-8.243E-01	-9.535E-01			-7.696E-01	-6.124E-01	-8.443E-01	-3.824E-01	-4.387E-01	-5.004E-01		
45	-6.891E-01	-1.185E+00	-7.911E-01	-6.891E-01	-1.185E+00				-3.057E-01	-5.743E-01	-3.527E-01	-3.057E-01	-5.743E-01			
46	-9.839E-01	-6.315E-01	-6.315E-01	-9.839E-01					-4.430E-01	-2.962E-01	-2.962E-01	-4.430E-01				
47	-7.448E-01	-7.448E-01	-7.448E-01	-7.448E-01	-7.448E-01	-7.448E-01			-3.268E-01	-3.268E-01	-3.269E-01	-3.268E-01	-3.269E-01	-3.268E-01		
48	-5.491E-01	-1.232E+00	-1.232E+00	-8.916E-01	-8.915E-01				-2.281E-01	-5.958E-01	-5.958E-01	-4.019E-01	-4.018E-01			
49	-1.150E+00	-7.448E-01	-6.435E-01	-1.150E+00	-6.944E-01	-6.943E-01			-5.613E-01	-3.268E-01	-2.782E-01	-5.613E-01	-3.035E-01	-3.034E-01		
50	-1.171E+00	-5.952E-01	-9.900E-01	-5.952E-01					-5.736E-01	-2.801E-01	-4.428E-01	-2.801E-01				
51	-1.244E+00	-1.244E+00	-7.033E-01	-7.033E-01	-7.032E-01	-7.032E-01			-6.092E-01	-6.092E-01	-3.026E-01	-3.026E-01	-3.025E-01	-3.025E-01		
52	-8.207E-01	-1.221E+00	-1.221E+00	-6.839E-01	-6.093E-01	-6.839E-01			-3.551E-01	-5.883E-01	-5.883E-01	-2.966E-01	-2.665E-01	-2.966E-01		
53	-3.961E-01	-3.962E-01	-1.173E+00						-3.179E-01	-3.179E-01	-6.996E-01					
54	-4.936E-01	-4.935E-01	-4.936E-01						-3.090E-01	-3.090E-01	-3.090E-01					
55	-6.108E-01	-6.132E-01	-6.891E-01	-1.233E+00	-6.110E-01	-6.891E-01			-2.680E-01	-2.703E-01	-2.991E-01	-6.036E-01	-2.680E-01	-2.991E-01		
56	-6.916E-01	-1.724E+00	-5.666E-01	-6.916E-01	-1.211E+00	-5.666E-01			-3.254E-01	-9.764E-01	-2.563E-01	-3.254E-01	-5.996E-01	-2.563E-01		
57	-1.685E+00	-7.546E-01	-1.143E+00	-6.791E-01	-6.544E-01	-4.612E-01			-9.862E-01	-3.597E-01	-5.612E-01	-3.233E-01	-2.853E-01	-2.161E-01		
58	-5.625E-01	-5.625E-01	-6.691E-01	-6.691E-01	-1.243E+00				-2.519E-01	-2.519E-01	-2.956E-01	-2.956E-01	-6.091E-01			
59	-6.308E-01	-6.308E-01	-5.017E-01	-1.214E+00					-2.903E-01	-2.903E-01	-2.342E-01	-5.971E-01				
60	-1.086E+00	-6.204E-01	-6.204E-01	-4.831E-01					-4.927E-01	-2.841E-01	-2.841E-01	-2.260E-01				
61	-5.182E-01	-5.696E-01	-5.085E-01	-5.914E-01	-1.257E+00	-1.270E+00			-2.345E-01	-2.663E-01	-2.355E-01	-2.749E-01	-6.359E-01	-6.358E-01		
62	-4.647E-01	-4.647E-01	-1.229E+00	-1.229E+00					-2.245E-01	-2.245E-01	-6.118E-01	-6.118E-01				
63	-6.083E-01	-6.085E-01	-6.085E-01	-6.083E-01	-6.084E-01	-6.084E-01			-2.682E-01	-2.683E-01	-2.683E-01	-2.682E-01	-2.683E-01	-2.683E-01		

64	-5.991E-01	-6.096E-01	-5.947E-01	-5.991E-01	-5.906E-01	-5.947E-01	-5.906E-01		-2.648E-01	-2.683E-01	-2.629E-01	-2.648E-01	-2.610E-01	-2.629E-01	-2.610E-01	
65	-1.368E+00	-1.368E+00	-5.441E-01	-6.215E-01	-6.217E-01	-5.441E-01			-1.053E+00	-1.053E+00	-2.654E-01	-2.900E-01	-2.900E-01	-2.654E-01		
66	-5.963E-01	-5.963E-01	-5.963E-01	-5.963E-01	-5.963E-01	-5.963E-01			-2.653E-01	-2.653E-01	-2.653E-01	-2.653E-01	-2.653E-01	-2.653E-01		
67	-5.855E-01	-5.720E-01	-5.959E-01	-5.936E-01	-5.855E-01	-5.934E-01	-5.786E-01	-5.957E-01	-2.594E-01	-2.550E-01	-2.636E-01	-2.627E-01	-2.594E-01	-2.627E-01	-2.562E-01	-2.635E-01
68	-5.777E-01	-5.777E-01	-5.777E-01	-6.130E-01	-5.777E-01	-6.131E-01			-2.562E-01	-2.562E-01	-2.562E-01	-2.706E-01	-2.562E-01	-2.706E-01		
69	-6.788E-01	-6.788E-01	-4.878E-01	-4.878E-01	-5.826E-01	-1.708E+00			-3.222E-01	-3.222E-01	-2.265E-01	-2.265E-01	-2.577E-01	-9.665E-01		
70	-5.867E-01	-6.014E-01	-5.920E-01	-5.461E-01	-5.528E-01				-2.628E-01	-2.690E-01	-2.650E-01	-2.464E-01	-2.491E-01			
71	-5.498E-01	-5.498E-01	-5.498E-01	-5.498E-01					-2.526E-01	-2.526E-01	-2.526E-01	-2.526E-01				
72	-5.473E-01	-5.473E-01	-5.473E-01	-5.473E-01					-2.529E-01	-2.529E-01	-2.529E-01	-2.529E-01				
73	-4.995E-01	-4.375E-01	-7.608E-01	-1.668E-01	-1.183E+00				-2.361E-01	-2.041E-01	-3.616E-01	-1.726E-01	-9.814E-01			
	G								K							
#	BCP1	BCP2	BCP3	BCP4	BCP5	BCP6	BCP7	BCP8	BCP1	BCP2	BCP3	BCP4	BCP5	BCP6	BCP7	BCP8
1	1.067E-01	1.067E-01	1.067E-01	1.067E-01	1.067E-01	1.067E-01			3.693E-01	3.693E-01	3.693E-01	3.693E-01	3.693E-01	3.693E-01		
2	1.067E-01	1.067E-01	1.068E-01	1.067E-01	1.068E-01	1.067E-01			3.690E-01	3.690E-01	3.692E-01	3.690E-01	3.692E-01	3.690E-01		
3	1.064E-01	1.064E-01	1.064E-01	1.064E-01	1.064E-01	1.064E-01			3.679E-01	3.679E-01	3.680E-01	3.681E-01	3.679E-01	3.682E-01		
4	1.106E-01	1.108E-01	1.043E-01	1.047E-01	1.043E-01	1.108E-01			3.821E-01	3.777E-01	3.555E-01	3.582E-01	3.555E-01	3.777E-01		
5	9.424E-02	9.424E-02	9.424E-02	9.424E-02	9.424E-02	9.424E-02	9.424E-02	9.424E-02	3.539E-01	3.539E-01	3.539E-01	3.539E-01	3.539E-01	3.539E-01	3.539E-01	3.539E-01
6	9.947E-02	9.709E-02	1.137E-01	1.026E-01	1.137E-01	9.947E-02			3.457E-01	3.375E-01	3.878E-01	3.627E-01	3.878E-01	3.457E-01		
7	9.420E-02	1.150E-01	9.736E-02	9.999E-02	9.845E-02	1.141E-01			3.310E-01	3.913E-01	3.446E-01	3.531E-01	3.440E-01	3.872E-01		
8	1.077E-01	8.762E-02	9.633E-02	1.133E-01	9.536E-02	8.736E-02	1.084E-01		3.576E-01	3.257E-01	3.385E-01	3.678E-01	3.366E-01	3.262E-01	3.586E-01	
9	1.038E-01	1.038E-01	1.038E-01	1.038E-01	1.038E-01				3.304E-01	3.304E-01	3.304E-01	3.304E-01	3.304E-01			
10	1.004E-01	8.782E-02	1.004E-01	1.129E-01	8.782E-02	9.271E-02	1.129E-01		3.682E-01	3.354E-01	3.682E-01	3.992E-01	3.354E-01	3.373E-01	3.992E-01	
11	1.150E-01	8.805E-02	1.156E-01	8.797E-02	1.156E-01	8.796E-02	8.805E-02	1.150E-01	3.703E-01	3.037E-01	3.726E-01	3.033E-01	3.726E-01	3.033E-01	3.037E-01	3.702E-01
12	1.116E-01	9.852E-02	9.613E-02	1.200E-01	9.613E-02	1.200E-01			3.756E-01	3.445E-01	3.334E-01	3.994E-01	3.334E-01	3.994E-01		
13	1.180E-01	9.052E-02	9.397E-02	9.575E-02	9.397E-02	1.180E-01			3.973E-01	3.228E-01	3.361E-01	3.401E-01	3.361E-01	3.973E-01		
14	9.113E-02	9.001E-02	1.171E-01	9.520E-02	1.010E-01	1.107E-01			3.231E-01	3.184E-01	3.985E-01	3.411E-01	3.550E-01	3.744E-01		
15	1.147E-01	8.901E-02	9.421E-02	9.844E-02	9.421E-02	1.147E-01			3.867E-01	3.171E-01	3.334E-01	3.460E-01	3.334E-01	3.867E-01		
16	9.223E-02	9.261E-02	9.509E-02	1.202E-01	9.508E-02	9.262E-02			3.297E-01	3.326E-01	3.367E-01	4.025E-01	3.367E-01	3.326E-01		
17	8.510E-02	1.044E-01	1.044E-01	1.044E-01	8.510E-02	1.044E-01			3.074E-01	3.633E-01	3.633E-01	3.633E-01	3.074E-01	3.633E-01		
18	9.223E-02	9.223E-02	9.224E-02	9.223E-02	9.224E-02	9.223E-02			3.297E-01	3.297E-01	3.297E-01	3.297E-01	3.297E-01	3.297E-01		
19	1.014E-01	1.014E-01	1.014E-01	1.014E-01					3.348E-01	3.349E-01	3.349E-01	3.348E-01				
20	8.401E-02	8.969E-02	9.704E-02	1.148E-01	9.704E-02	8.401E-02			3.226E-01	3.288E-01	3.575E-01	4.057E-01	3.575E-01	3.226E-01		
21	1.015E-01	1.015E-01	9.943E-02	9.586E-02	9.585E-02				3.275E-01	3.275E-01	3.238E-01	3.117E-01	3.117E-01			
22	9.987E-02	1.111E-01	9.987E-02	9.231E-02	1.111E-01	7.946E-02	9.231E-02		3.447E-01	3.747E-01	3.447E-01	3.308E-01	3.747E-01	2.746E-01	3.308E-01	
23	9.064E-02	1.238E-01	9.115E-02	1.238E-01	1.141E-01	9.115E-02			3.178E-01	3.997E-01	3.139E-01	3.997E-01	3.753E-01	3.139E-01		
24	9.765E-02	1.286E-01	8.913E-02	1.286E-01	1.187E-01	8.913E-02			3.452E-01	4.150E-01	3.129E-01	4.150E-01	3.912E-01	3.129E-01		
25	9.000E-02	8.510E-02	1.220E-01	9.152E-02	1.220E-01	9.000E-02			3.257E-01	3.074E-01	4.067E-01	3.289E-01	4.067E-01	3.257E-01		
26	7.946E-02	1.049E-01	1.039E-01	1.049E-01	1.039E-01				2.746E-01	3.451E-01	3.425E-01	3.451E-01	3.425E-01			
27	1.139E-01	1.139E-01	7.270E-02	1.139E-01	1.139E-01	7.271E-02			3.697E-01	3.697E-01	3.028E-01	3.697E-01	3.697E-01	3.028E-01		
28	1.047E-01	1.043E-01	1.047E-01	1.060E-01	1.043E-01	6.704E-02	1.060E-01		3.651E-01	3.654E-01	3.651E-01	3.757E-01	3.654E-01	2.468E-01	3.757E-01	
29	9.420E-02	8.010E-02	8.700E-02	1.277E-01	8.700E-02	9.420E-02			3.310E-01	2.911E-01	3.183E-01	4.229E-01	3.183E-01	3.310E-01		
30	6.704E-02	1.059E-01	1.056E-01	1.059E-01	1.056E-01				2.468E-01	3.543E-01	3.538E-01	3.543E-01	3.538E-01			
31	6.963E-02	1.121E-01	1.031E-01	1.121E-01	7.632E-02	1.031E-01			2.660E-01	3.895E-01	3.703E-01	3.895E-01	2.963E-01	3.703E-01		
32	1.182E-01	1.269E-01	1.067E-01	1.182E-01					3.088E-01	3.404E-01	2.405E-01	3.088E-01				
33	7.641E-02	9.709E-02	9.708E-02	7.642E-02	7.641E-02	9.708E-02			2.819E-01	3.375E-01	3.375E-01	2.820E-01	2.819E-01	3.375E-01		
34	1.106E-01	8.263E-02	1.106E-01	1.351E-01	8.270E-02	6.719E-02	1.350E-01		3.582E-01	2.874E-01	3.580E-01	4.268E-01	2.876E-01	2.497E-01	4.267E-01	
35	9.113E-02	7.287E-02	9.113E-02	9.113E-02	7.287E-02	9.113E-02			3.231E-01	2.713E-01	3.231E-01	3.231E-01	2.713E-01	3.231E-01		
36	8.598E-02	9.363E-02	6.963E-02	9.363E-02					3.169E-01	3.363E-01	2.660E-01	3.363E-01				
37	2.201E-01	1.206E-01	1.038E-01	1.205E-01	1.038E-01	1.035E-01			6.461E-01	3.566E-01	3.385E-01	3.562E-01	3.386E-01	3.539E-01		
38	9.271E-02	6.582E-02	1.125E-01	6.582E-02	1.125E-01				3.373E-01	2.585E-01	3.874E-01	2.585E-01	3.874E-01			
39	7.066E-02	1.423E-01	7.066E-02	7.066E-02	1.423E-01	1.423E-01	1.423E-01	7.066E-02	2.735E-01	4.467E-01	2.735E-01	2.735E-01	4.467E-01	4.467E-01	4.467E-01	2.735E-01
40	6.981E-02	1.354E-01	8.579E-02	7.455E-02	1.334E-01				2.479E-01	4.242E-01	2.992E-01	2.672E-01	4.207E-01			
41	2.362E-01	2.362E-01	2.362E-01	2.362E-01	2.362E-01	2.362E-01			4.654E-01	4.654E-01	4.654E-01	4.654E-01	4.654E-01	4.654E-01		
42	1.238E-01	7.325E-02	9.064E-02	7.325E-02					3.824E-01	2.314E-01	3.178E-01	2.314E-01				
43	6.387E-02	1.356E-01	1.327E-01	8.269E-02	6.762E-02				2.267E-01	4.238E-01	4.261E-01	2.862E-01	2.410E-01			
44	2.078E-01	1.772E-01	2.610E-01	9.674E-02	1.163E-01	1.310E-01			5.618E-01	4.351E-01	5.833E-01	2.856E-01	3.224E-01	3.694E-01		
45	6.672E-02	1.390E-01	7.746E-02	6.672E-02	1.390E-01				2.390E-01	4.353E-01	2.752E-01	2.390E-01	4.353E-01			
46	9.852E-02	6.915E-02	6.915E-02	9.852E-02					3.445E-01	2.270E-01	2.270E-01	3.445E-01				
47	7.032E-02	7.032E-02	7.033E-02	7.032E-02	7.033E-02	7.032E-02			2.565E-01	2.565E-01	2.565E-01	2.565E-01	2.565E-01	2.565E-01		
48	4.542E-02	1.439E-01	1.439E-01	8.948E-02	8.946E-02				1.827E-01	4.519E-01	4.519E-01	3.124E-01	3.123E-01			
49	1.369E-01	7.032E-02	5.867E-02	1.369E-01	6.494E-02	6.493E-02			4.244E-01	2.565E-01	2.195E-01	4.244E-01	2.385E-01	2.385E-01		
50	1.404E-01	6.567E-02	9.765E-02	6.567E-02					4.332E-01	2.145E-01	3.452E-01	2.145E-01				
51	1.491E-01	1.491E-01	6.337E-02	6.337E-02	6.337E-02	6.336E-02			4.601E-01	4.601E-01	2.392E-01	2.392E-01	2.392E-01	2.392E-01		
52	7.496E-02	1.415E-01	1.415E-01	6.282E-02	5.707E-02	6.282E-02			2.801E-01	4.468E-01	4.468E-01	2.338E-01	2.094E-01	2.338E-01		
53	1.094E-01	1.094E-01	2.031E-01						2.085E-01	2.085E-01	4.964E-01					
54	9.279E-02	9.279E-02	9.279E-02						2.162E-01	2.162E-01	2.162E-01					
55	5.764E-02	5.852E-02	6.340E-02	1.477E-01	5.764E-02	6.340E-02			2.103E-01	2.118E-01	2.357E-01	4.560E-01	2.104E-01	2.357E-01		
56	7.623E-02	2.727E-01	5.733E-02	7.624E-02	1.484E-01	5.732E-02			2.491E-01	7.036E-01	1.990E-01	2.491E-01	4.512E-01	1.990E-01		
57	2.825E-01	8.552E-02	1.378E-01	7.675E-02	6.084E-02	5.042E-02			7.037E-01	2.742E-01	4.234E-01	2.465E-01	2.244E-01	1.657E-01		
58	5.562E-02	5.562E-02	6.419E-02	6.419E-02	1.491E-01				1.962E-01	1.962E-01	2.315E-01	2.315E-01	4.600E-01			
59	6.629E-02	6.629E-02	5.442E-02	1.468E-01					2.240E-01	2.240E-01	1.798E-01	4.504E-01				
60	1.106E-01	6.451E-02	6.451E-02	5.263E-02					3.821E-01	2.196E-01	2.196E-01	1.734E-01				
61	5.248E-02	6.196E-02	5.419E-02	6.354E-02	1.608E-01	1.592E-01			1.820E-01	2.044E-01	1.813E-01	2.114E-01	4.751E-01	4.767E-01		
62	5.417E-02	5.417E-02	1.523E-01	1.523E-01					1.703E-01	1.703E-01	4.595E-01	4.595E-01				
63	5.807E-02	5.808E-02	5.808E-02	5.807E-02	5.807E-02	5.807E-02			2.102E-01	2.102E-01	2.102E-01	2.102E-01	2.102E-01	2.102E-01		
64	5.751E-02	5.798E-02	5.712E-02	5.751E-02	5.670E-02	5.712E-02	5.670E-02		2.073E-01	2.104E-01	2.058E-01	2.073E-01	2.043E-01	2.058E-01	2.043E-01	
65	3.555E-01	3.555E-01	6.470E-02	6.729E-02	6.731E-02	6.470E-02			6.975E-01	6.975E-01	2.007E-01	2.227E-01	2.227E-01	2.007E-01		
66	5.811E-02	5.811E-02	5.811E-02	5.811E-02	5.811E-02	5.811E-02			2.072E-01	2.072E-01	2.072E-01	2.072E-01	2.072E-01	2.072E-01		
67	5.652E-02	5.601E-02	5.731E-02	5.716E-02	5.651E-02	5.715E-02	5.576E-02	5.730E-02	2.029E-01	1.990E-01	2.063E-01	2.056E-01	2.029E-01	2.055E-01	2.004E-01	2.062E-01
68	5.587E-02	5.587E-02	5.587E-02	5.865E-02	5.587E-02	5.865E-02			2.003E-01	2.003E-01	2.003E-01	2.119E-01	2.003E-01	2.119E-01		
69	7.625E-02	7.626E-02	5.225E-02	5.225E-02	5.605E-02	2.697E-01			2.460E-01	2.460E-01	1.742E-01	1.742E-01	2.017E-01	6.968E-01		
70	5.805E-02	5.930E-02	5.848E-02	5.495E-02	5.545E-02				2.047E-01	2.097E-01	2.065E-01	1.915E-01	1.937E-01			
71	5.760E-02	5.760E-02	5.760E-02	5.760E-02					1.950E-01	1.950E-01	1.950E-01	1.950E-01				
72	5.806E-02	5.806E-02	5.806E-02	5.806E-02					1.949E-01	1.949E-01	1.949E-01	1.949E-01				
73	5.564E-02	4.736E-02	8.570E-02	6.545E-02	3.429E-01				1.805E-01	1.567E-01	2.759E-01	1.072E-01	6.386E-01			
	L								ESP							
#	BCP1	BCP2	BCP3	BCP4	BCP5	BCP6	BCP7	BCP8	BCP1	BCP2	BCP3	BCP4	BCP5	BCP6	BCP7	BCP8
1	2.625E-01	2.626E-01	2.625E-01	2.625E-01	2.625E-01	2.626E-01			8.628E-01	8.628E-01	8.628E-01	8.628E-01	8.628E-01	8.628E-01		
2	2.622E-01	2.622E-01	2.624E-01	2.622E-01	2.624E-01	2.622E-01			7.534E-01	7.534E-01	7.538E-01	7.534E-01	7.538E-01	7.534E-01		
3	2.615E-01	2.615E-01	2.615E-01	2.617E-01	2.615E-01	2.617E-01			6.741E-01	6.741E-01	6.742E-01	6.767E-01	6.741E-01	6.767E-01		
4	2.714E-01	2.668E-01	2.513E-01	2.535E-01	2.513E-01	2.668E-01			8.464E-01	8.769E-01	8.447E-01	8.460E-01	8.447E-01	8.769E-01		
5	2.597E-01	2.597E-01	2.597E-01	2.597E-01	2.597E-01	2.596E-01	2.596E-01	2.597E-01	1.257E+00	1.257E+00	1.257E+00	1.257E+00	1.257E+00	1.257E+00	1.257E+00	1.257E+00
6	2.463E-01	2.404E-01	2.742E-01	2.601E-01	2.742E-01	2.463E-01			8.380E-01	8.260E-01	8.898E-01	8.551E-01	8.898E-01	8.380E-01		
7	2.368E-01	2.763E-01	2.473E-01	2.531E-01	2.456E-01	2.731E-01			8.156E-01	8.940E-01	8.326E-01	8.433E-01	8.349E-01	8.895E-01		
8	2.499E-01	2.381E-01	2.421E-01	2.545E-01	2.413E-01	2.388E-01	2.502E-01		6.643E-01	6.191E-01	6.384E-01	6.776E-01	6.361E-01	6.190E-01	6.660E-01	
9	2.266E-01	2.266E-01	2.266E-01	2.266E-01	2.266E-01				5.930E-01	5.930E-01	5.930E-01	5.930E-01	5.930E-01			
10	2.678E-01	2.476E-01	2.678E-01	2.862E-01	2.476E-01	2.445E-01	2.862E-01		1.235E+00	1.196E+00	1.235E+00	1.295E+00	1.196E+00	1.208E+00	1.295E+00	
11	2.553E-01	2.157E-01	2.570E-01	2.153E-01	2.570E-01	2.153E-01	2.157E-01	2.553E-01	5.935E-01	5.000E-01	5.943E-01	4.998E-01	5.943E-01	4.998E-01	5.000E-01	5.935E-01
12	2.640E-01	2.460E-01	2.373E-01	2.794E-01	2.373E-01	2.794E-01			8.764E-01	8.107E-01	8.231E-01	9.226E-01	8.231E-01	9.226E-01		
13	2.793E-01	2.322E-01	2.421E-01	2.444E-01	2.421E-01	2.793E-01			9.019E-01	8.020E-01	8.205E-01	8.264E-01	8.205E-01	9.019E-01		
14	2.319E-01	2.284E-01	2.814E-01	2.459E-01	2.540E-01	2.637E-01			8.073E-01	8.006E-01	9.033E-01	8.276E-01	8.481E-01	8.769E-01		
15	2.719E-01	2.281E-01	2.392E-01	2.475E-01	2.392E-01	2.719E-01			7.054E-01	6.128E-01	6.357E-01	6.500E-01	6.357E-01	7.054E-01		
16	2.375E-01	2.400E-01	2.416E-01	2.822E-01	2.416E-01	2.400E-01			8.176E-01	8.192E-01	8.253E-01	9.124E-01	8.252E-01	8.192E-01		
17	2.223E-01	2.589E-01	2.589E-01	2.589E-01	2.223E-01	2.589E-01			7.829E-01	8.594E-01	8.594E-01	8.594E-01	7.829E-01	8.594E-01		
18	2.375E-01	2.375E-01	2.375E-01	2.375E-01	2.375E-01	2.375E-01			8.176E-01	8.176E-01	8.176E-01	8.176E-01	8.176E-01	8.176E-01		
19	2.334E-01	2.335E-01	2.335E-01	2.334E-01					1.407E+00	1.407E+00	1.407E+00	1.407E+00				
20	2.385E-01	2.391E-01	2.605E-01	2.909E-01	2.605E-01	2.385E-01			1.186E+00	1.189E+00	1.237E+00	1.278E+00	1.237E+00	1.186E+00		
21	2.260E-01	2.260E-01	2.244E-01	2.158E-01	2.158E-01				5.362E-01	5.362E-01	4.928E-01	5.184E-01	5.184E-01			
22	2.448E-01	2.636E-01	2.448E-01	2.385E-01	2.636E-01	1.951E-01	2.385E-01		6.159E-01	6.579E-01	6.159E-01	5.944E-01	6.579E-01	5.045E-01	5.944E-01	
23	2.272E-01	2.759E-01	2.227E-01	2.759E-01	2.612E-01	2.227E-01			5.563E-01	7.060E-01	5.781E-01	7.060E-01	6.560E-01	5.781E-01		
24	2.475E-01	2.864E-01	2.237E-01	2.864E-01	2.725E-01	2.237E-01			8.092E-01	9.434E-01	7.895E-01	9.434E-01	8.904E-01	7.895E-01		
25	2.357E-01	2.223E-01	2.847E-01	2.374E-01	2.847E-01	2.357E-01			8.076E-01	7.829E-01	9.149E-01	8.121E-01	9.149E-01	8.076E-01		
26	1.951E-01	2.402E-01	2.386E-01	2.402E-01	2.386E-01				5.045E-01	5.998E-01	5.994E-01	5.998E-01	5.994E-01			
27	2.558E-01	2.558E-01	2.301E-01	2.558E-01	2.558E-01	2.301E-01			1.383E+00	1.383E+00	1.250E+00	1.383E+00	1.383E+00	1.250E+00		
28	2.604E-01	2.611E-01	2.604E-01	2.697E-01	2.611E-01	1.798E-01	2.697E-01		8.721E-01	8.731E-01	8.721E-01	8.841E-01	8.730E-01	6.917E-01	8.841E-01	
29	2.368E-01	2.110E-01	2.313E-01	2.951E-01	2.313E-01	2.368E-01			8.156E-01	7.641E-01	7.969E-01	9.361E-01	7.969E-01	8.156E-01		
30	1.798E-01	2.485E-01	2.482E-01	2.484E-01	2.482E-01				6.917E-01	8.392E-01	8.368E-01	8.392E-01	8.368E-01			
31	1.963E-01	2.774E-01	2.672E-01	2.774E-01	2.199E-01	2.672E-01			1.135E+00	1.356E+00	1.276E+00	1.356E+00	1.177E+00	1.276E+00		
32	1.906E-01	2.135E-01	1.338E-01	1.906E-01					9.120E-01	9.566E-01	8.116E-01	9.120E-01				
33	2.055E-01	2.404E-01	2.404E-01	2.055E-01	2.055E-01	2.404E-01			7.511E-01	8.260E-01	8.260E-01	7.511E-01	7.511E-01	8.260E-01		
34	2.476E-01	2.048E-01	2.474E-01	2.917E-01	2.049E-01	1.825E-01	2.916E-01		6.729E-01	5.734E-01	6.726E-01	7.623E-01	5.737E-01	5.147E-01	7.621E-01	
35	2.319E-01	1.985E-01	2.319E-01	2.319E-01	1.985E-01	2.319E-01			8.073E-01	7.373E-01	8.073E-01	8.073E-01	7.373E-01	8.073E-01		
36	2.309E-01	2.427E-01	1.963E-01	2.427E-01					1.227E+00	1.262E+00	1.135E+00	1.262E+00				
37	4.260E-01	2.360E-01	2.347E-01	2.357E-01	2.348E-01	2.504E-01			1.200E+00	9.579E-01	8.560E-01	9.573E-01	8.560E-01	8.602E-01		
38	2.445E-01	1.927E-01	2.749E-01	1.927E-01	2.749E-01				1.208E+00	1.098E+00	1.288E+00	1.098E+00	1.288E+00			
39	2.028E-01	3.044E-01	2.028E-01	2.028E-01	3.044E-01	3.044E-01	3.044E-01	2.028E-01	7.327E-01	9.730E-01	7.327E-01	7.327E-01	9.730E-01	9.730E-01	9.730E-01	7.327E-01
40	1.781E-01	2.888E-01	2.134E-01	1.927E-01	2.874E-01				6.997E-01	9.373E-01	7.421E-01	7.319E-01	9.467E-01			
41	2.292E-01	2.292E-01	2.292E-01	2.292E-01	2.292E-01	2.292E-01			1.360E+00	1.360E+00	1.360E+00	1.360E+00	1.360E+00	1.360E+00		
42	2.586E-01	1.581E-01	2.272E-01	1.581E-01					6.725E-01	4.724E-01	5.563E-01	4.724E-01				

43	1.628E-01	2.883E-01	2.934E-01	2.035E-01	1.734E-01				6.509E-01	9.298E-01	9.029E-01	7.513E-01	6.796E-01			
44	3.540E-01	2.579E-01	3.223E-01	1.889E-01	2.061E-01	2.384E-01			1.203E+00	1.158E+00	1.430E+00	8.238E-01	9.022E-01	1.018E+00		
45	1.723E-01	2.963E-01	1.978E-01	1.723E-01	2.963E-01				6.705E-01	9.436E-01	7.276E-01	6.705E-01	9.436E-01			
46	2.460E-01	1.579E-01	1.579E-01	2.460E-01					8.107E-01	6.876E-01	6.876E-01	8.107E-01				
47	1.862E-01	1.862E-01	1.862E-01	1.862E-01	1.862E-01	1.862E-01			6.906E-01	6.906E-01	6.906E-01	6.906E-01	6.906E-01	6.906E-01		
48	1.373E-01	3.080E-01	3.081E-01	2.229E-01	2.229E-01				8.338E-01	1.233E+00	1.233E+00	1.066E+00	1.066E+00			
49	2.875E-01	1.862E-01	1.609E-01	2.875E-01	1.736E-01	1.736E-01			9.230E-01	6.906E-01	6.260E-01	9.229E-01	6.580E-01	6.580E-01		
50	2.928E-01	1.488E-01	2.475E-01	1.488E-01					9.603E-01	6.638E-01	8.092E-01	6.638E-01				
51	3.111E-01	3.111E-01	1.758E-01	1.758E-01	1.758E-01	1.758E-01			9.767E-01	9.767E-01	6.690E-01	6.690E-01	6.689E-01	6.689E-01		
52	2.052E-01	3.052E-01	3.052E-01	1.710E-01	1.523E-01	1.710E-01			7.342E-01	9.582E-01	9.582E-01	6.603E-01	6.156E-01	6.603E-01		
53	9.903E-02	9.905E-02	2.933E-01						7.365E-01	7.365E-01	1.102E+00					
54	1.234E-01	1.234E-01	1.234E-01						7.054E-01	7.054E-01	7.054E-01					
55	1.527E-01	1.533E-01	1.723E-01	3.083E-01	1.527E-01	1.723E-01			6.096E-01	6.102E-01	6.574E-01	9.662E-01	6.097E-01	6.574E-01		
56	1.729E-01	4.309E-01	1.417E-01	1.729E-01	3.028E-01	1.417E-01			7.749E-01	1.279E+00	6.366E-01	7.749E-01	9.875E-01	6.365E-01		
57	4.212E-01	1.886E-01	2.856E-01	1.698E-01	1.636E-01	1.153E-01			1.342E+00	8.152E-01	9.580E-01	7.790E-01	6.639E-01	5.783E-01		
58	1.406E-01	1.406E-01	1.673E-01	1.673E-01	3.108E-01				5.891E-01	5.891E-01	6.524E-01	6.524E-01	9.698E-01			
59	1.577E-01	1.577E-01	1.254E-01	3.036E-01					6.455E-01	6.455E-01	5.695E-01	9.591E-01				
60	2.714E-01	1.551E-01	1.551E-01	1.208E-01					8.464E-01	6.469E-01	6.469E-01	5.612E-01				
61	1.295E-01	1.424E-01	1.271E-01	1.478E-01	3.143E-01	3.175E-01			5.769E-01	6.420E-01	5.819E-01	6.530E-01	1.039E+00	1.035E+00		
62	1.162E-01	1.162E-01	3.073E-01	3.073E-01					5.795E-01	5.795E-01	9.860E-01	9.860E-01				
63	1.521E-01	1.521E-01	1.521E-01	1.521E-01	1.521E-01	1.521E-01			6.028E-01	6.028E-01	6.028E-01	6.028E-01	6.028E-01	6.028E-01		
64	1.498E-01	1.524E-01	1.487E-01	1.498E-01	1.477E-01	1.487E-01	1.477E-01		5.983E-01	6.027E-01	5.961E-01	5.983E-01	5.937E-01	5.961E-01	5.937E-01	
65	3.419E-01	3.420E-01	1.360E-01	1.554E-01	1.554E-01	1.360E-01			1.591E+00	1.591E+00	7.054E-01	7.692E-01	7.693E-01	7.054E-01		
66	1.491E-01	1.491E-01	1.491E-01	1.491E-01	1.491E-01	1.491E-01			5.956E-01	5.956E-01	5.956E-01	5.956E-01	5.956E-01	5.956E-01		
67	1.464E-01	1.430E-01	1.490E-01	1.484E-01	1.464E-01	1.484E-01	1.447E-01	1.489E-01	5.919E-01	5.866E-01	5.964E-01	5.954E-01	5.918E-01	5.953E-01	5.865E-01	5.963E-01
68	1.444E-01	1.444E-01	1.444E-01	1.533E-01	1.444E-01	1.533E-01			5.873E-01	5.873E-01	5.873E-01	6.062E-01	5.873E-01	6.062E-01		
69	1.697E-01	1.697E-01	1.220E-01	1.220E-01	1.457E-01	4.271E-01			7.652E-01	7.652E-01	5.783E-01	5.783E-01	6.089E-01	1.264E+00		
70	1.467E-01	1.504E-01	1.480E-01	1.365E-01	1.382E-01				5.963E-01	6.044E-01	5.992E-01	5.750E-01	5.785E-01			
71	1.374E-01	1.374E-01	1.374E-01	1.374E-01					5.851E-01	5.851E-01	5.851E-01	5.851E-01				
72	1.368E-01	1.368E-01	1.368E-01	1.368E-01					3.687E-01	3.687E-01	3.687E-01	3.687E-01				
73	1.249E-01	1.094E-01	1.902E-01	4.170E-02	2.957E-01				6.251E-01	5.553E-01	8.551E-01	5.534E-01	1.631E+00			
	ESPe								ESPn							
#	BCP1	BCP2	BCP3	BCP4	BCP5	BCP6	BCP7	BCP8	BCP1	BCP2	BCP3	BCP4	BCP5	BCP6	BCP7	BCP8
1	-1.560E+01	-1.560E+01	-1.560E+01	-1.560E+01	-1.560E+01	-1.560E+01			1.646E+01	1.646E+01	1.646E+01	1.646E+01	1.646E+01	1.646E+01		
2	-1.570E+01	-1.570E+01	-1.570E+01	-1.570E+01	-1.570E+01	-1.570E+01			1.646E+01	1.646E+01	1.646E+01	1.646E+01	1.646E+01	1.646E+01		
3	-1.578E+01	-1.578E+01	-1.578E+01	-1.577E+01	-1.578E+01	-1.577E+01			1.645E+01	1.645E+01	1.645E+01	1.645E+01	1.645E+01	1.645E+01		
4	-1.953E+01	-1.837E+01	-1.753E+01	-1.736E+01	-1.753E+01	-1.837E+01			2.038E+01	1.924E+01	1.837E+01	1.820E+01	1.837E+01	1.924E+01		
5	-1.662E+01	-1.662E+01	-1.662E+01	-1.662E+01	-1.662E+01	-1.662E+01	-1.662E+01	-1.662E+01	1.788E+01	1.788E+01	1.788E+01	1.788E+01	1.788E+01	1.788E+01	1.788E+01	1.788E+01
6	-2.629E+01	-2.788E+01	-2.403E+01	-2.316E+01	-2.403E+01	-2.629E+01			2.713E+01	2.871E+01	2.492E+01	2.401E+01	2.492E+01	2.713E+01		
7	-2.459E+01	-2.125E+01	-2.279E+01	-2.083E+01	-2.365E+01	-2.175E+01			2.540E+01	2.214E+01	2.363E+01	2.168E+01	2.448E+01	2.264E+01		
8	-1.652E+01	-1.641E+01	-1.645E+01	-1.655E+01	-1.645E+01	-1.641E+01	-1.652E+01		1.718E+01	1.703E+01	1.709E+01	1.723E+01	1.708E+01	1.703E+01	1.719E+01	
9	-1.462E+01	-1.462E+01	-1.462E+01	-1.462E+01	-1.462E+01				1.521E+01	1.521E+01	1.521E+01	1.521E+01	1.521E+01			
10	-1.813E+01	-1.844E+01	-1.813E+01	-1.970E+01	-1.844E+01	-2.065E+01	-1.970E+01		1.937E+01	1.964E+01	1.937E+01	2.099E+01	1.964E+01	2.185E+01	2.099E+01	
11	-1.737E+01	-1.718E+01	-1.738E+01	-1.718E+01	-1.738E+01	-1.718E+01	-1.718E+01	-1.737E+01	1.797E+01	1.768E+01	1.797E+01	1.768E+01	1.797E+01	1.768E+01	1.768E+01	1.797E+01
12	-1.950E+01	-2.284E+01	-1.975E+01	-2.127E+01	-1.975E+01	-2.127E+01			2.038E+01	2.365E+01	2.058E+01	2.219E+01	2.058E+01	2.219E+01		
13	-1.902E+01	-2.126E+01	-2.014E+01	-1.852E+01	-2.014E+01	-1.902E+01			1.992E+01	2.206E+01	2.096E+01	1.935E+01	2.096E+01	1.992E+01		
14	-2.952E+01	-2.862E+01	-2.481E+01	-2.617E+01	-2.529E+01	-2.777E+01			3.032E+01	2.942E+01	2.572E+01	2.700E+01	2.614E+01	2.865E+01		
15	-1.916E+01	-2.140E+01	-2.029E+01	-1.870E+01	-2.029E+01	-1.916E+01			1.986E+01	2.201E+01	2.093E+01	1.935E+01	2.093E+01	1.986E+01		
16	-3.285E+01	-2.872E+01	-3.158E+01	-2.753E+01	-3.158E+01	-2.872E+01			3.367E+01	2.954E+01	3.240E+01	2.845E+01	3.240E+01	2.954E+01		
17	-2.420E+01	-2.358E+01	-2.358E+01	-2.358E+01	-2.420E+01	-2.358E+01			2.499E+01	2.444E+01	2.444E+01	2.444E+01	2.499E+01	2.444E+01		
18	-3.285E+01	-3.285E+01	-3.285E+01	-3.285E+01	-3.285E+01	-3.285E+01			3.367E+01	3.367E+01	3.367E+01	3.367E+01	3.367E+01	3.367E+01		
19	-1.258E+01	-1.258E+01	-1.258E+01	-1.258E+01					1.399E+01	1.399E+01	1.399E+01	1.399E+01				
20	-1.831E+01	-2.084E+01	-1.977E+01	-1.820E+01	-1.977E+01	-1.831E+01			1.950E+01	2.203E+01	2.101E+01	1.948E+01	2.101E+01	1.950E+01		
21	-1.730E+01	-1.730E+01	-1.954E+01	-1.819E+01	-1.819E+01				1.784E+01	1.784E+01	2.003E+01	1.871E+01	1.871E+01			
22	-1.868E+01	-1.916E+01	-1.868E+01	-2.015E+01	-1.916E+01	-2.112E+01	-2.015E+01		1.930E+01	1.982E+01	1.930E+01	2.075E+01	1.982E+01	2.163E+01	2.075E+01	
23	-1.919E+01	-1.824E+01	-1.733E+01	-1.824E+01	-1.734E+01	-1.733E+01			1.974E+01	1.894E+01	1.791E+01	1.894E+01	1.799E+01	1.791E+01		
24	-1.906E+01	-1.808E+01	-1.714E+01	-1.808E+01	-1.719E+01	-1.714E+01			1.987E+01	1.902E+01	1.793E+01	1.902E+01	1.808E+01	1.793E+01		
25	-2.271E+01	-2.420E+01	-2.117E+01	-2.047E+01	-2.117E+01	-2.271E+01			2.352E+01	2.499E+01	2.208E+01	2.128E+01	2.208E+01	2.352E+01		
26	-2.112E+01	-2.014E+01	-1.885E+01	-2.014E+01	-1.885E+01				2.163E+01	2.074E+01	1.945E+01	2.074E+01	1.945E+01			
27	-1.507E+01	-1.507E+01	-1.495E+01	-1.507E+01	-1.507E+01	-1.495E+01			1.645E+01	1.645E+01	1.620E+01	1.645E+01	1.645E+01	1.620E+01		
28	-1.856E+01	-1.895E+01	-1.856E+01	-2.009E+01	-1.895E+01	-2.083E+01	-2.009E+01		1.943E+01	1.982E+01	1.943E+01	2.097E+01	1.982E+01	2.152E+01	2.097E+01	
29	-2.459E+01	-2.455E+01	-2.310E+01	-2.243E+01	-2.310E+01	-2.459E+01			2.540E+01	2.532E+01	2.389E+01	2.337E+01	2.389E+01	2.540E+01		
30	-2.083E+01	-1.994E+01	-1.870E+01	-1.994E+01	-1.870E+01				2.152E+01	2.078E+01	1.954E+01	2.078E+01	1.954E+01			
31	-1.838E+01	-1.747E+01	-1.681E+01	-1.747E+01	-1.649E+01	-1.681E+01			1.952E+01	1.882E+01	1.809E+01	1.882E+01	1.766E+01	1.809E+01		
32	-1.261E+01	-1.282E+01	-1.236E+01	-1.261E+01					1.352E+01	1.378E+01	1.318E+01	1.352E+01				
33	-2.749E+01	-2.788E+01	-2.788E+01	-2.749E+01	-2.749E+01	-2.788E+01			2.825E+01	2.871E+01	2.871E+01	2.825E+01	2.825E+01	2.871E+01		
34	-1.659E+01	-1.637E+01	-1.659E+01	-1.673E+01	-1.637E+01	-1.624E+01	-1.673E+01		1.726E+01	1.694E+01	1.726E+01	1.750E+01	1.695E+01	1.675E+01	1.750E+01	
35	-2.952E+01	-2.891E+01	-2.952E+01	-2.952E+01	-2.891E+01	-2.952E+01			3.032E+01	2.965E+01	3.032E+01	3.032E+01	2.965E+01	3.032E+01		
36	-1.634E+01	-1.721E+01	-1.838E+01	-1.721E+01					1.757E+01	1.847E+01	1.952E+01	1.847E+01				
37	-1.589E+01	-1.515E+01	-1.521E+01	-1.515E+01	-1.521E+01	-1.533E+01			1.709E+01	1.611E+01	1.607E+01	1.611E+01	1.607E+01	1.619E+01		
38	-2.065E+01	-1.921E+01	-1.821E+01	-1.921E+01	-1.821E+01				2.185E+01	2.031E+01	1.950E+01	2.031E+01	1.950E+01			
39	-1.721E+01	-1.765E+01	-1.721E+01	-1.721E+01	-1.765E+01	-1.765E+01	-1.765E+01	-1.721E+01	1.794E+01	1.863E+01	1.794E+01	1.794E+01	1.863E+01	1.863E+01	1.863E+01	1.794E+01
40	-1.683E+01	-1.725E+01	-1.919E+01	-1.774E+01	-1.823E+01				1.753E+01	1.819E+01	1.993E+01	1.848E+01	1.918E+01			
41	-1.506E+01	-1.506E+01	-1.506E+01	-1.506E+01	-1.506E+01	-1.506E+01			1.642E+01	1.642E+01	1.642E+01	1.642E+01	1.642E+01	1.642E+01		
42	-1.711E+01	-1.752E+01	-1.919E+01	-1.752E+01					1.778E+01	1.800E+01	1.974E+01	1.800E+01				
43	-1.706E+01	-1.757E+01	-1.982E+01	-1.818E+01	-1.803E+01				1.771E+01	1.850E+01	2.073E+01	1.893E+01	1.871E+01			
44	-1.538E+01	-1.511E+01	-1.545E+01	-1.483E+01	-1.483E+01	-1.495E+01			1.658E+01	1.627E+01	1.688E+01	1.565E+01	1.573E+01	1.597E+01		
45	-1.434E+01	-1.481E+01	-1.441E+01	-1.434E+01	-1.481E+01				1.501E+01	1.576E+01	1.513E+01	1.501E+01	1.576E+01			
46	-2.284E+01	-2.193E+01	-2.193E+01	-2.284E+01					2.365E+01	2.262E+01	2.262E+01	2.365E+01				
47	-3.361E+01	-3.361E+01	-3.361E+01	-3.361E+01	-3.361E+01	-3.361E+01			3.430E+01	3.430E+01	3.430E+01	3.430E+01	3.430E+01	3.430E+01		
48	-1.354E+01	-1.432E+01	-1.432E+01	-1.400E+01	-1.400E+01				1.437E+01	1.556E+01	1.556E+01	1.507E+01	1.507E+01			
49	-3.295E+01	-3.361E+01	-2.806E+01	-3.295E+01	-2.961E+01	-2.961E+01			3.387E+01	3.430E+01	2.868E+01	3.387E+01	3.027E+01	3.027E+01		
50	-1.700E+01	-1.727E+01	-1.906E+01	-1.727E+01					1.796E+01	1.794E+01	1.987E+01	1.794E+01				
51	-1.607E+01	-1.607E+01	-1.546E+01	-1.546E+01	-1.546E+01	-1.546E+01			1.704E+01	1.704E+01	1.613E+01	1.613E+01	1.613E+01	1.613E+01		
52	-1.562E+01	-1.606E+01	-1.606E+01	-1.553E+01	-1.550E+01	-1.553E+01			1.635E+01	1.702E+01	1.702E+01	1.619E+01	1.612E+01	1.619E+01		
53	-1.149E+01	-1.149E+01	-1.204E+01						1.223E+01	1.223E+01	1.314E+01					
54	-1.193E+01	-1.193E+01	-1.193E+01						1.264E+01	1.264E+01	1.264E+01					
55	-1.584E+01	-1.591E+01	-1.583E+01	-1.641E+01	-1.584E+01	-1.583E+01			1.645E+01	1.652E+01	1.649E+01	1.738E+01	1.645E+01	1.649E+01		
56	-1.506E+01	-1.628E+01	-1.501E+01	-1.506E+01	-1.581E+01	-1.501E+01			1.583E+01	1.756E+01	1.565E+01	1.583E+01	1.680E+01	1.565E+01		
57	-1.628E+01	-1.514E+01	-1.565E+01	-1.510E+01	-1.523E+01	-1.497E+01			1.762E+01	1.596E+01	1.661E+01	1.588E+01	1.589E+01	1.555E+01		
58	-1.452E+01	-1.452E+01	-1.460E+01	-1.460E+01	-1.518E+01				1.511E+01	1.511E+01	1.525E+01	1.525E+01	1.615E+01			
59	-1.314E+01	-1.314E+01	-1.303E+01	-1.370E+01					1.378E+01	1.378E+01	1.359E+01	1.465E+01				
60	-1.953E+01	-1.768E+01	-1.768E+01	-1.673E+01					2.038E+01	1.832E+01	1.832E+01	1.729E+01				
61	-1.543E+01	-1.541E+01	-1.549E+01	-1.551E+01	-1.616E+01	-1.619E+01			1.600E+01	1.605E+01	1.608E+01	1.616E+01	1.720E+01	1.723E+01		
62	-1.246E+01	-1.246E+01	-1.324E+01	-1.324E+01					1.304E+01	1.304E+01	1.422E+01	1.422E+01				
63	-1.620E+01	-1.620E+01	-1.620E+01	-1.620E+01	-1.620E+01	-1.620E+01			1.680E+01	1.680E+01	1.680E+01	1.680E+01	1.680E+01	1.680E+01		
64	-1.728E+01	-1.721E+01	-1.735E+01	-1.728E+01	-1.720E+01	-1.735E+01	-1.720E+01		1.788E+01	1.781E+01	1.794E+01	1.788E+01	1.780E+01	1.794E+01	1.780E+01	
65	-1.600E+01	-1.600E+01	-1.455E+01	-1.467E+01	-1.467E+01	-1.455E+01			1.759E+01	1.759E+01	1.526E+01	1.544E+01	1.544E+01	1.526E+01		
66	-2.170E+01	-2.170E+01	-2.170E+01	-2.170E+01	-2.170E+01	-2.170E+01			2.229E+01	2.229E+01	2.229E+01	2.229E+01	2.229E+01	2.229E+01		
67	-1.831E+01	-1.828E+01	-1.830E+01	-1.822E+01	-1.831E+01	-1.822E+01	-1.855E+01	-1.830E+01	1.890E+01	1.887E+01	1.889E+01	1.882E+01	1.890E+01	1.882E+01	1.914E+01	1.889E+01
68	-1.618E+01	-1.618E+01	-1.618E+01	-1.629E+01	-1.618E+01	-1.629E+01			1.676E+01	1.676E+01	1.676E+01	1.689E+01	1.676E+01	1.689E+01		
69	-1.540E+01	-1.540E+01	-1.534E+01	-1.534E+01	-1.559E+01	-1.662E+01			1.617E+01	1.617E+01	1.592E+01	1.592E+01	1.620E+01	1.789E+01		
70	-1.492E+01	-1.497E+01	-1.494E+01	-1.484E+01	-1.485E+01				1.552E+01	1.557E+01	1.553E+01	1.541E+01	1.543E+01			
71	-1.345E+01	-1.345E+01	-1.345E+01	-1.345E+01					1.404E+01	1.404E+01	1.404E+01	1.404E+01				
72	-1.368E+01	-1.368E+01	-1.368E+01	-1.368E+01					1.405E+01	1.405E+01	1.405E+01	1.405E+01				
73	-1.403E+01	-1.399E+01	-1.430E+01	-1.379E+01	-1.489E+01				1.465E+01	1.455E+01	1.516E+01	1.434E+01	1.652E+01			

Table S2b. The HOMA (AIM) parameters in BCPs were obtained for all considered structures (Figure 1) with different functionals: B3LYP, ωB97-XD, and TPSSh and the aug-cc-pVTZ basis set.

B3LYP										
#	H(Elip)	H(Rho)	H(DelSqRho)	H(V)	H(G)	H(K)	H(L)	H(ESP)	H(ESPe)	H(ESPn)
1	1.00E+00	1.00E+00	1.00E+00	1.00E+00	1.00E+00	1.00E+00	1.00E+00	1.00E+00	1.00E+00	1.00E+00
2	1.00E+00	1.00E+00	1.00E+00	1.00E+00	1.00E+00	1.00E+00	1.00E+00	5.00E-02	8.10E-01	1.00E+00
3	1.00E+00	1.00E+00	1.00E+00	1.00E+00	1.00E+00	1.00E+00	1.00E+00	-1.55E+00	5.40E-01	1.00E+00
4	1.00E+00	9.70E-01	9.70E-01	9.70E-01	9.70E-01	9.70E-01	9.70E-01	9.80E-01	-9.90E+01	-3.17E+02
5	-3.20E-01	9.80E-01	1.00E+00	9.40E-01	7.10E-01	9.70E-01	1.00E+00	-1.09E+01	-1.48E+01	-9.53E+01
6	9.50E-01	9.20E-01	9.10E-01	9.00E-01	8.90E-01	9.00E-01	9.10E-01	9.50E-01	-1.39E+03	-4.44E+03
7	9.00E-01	9.00E-01	8.90E-01	8.60E-01	8.20E-01	8.70E-01	8.90E-01	9.20E-01	-7.08E+02	-2.25E+03
8	9.40E-01	7.70E-01	7.20E-01	7.90E-01	8.70E-01	7.70E-01	7.20E-01	-6.48E+00	-1.14E+01	-1.62E+01
9	7.20E-01	7.20E-01	5.20E-01	8.00E-01	9.80E-01	7.20E-01	5.20E-01	-4.39E+00	-1.28E+01	-7.07E+01
10	3.10E-01	8.80E-01	8.70E-01	8.20E-01	6.80E-01	8.50E-01	8.70E-01	-9.73E+00	-1.83E+02	-7.13E+02
11	5.80E-01	6.50E-01	5.30E-01	7.40E-01	9.10E-01	6.80E-01	5.30E-01	-1.07E+01	-4.09E+01	-7.78E+01
12	8.70E-01	8.70E-01	8.50E-01	8.00E-01	7.30E-01	8.20E-01	8.50E-01	8.20E-01	-4.01E+02	-1.28E+03
13	7.60E-01	8.20E-01	8.20E-01	7.50E-01	6.50E-01	7.70E-01	8.20E-01	8.50E-01	-2.53E+02	-8.02E+02
14	8.40E-01	8.20E-01	8.00E-01	7.60E-01	7.00E-01	7.80E-01	8.00E-01	8.70E-01	-1.93E+03	-6.13E+03
15	7.70E-01	8.10E-01	8.00E-01	7.40E-01	6.40E-01	7.60E-01	8.00E-01	-1.72E+00	-2.71E+02	-7.94E+02
16	7.50E-01	8.10E-01	8.10E-01	7.30E-01	6.20E-01	7.60E-01	8.10E-01	8.60E-01	-3.12E+03	-9.92E+03
17	8.00E-01	8.00E-01	7.80E-01	7.30E-01	6.70E-01	7.50E-01	7.80E-01	8.50E-01	-9.71E+02	-3.08E+03
18	7.20E-01	7.70E-01	7.50E-01	6.60E-01	5.40E-01	6.90E-01	7.50E-01	8.50E-01	-4.30E+03	-1.36E+04
19	-8.60E-01	7.90E-01	7.00E-01	8.10E-01	9.10E-01	7.80E-01	7.00E-01	-2.08E+01	-1.32E+02	-2.86E+02
20	4.00E-02	7.90E-01	8.10E-01	6.80E-01	4.40E-01	7.40E-01	8.10E-01	-8.64E+00	-2.02E+02	-7.72E+02
21	9.10E-01	5.40E-01	3.30E-01	6.10E-01	8.40E-01	5.20E-01	3.30E-01	-8.14E+00	-9.94E+01	-2.43E+02
22	8.10E-01	6.60E-01	6.30E-01	6.20E-01	5.90E-01	6.30E-01	6.30E-01	-4.29E+00	-2.40E+02	-6.71E+02
23	5.90E-01	7.10E-01	6.70E-01	6.10E-01	4.80E-01	6.40E-01	6.70E-01	-3.42E+00	-8.53E+01	-2.26E+02
24	5.60E-01	6.80E-01	6.70E-01	5.30E-01	3.00E-01	5.90E-01	6.70E-01	6.40E-01	-7.51E+01	-2.43E+02
25	5.60E-01	6.60E-01	6.70E-01	5.40E-01	3.60E-01	5.90E-01	6.70E-01	7.40E-01	-6.28E+02	-1.99E+03
26	8.40E-01	6.10E-01	4.90E-01	6.10E-01	6.90E-01	5.70E-01	4.90E-01	-5.24E+00	-2.68E+02	-7.48E+02
27	-8.00E-01	7.50E-01	8.10E-01	5.50E-01	1.00E-02	6.60E-01	8.10E-01	-1.62E+01	-3.92E+00	-4.20E-01
28	6.70E-01	6.00E-01	5.90E-01	5.60E-01	5.10E-01	5.70E-01	5.90E-01	6.70E-01	-2.22E+02	-7.03E+02
29	5.20E-01	5.30E-01	5.20E-01	3.70E-01	1.80E-01	4.30E-01	5.20E-01	6.40E-01	-9.63E+02	-3.04E+03
30	5.40E-01	4.20E-01	3.80E-01	3.70E-01	3.20E-01	3.70E-01	3.80E-01	5.30E-01	-2.43E+02	-7.54E+02
31	-4.20E-01	5.90E-01	5.50E-01	3.90E-01	1.60E-01	4.50E-01	5.50E-01	-1.15E+01	-4.39E+01	-2.10E+02
32	-5.40E-01	-5.20E-01	-1.54E+00	4.00E-01	6.60E-01	-1.00E-01	-1.54E+00	6.70E-01	-1.30E+02	-4.07E+02
33	4.80E-01	2.60E-01	2.20E-01	8.00E-02	-1.10E-01	1.30E-01	2.20E-01	4.70E-01	-2.11E+03	-6.64E+03
34	-2.60E-01	7.00E-02	1.00E-01	-7.00E-02	-3.80E-01	0.00000	1.00E-01	-3.04E+00	-1.17E+01	-2.55E+01
35	4.00E-01	1.90E-01	1.60E-01	-1.00E-02	-2.20E-01	5.00E-02	1.60E-01	4.30E-01	-2.71E+03	-8.55E+03
36	-1.09E+00	4.40E-01	3.90E-01	1.80E-01	-1.20E-01	2.60E-01	3.90E-01	-8.92E+00	-4.80E+01	-2.16E+02
37	1.50E-01	-9.30E-01	-1.09E+00	-2.14E+00	-4.01E+00	-1.72E+00	-1.09E+00	-6.70E-01	-1.04E+00	-7.02E+00
38	-6.60E-01	1.40E-01	1.60E-01	-1.40E-01	-5.50E-01	-3.00E-02	1.60E-01	-7.93E+00	-1.89E+02	-7.15E+02
39	-1.69E+00	-3.00E-01	-1.50E-01	-8.20E-01	-1.86E+00	-5.70E-01	-1.50E-01	-1.10E-01	-4.83E+01	-1.58E+02
40	-4.70E-01	-3.50E-01	-3.70E-01	-6.20E-01	-1.05E+00	-5.20E-01	-3.70E-01	-1.30E-01	-8.16E+01	-2.53E+02
41	-9.00E-02	6.40E-01	9.10E-01	-4.41E+00	-2.88E+01	-9.60E-01	9.10E-01	-1.37E+01	-3.17E+00	6.10E-01
42	-4.80E-01	-1.05E+00	-1.31E+00	-8.60E-01	-5.00E-01	-1.00E+00	-1.31E+00	-7.27E+00	-8.00E+01	-1.95E+02
43	-1.04E+00	-9.30E-01	-9.00E-01	-1.08E+00	-1.40E+00	-1.01E+00	-9.00E-01	-4.60E-01	-1.04E+02	-3.23E+02
44	-1.04E+00	-1.26E+00	-1.14E+00	-3.78E+00	-9.70E+00	-2.61E+00	-1.14E+00	-3.76E+00	-3.69E+00	-1.19E+01
45	-1.21E+00	-9.20E-01	-9.20E-01	-1.22E+00	-1.71E+00	-1.10E+00	-9.20E-01	-6.00E-01	-1.59E+01	-6.34E+01
46	-6.00E-01	-1.12E+00	-1.29E+00	-9.70E-01	-6.30E-01	-1.08E+00	-1.29E+00	-2.60E-01	-6.65E+02	-2.06E+03
47	-3.50E-01	-1.38E+00	-1.42E+00	-1.64E+00	-1.92E+00	-1.56E+00	-1.42E+00	-1.28E+00	-4.68E+03	-1.46E+04
48	-7.30E-01	-1.05E+00	-8.80E-01	-1.34E+00	-2.06E+00	-1.17E+00	-8.80E-01	-4.31E+00	-3.51E+01	-8.93E+01
49	-1.90E+00	-1.34E+00	-1.26E+00	-1.64E+00	-2.24E+00	-1.49E+00	-1.26E+00	-1.18E+00	-3.55E+03	-1.12E+04
50	-1.52E+00	-1.71E+00	-1.78E+00	-1.60E+00	-1.54E+00	-1.65E+00	-1.78E+00	-7.40E-01	-6.91E+01	-2.07E+02
51	-2.42E+00	-1.60E+00	-1.45E+00	-2.08E+00	-3.03E+00	-1.84E+00	-1.45E+00	-1.21E+00	-2.90E-01	-7.92E+00
52	-1.98E+00	-1.59E+00	-1.50E+00	-1.91E+00	-2.53E+00	-1.76E+00	-1.50E+00	-1.22E+00	-1.10E-01	-6.14E+00
53	-1.92E+01	-3.96E+00	-6.50E+00	-3.11E+00	-6.01E+00	-3.70E+00	-6.50E+00	-1.28E+00	-2.22E+02	-7.18E+02
54	-1.36E+01	-4.64E+00	-6.82E+00	-2.30E+00	5.80E-01	-3.68E+00	-6.82E+00	-7.90E-01	-1.94E+02	-6.75E+02
55	-3.18E+00	-2.83E+00	-2.75E+00	-3.05E+00	-3.49E+00	-2.93E+00	-2.75E+00	-2.56E+00	-1.34E+00	-5.61E+00
56	-2.20E+00	-4.83E+00	-4.33E+00	-7.01E+00	-1.19E+01	-5.92E+00	-4.33E+00	-2.69E+00	-3.42E+00	-2.58E+01
57	-2.23E+00	-4.63E+00	-4.10E+00	-6.85E+00	-1.20E+01	-5.72E+00	-4.10E+00	-3.17E+00	-2.62E+00	-2.29E+01
58	-3.00E+00	-3.19E+00	-3.14E+00	-3.29E+00	-3.59E+00	-3.23E+00	-3.14E+00	-2.73E+00	-1.22E+01	-6.13E+01
59	-2.63E+00	-3.39E+00	-3.42E+00	-3.22E+00	-3.16E+00	-3.28E+00	-3.42E+00	-2.58E+00	-8.06E+01	-3.00E+02
60	-2.07E+00	-3.37E+00	-3.40E+00	-2.97E+00	-2.48E+00	-3.12E+00	-3.40E+00	-2.42E+00	-9.05E+01	-2.64E+02
61	-1.63E+00	-3.84E+00	-3.76E+00	-3.93E+00	-4.50E+00	-3.84E+00	-3.76E+00	-2.93E+00	-8.80E-01	-1.30E+01
62	-3.89E+00	-4.03E+00	-3.80E+00	-3.80E+00	-4.25E+00	-3.76E+00	-3.80E+00	-2.55E+00	-1.11E+02	-3.85E+02

63	-4.00E+00	-4.00E+00	-4.00E+00	-4.00E+00	-4.00E+00	-4.00E+00	-4.00E+00	-4.00E+00	-4.00E+00	-4.00E+00
64	-3.88E+00	-4.27E+00	-4.24E+00	-4.21E+00	-4.17E+00	-4.22E+00	-4.24E+00	-4.21E+00	-3.87E+01	-8.86E+01
65	-2.05E+00	-7.85E+00	-6.69E+00	-1.20E+01	-2.17E+01	-9.82E+00	-6.69E+00	-4.20E+00	-9.88E+00	-5.10E+01
66	-4.17E+00	-4.20E+00	-4.33E+00	-4.19E+00	-4.03E+00	-4.24E+00	-4.33E+00	-4.37E+00	-5.32E+02	-1.55E+03
67	-3.82E+00	-4.47E+00	-4.43E+00	-4.36E+00	-4.26E+00	-4.38E+00	-4.43E+00	-4.37E+00	-1.04E+02	-2.71E+02
68	-3.94E+00	-4.48E+00	-4.45E+00	-4.37E+00	-4.28E+00	-4.40E+00	-4.45E+00	-4.38E+00	-4.14E+00	-4.06E+00
69	-2.91E+00	-6.28E+00	-5.78E+00	-8.05E+00	-1.24E+01	-7.11E+00	-5.78E+00	-3.99E+00	-2.09E+00	-2.13E+01
70	-4.00E+00	-4.71E+00	-4.74E+00	-4.49E+00	-4.19E+00	-4.58E+00	-4.74E+00	-4.49E+00	-6.26E+00	-4.33E+01
71	-3.66E+00	-5.33E+00	-5.39E+00	-4.83E+00	-4.20E+00	-5.03E+00	-5.39E+00	-4.77E+00	-6.70E+01	-2.75E+02
72	-3.56E+00	-5.39E+00	-5.49E+00	-4.85E+00	-4.13E+00	-5.07E+00	-5.49E+00	-1.73E+01	-5.38E+01	-2.74E+02
73	-2.21E+00	-8.35E+00	-8.02E+00	-9.91E+00	-1.34E+01	-9.14E+00	-8.02E+00	-4.45E+00	-2.83E+01	-1.19E+02
**ωB97-XD**										
**#**	**H(Elip)**	**H(Rho)**	**H(DelSqRho)**	**H(V)**	**H(G)**	**H(K)**	**H(L)**	**H(ESP)**	**H(ESPe)**	**H(ESPn)**
1	1.00E+00	1.00E+00	1.00E+00	1.00E+00	1.00E+00	1.00E+00	1.00E+00	1.00E+00	1.00E+00	1.00E+00
2	1.00E+00	1.00E+00	1.00E+00	1.00E+00	1.00E+00	1.00E+00	1.00E+00	3.30E-01	8.70E-01	1.00E+00
3	1.00E+00	1.00E+00	1.00E+00	1.00E+00	1.00E+00	1.00E+00	1.00E+00	-1.61E+00	5.10E-01	1.00E+00
4	9.90E-01	9.70E-01	9.70E-01	9.70E-01	9.70E-01	9.70E-01	9.70E-01	9.80E-01	-9.61E+01	-3.05E+02
5	-3.00E-01	9.80E-01	1.00E+00	9.40E-01	7.10E-01	9.70E-01	1.00E+00	-1.07E+01	-1.48E+01	-9.38E+01
6	9.50E-01	9.40E-01	9.20E-01	9.10E-01	9.00E-01	9.20E-01	9.20E-01	9.60E-01	-1.35E+03	-4.27E+03
7	9.00E-01	9.10E-01	9.00E-01	8.70E-01	8.30E-01	8.80E-01	9.00E-01	9.30E-01	-6.88E+02	-2.17E+03
8	9.30E-01	7.70E-01	7.20E-01	8.00E-01	8.70E-01	7.70E-01	7.20E-01	-6.43E+00	-1.12E+01	-1.57E+01
9	6.90E-01	7.20E-01	5.20E-01	8.10E-01	9.80E-01	7.20E-01	5.20E-01	-4.32E+00	-1.23E+01	-6.76E+01
10	3.30E-01	8.90E-01	8.70E-01	8.20E-01	6.80E-01	8.50E-01	8.70E-01	-9.71E+00	-1.78E+02	-6.89E+02
11	5.40E-01	6.50E-01	5.20E-01	7.40E-01	9.10E-01	6.70E-01	5.20E-01	-1.06E+01	-3.96E+01	-7.44E+01
12	8.50E-01	8.70E-01	8.50E-01	8.00E-01	7.30E-01	8.20E-01	8.50E-01	8.20E-01	-3.89E+02	-1.23E+03
13	7.20E-01	8.10E-01	8.20E-01	7.30E-01	6.20E-01	7.60E-01	8.20E-01	8.50E-01	-2.46E+02	-7.72E+02
14	7.90E-01	8.20E-01	8.10E-01	7.50E-01	6.60E-01	7.70E-01	8.10E-01	8.60E-01	-1.87E+03	-5.90E+03
15	7.30E-01	8.00E-01	8.10E-01	7.20E-01	6.10E-01	7.50E-01	8.10E-01	-1.64E+00	-2.64E+02	-7.67E+02
16	7.10E-01	8.00E-01	8.10E-01	7.10E-01	5.80E-01	7.50E-01	8.10E-01	8.60E-01	-3.03E+03	-9.54E+03
17	8.20E-01	8.50E-01	8.40E-01	7.90E-01	7.40E-01	8.10E-01	8.40E-01	8.80E-01	-9.44E+02	-2.97E+03
18	6.20E-01	7.20E-01	7.20E-01	6.00E-01	4.40E-01	6.50E-01	7.20E-01	8.20E-01	-4.16E+03	-1.31E+04
19	-4.90E-01	8.00E-01	7.20E-01	8.40E-01	9.30E-01	8.00E-01	7.20E-01	-2.06E+01	-1.27E+02	-2.70E+02
20	-2.00E-02	7.40E-01	7.70E-01	6.30E-01	3.70E-01	6.90E-01	7.70E-01	-8.59E+00	-1.97E+02	-7.46E+02
21	7.80E-01	2.60E-01	-7.00E-02	4.00E-01	7.60E-01	2.50E-01	-7.00E-02	-1.34E+01	-9.56E+01	-2.14E+02
22	7.30E-01	6.10E-01	5.90E-01	5.50E-01	4.80E-01	5.70E-01	5.90E-01	-5.18E+00	-2.33E+02	-6.40E+02
23	5.20E-01	7.00E-01	6.80E-01	5.70E-01	3.70E-01	6.10E-01	6.80E-01	-2.48E+00	-8.33E+01	-2.25E+02
24	4.90E-01	6.70E-01	6.60E-01	5.10E-01	2.50E-01	5.70E-01	6.60E-01	6.20E-01	-7.30E+01	-2.34E+02
25	4.50E-01	6.20E-01	6.40E-01	4.70E-01	2.40E-01	5.30E-01	6.40E-01	7.00E-01	-6.09E+02	-1.91E+03
26	8.40E-01	6.60E-01	5.20E-01	6.80E-01	7.90E-01	6.30E-01	5.20E-01	-5.91E+00	-2.63E+02	-7.20E+02
27	-6.80E-01	7.10E-01	7.90E-01	4.80E-01	-1.40E-01	6.10E-01	7.90E-01	-1.62E+01	-3.60E+00	-4.10E-01
28	6.40E-01	6.20E-01	6.10E-01	5.70E-01	5.10E-01	5.90E-01	6.10E-01	6.80E-01	-2.15E+02	-6.78E+02
29	4.00E-01	4.80E-01	4.80E-01	3.00E-01	5.00E-02	3.70E-01	4.80E-01	6.00E-01	-9.35E+02	-2.93E+03
30	5.10E-01	4.50E-01	4.20E-01	3.90E-01	3.20E-01	4.00E-01	4.20E-01	5.50E-01	-2.36E+02	-7.28E+02
31	-4.30E-01	6.00E-01	5.80E-01	4.00E-01	1.30E-01	4.70E-01	5.80E-01	-1.16E+01	-4.29E+01	-2.05E+02
32	-4.30E-01	-6.60E-01	-1.63E+00	3.20E-01	6.10E-01	-1.80E-01	-1.63E+00	5.90E-01	-1.26E+02	-3.93E+02
33	3.50E-01	1.70E-01	1.70E-01	-2.00E-02	-2.60E-01	5.00E-02	1.70E-01	4.20E-01	-2.04E+03	-6.38E+03
34	-4.80E-01	-5.00E-02	0.00000	-1.80E-01	-5.30E-01	-1.10E-01	0.00000	-3.19E+00	-1.12E+01	-2.41E+01
35	2.40E-01	7.00E-02	6.00E-02	-1.50E-01	-4.10E-01	-7.00E-02	6.00E-02	3.50E-01	-2.62E+03	-8.19E+03
36	-1.05E+00	5.00E-01	4.80E-01	2.30E-01	-1.20E-01	3.20E-01	4.80E-01	-8.95E+00	-4.69E+01	-2.09E+02
37	1.40E-01	-9.80E-01	-1.12E+00	-2.19E+00	-4.06E+00	-1.76E+00	-1.12E+00	-6.90E-01	-1.01E+00	-6.84E+00
38	-8.00E-01	3.00E-02	8.00E-02	-2.70E-01	-7.50E-01	-1.40E-01	8.00E-02	-8.12E+00	-1.82E+02	-6.86E+02
39	-2.03E+00	-4.20E-01	-2.40E-01	-9.80E-01	-2.09E+00	-7.00E-01	-2.40E-01	-2.00E-01	-4.74E+01	-1.54E+02
40	-7.80E-01	-4.90E-01	-4.70E-01	-7.90E-01	-1.31E+00	-6.70E-01	-4.70E-01	-2.40E-01	-7.87E+01	-2.42E+02
41	3.60E-01	7.20E-01	7.60E-01	-4.88E+00	-3.27E+01	-9.80E-01	7.60E-01	-1.58E+01	-2.96E+00	8.80E-01
42	-1.14E+00	-1.45E+00	-1.59E+00	-1.28E+00	-1.08E+00	-1.38E+00	-1.59E+00	-6.39E+00	-7.71E+01	-1.91E+02
43	-1.23E+00	-1.02E+00	-9.70E-01	-1.19E+00	-1.56E+00	-1.10E+00	-9.70E-01	-5.00E-01	-1.01E+02	-3.11E+02
44	-9.00E-01	-6.60E-01	-1.00E-02	-5.61E+00	-2.31E+01	-2.65E+00	-1.00E-02	-1.00E+01	-3.10E+00	-1.25E+01
45	-1.38E+00	-9.80E-01	-9.50E-01	-1.27E+00	-1.80E+00	-1.15E+00	-9.50E-01	-6.30E-01	-1.54E+01	-6.12E+01
46	-7.20E-01	-1.25E+00	-1.36E+00	-1.07E+00	-7.60E-01	-1.17E+00	-1.36E+00	-3.20E-01	-6.45E+02	-1.98E+03
47	-5.60E-01	-1.53E+00	-1.53E+00	-1.81E+00	-2.15E+00	-1.71E+00	-1.53E+00	-1.39E+00	-4.52E+03	-1.40E+04
48	-7.80E-01	-1.02E+00	-8.20E-01	-1.34E+00	-2.11E+00	-1.14E+00	-8.20E-01	-4.42E+00	-3.36E+01	-8.40E+01
49	-2.13E+00	-1.45E+00	-1.34E+00	-1.77E+00	-2.43E+00	-1.61E+00	-1.34E+00	-1.26E+00	-3.44E+03	-1.07E+04
50	-1.76E+00	-1.79E+00	-1.82E+00	-1.68E+00	-1.68E+00	-1.72E+00	-1.82E+00	-7.90E-01	-6.70E+01	-1.99E+02
51	-2.60E+00	-1.66E+00	-1.47E+00	-2.13E+00	-3.08E+00	-1.88E+00	-1.47E+00	-1.24E+00	-2.50E-01	-7.70E+00
52	-2.17E+00	-1.67E+00	-1.55E+00	-2.00E+00	-2.65E+00	-1.83E+00	-1.55E+00	-1.27E+00	-8.00E-02	-6.02E+00
53	-1.82E+01	-4.08E+00	-6.66E+00	-3.18E+00	-5.83E+00	-3.79E+00	-6.66E+00	-1.27E+00	-2.16E+02	-6.94E+02
54	-1.42E+01	-4.61E+00	-6.86E+00	-2.22E+00	6.10E-01	-3.61E+00	-6.86E+00	-8.00E-01	-1.87E+02	-6.46E+02

55	-3.26E+00	-2.87E+00	-2.77E+00	-3.07E+00	-3.51E+00	-2.96E+00	-2.77E+00	-2.58E+00	-1.28E+00	-5.31E+00
56	*ND*	*ND*	*ND*	*ND*	*ND*	*ND*	*ND*	*ND*	*ND*	*ND*
57	-2.36E+00	-4.78E+00	-3.98E+00	-7.59E+00	-1.46E+01	-6.07E+00	-3.98E+00	-3.94E+00	-2.70E+00	-2.41E+01
58	-3.10E+00	-3.20E+00	-3.15E+00	-3.30E+00	-3.59E+00	-3.24E+00	-3.15E+00	-2.73E+00	-1.17E+01	-5.87E+01
59	-2.76E+00	-3.37E+00	-3.39E+00	-3.20E+00	-3.14E+00	-3.25E+00	-3.39E+00	-2.57E+00	-7.77E+01	-2.87E+02
60	-2.09E+00	-3.38E+00	-3.41E+00	-2.97E+00	-2.49E+00	-3.12E+00	-3.41E+00	-2.41E+00	-8.80E+01	-2.54E+02
61	-1.69E+00	-3.84E+00	-3.75E+00	-3.94E+00	-4.54E+00	-3.84E+00	-3.75E+00	-2.96E+00	-8.00E-01	-1.24E+01
62	-4.20E+00	-3.93E+00	-3.70E+00	-3.72E+00	-4.18E+00	-3.67E+00	-3.70E+00	-2.49E+00	-1.06E+02	-3.67E+02
63	-4.00E+00	-4.00E+00	-4.00E+00	-4.00E+00	-4.00E+00	-4.00E+00	-4.00E+00	-4.00E+00	-4.00E+00	-4.00E+00
64	-3.86E+00	-4.27E+00	-4.24E+00	-4.21E+00	-4.18E+00	-4.22E+00	-4.24E+00	-4.22E+00	-3.82E+01	-8.65E+01
65	*ND*	*ND*	*ND*	*ND*	*ND*	*ND*	*ND*	*ND*	*ND*	*ND*
66	-4.11E+00	-4.21E+00	-4.34E+00	-4.21E+00	-4.06E+00	-4.25E+00	-4.34E+00	-4.38E+00	-5.17E+02	-1.50E+03
67	-3.79E+00	-4.47E+00	-4.43E+00	-4.36E+00	-4.27E+00	-4.38E+00	-4.43E+00	-4.37E+00	-1.02E+02	-2.64E+02
68	-3.92E+00	-4.48E+00	-4.45E+00	-4.37E+00	-4.27E+00	-4.40E+00	-4.45E+00	-4.37E+00	-4.18E+00	-4.14E+00
69	*ND*	*ND*	*ND*	*ND*	*ND*	*ND*	*ND*	*ND*	*ND*	*ND*
70	-3.97E+00	-4.68E+00	-4.73E+00	-4.46E+00	-4.15E+00	-4.55E+00	-4.73E+00	-4.46E+00	-5.80E+00	-4.07E+01
71	-3.69E+00	-5.31E+00	-5.39E+00	-4.79E+00	-4.14E+00	-5.00E+00	-5.39E+00	-4.75E+00	-6.42E+01	-2.62E+02
72	-3.59E+00	-5.30E+00	-5.41E+00	-4.74E+00	-4.02E+00	-4.97E+00	-5.41E+00	-1.72E+01	-5.08E+01	-2.59E+02
73	-3.65E+00	-7.85E+00	-6.85E+00	-1.06E+01	-2.60E+01	-8.26E+00	-6.85E+00	-1.08E+01	-2.82E+01	-1.15E+02

TPSSh

#	H(Elip)	H(Rho)	H(DelSqRho)	H(V)	H(G)	H(K)	H(L)	H(ESP)	H(ESPe)	H(ESPn)
1	1.00E+00	1.00E+00	1.00E+00	1.00E+00	1.00E+00	1.00E+00	1.00E+00	1.00E+00	1.00E+00	1.00E+00
2	1.00E+00	1.00E+00	1.00E+00	1.00E+00	1.00E+00	1.00E+00	1.00E+00	1.20E-01	8.40E-01	1.00E+00
3	1.00E+00	1.00E+00	1.00E+00	1.00E+00	1.00E+00	1.00E+00	1.00E+00	-1.61E+00	5.60E-01	1.00E+00
4	1.00E+00	9.70E-01	9.70E-01	9.80E-01	9.80E-01	9.70E-01	9.70E-01	9.80E-01	-9.47E+01	-2.96E+02
5	-1.80E-01	9.70E-01	1.00E+00	9.10E-01	6.70E-01	9.50E-01	1.00E+00	-1.05E+01	-1.35E+01	-8.56E+01
6	9.50E-01	9.30E-01	9.10E-01	9.00E-01	8.90E-01	9.10E-01	9.10E-01	9.50E-01	-1.34E+03	-4.16E+03
7	9.00E-01	9.00E-01	8.90E-01	8.70E-01	8.30E-01	8.80E-01	8.90E-01	9.30E-01	-6.81E+02	-2.11E+03
8	6.60E-01	8.40E-01	8.60E-01	8.10E-01	6.90E-01	8.30E-01	8.60E-01	-2.52E+00	-9.70E+00	-1.80E+01
9	7.60E-01	7.00E-01	4.70E-01	8.00E-01	9.80E-01	7.00E-01	4.70E-01	-4.38E+00	-1.23E+01	-6.62E+01
10	3.70E-01	8.90E-01	8.90E-01	8.30E-01	6.80E-01	8.60E-01	8.90E-01	-9.48E+00	-1.74E+02	-6.61E+02
11	-1.18E+01	-2.27E+01	-6.30E+00	-6.28E+00	-7.80E+00	-6.14E+00	-6.30E+00	-2.60E+01	-3.25E+02	-1.24E+03
12	8.90E-01	8.80E-01	8.60E-01	8.20E-01	7.70E-01	8.30E-01	8.60E-01	8.40E-01	-3.84E+02	-1.20E+03
13	7.70E-01	8.20E-01	8.20E-01	7.50E-01	6.60E-01	7.80E-01	8.20E-01	8.60E-01	-2.43E+02	-7.50E+02
14	8.40E-01	8.20E-01	8.10E-01	7.70E-01	7.20E-01	7.80E-01	8.10E-01	8.70E-01	-1.85E+03	-5.75E+03
15	8.20E-01	8.30E-01	8.20E-01	7.70E-01	7.10E-01	7.90E-01	8.20E-01	-2.21E+00	-2.60E+02	-7.35E+02
16	7.50E-01	8.10E-01	8.00E-01	7.30E-01	6.30E-01	7.50E-01	8.00E-01	8.60E-01	-3.00E+03	-9.29E+03
17	7.90E-01	7.90E-01	7.80E-01	7.30E-01	6.60E-01	7.40E-01	7.80E-01	8.40E-01	-9.32E+02	-2.88E+03
18	7.10E-01	7.60E-01	7.40E-01	6.60E-01	5.60E-01	6.90E-01	7.40E-01	8.50E-01	-4.13E+03	-1.28E+04
19	-7.30E-01	7.80E-01	6.50E-01	8.20E-01	9.40E-01	7.70E-01	6.50E-01	-2.09E+01	-1.25E+02	-2.63E+02
20	1.30E-01	8.00E-01	8.30E-01	6.90E-01	4.50E-01	7.50E-01	8.30E-01	-8.48E+00	-1.93E+02	-7.20E+02
21	9.30E-01	5.40E-01	3.00E-01	6.20E-01	8.60E-01	5.20E-01	3.00E-01	-7.69E+00	-9.57E+01	-2.30E+02
22	8.10E-01	6.60E-01	6.30E-01	6.30E-01	6.10E-01	6.30E-01	6.30E-01	-4.05E+00	-2.30E+02	-6.29E+02
23	6.40E-01	7.20E-01	6.70E-01	6.30E-01	5.10E-01	6.50E-01	6.70E-01	-3.29E+00	-8.19E+01	-2.12E+02
24	6.10E-01	7.10E-01	6.90E-01	5.70E-01	3.70E-01	6.20E-01	6.90E-01	6.60E-01	-7.21E+01	-2.27E+02
25	5.90E-01	6.70E-01	6.80E-01	5.60E-01	3.90E-01	6.00E-01	6.80E-01	7.50E-01	-6.03E+02	-1.86E+03
26	8.30E-01	5.80E-01	4.50E-01	5.80E-01	6.80E-01	5.40E-01	4.50E-01	-5.00E+00	-2.57E+02	-7.00E+02
27	-5.90E-01	7.80E-01	8.40E-01	6.10E-01	1.10E-01	7.10E-01	8.40E-01	-1.61E+01	-3.53E+00	3.00E-02
28	6.80E-01	6.00E-01	5.90E-01	5.60E-01	5.20E-01	5.70E-01	5.90E-01	6.80E-01	-2.12E+02	-6.56E+02
29	5.40E-01	5.40E-01	5.20E-01	3.90E-01	2.10E-01	4.40E-01	5.20E-01	6.50E-01	-9.27E+02	-2.85E+03
30	5.60E-01	4.20E-01	3.70E-01	3.70E-01	3.30E-01	3.70E-01	3.70E-01	5.30E-01	-2.33E+02	-7.06E+02
31	-3.20E-01	5.80E-01	5.40E-01	3.90E-01	1.60E-01	4.50E-01	5.40E-01	-1.14E+01	-4.18E+01	-1.95E+02
32	-7.10E-01	-7.20E-01	-2.01E+00	3.80E-01	6.50E-01	-2.20E-01	-2.01E+00	7.00E-01	-1.24E+02	-3.80E+02
33	4.90E-01	2.60E-01	2.30E-01	1.00E-01	-7.00E-02	1.50E-01	2.30E-01	4.90E-01	-2.03E+03	-6.24E+03
34	-2.10E-01	8.00E-02	1.10E-01	-4.00E-02	-3.20E-01	3.00E-02	1.10E-01	-3.03E+00	-1.12E+01	-2.36E+01
35	4.20E-01	2.10E-01	1.80E-01	4.00E-02	-1.50E-01	9.00E-02	1.80E-01	4.60E-01	-2.61E+03	-8.04E+03
36	-1.00E+00	4.30E-01	3.70E-01	1.50E-01	-1.30E-01	2.30E-01	3.70E-01	-8.72E+00	-4.60E+01	-2.01E+02
37	1.50E-01	-8.70E-01	-1.04E+00	-1.99E+00	-3.68E+00	-1.60E+00	-1.04E+00	-6.30E-01	-9.70E-01	-6.37E+00
38	-5.40E-01	1.30E-01	1.50E-01	-1.40E-01	-5.30E-01	-4.00E-02	1.50E-01	-7.75E+00	-1.80E+02	-6.66E+02
39	-1.55E+00	-2.50E-01	-9.00E-02	-7.50E-01	-1.71E+00	-5.00E-01	-9.00E-02	-8.00E-02	-4.65E+01	-1.48E+02
40	-3.20E-01	-2.70E-01	-2.90E-01	-4.90E-01	-8.50E-01	-4.10E-01	-2.90E-01	-5.00E-02	-7.88E+01	-2.38E+02
41	3.10E-01	8.30E-01	5.50E-01	-4.90E+00	-3.44E+01	-8.30E-01	5.50E-01	-1.73E+01	-3.00E+00	9.30E-01
42	-4.70E-01	-1.07E+00	-1.36E+00	-8.60E-01	-4.70E-01	-1.02E+00	-1.36E+00	-7.04E+00	-7.74E+01	-1.85E+02
43	-9.90E-01	-9.00E-01	-8.90E-01	-1.03E+00	-1.30E+00	-9.70E-01	-8.90E-01	-4.40E-01	-1.00E+02	-3.02E+02
44	-1.50E+00	-7.40E-01	-4.50E-01	-3.85E+00	-1.30E+01	-2.18E+00	-4.50E-01	-5.80E+00	-3.43E+00	-1.10E+01
45	-1.14E+00	-8.70E-01	-8.70E-01	-1.14E+00	-1.60E+00	-1.04E+00	-8.70E-01	-5.60E-01	-1.51E+01	-5.90E+01
46	-5.70E-01	-1.11E+00	-1.30E+00	-9.40E-01	-5.60E-01	-1.06E+00	-1.30E+00	-2.40E-01	-6.42E+02	-1.94E+03

47	-3.10E-01	-1.34E+00	-1.39E+00	-1.58E+00	-1.80E+00	-1.51E+00	-1.39E+00	-1.19E+00	-4.50E+03	-1.37E+04
48	-7.10E-01	-1.06E+00	-8.80E-01	-1.34E+00	-2.01E+00	-1.17E+00	-8.80E-01	-4.28E+00	-3.39E+01	-8.43E+01
49	-1.78E+00	-1.35E+00	-1.27E+00	-1.62E+00	-2.15E+00	-1.48E+00	-1.27E+00	-1.18E+00	-3.41E+03	-1.05E+04
50	-1.45E+00	-1.66E+00	-1.77E+00	-1.53E+00	-1.42E+00	-1.60E+00	-1.77E+00	-6.90E-01	-6.70E+01	-1.96E+02
51	-2.34E+00	-1.55E+00	-1.38E+00	-2.01E+00	-2.91E+00	-1.77E+00	-1.38E+00	-1.17E+00	-2.00E-01	-7.07E+00
52	-1.89E+00	-1.55E+00	-1.45E+00	-1.86E+00	-2.44E+00	-1.71E+00	-1.45E+00	-1.19E+00	-2.00E-02	-5.41E+00
53	-1.36E+01	-3.77E+00	-6.44E+00	-2.86E+00	-5.56E+00	-3.47E+00	-6.44E+00	-1.20E+00	-2.13E+02	-6.73E+02
54	-1.07E+01	-4.59E+00	-6.94E+00	-2.23E+00	5.90E-01	-3.63E+00	-6.94E+00	-8.30E-01	-1.85E+02	-6.30E+02
55	-3.12E+00	-2.81E+00	-2.72E+00	-3.01E+00	-3.43E+00	-2.90E+00	-2.72E+00	-2.54E+00	-1.28E+00	-5.08E+00
56	-2.18E+00	-4.70E+00	-4.14E+00	-6.87E+00	-1.17E+01	-5.76E+00	-4.14E+00	-2.78E+00	-3.15E+00	-2.38E+01
57	-2.19E+00	-4.50E+00	-3.87E+00	-6.89E+00	-1.26E+01	-5.63E+00	-3.87E+00	-3.54E+00	-2.37E+00	-2.15E+01
58	-2.92E+00	-3.15E+00	-3.12E+00	-3.25E+00	-3.50E+00	-3.19E+00	-3.12E+00	-2.70E+00	-1.15E+01	-5.66E+01
59	-2.57E+00	-3.28E+00	-3.35E+00	-3.12E+00	-3.02E+00	-3.18E+00	-3.35E+00	-2.51E+00	-7.65E+01	-2.78E+02
60	-2.03E+00	-3.36E+00	-3.43E+00	-2.95E+00	-2.44E+00	-3.12E+00	-3.43E+00	-2.41E+00	-8.72E+01	-2.47E+02
61	-1.61E+00	-3.81E+00	-3.74E+00	-3.86E+00	-4.37E+00	-3.78E+00	-3.74E+00	-2.87E+00	-7.50E-01	-1.16E+01
62	-3.85E+00	-3.99E+00	-3.80E+00	-3.73E+00	-4.11E+00	-3.71E+00	-3.80E+00	-2.53E+00	-1.06E+02	-3.59E+02
63	-4.00E+00	-4.00E+00	-4.00E+00	-4.00E+00	-4.00E+00	-4.00E+00	-4.00E+00	-4.00E+00	-4.00E+00	-4.00E+00
64	-3.89E+00	-4.29E+00	-4.26E+00	-4.22E+00	-4.17E+00	-4.24E+00	-4.26E+00	-4.23E+00	-3.78E+01	-8.44E+01
65	-1.53E+00	-6.11E+00	-3.62E+00	-1.49E+01	-4.50E+01	-9.38E+00	-3.62E+00	-1.29E+01	-8.82E+00	-5.34E+01
66	-4.13E+00	-4.16E+00	-4.28E+00	-4.14E+00	-3.99E+00	-4.19E+00	-4.28E+00	-4.28E+00	-5.16E+02	-1.47E+03
67	-3.85E+00	-4.51E+00	-4.49E+00	-4.39E+00	-4.28E+00	-4.42E+00	-4.49E+00	-4.40E+00	-1.01E+02	-2.57E+02
68	-3.96E+00	-4.47E+00	-4.45E+00	-4.36E+00	-4.27E+00	-4.39E+00	-4.45E+00	-4.37E+00	-4.28E+00	-4.32E+00
69	-2.92E+00	-6.20E+00	-5.66E+00	-7.89E+00	-1.20E+01	-6.96E+00	-5.66E+00	-4.01E+00	-1.90E+00	-1.96E+01
70	-3.95E+00	-4.71E+00	-4.78E+00	-4.49E+00	-4.18E+00	-4.59E+00	-4.78E+00	-4.49E+00	-5.74E+00	-3.96E+01
71	-3.58E+00	-5.28E+00	-5.42E+00	-4.78E+00	-4.10E+00	-5.00E+00	-5.42E+00	-4.70E+00	-6.27E+01	-2.52E+02
72	-3.49E+00	-5.31E+00	-5.48E+00	-4.76E+00	-4.00E+00	-5.01E+00	-5.48E+00	-1.71E+01	-5.00E+01	-2.50E+02
73	-3.53E+00	-7.83E+00	-7.00E+00	-1.04E+01	-2.61E+01	-8.12E+00	-7.00E+00	-1.14E+01	-2.81E+01	-1.12E+02

ND – Not determined

Table S3. The HOMA(AIM) parameters in RCPs were obtained for all considered structures (Figure 1) with B3LYP functional.

#	H(Rcp(A))	H(Rho_Nuc(A))	H(ESP_Nuc(A))	H(Vnn(A,Mol)/2)	H(q(A))	H(L(A))	H (K(A))	H (K_Scaled(A))	H (Mu_Intra(A))
1	1.00E+00	1.00E+00	1.00E+00	1.00E+00	1.00E+00	-1.66E+00	1.00E+00	1.00E+00	1.00E+00
2	-3.45E+00	9.89E-01	-4.82E+02	1.00E+00	-5.82E-01	1.98E-01	9.83E-01	9.93E-01	-1.49E+00
3	-2.16E+00	1.00E+00	-1.27E+03	ND	*ND*	*ND*	*ND*	*ND*	*ND*
4	-4.52E+00	6.79E-01	1.90E-01	-4.69E+01	8.04E-01	-4.36E-01	-1.69E+00	-3.20E+00	6.53E-01
5	-5.58E+01	-2.50E+00	-6.83E+03	-7.98E+01	-5.76E+00	-2.93E+00	-1.01E-01	-1.56E+00	7.05E-01
6	-3.97E+01	5.90E-01	-1.84E+00	-6.58E+02	9.66E-01	-1.62E+00	7.62E-01	9.52E-01	-1.03E+00
7	-4.51E+01	7.26E-01	-1.05E+00	-3.39E+02	9.62E-01	-1.01E+01	7.14E-01	8.25E-01	-8.16E-01
8	-4.00E+01	1.14E-01	-2.84E+03	*ND*	*ND*	*ND*	*ND*	*ND*	*ND*
9	-8.86E+01	-6.25E-01	-1.92E+03	-8.70E+00	-8.35E+00	4.19E-01	2.97E-01	2.21E-01	-5.74E+00
10	-1.14E+02	-1.29E+00	-5.74E+03	-1.18E+02	-4.26E+00	1.61E-01	8.20E-02	-8.76E-01	2.60E-01
11	-1.07E+02	-3.05E-01	-4.59E+03	-1.42E+01	-6.72E+00	-4.03E+00	-2.93E+00	-5.35E+00	-1.25E-01
12	-8.46E+01	2.17E-01	-1.46E+00	-1.90E+02	7.08E-01	-2.04E-01	-1.51E+00	-2.10E+00	-3.16E-01
13	-6.23E+01	8.19E-01	-3.38E-01	-1.24E+02	9.60E-01	2.96E-01	6.24E-01	6.34E-01	-6.01E-01
14	-7.31E+01	5.41E-01	-2.56E+00	-9.16E+02	9.39E-01	-8.46E-01	4.64E-01	7.38E-01	-1.79E+00
15	-7.36E+01	8.20E-01	-1.01E+03	*ND*	*ND*	*ND*	*ND*	*ND*	*ND*
16	-8.39E+01	3.47E-01	-2.61E+00	-1.48E+03	9.08E-01	5.53E-01	-4.38E-01	-8.10E-02	-2.64E+00
17	-2.41E+01	4.30E-01	-2.36E+00	-4.65E+02	9.11E-01	5.49E-01	1.69E-01	3.63E-01	-2.50E+00
18	-1.61E+02	1.52E-01	-4.18E+00	-2.04E+03	9.41E-01	9.18E-01	-1.94E+00	-1.54E+00	-6.25E+00
19	-1.81E+02	-1.29E+01	-1.35E+04	-4.19E+01	-1.84E+01	5.77E-01	-3.84E+00	-7.34E+00	-4.21E+01
20	-1.43E+02	-2.61E+00	-5.85E+03	-1.21E+02	-4.04E+00	6.37E-01	6.51E-01	1.74E-01	-2.75E+00
21	-5.28E+01	4.41E-01	-3.12E+03	*ND*	*ND*	*ND*	*ND*	*ND*	*ND*
22	-2.00E+01	8.88E-01	-1.88E+03	*ND*	*ND*	*ND*	*ND*	*ND*	*ND*
23	-5.97E+01	6.49E-01	-1.75E+03	*ND*	*ND*	*ND*	*ND*	*ND*	*ND*
24	-1.46E+02	5.57E-01	4.09E-01	-3.58E+01	4.61E-01	-8.70E-02	-1.87E+00	-3.49E+00	-1.38E+00
25	-2.74E+01	7.13E-01	-7.14E-01	-3.04E+02	9.56E-01	1.94E-01	6.11E-01	6.86E-01	-7.41E-01
26	-4.11E+01	9.92E-01	-2.09E+03	*ND*	*ND*	*ND*	*ND*	*ND*	*ND*
27	-5.61E+02	-5.28E+00	-9.11E+03	9.16E-01	-8.70E+00	-6.42E-01	-9.46E-01	-2.76E+00	-1.03E+01
28	-5.39E+00	2.92E-01	-5.81E+00	-1.11E+02	9.17E-01	-1.65E+00	5.23E-01	3.09E-01	-1.60E+00
29	-1.07E+02	5.90E-01	-3.09E+00	-4.67E+02	9.31E-01	-9.78E+00	4.52E-01	6.32E-01	-2.64E+00
30	-5.73E+00	7.24E-01	-3.72E+00	-1.21E+02	7.49E-01	-1.20E+00	4.24E-01	4.04E-01	-2.68E+00
31	-4.31E+02	-2.71E+00	-7.16E+03	-3.47E+01	-7.49E+00	-1.83E+00	-2.92E+00	-5.75E+00	-1.04E+01
32	-4.74E+01	-5.39E+01	-1.33E+02	-1.37E+02	-8.25E-01	-5.24E+00	-7.05E+00	-1.18E+01	-3.11E+02
33	-1.13E+02	5.59E-01	-7.52E+00	-1.02E+03	9.29E-01	-4.41E+00	7.48E-01	9.52E-01	-4.85E+00
34	-1.50E+02	-9.93E-01	-1.30E+03	-3.54E+00	-2.62E+00	-7.78E+00	-5.38E-01	-1.24E+00	-1.75E+01
35	-9.47E+01	5.22E-01	-7.53E+00	-1.31E+03	9.42E-01	3.30E-01	4.91E-01	7.84E-01	-4.64E+00
36	-1.75E+02	-9.27E+00	-8.20E+03	-3.98E+01	-6.43E+00	7.55E-01	-3.36E+00	-5.96E+00	-1.33E+01
37	-6.73E+02	8.70E-02	-2.01E+01	2.56E-01	-5.90E+00	3.88E-01	-6.03E+00	-1.04E+01	-3.19E+01
38	-4.21E+02	-5.39E+00	-6.15E+03	-1.17E+02	-3.82E+00	5.10E-01	1.34E-01	-3.78E-01	-5.74E+00
39	-1.72E+02	9.23E-01	8.21E-01	-2.28E+01	9.96E-01	-9.72E+00	9.30E-01	8.41E-01	2.43E-01
40	-1.48E+02	5.21E-01	-2.31E+00	-4.17E+01	8.75E-01	6.26E-01	-1.35E+00	-2.40E+00	-1.20E+00
41	-4.12E+03	-5.30E+00	-3.11E+02	-2.10E+02	-6.15E+01	1.04E-01	-6.59E+01	-1.08E+02	-2.47E+02
42	-7.73E+00	7.40E-01	-1.67E+03	*ND*	*ND*	*ND*	*ND*	*ND*	*ND*
43	-5.12E+01	1.81E-01	-3.34E+00	-2.06E+02	-1.00E-02	-3.55E+00	-2.64E+00	-4.64E+00	-2.68E-01
44	-1.71E+03	5.91E-01	-7.58E+01	-1.19E+02	-9.29E+00	-3.91E-01	-1.11E+01	-1.91E+01	-1.03E+02
45	-9.17E+01	-3.50E-02	-1.37E+00	-5.96E+00	2.13E-01	-1.95E+00	5.62E-01	3.28E-01	-6.19E-01
46	-5.01E+01	-1.29E+00	-4.19E+00	-3.31E+02	5.24E-01	9.44E-01	-4.33E+00	-5.61E+00	-1.71E+00
47	-1.17E+02	8.46E-01	-5.17E+00	-2.26E+03	9.56E-01	7.37E-01	8.39E-01	9.55E-01	-4.78E+00
48	-7.84E+02	-5.21E+00	-2.55E+03	-9.94E+00	-3.40E+00	-2.82E-01	2.09E-01	-4.05E-01	-1.24E+01
49	-5.61E+01	-7.90E-02	-4.22E+00	-1.69E+03	-8.91E-01	-9.85E+00	-1.21E+00	-2.00E+00	-4.51E+00
50	-6.14E+01	-2.50E-01	-8.23E+00	-3.96E+01	2.92E-01	7.82E-01	-2.76E+00	-4.58E+00	-5.48E+00
51	-1.57E+02	-8.19E-01	-1.73E+00	7.15E-01	-9.98E-01	-2.40E+00	-7.48E-01	-1.54E+00	-6.27E-01
52	-1.07E+02	-8.24E-01	-6.12E-01	6.22E-01	-1.00E+00	-5.99E+00	-8.53E-01	-1.71E+00	3.37E-01
53	-4.48E+02	-3.32E+00	-5.17E+00	-1.07E+02	-6.35E+00	-1.61E+00	-7.74E+00	-1.22E+01	-1.60E+01
54	-4.50E+00	-9.55E+00	9.92E-01	-8.54E+01	9.99E-01	-1.48E+01	-2.36E+00	-1.16E+00	-5.96E+00
55	-7.29E+01	-2.32E+00	-2.74E+00	-9.71E-01	-2.75E+00	-1.22E+00	-2.41E+00	-3.26E+00	-1.54E+00
56	-9.16E+02	-1.38E+00	-2.52E+01	6.82E-01	-1.13E+01	-2.54E-01	-8.92E+00	-1.50E+01	-2.76E+01
57	-1.11E+03	-1.15E+00	-2.11E+01	8.04E-01	-1.80E+01	-7.57E-01	-1.25E+01	-2.08E+01	-4.15E+01
58	-9.22E+01	-1.43E+00	-2.87E+00	-2.79E+00	-2.03E+00	-1.67E+01	-2.57E+00	-3.91E+00	2.44E-01
59	-8.28E+01	-1.13E+00	-1.30E-01	-3.27E+01	-1.21E+00	-1.83E+00	-2.39E+00	-3.51E+00	-7.18E-01
60	-4.70E+00	-3.64E-01	-1.13E+00	-5.17E+01	-7.14E-01	-4.95E+00	-6.26E+00	-1.07E+01	4.11E-01
61	-3.64E+02	-2.89E+00	-3.62E+00	5.69E-01	-8.89E+00	-9.10E+00	-8.36E+00	-1.37E+01	-2.70E+01
62	-2.11E+02	8.27E-01	-1.84E+00	-4.76E+01	4.60E-02	-6.80E-02	6.15E-01	6.13E-01	-8.82E+00
63	-4.00E+00	-4.00E+00	-4.00E+00	-4.00E+00	-4.00E+00	-6.93E+00	-4.00E+00	-4.00E+00	-4.00E+00

64	-3.96E+00	-2.54E+00	-4.45E+00	-2.47E+01	-4.05E+00	-1.12E+01	-4.39E+00	-4.92E+00	-6.06E+00
65	-1.67E+03	-2.38E+00	-6.83E+01	-9.06E-01	-1.95E+01	9.13E-01	-1.60E+01	-2.67E+01	-4.91E+01
66	-4.43E+00	-2.18E+00	-4.41E+00	-2.59E+02	-3.20E+00	-1.60E+01	-1.69E+00	-2.44E+00	-4.22E+00
67	-5.79E+00	-2.14E+00	-4.74E+00	-5.94E+01	-3.95E+00	-1.53E+01	-4.64E+00	-5.53E+00	-5.70E+00
68	-5.21E+00	-2.71E+00	-4.34E+00	-4.13E+00	-3.81E+00	-1.92E+01	-4.38E+00	-4.92E+00	-4.37E+00
69	-8.30E+02	-2.84E+00	-2.08E+01	6.10E-01	-1.38E+01	-2.19E+00	-1.19E+01	-1.91E+01	-2.97E+01
70	-4.67E+00	-2.14E+00	-4.60E+00	-2.65E-01	-2.91E+00	-2.08E+01	-3.72E+00	-3.97E+00	-1.31E+00
71	-1.82E+00	-2.41E+00	-1.44E+00	-2.34E+01	-1.68E+00	-1.57E+01	-3.91E+00	-3.74E+00	9.38E-01
72	-1.76E-01	-1.98E+00	-1.92E+03	*ND*	*ND*	*ND*	*ND*	*ND*	*ND*
73	-9.53E+02	-2.02E+00	-2.78E+01	-1.07E+02	-1.60E+01	-2.78E+01	-1.40E+01	-2.28E+01	-4.52E+01

ND – Not determined

Table S4. The HOMA(R) and HOMA(σ(^{13}C)) indices for 73 rings (Figure 1) calculated with the B3LYP/D3, ωB97-XD, and TPSSh functionals based on geometries optimiized with the aug-cc-pVTZ basis set, and the isotropic σ(^{13}C) shielding constants calculated with the pcSeg-2 basis set.

#	B3LYP			ωB97-XD			TPSSh		
	HOMA(R)	HOMA(σ(^{13}C))	HOMA($^1J_{CC}$)	HOMA(R)	HOMA(σ(^{13}C))	HOMA($^1J_{CC}$)	HOMA(R)	HOMA(σ(^{13}C))	HOMA($^1J_{CC}$)
1	1.0000	1.0000	1.0000	1.0000	1.0000	1.0000	1.0000	1.0000	1.0000
2	1.0000	-1.2662	-0.3322	1.0000	-0.4565	-7.7448	1.0000	0.4017	-19.1156
3	0.9999	0.9988	-140.3157	1.0000	-23.5185	0.7397	0.9998	0.9995	-42.8555
4	0.9861	0.9397	0.6040	0.9870	0.9508	0.5496	0.9879	0.9390	0.4908
5	0.9597	0.1373	0.7849	0.9619	0.1704	0.8913	0.9428	0.1211	0.8790
6	0.9334	0.9929	0.9567	0.9434	0.9948	0.9581	0.9365	0.9937	0.9555
7	0.9093	0.9927	0.9511	0.9164	0.9948	0.9610	0.9123	0.9937	0.9573
8	0.8968	0.0383	-6689.3615	0.8982	0.7741	-2100.21	0.8860	0.8536	-1.0886
9	0.8923	0.5458	0.9043	0.8959	0.5847	0.8948	0.8922	0.5393	0.8925
10	0.8858	0.3087	0.5894	0.8883	0.3888	-0.0217	0.8872	0.2801	0.0355
11	0.8794	0.2444	-0.0033	0.8756	0.2227	-0.1822	0.7435	0.1740	-0.2978
12	0.8658	0.8652	0.3413	0.8669	0.8911	0.3141	0.8763	0.8634	0.0751
13	0.8373	0.9912	0.8960	0.8299	0.9935	0.9130	0.8403	0.9925	0.9158
14	0.8353	0.9839	0.8976	0.8297	0.9877	0.8773	0.8419	0.9855	0.8982
15	0.8293	-0.3250	-76.2839	0.8225	-0.5166	-0.3975	0.8458	0.4174	-17.3780
16	0.8191	0.9943	0.9200	0.8104	0.9953	0.9324	0.8185	0.9942	0.9353
17	0.7994	0.9926	0.8064	0.8446	0.9949	0.8449	0.7960	0.9941	0.8079
18	0.7604	0.9870	0.9200	0.7179	0.9885	0.9324	0.7638	0.9854	0.9353
19	0.7586	-0.3545	-6.4525	0.7728	-0.0683	-6.3113	0.7748	-0.0544	-6.3889
20	0.7571	0.0085	0.5718	0.7137	0.0437	0.6591	0.7624	0.0184	0.5350
21	0.7432	0.0795	0.3541	0.6006	0.2014	0.3813	0.7547	0.1033	0.2494
22	0.7350	0.7809	-3.3410	0.7017	0.6535	-3.0395	0.7373	0.7590	-11.7028
23	0.7224	0.3114	-0.1527	0.7094	0.5694	-0.2209	0.7347	-0.2769	-0.7542
24	0.7071	0.8374	-0.1175	0.6893	0.8633	-0.1674	0.7295	0.8394	-0.6901
25	0.7038	0.9948	0.8107	0.6612	0.9959	0.8277	0.7142	0.9957	0.8507
26	0.6999	0.5681	0.4935	0.7702	0.6906	0.4880	0.6866	0.6002	-1.3389
27	0.6344	-3.7959	0.3278	0.5746	-3.0230	0.3840	0.6830	-3.4806	0.8885
28	0.6089	0.9514	0.6716	0.6214	0.9516	0.6259	0.6189	0.9514	0.6066
29	0.5774	0.9933	0.8663	0.5228	0.9957	0.8811	0.5878	0.9945	0.8980
30	0.4381	0.9311	0.6720	0.4574	0.9368	0.6614	0.4493	0.9326	0.6491
31	0.4341	-0.0281	-0.2665	0.4383	0.0318	-0.1711	0.4287	-0.0381	-10.9906
32	0.3699	-0.9290	-12.6124	0.2211	-1.0781	-27.6539	0.3709	-0.5279	-27.9984
33	0.2977	0.9959	0.9450	0.2033	0.9948	0.9518	0.3227	0.9937	0.9793
34	0.2749	0.4426	-5.5912	0.1667	0.4172	-4.6142	0.2958	0.4270	-10.0492
35	0.2276	0.9902	0.9428	0.1010	0.9950	0.9687	0.2716	0.9904	0.9873
36	0.1221	-1.6890	-2.1039	0.1646	-1.5902	-1.9831	0.1084	-1.6694	-0.7805
37	0.0190	0.3019	-85.1270	-0.0111	0.3550	-137.23	0.0445	0.3101	-186.14
38	-0.0242	-1.4276	0.6857	-0.1659	-1.4108	-28.5337	-0.0252	-1.4001	0.6729
39	-0.1434	0.9689	-0.3871	-0.2812	0.9812	-0.2161	-0.1045	0.9579	-0.1823
40	-0.2234	0.8404	-3.9767	-0.3701	0.8777	-3.4792	-0.1237	0.8365	-4.0527
41	-0.2982	0.1155	-1.6228	-0.2120	0.2761	-7.1044	-0.1202	0.2148	-16.5323
42	-0.6112	0.0583	0.2807	-1.0379	-0.7471	0.2301	-0.5888	-0.2719	0.4905
43	-0.6344	-0.0071	-0.3825	-0.7123	0.0372	-0.2912	-0.6006	-0.0087	-0.2513
44	-0.6961	-0.5781	-209.45	-0.3857	-0.2875	-57.6282	-0.5244	-0.6258	-1878.52
45	-0.7182	0.2560	-0.6046	-0.7812	0.2670	-0.4975	-0.6640	0.2533	-0.4301
46	-0.7665	0.6708	-0.3030	-0.8899	0.7378	-0.2555	-0.7046	0.6562	0.5170
47	-1.1627	0.9856	0.9840	-1.3235	0.9750	0.9976	-1.0802	0.9899	0.9874
48	-1.1657	-2.3706	-2.5096	-1.1562	-2.2291	-1.9331	-1.1769	-2.2606	-1.9989
49	-1.2193	-0.5889	-1.6945	-1.3417	-0.5917	-1.6239	-1.2216	-0.5741	-1.6172
50	-1.3176	0.7330	-0.2484	-1.4165	0.7804	-0.1761	-1.2300	0.7204	-0.6901
51	-1.3722	-0.6591	-1.2273	-1.4557	-0.6636	-1.0695	-1.3266	-0.6372	-1.0191
52	-1.4226	-0.8071	-1.0344	-1.5165	-0.7929	-0.9423	-1.3906	-0.8002	-0.8803
53	-2.1619	-1.8028	-10.3944	-2.2641	-1.6663	-9.6848	-2.0402	-1.7218	-9.5496
54	-2.2500	-7.7478	-13.8342	-2.2352	-7.2747	-13.0808	-2.2083	-7.6928	-13.0909
55	-2.7079	-2.5180	-2.6076	-2.7614	-2.5013	-2.5358	-2.6860	-2.5103	-1.3210
56	-2.9685	-0.2019	-42.7587	-3.0936	-0.2510	-48.2483	-2.8741	-0.1030	-52.5256
57	-2.9934	-0.4879	-51.3325	-3.1174	-0.5273	-63.7522	-2.8975	-0.4066	-71.6401
58	-3.0478	-1.8514	-2.4934	-3.0747	-1.8181	-2.4215	-2.9970	-1.8212	-2.3852
59	-3.1844	-1.3671	-3.4880	-3.1751	-1.3809	-3.5524	-3.0511	-1.3407	-3.4712
60	-3.2855	-1.5465	-3.0356	-3.2858	-1.5180	-3.1664	-3.2272	-1.4950	-1.2206
61	-3.5394	-2.1380	-5.4135	-3.5825	-2.0669	-4.8630	-3.4717	-2.0744	-6.4153
62	-3.7225	0.9234	-59.0788	-3.6703	0.9357	-26.4054	-3.6371	0.9216	-39.9600
63	-4.0000	-4.0000	-4.0000	-4.0000	-4.0000	-4.0000	-4.0000	-4.0000	-4.0000

64	-4.2335	-3.8503	-3.6988	-4.2425	-3.8361	-3.6626	-4.2579	-3.8373	-3.6715
65	-4.3477	-2.8458	-216.0688	-4.5545	-2.5869	-263.5262	-3.7436	-2.5646	-30.2431
66	-4.4241	-3.3339	-4.6905	-4.4323	-3.4349	-4.7282	-4.3318	-3.3364	-4.6922
67	-4.4261	-4.1575	-3.4236	-4.4288	-4.1025	-3.3655	-4.4763	-4.1380	-3.3922
68	-4.5036	-4.7413	-3.8596	-4.4941	-4.6615	-3.8649	-4.4861	-4.7103	-3.8747
69	-4.6770	-1.7847	-34.6802	-4.7493	-1.8355	-38.8046	-4.5991	-1.6588	-41.2001
70	-4.7215	-4.1187	-4.1093	-4.6759	-4.0469	-4.1403	-4.7064	-4.0737	-4.1473
71	-5.3762	-4.5384	-5.8397	-5.3318	-4.5244	-6.0684	-5.2684	-4.4353	-6.0583
72	-5.3918	-5.4983	-84.8550	-5.2520	-5.3042	-30.9502	-5.2398	-5.3876	-34.7535
73	-5.7345	-3.3097	-24.9569	-7.2129	-3.1999	-10.9943	-7.1679	-3.2013	-11.5581
α	250.808	254.2175	253.2535	$4.51003\cdot10^{-4}$	$4.2196\cdot10^{-4}$	$4.89006\cdot10^{-4}$	$6.29888\cdot10^{-3}$	$7.27813\cdot10^{-3}$	$7.2791\cdot10^{-3}$

Table S5. The fit parameters for all correlations presented in the manuscript. R^2 is reflecting the percentage of the dependent variable variation that the used model explains, coefficient value,σ standard error, t Student's t-test value, P values indicate whether the for the coefficients in the elationships are statistically significant.

Figure	Fitting equation	R^2	Coefficient, σ, t, P	
2a Red line	f = a·x+b	0.9941	b<br>a	-0.0697,0.0206,-3.3850,0.0012<br>1.0066,0.0099,101.2757,<0.0001
2a Blue line	f = a·x+b	0.9549	b<br>a	-1.1189,0.3553,-3.1490,0.0162<br>1.2900,0.1059,12.1807,<0.0001
2b Red line	f = a·x+b	0.9922	b<br>a	-0.0850,0.0236,-3.6074,0.0006<br>0.9995,0.0114,87.8898,<0.0001
2b Blue line	f = a·x+b	0.9565	b<br>a	-1.3799,0.3714,-3.7153,0.0138<br>1.0766,0.1027,10.4844,0.0001
2c Red curve	f = a·exp(c·x)+b	0.9652	b<br>a<br>c	-5.5387,0.4582,-12.0874,<0.0001<br>4.9559,0.5065,9.7838,<0.0001<br>0.2810,0.0390,7.2128,<0.0001
2d Red curve	f = a·exp(c·x)+b	0.9938	b<br>a<br>c	-9.2201,0.7717,-11.9480,<0.0001<br>8.9376,0.7882,11.3397,<0.0001<br>0.1338,0.0143,9.3507,<0.0001
2d Blue curve	f = a·exp(c·x)+b	0.9509	b<br>a<br>c	-17.7416,17.1782,-1.0328,0.3601<br>14.8032,16.9505,0.8733,0.4318<br>0.1033,0.1570,0.6579,0.5465
2e Red curve	f = a·exp(c·x)+b	0.9960	b<br>a<br>c	-11.8487,1.1383,-10.4088,<0.0001<br>11.6202,1.1511,10.0945,<0.0001<br>0.0984,0.0114,8.6549,<0.0001
2e Blue curve	f = a·exp(c·x)+b	0.9875	b<br>a<br>c	-37.4594,65.3865,-0.5729,0.6068<br>35.7164,65.2203,0.5476,0.6221<br>0.0388,0.0789,0.4913,0.6569
2f Red curve	f = a·exp(c·x)+b	0.8737	b<br>a<br>b	-4.7439,0.8498,-5.5821,<0.0001<br>4.3726,0.9184,4.7613,<0.0001<br>0.2441,0.0700,3.4868,0.0009
2g Red line	f = a·x+b	0.9706	b<br>a	0.1080,0.0602,1.7937,0.0793<br>0.9139,0.0232,39.3660,<0.0001
3c Red line	f = a·x+b	0.9384	b<br>a	0.1620,0.1586,1.0210,0.3167<br>0.6240,0.0728,8.5678,<0.0001
3c Blue line	f = a·x+b	0.9877	b<br>a	-4.8341,0.2508,-19.2734,<0.0001<br>5.9615,0.3330,17.9024,<0.0001
3c Green curve	f = a·exp(c·x)+b	0.9999	b<br>a<br>c	-74.0066,2.8187,-26.2558,0.0242<br>61.2382,2.8654,21.3716,0.0298<br>0.1788,0.0117,15.2995,0.0416
3d Red line	f = a·x+b	0.9537	b<br>a	-176.0314,2.7826,-63.2622,<0.0001<br>-19.2088,1.4963,-12.8372,<0.0001
3e Red curve	f = (1+a·x)/(b+c·x)	0.9453	a<br>b<br>c	-0.5108,0.1333,-3.8311,0.0064<br>-0.0005,2.8080E-005,-19.5198,<0.0001<br>0.0004,8.2429E-005,5.3970,0.0010
3g Red line	f = a·x+b	0.4569	b<br>a	0.5750,0.2004,2.8693,0.0081<br>0.4303,0.0920,4.6767,<0.0001
3g Blue line	f = a·x+b	0.9880	b<br>a	-1.2561,0.1022,-12.2962,0.0003<br>2.4590,0.1356,18.1311,<0.0001
3h Red line	f = a·x+b	0.9517	b<br>a	-45.7755,1.2313,-37.1756,<0.0001<br>-8.3093,0.6622,-12.5490,<0.0001

Table S5. (continued)

4a<br>Black curve	f = (1+a·x)/(b+c·x)	0.9394	a<br>b<br>c	1.0838,0.1708,6.3472,<0.0001<br>1.6450,0.2457,6.6938,<0.0001<br>0.1393,0.0327,4.2550,0.0001
4b<br>Black curve	f = (1+a·x)/(b+c·x)	0.8534	a<br>b<br>c	1.2417,0.3127,3.9708,0.0002<br>1.9508,0.4720,4.1328,0.0001<br>0.1141,0.0579,1.9707,0.0548
S4b<br>Green curve	f = (1+a·x)/(b+c·x)	0.8551	a<br>b<br>c	0.6061,0.2017,3.0044,0.0084<br>-0.1032,0.0260,-3.9630,0.0011<br>0.0788,0.0269,2.9322,0.0098
S4c<br>Green curve	f = (1+a·x)/(b+c·x)	0.7235	a<br>b<br>c	0.2629,0.1478,1.7790,0.0851<br>-0.0159,0.0038,-4.2086,0.0002<br>0.0085,0.0044,1.9413,0.0614
5a<br>Red line	f = a·x+b	0.9361	b<br>a	0.1447,0.0982,1.4737,0.1495<br>0.9517,0.0420,22.6431,<0.0001
5b<br>Blue line	f = a·x+b	0.9107	b<br>a	-0.4158,0.1057,-3.9328,0.0004<br>0.9800,0.0519,18.8895,<0.0001
S6a<br>Green curve	f = (1+a·x)/(b+c·x)	0.8548	a<br>b<br>c	-2.5952,0.9695,-2.6768,0.0190<br>0.0132,0.0055,2.4024,0.0319<br>-0.0043,0.0021,-2.0952,0.0563
S6a<br>Blue<br>curve	f = (1+a·x)/(b+c·x)	0.8292	a<br>b<br>c	0.2457,0.0907,2.7085,0.0112<br>-0.0137,0.0024,-5.8064,<0.0001<br>0.0066,0.0028,2.3320,0.0269
S6b<br>Green curve	f = (1+a·x)/(b+c·x)	0.8599	a<br>b<br>c	0.5391,0.2055,2.6229,0.0201<br>-0.0214,0.0053,-4.0230,0.0013<br>0.0163,0.0056,2.9367,0.0108
7a<br>Red line	f = a·x+b	0.9971	b<br>a	-0.0980,0.0102,-9.5902,<0.0001<br>1.0905,0.0130,83.5671,<0.0001
7a<br>Green line	f = a·x+b	0.9737	b<br>a	0.2291,0.0396,5.7899,0.0044<br>0.8134,0.0669,12.1630,0.0003
7b<br>Red line	f = a·x+b	0.9844	b<br>a	1.468e-1, 1.164e-2,7.928e+0, 0.3944879<br>8.317e-1, 2.538e-2,3.051e+0, 0.3944879
7d<br>Red line	f = a·x+b	0.8958	b<br>a	0.3439,0.0600,5.7304,0.0003<br>0.6498,0.0739,8.7977,<0.0001
7d<br>Green line	f = a·x+b	0.9942	y0<br>a	-1.9434,0.1713,-11.3448,0.0560<br>2.6018,0.1991,13.0713,0.0486

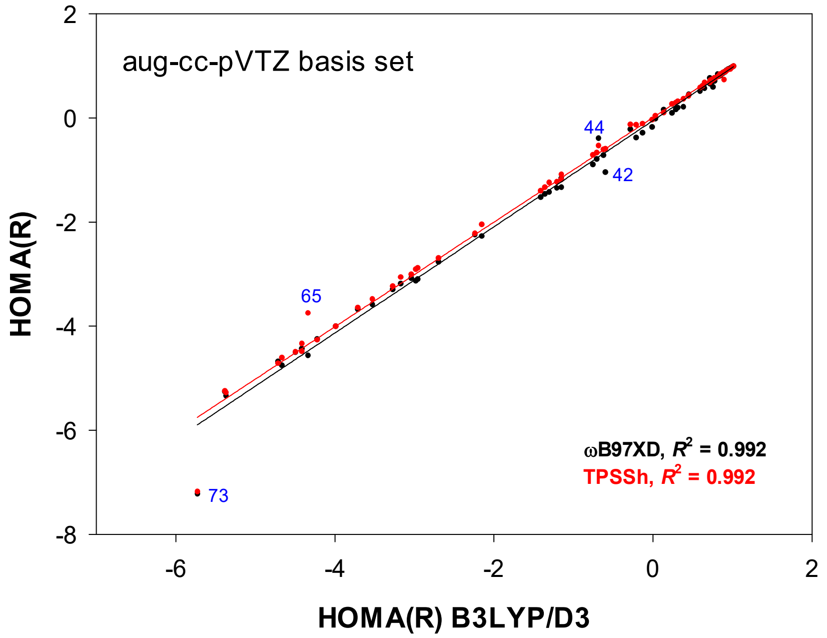

Figure S2. Linear correlations between the HOMA(R) indices calculated using the three DFT functions. The outlying points were excluded: four-membered ring in benzocyclobutadiene dianion (42), cyclohex-1-en-3,5-diyne (44), cyclohex-1,4-diyne (65), and cyclopent-1-yne (73).

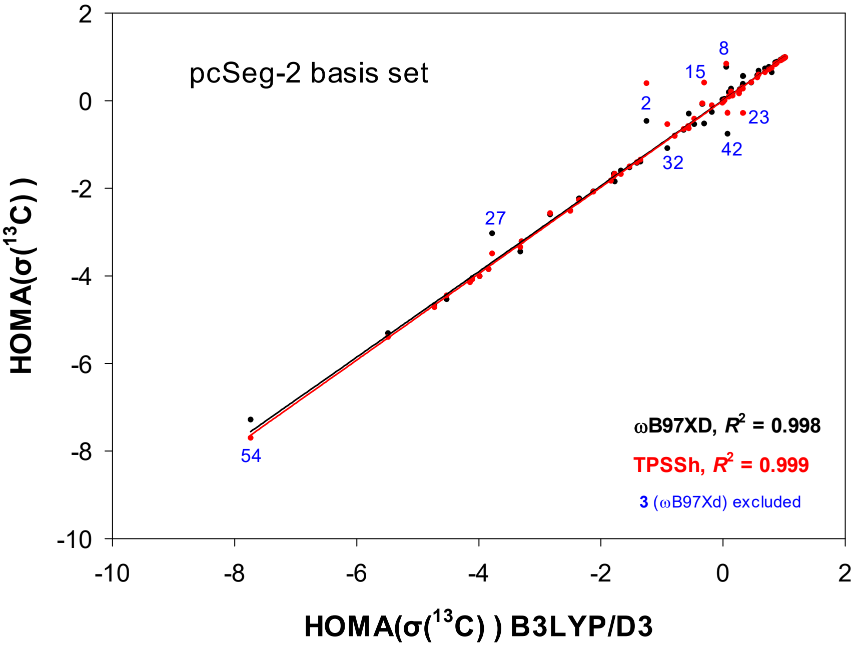

Figure S3. HOMA(σ(^{13}C)) indices obtained with different functionals display linear correlations. The outlying points were excluded: benzene anion (2), benzene dianion (27), cycloheptatriene dianion (8), naphthalene dianion (15), six- and four-membered ring in benzocyclobutadiene dianion (23) and (42), cyclobuta-1-en-3-yne (32), and cyclopropene (54).

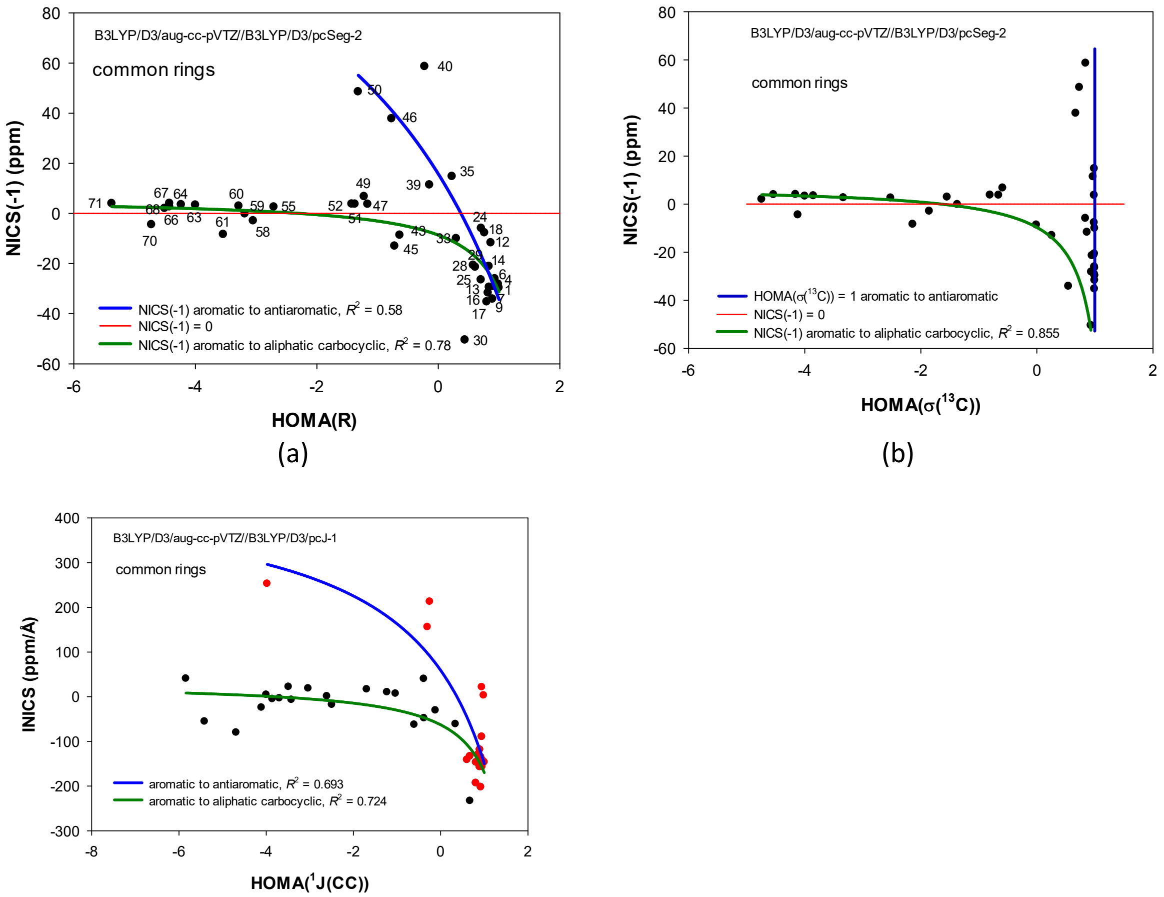

(a) (b) (c)

Figure S4. Correlations between the HOMA(R) and HOMA(σ(^{13}C)) and NICS(-1) indices, a) and b), respectively calculated with B3LYP functional depend on types of the carbocyclic rings considered for common neutral carbocyclic rings devided to aromatic, aliphatic, and antiaromaetic rings.

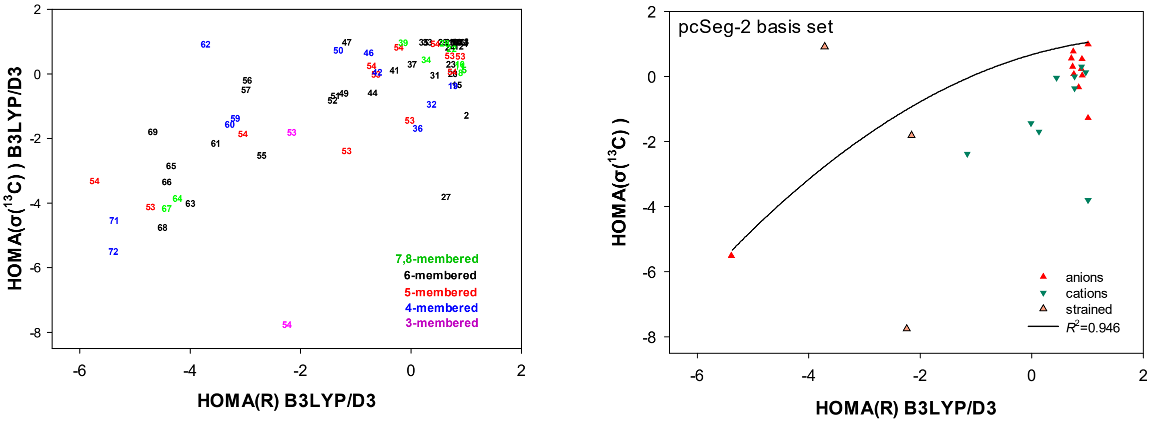

(a) (b)

Figure S5. Correlations between the HOMA(R) and HOMA(σ(^{13}C)) indices calculated with B3LYP functional depend on types of the carbocyclic rings considered: a) rings of different size, b) deviation of the HOMA(σ(^{13}C)) indices for anionic, cationic, and strained rings, from the correlation line for the common rings and rings with triple bonds, fitting ponts in S5a.

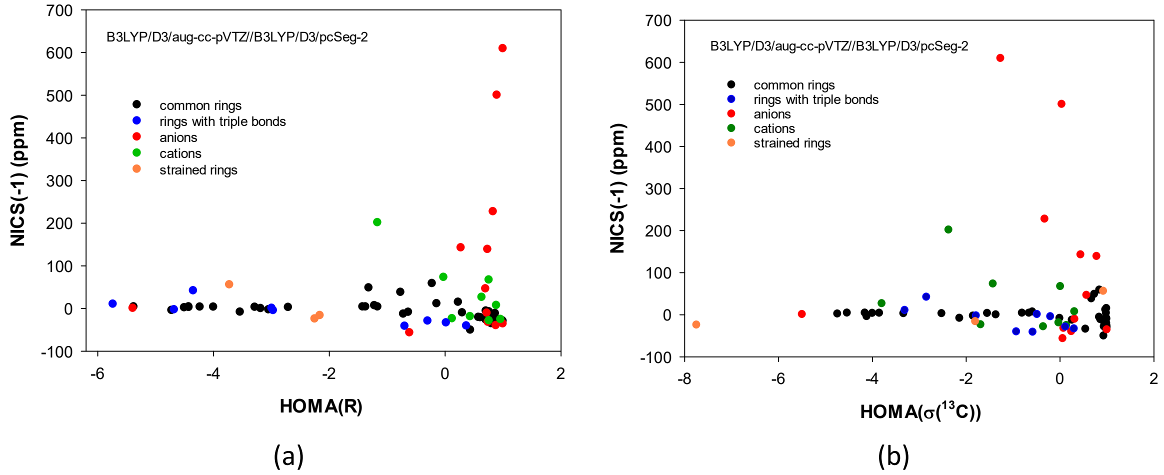

Figure S6. Correlations between the HOMA(R) and HOMA(σ(^{13}C)) and NICS(-1) indices, a) and b) respectively, calculated with B3LYP functional depend on types of the carbocyclic rings considered for all types of rings considered.

Illustration of the role of the bias (EN) and variance (GEO) components in the correlations presented in the manuscript

The relationships in Figure S7a-n are shown according to the order in which the parameters appear in the manuscript, *i.e.*, starting from the electrical ones. HOMA(X), 1-EN(X), and 1-GEO(X) data can be find in Table S4. The 1–EN(R) and 1–GEO(R) components are used in the graphs (Figure S7a-n) to conserve the way HOMA varies. Additionally, to avoid point overlapping, –EN and –GEO instead of 1–EN and 1–GEO parameters are shown on the ordinate axis, which shifts the values from the HOMA ones. At the plots denoted by "1", comparisons of the HOMA(X), –EN(X), and –GEO(X) are shown as functions of HOMA(R), 1–EN(R), and 1–GEO(R), where X is an arbitrary variable and R is bond distance. At the plots denoted by "2", comparisons of the 1–EN(X) and 1–GEO(X) are presented as functions of HOMA(X).

In Figures S7a1 and S7a2, X is set to be R to demonstrate the ideal case. In Figure S7a1, –EN(R) and –GEO(R) are positioned on a straight line parallel to HOMA(R). Figure S7a2 shows that 1–EN(R) and 1–GEO(R) are at Y=1 and Y=X straight lines and between them. **Hence, neither of the components alone determines HOMA(R) for the large and diversified set of rings.**

In Figures S7b1 and S7b2, X is set to be Rho—the electron density in a bond critical point**.** Figure S7b1 shows that both HOMA and EN correlate quite well, while **GEO is responsible for the side trend in Figure 2a of the manuscript.** Thus, for some rings, the variance of Rho varies stronger than the variance of bond distance, while biases vary pretty much the same (which can be better seen in Figures S7b3 and S7b4)**.**

Figures S7a2 and S7b2 look very similar at first glance; however, a closer look reveals a relatively different spread of the red (EN, bias) and white (GEO, variance) points between the Y=1 and Y=X straight lines**.** Different variables disclose diverse aspects of the ring's "aromaticity" in terms of similarity to the benzene ring.

The patterns observed for the Rho variable (Figures S7b1 and S7b2) are similar for the Δ, G, V, and K electron density parameters at bond critical points (Figures S7c1-g1 and S7c2-g2). For G, they seemingly are different; however, including the outlying points (Figure S7d1) has fouled up the picture. For ε and ESP, the patterns are less similar (Figures S7h1,i1 and S7h2,i2) for the reasons discussed in the manuscript.

The colossal difference in the pictures occurs for the Rho and Δ electron density parameters at ring critical points (Figures S7j1,k1 and S7j2,k2). They have no GEO components (no variance) because one value in RCP characterizes the entire ring in comparison to six BCP values for six bonds in six membered rings. For these parameters clearly only their EN (bias) components determine the corresponding HOMA values.

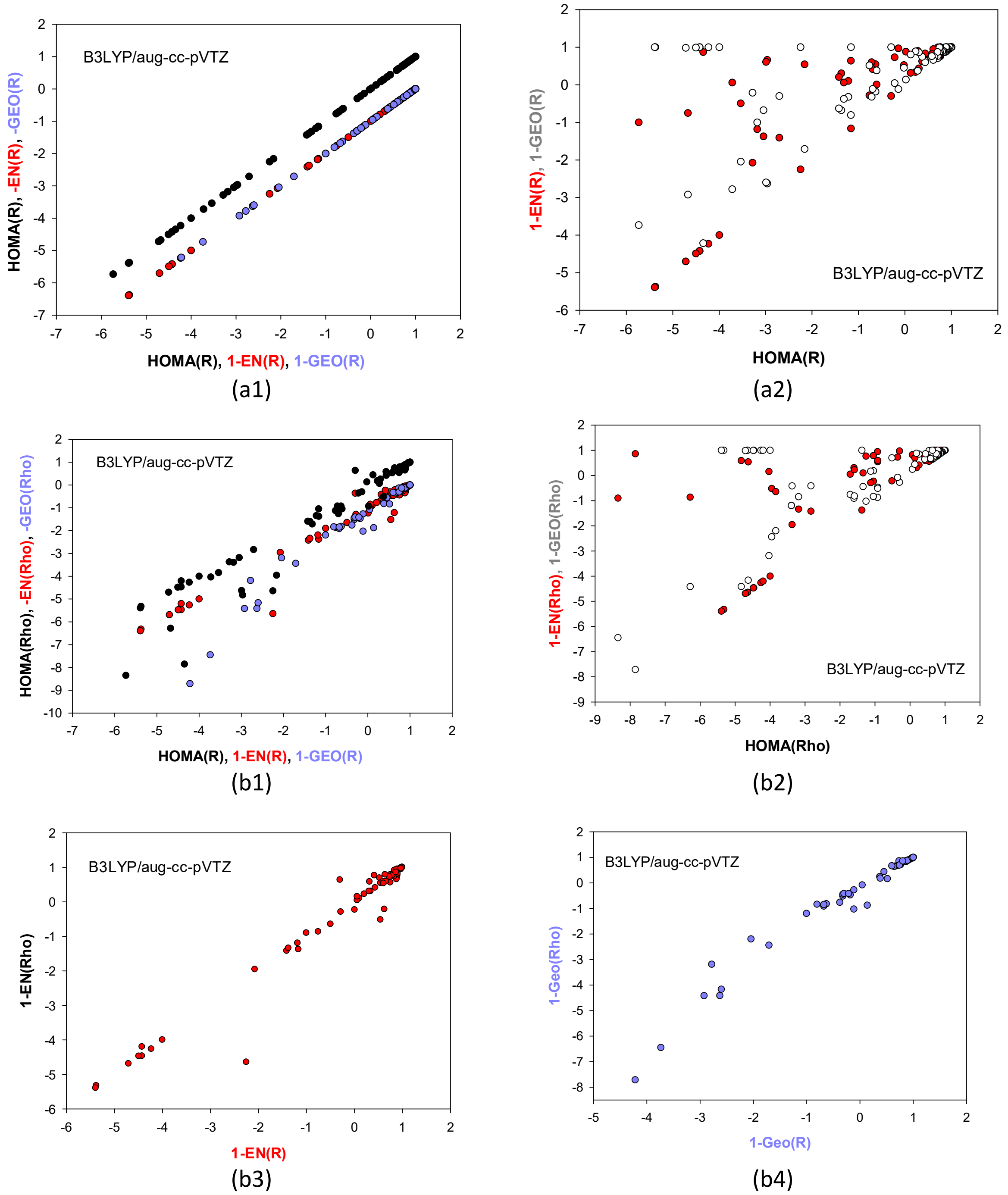

Figure S7. The relationships between the HOMA(R), 1-EN(R), and 1-GEO(R) and analogous parameters of X variables (see Table S4) for the optimized molecules from Figure 1 calculated using B3LYP functional and the aug-cc-pVTZ, (optimization and AIM parameters) the pcSeg−2 (^{13}CNMR)and pcJ−1 (^{1}J(CC)) basis and set**s.**

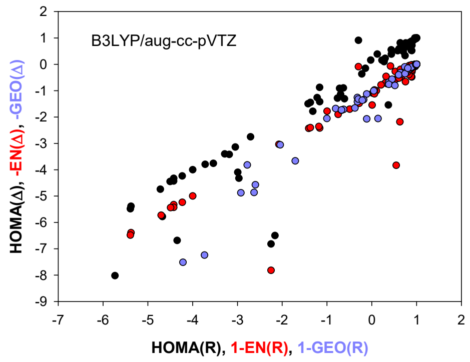

(c1)

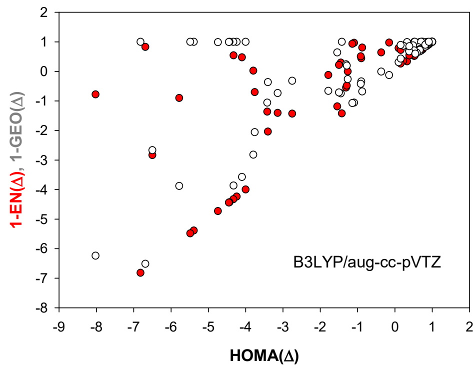

(c2)

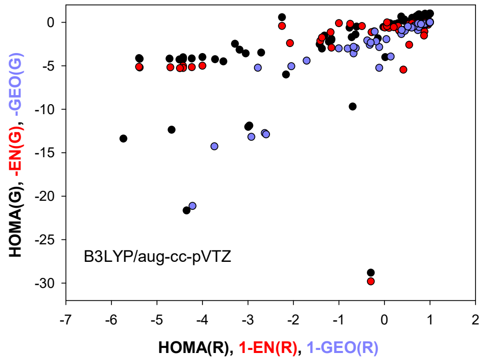

(d1)

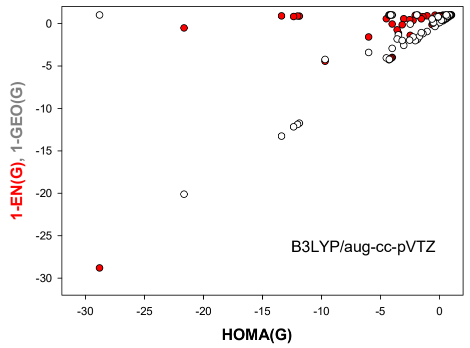

(d2)

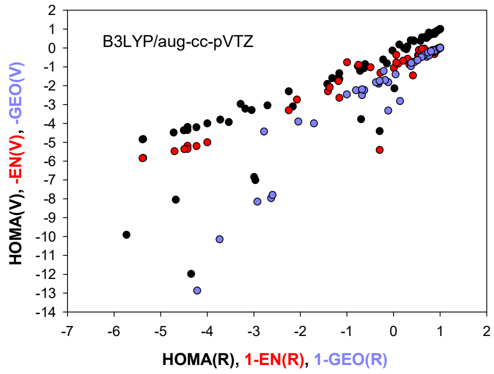

(e1)

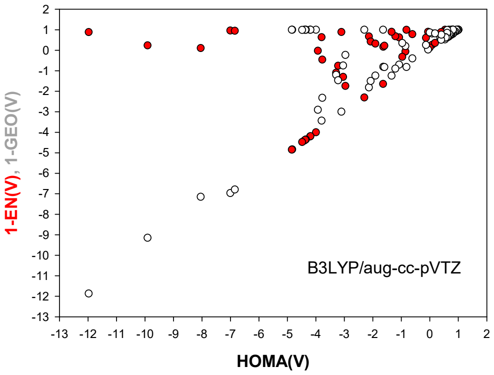

(e2)

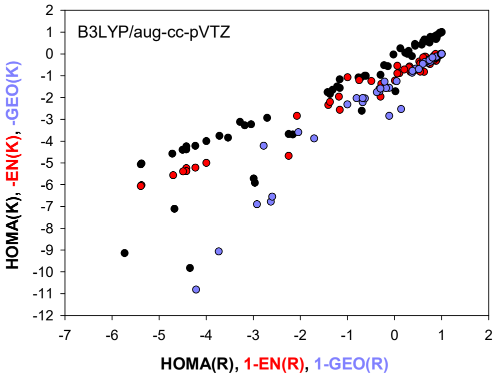

(f1)

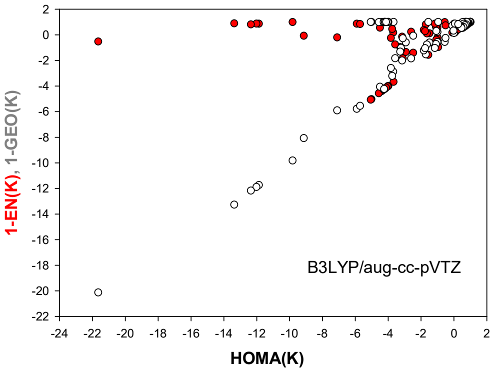

(f2)

Figure S7. (contined)

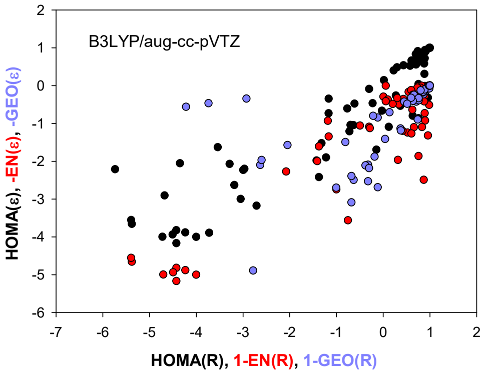

(g1)

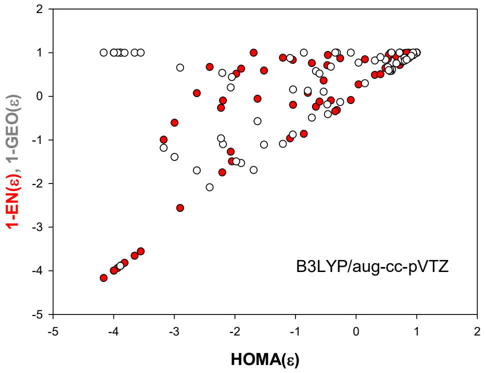

(g2)

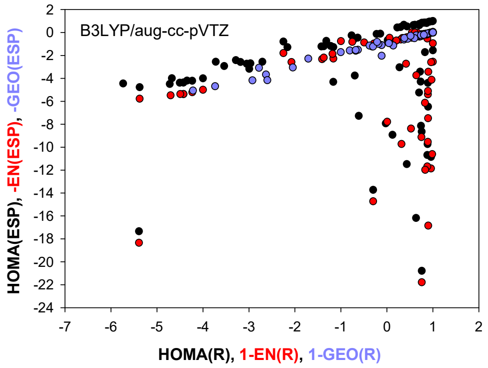

(h1)

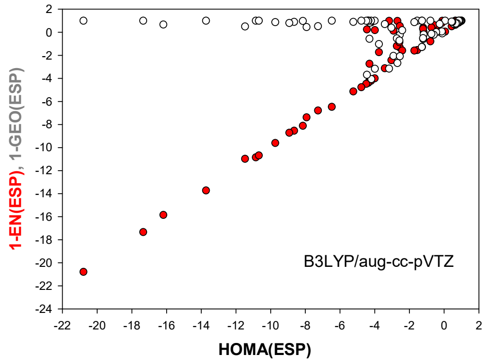

(h2)

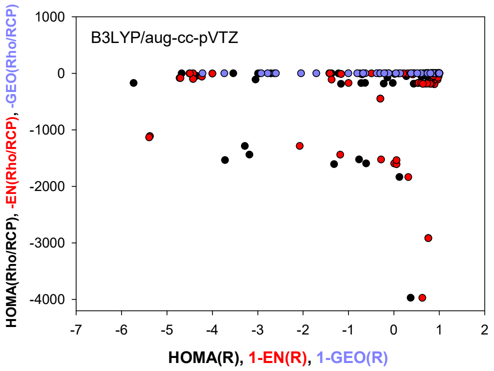

(i1)

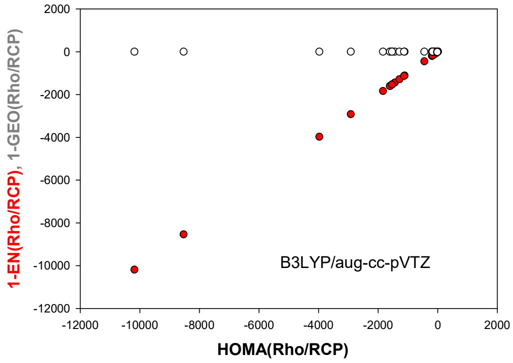

(i2)

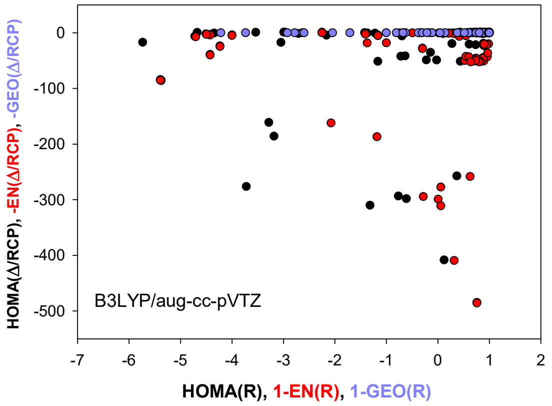

(j1)

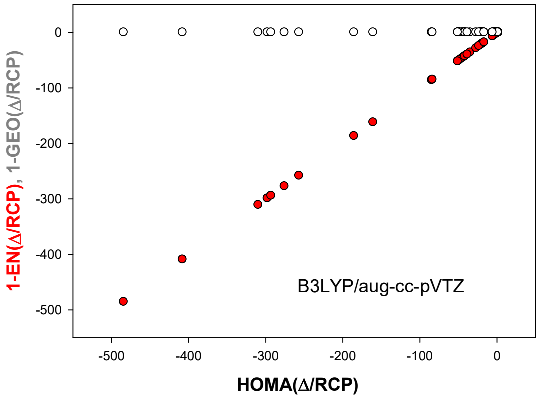

(j2)

Figure S7. (contined)

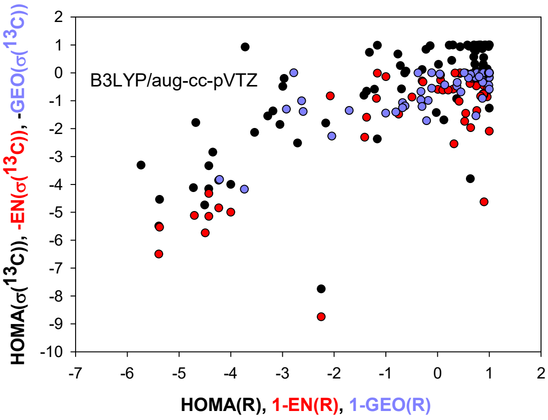

(k1)

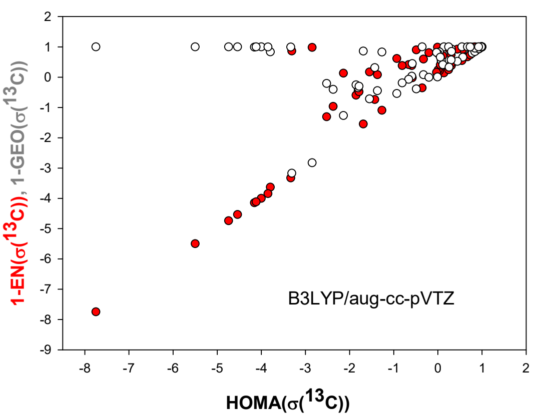

(k2)

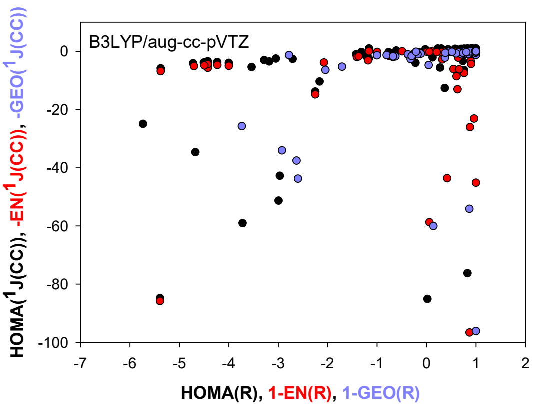

(l1)

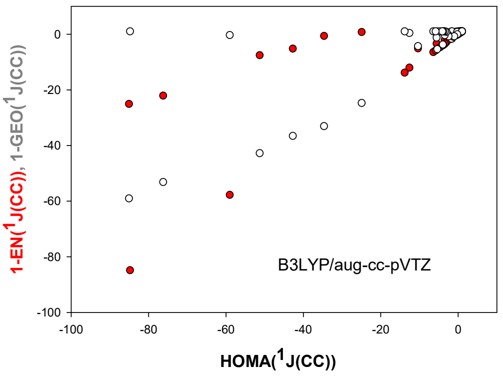

(l2)

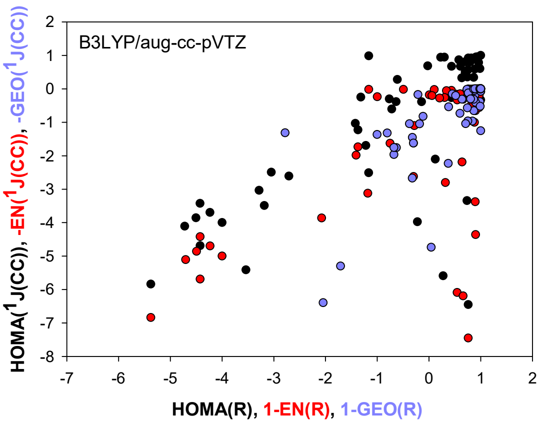

(m1)

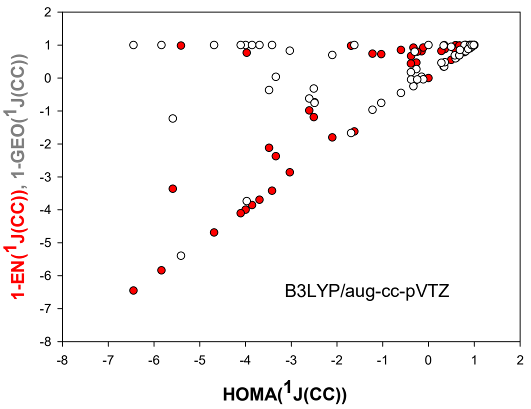

(m2)

Figure S7. (contined)

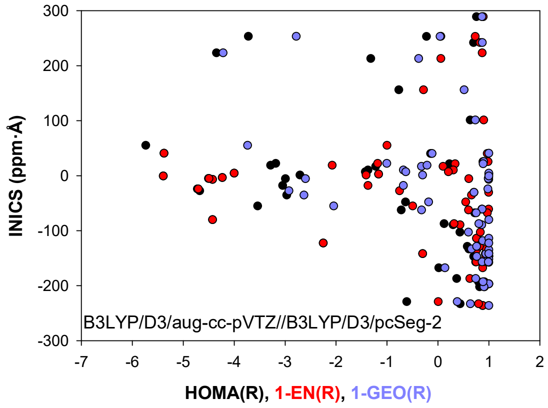

(m3)

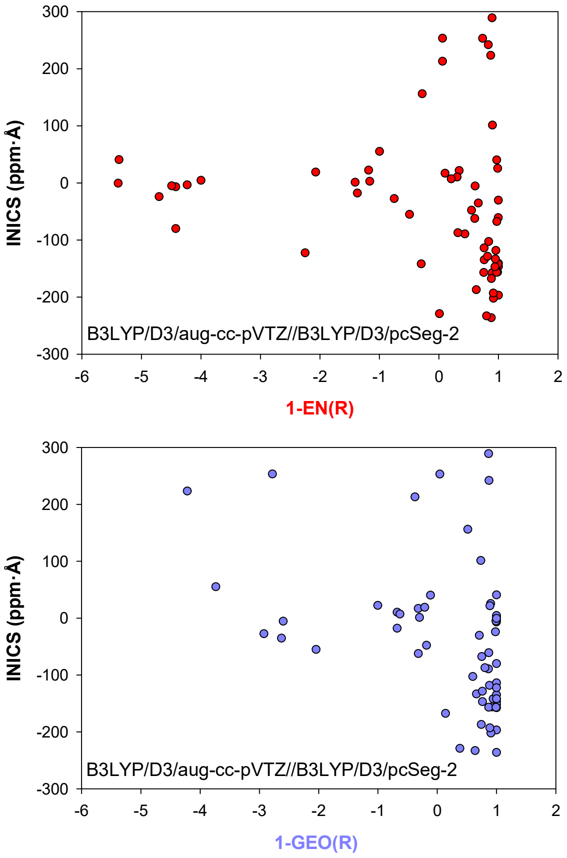

(m4)

Figure S7. (contined)

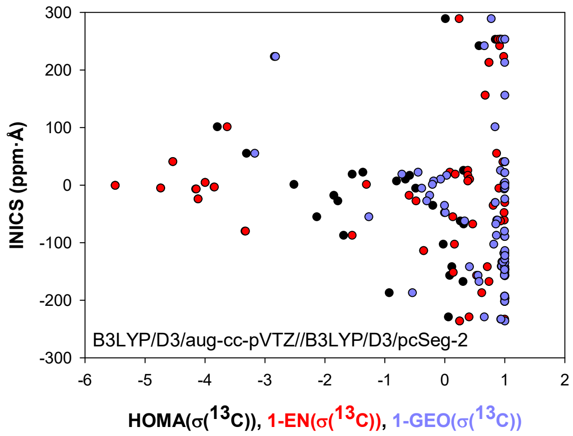

(n1)

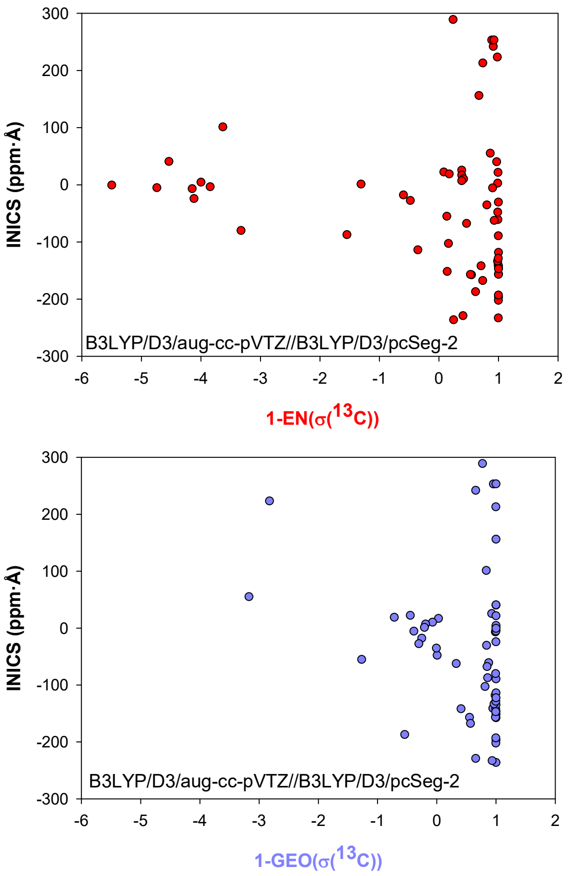

(n2)

Table S6. The HOMA, EN, and GEO values for the optimized molecules from Figure 1 calculated using B3LYP functional and the aug-cc-pVTZ, (optimization and AIM parameters) the pcSeg−2 (^{13}CNMR)and pcJ−1 (^{1}J(CC)) basis and sets.

mol	HOMA(R)	EN(R)	GEO(R)	HOMA(Rho)	EN(Rho)	GEO(Rho)	HOMA(Δ)	EN(Δ)	GEO(Δ)
**1**	**1.0000**	**1.0000**	**1.0000**	**1.0000**	**1.0000**	**1.0000**	**1.0000**	**1.0000**	**1.0000**
2	1.0000	1.0000	1.0000	1.0000	1.0000	1.0000	1.0000	1.0000	1.0000
3	0.9999	0.9999	1.0000	0.9999	0.9999	1.0000	0.9999	0.9999	1.0000
4	0.9861	1.0000	0.9861	0.9705	1.0000	0.9705	0.9666	0.9993	0.9673
5	0.9597	0.9597	1.0000	0.9800	0.9800	1.0000	0.9995	0.9995	1.0000
6	0.9334	0.9914	0.9420	0.9237	0.9923	0.9314	0.9073	0.9899	0.9174
7	0.9093	0.9842	0.9251	0.8998	0.9853	0.9145	0.8888	0.9828	0.9060
8	0.8968	0.8969	1.0000	0.7702	0.7703	0.9999	0.7173	0.7174	0.9999
9	0.8923	0.8923	1.0000	0.7178	0.7178	1.0000	0.5187	0.5187	1.0000
10	0.8858	0.9859	0.9000	0.8844	0.9989	0.8855	0.8711	0.9972	0.8739
11	0.8794	0.8794	1.0000	0.6545	0.6545	1.0000	0.5328	0.5328	1.0000
12	0.8658	0.9965	0.8693	0.8688	0.9966	0.8722	0.8454	0.9923	0.8531
13	0.8373	0.9693	0.8680	0.8193	0.9712	0.8482	0.8162	0.9694	0.8468
14	0.8353	0.9549	0.8804	0.8212	0.9570	0.8642	0.8023	0.9508	0.8514
15	0.8293	0.9629	0.8664	0.8095	0.9632	0.8462	0.8037	0.9595	0.8442
16	0.8191	0.9141	0.9050	0.8104	0.9188	0.8915	0.8054	0.9131	0.8923
17	0.7994	0.9128	0.8866	0.7954	0.9182	0.8772	0.7807	0.9106	0.8701
18	0.7604	0.7604	1.0000	0.7647	0.7647	1.0000	0.7494	0.7494	1.0000
19	0.7586	0.7586	1.0000	0.7936	0.7936	1.0000	0.7028	0.7028	1.0000
20	0.7571	0.8915	0.8656	0.7866	0.9500	0.8367	0.8105	0.9758	0.8347
21	0.7432	0.7519	0.9914	0.5436	0.5601	0.9835	0.3272	0.3377	0.9895
22	0.7350	0.8937	0.8413	0.6586	0.8317	0.8269	0.6340	0.8204	0.8136
23	0.7224	0.9712	0.7512	0.7092	0.9473	0.7619	0.6675	0.9151	0.7524
24	0.7071	0.9970	0.7102	0.6822	0.9957	0.6865	0.6688	0.9898	0.6790
25	0.7038	0.9409	0.7628	0.6638	0.9425	0.7213	0.6680	0.9416	0.7264
26	0.6999	0.8279	0.8720	0.6089	0.7400	0.8689	0.4872	0.6094	0.8778
27	0.6344	0.8992	0.7351	0.7475	0.8842	0.8634	0.8139	0.9146	0.8992
28	0.6089	0.9484	0.6605	0.6000	0.9444	0.6556	0.5886	0.9603	0.6284
29	0.5774	0.8163	0.7611	0.5310	0.8189	0.7121	0.5148	0.8074	0.7074
30	0.4381	0.7994	0.6387	0.4214	0.7753	0.6461	0.3837	0.7056	0.6782
31	0.4341	0.8346	0.5995	0.5858	0.9139	0.6719	0.5478	0.9484	0.5995
32	0.3699	0.6269	0.7430	-0.5171	-0.2142	0.6971	-1.5435	-1.1850	0.6415
33	0.2977	0.4356	0.8621	0.2558	0.4108	0.8451	0.2245	0.3573	0.8672
34	0.2749	0.9009	0.3740	0.0684	0.8227	0.2457	0.0983	0.7873	0.3110
35	0.2276	0.3387	0.8889	0.1845	0.3066	0.8780	0.1548	0.2621	0.8927
36	0.1221	0.3178	0.8043	0.4432	0.5836	0.8596	0.3870	0.5382	0.8488
37	0.0190	0.8802	0.1388	-0.9292	0.9432	-0.8724	-1.0935	0.9658	-1.0593
38	-0.0242	0.5243	0.4516	0.1368	0.6937	0.4431	0.1564	0.7249	0.4315
39	-0.1434	0.9703	-0.1136	-0.3002	0.9701	-0.2703	-0.1530	0.9738	-0.1268
40	-0.2234	0.7330	0.0436	-0.3544	0.7256	-0.0800	-0.3668	0.6415	-0.0083
41	-0.2982	-0.2982	1.0000	0.6373	0.6373	1.0000	0.9094	0.9094	1.0000
42	-0.6112	0.0074	0.3814	-1.0465	-0.2306	0.1841	-1.3082	-0.5466	0.2384
43	-0.6344	0.5472	-0.1816	-0.9257	0.5441	-0.4699	-0.8996	0.4421	-0.3417
44	-0.6961	0.4170	-0.1131	-1.2588	0.7664	-1.0252	-1.1406	0.9319	-1.0725
45	-0.7182	0.6013	-0.3195	-0.9234	0.5905	-0.5139	-0.9147	0.5071	-0.4219
46	-0.7665	-0.2818	0.5153	-1.1239	-0.2910	0.1671	-1.2870	-0.4815	0.1944
47	-1.1627	-1.1627	1.0000	-1.3766	-1.3766	1.0000	-1.4210	-1.4210	1.0000
48	-1.1657	0.6401	-0.8058	-1.0470	0.7881	-0.8351	-0.8750	0.8052	-0.6802
49	-1.2193	0.1019	-0.3212	-1.3439	0.0991	-0.4430	-1.2635	-0.0023	-0.2611
50	-1.3176	0.0592	-0.3767	-1.7105	0.0486	-0.7592	-1.7828	-0.1229	-0.6600
51	-1.3722	0.3045	-0.6767	-1.6031	0.3059	-0.9091	-1.4480	0.2977	-0.7457
52	-1.4226	0.2066	-0.6293	-1.5918	0.2262	-0.8180	-1.4961	0.2165	-0.7126
53	-2.1619	0.5458	-1.7078	-3.9554	-0.5186	-2.4368	-6.4988	-2.8332	-2.6656
54	-2.2500	-2.2500	1.0000	-4.6442	-4.6442	1.0000	-6.8179	-6.8179	1.0000
55	-2.7079	-1.4084	-0.2995	-2.8324	-1.4186	-0.4139	-2.7514	-1.4321	-0.3193
56	-2.9685	0.6612	-2.6297	-4.8256	0.5903	-4.4158	-4.3252	0.5412	-3.8664
57	-2.9934	0.6072	-2.6006	-4.6261	0.5383	-4.1644	-4.0997	0.4755	-3.5752
58	-3.0478	-1.3713	-0.6765	-3.1854	-1.3402	-0.8452	-3.1392	-1.4053	-0.7339
59	-3.1844	-1.1816	-1.0028	-3.3900	-1.1937	-1.1962	-3.4191	-1.3618	-1.0573
60	-3.2855	-2.0737	-0.2118	-3.3708	-1.9553	-0.4156	-3.4017	-2.0389	-0.3629
61	-3.5394	-0.4943	-2.0451	-3.8401	-0.6446	-2.1955	-3.7572	-0.7011	-2.0561
62	-3.7225	0.0591	-2.7815	-4.0332	0.1530	-3.1861	-3.7946	0.0241	-2.8187
**63**	**-4.0000**	**-4.0000**	**1.0000**	**-4.0000**	**-4.0000**	**1.0000**	**-4.0000**	**-4.0000**	**1.0000**
64	-4.2335	-4.2324	0.9989	-4.2655	-4.2644	0.9989	-4.2388	-4.2378	0.9990
65	-4.3477	0.8701	-4.2178	-7.8529	0.8600	-7.7129	-6.6868	0.8278	-6.5146
66	-4.4241	-4.4241	1.0000	-4.2025	-4.2025	1.0000	-4.3294	-4.3294	1.0000
67	-4.4261	-4.4242	0.9982	-4.4686	-4.4671	0.9985	-4.4320	-4.4306	0.9986
68	-4.5036	-4.4927	0.9891	-4.4804	-4.4718	0.9915	-4.4491	-4.4416	0.9925
69	-4.6770	-0.7521	-2.9250	-6.2841	-0.8652	-4.4190	-5.7796	-0.8996	-3.8800
70	-4.7215	-4.7034	0.9819	-4.7060	-4.6914	0.9854	-4.7429	-4.7306	0.9876
71	-5.3762	-5.3762	1.0000	-5.3249	-5.3249	1.0000	-5.3892	-5.3892	1.0000
72	-5.3918	-5.3918	1.0000	-5.3934	-5.3934	1.0000	-5.4849	-5.4849	1.0000
73	-5.7345	-0.9986	-3.7359	-8.3476	-0.9007	-6.4469	-8.0213	-0.7800	-6.2413

Table S6. (continued)

mol	HOMA(ε)	EN(ε)	GEO(ε)	HOMA(V)	EN(V)	GEO(V)	HOMA(G)	EN(G)	GEO(G)
1	1.0000	1.0000	1.0000	1.0000	1.0000	1.0000	1.0000	1.0000	1.0000
2	0.9984	0.9984	1.0000	0.9999	1.0000	1.0000	0.9999	0.9999	1.0000
3	0.9988	0.9993	0.9995	0.9999	0.9999	1.0000	0.9999	1.0000	1.0000
4	0.9966	0.9970	0.9996	0.9707	0.9999	0.9709	0.9709	0.9968	0.9741
5	-0.3170	-0.3170	1.0000	0.9345	0.9346	1.0000	0.7140	0.7140	1.0000
6	0.9493	0.9952	0.9542	0.8985	0.9901	0.9084	0.8848	0.9904	0.8945
7	0.9020	0.9884	0.9136	0.8620	0.9803	0.8817	0.8242	0.9771	0.8471
8	0.9407	0.9407	1.0000	0.7944	0.7945	0.9999	0.8704	0.8704	0.9999
9	0.7242	0.7242	1.0000	0.8045	0.8045	1.0000	0.9798	0.9798	1.0000
10	0.3094	0.4895	0.8199	0.8201	0.9877	0.8324	0.6767	0.9059	0.7708
11	0.5833	0.5833	1.0000	0.7441	0.7441	1.0000	0.9136	0.9136	1.0000
12	0.8696	0.9975	0.8721	0.8029	0.9989	0.8040	0.7342	0.9990	0.7352
13	0.7642	0.9707	0.7935	0.7473	0.9616	0.7857	0.6493	0.9510	0.6983
14	0.8360	0.9667	0.8694	0.7595	0.9431	0.8165	0.6986	0.9330	0.7656
15	0.7703	0.9650	0.8053	0.7371	0.9530	0.7841	0.6431	0.9446	0.6985
16	0.7492	0.9104	0.8388	0.7286	0.8855	0.8431	0.6195	0.8476	0.7720
17	0.7967	0.9170	0.8797	0.7336	0.8850	0.8486	0.6711	0.8502	0.8209
18	0.7158	0.7158	1.0000	0.6629	0.6629	1.0000	0.5430	0.5430	1.0000
19	-0.8624	-0.8624	1.0000	0.8107	0.8108	1.0000	0.9078	0.9078	1.0000
20	0.0407	0.2713	0.7695	0.6815	0.8888	0.7927	0.4345	0.7021	0.7325
21	0.9097	0.9831	0.9266	0.6096	0.6231	0.9865	0.8412	0.8590	0.9823
22	0.8050	0.9897	0.8153	0.6198	0.8283	0.7914	0.5896	0.8375	0.7521
23	0.5945	0.9831	0.6114	0.6109	0.9682	0.6427	0.4826	0.9981	0.4844
24	0.5627	0.9871	0.5755	0.5307	0.9999	0.5308	0.3021	0.9900	0.3121
25	0.5594	0.9463	0.6131	0.5399	0.9287	0.6112	0.3573	0.9115	0.4458
26	0.8346	0.9956	0.8390	0.6057	0.7611	0.8446	0.6933	0.8936	0.7997
27	-0.7966	0.0781	0.1253	0.5461	0.9124	0.6337	0.0098	0.9096	0.1002
28	0.6646	0.9100	0.7546	0.5564	0.9361	0.6203	0.5086	0.8996	0.6090
29	0.5235	0.8556	0.6680	0.3734	0.7674	0.6060	0.1754	0.7149	0.4605
30	0.5390	0.9432	0.5957	0.3650	0.7737	0.5913	0.3174	0.8433	0.4741
31	-0.4164	-0.0945	0.6781	0.3945	0.8860	0.5085	0.1626	0.7797	0.3829
32	-0.5354	0.3612	0.1035	0.4015	0.6440	0.7576	0.6559	0.7929	0.8630
33	0.4843	0.6468	0.8375	0.0788	0.2614	0.8175	-0.1140	0.1382	0.7477
34	-0.2620	0.8704	-0.1324	-0.0661	0.8989	0.0350	-0.3779	0.9783	-0.3562
35	0.4019	0.5062	0.8958	-0.0075	0.1268	0.8656	-0.2201	-0.0495	0.8294
36	-1.0897	-0.9638	0.8740	0.1778	0.3550	0.8229	-0.1146	0.0964	0.7890
37	0.1451	0.8479	0.2972	-2.1414	0.6728	-1.8142	-4.0097	-0.0688	-2.9409
38	-0.6641	-0.2402	0.5761	-0.1381	0.6039	0.2580	-0.5496	0.4308	0.0195
39	-1.6919	0.9975	-1.6894	-0.8241	0.9931	-0.8171	-1.8557	0.9999	-1.8556
40	-0.4652	0.9461	-0.4113	-0.6171	0.7808	-0.3979	-1.0472	0.9024	-0.9496
41	-0.0871	-0.0871	1.0000	-4.4093	-4.4093	1.0000	-28.8096	-28.8096	1.0000
42	-0.4757	0.7103	-0.1860	-0.8617	-0.0659	0.2042	-0.5017	0.3909	0.1074
43	-1.0445	0.8336	-0.8781	-1.0795	0.6248	-0.7043	-1.4035	0.7955	-1.1990
44	-1.0401	-0.1953	0.1552	-3.7783	-0.4571	-2.3212	-9.6947	-4.4563	-4.2385
45	-1.2081	0.8820	-1.0901	-1.2146	0.6820	-0.8966	-1.7145	0.8408	-1.5552
46	-0.6037	-0.1228	0.5191	-0.9711	-0.3232	0.3521	-0.6283	-0.1461	0.5179
47	-0.3448	-0.3449	1.0000	-1.6435	-1.6435	1.0000	-1.9218	-1.9218	1.0000
48	-0.7310	0.7600	-0.4909	-1.3442	0.8979	-1.2421	-2.0546	0.9696	-2.0242
49	-1.8984	0.6325	-1.5310	-1.6385	0.1708	-0.8093	-2.2382	0.3560	-1.5941
50	-1.5216	0.5879	-1.1095	-1.5996	0.2217	-0.8213	-1.5394	0.5503	-1.0897
51	-2.4175	0.6716	-2.0891	-2.0794	0.4212	-1.5005	-3.0253	0.5528	-2.5781
52	-1.9798	0.5149	-1.4947	-1.9140	0.3214	-1.2354	-2.5283	0.4367	-1.9650
53	-19.2441	-12.3975	-5.8465	-3.1075	0.8906	-2.9981	-6.0076	-1.5940	-3.4136
54	-13.6290	-13.6290	1.0000	-2.3031	-2.3031	1.0000	0.5777	0.5777	1.0000
55	-3.1765	-0.9955	-1.1810	-3.0448	-1.2986	-0.7462	-3.4851	-1.1443	-1.3408
56	-2.1981	-0.1001	-1.0980	-7.0097	0.9597	-6.9694	-11.8787	0.8646	-11.7433
57	-2.2314	-0.2660	-0.9654	-6.8492	0.9473	-6.7966	-12.0232	0.8698	-11.8930
58	-2.9983	-0.6076	-1.3908	-3.2927	-1.0949	-1.1978	-3.5846	-0.7526	-1.8320
59	-2.6292	0.0713	-1.7005	-3.2219	-0.7577	-1.4642	-3.1572	-0.1536	-2.0036
60	-2.0702	-1.2704	0.2002	-2.9680	-1.7405	-0.2275	-2.4806	-1.4046	-0.0760
61	-1.6279	-0.0572	-0.5707	-3.9339	-0.0271	-2.9068	-4.5042	0.5558	-4.0600
62	-3.8924	0.9984	-3.8908	-3.8018	0.6330	-3.4348	-4.2525	0.9775	-4.2300
63	-4.0000	-4.0000	1.0000	-4.0000	-4.0000	1.0000	-4.0000	-4.0000	1.0000
64	-3.8841	-3.8823	0.9982	-4.2052	-4.2046	0.9993	-4.1654	-4.1650	0.9996
65	-2.0523	-1.4924	0.4401	-11.9816	0.8864	-11.8680	-21.6515	-0.5239	-20.1276
66	-4.1669	-4.1669	1.0000	-4.1937	-4.1937	1.0000	-4.0341	-4.0341	1.0000
67	-3.8236	-3.8191	0.9955	-4.3549	-4.3539	0.9990	-4.2638	-4.2632	0.9994
68	-3.9372	-3.9371	0.9998	-4.3708	-4.3649	0.9941	-4.2783	-4.2742	0.9958
69	-2.9057	-2.5622	0.6565	-8.0483	0.1045	-7.1528	-12.3615	0.8143	-12.1758
70	-3.9972	-3.9969	0.9997	-4.4860	-4.4765	0.9905	-4.1875	-4.1809	0.9934
71	-3.6569	-3.6569	1.0000	-4.8336	-4.8336	1.0000	-4.2038	-4.2038	1.0000
72	-3.5560	-3.5560	1.0000	-4.8469	-4.8469	1.0000	-4.1289	-4.1289	1.0000
73	-2.2103	-1.7451	0.5347	-9.9128	0.2369	-9.1497	-13.3800	0.8950	-13.2750

Table S6. (continued)

mol	HOMA(K)	EN(K)	GEO(K)	HOMA(ESP)	EN(ESP)	GEO(ESP)
1	1.0000	1.0000	1.0000	1.0000	1.0000	1.0000
2	1.0000	1.0000	1.0000	0.0542	0.0542	1.0000
3	0.9999	0.9999	1.0000	-1.5518	-1.5517	0.9999
4	0.9697	1.0000	0.9697	0.9785	0.9977	0.9808
5	0.9699	0.9699	1.0000	-10.8565	-10.8565	1.0000
6	0.9018	0.9900	0.9118	0.9464	0.9972	0.9492
7	0.8719	0.9812	0.8908	0.9232	0.9920	0.9312
8	0.7686	0.7687	0.9999	-6.4754	-6.4754	1.0000
9	0.7182	0.7182	1.0000	-4.3936	-4.3936	1.0000
10	0.8453	0.9972	0.8481	-9.7317	-9.6077	0.8761
11	0.6769	0.6769	1.0000	-10.6810	-10.6810	1.0000
12	0.8194	0.9972	0.8222	0.8237	0.9999	0.8239
13	0.7730	0.9644	0.8085	0.8542	0.9802	0.8740
14	0.7755	0.9459	0.8296	0.8670	0.9754	0.8915
15	0.7618	0.9554	0.8065	-1.7164	-1.5919	0.8755
16	0.7572	0.8956	0.8616	0.8619	0.9519	0.9100
17	0.7507	0.8944	0.8564	0.8447	0.9448	0.9000
18	0.6949	0.6949	1.0000	0.8460	0.8460	1.0000
19	0.7755	0.7755	1.0000	-20.7878	-20.7878	1.0000
20	0.7350	0.9267	0.8084	-8.6419	-8.5411	0.8991
21	0.5193	0.5316	0.9876	-8.1398	-8.1179	0.9781
22	0.6259	0.8255	0.8004	-4.2868	-4.1106	0.8238
23	0.6362	0.9524	0.6837	-3.4216	-3.1263	0.7047
24	0.5849	0.9983	0.5867	0.6393	0.9998	0.6394
25	0.5877	0.9334	0.6543	0.7384	0.9635	0.7748
26	0.5687	0.7119	0.8567	-5.2357	-5.1349	0.8992
27	0.6593	0.9132	0.7462	-16.1817	-15.8496	0.6679
28	0.5686	0.9452	0.6233	0.6682	0.9914	0.6768
29	0.4258	0.7819	0.6439	0.6437	0.8719	0.7718
30	0.3738	0.7507	0.6231	0.5273	0.7863	0.7410
31	0.4528	0.9108	0.5420	-11.4780	-10.9757	0.4977
32	-0.1036	0.1769	0.7195	0.6696	0.9066	0.7630
33	0.1318	0.2959	0.8359	0.4717	0.5827	0.8890
34	0.0019	0.8643	0.1376	-3.0395	-2.4255	0.3860
35	0.0513	0.1758	0.8755	0.4297	0.5149	0.9148
36	0.2553	0.4230	0.8323	-8.9241	-8.7212	0.7971
37	-1.7183	0.8102	-1.5285	-0.6658	0.4689	-0.1347
38	-0.0291	0.6491	0.3219	-7.9273	-7.3785	0.4512
39	-0.5675	0.9877	-0.5552	-0.1084	0.9946	-0.1030
40	-0.5174	0.7356	-0.2530	-0.1325	0.8027	0.0648
41	-0.9608	-0.9608	1.0000	-13.7273	-13.7273	1.0000
42	-1.0040	-0.2251	0.2211	-7.2697	-6.7888	0.5191
43	-1.0066	0.5645	-0.5711	-0.4556	0.5375	0.0070
44	-2.6074	0.2366	-1.8440	-3.7592	-1.7302	-1.0290
45	-1.0965	0.6248	-0.7213	-0.5978	0.6237	-0.2215
46	-1.0794	-0.3779	0.2985	-0.2593	0.0307	0.7100
47	-1.5640	-1.5640	1.0000	-1.2750	-1.2750	1.0000
48	-1.1658	0.8686	-1.0344	-4.3055	-2.7365	-0.5690
49	-1.4931	0.1117	-0.6048	-1.1837	0.0162	-0.2000
50	-1.6503	0.1075	-0.7578	-0.7348	0.4094	-0.1443
51	-1.8401	0.3790	-1.2192	-1.2063	0.3859	-0.5922
52	-1.7567	0.2853	-1.0420	-1.2164	0.2937	-0.5101
53	-3.6966	0.1827	-2.8793	-1.2827	0.9999	-1.2826
54	-3.6754	-3.6754	1.0000	-0.7940	-0.7940	1.0000
55	-2.9336	-1.3452	-0.5883	-2.5549	-1.3295	-0.2254
56	-5.9185	0.8641	-5.7826	-2.6918	0.9758	-2.6675
57	-5.7154	0.8369	-5.5523	-3.1725	0.9849	-3.1574
58	-3.2292	-1.2019	-1.0273	-2.7285	-1.1927	-0.5358
59	-3.2765	-0.9604	-1.3162	-2.5785	-0.8630	-0.7156
60	-3.1182	-1.8439	-0.2744	-2.4153	-1.5865	0.1712
61	-3.8403	-0.2453	-2.5950	-2.9246	0.1297	-2.0543
62	-3.7607	0.4518	-3.2125	-2.5507	0.5331	-2.0838
63	-4.0000	-4.0000	1.0000	-4.0000	-4.0000	1.0000
64	-4.2171	-4.2163	0.9992	-4.2113	-4.2106	0.9993
65	-9.8237	0.9948	-9.8185	-4.1962	0.8670	-4.0632
66	-4.2413	-4.2413	1.0000	-4.3683	-4.3683	1.0000
67	-4.3820	-4.3809	0.9989	-4.3688	-4.3678	0.9990
68	-4.3983	-4.3919	0.9936	-4.3780	-4.3714	0.9933
69	-7.1089	-0.2067	-5.9022	-3.9902	0.1819	-3.1721
70	-4.5758	-4.5654	0.9895	-4.4898	-4.4792	0.9894
71	-5.0265	-5.0265	1.0000	-4.7714	-4.7714	1.0000
72	-5.0679	-5.0679	1.0000	-17.3429	-17.3429	1.0000
73	-9.1408	-0.0730	-8.0679	-4.4482	0.2428	-3.6910

Table S6. (continued)

mol	HOMA(Rho/RCP)	EN(Rho/RCP)	GEO(Rho/RCP)	HOMA(Δ/RCP)	EN(Δ/RCP)	GEO(Δ/RCP)
1	1.0000	1.0000	1.0000	1.0000	1.0000	1.0000
2	0.9616	0.9616	1.0000	0.9984	0.9984	1.0000
3	0.9999	0.9999	1.0000	0.9998	0.9998	1.0000
4	0.9384	0.9384	1.0000	0.9937	0.9937	1.0000
5	-113.7169	-113.7169	1.0000	-42.3971	-42.3971	1.0000
6	0.9333	0.9333	1.0000	0.9834	0.9834	1.0000
7	0.8710	0.8710	1.0000	0.9629	0.9629	1.0000
8	-62.2371	-62.2371	1.0000	-22.1236	-22.1236	1.0000
9	-193.2150	-193.2150	1.0000	-49.8061	-49.8061	1.0000
10	-55.3612	-55.3612	1.0000	-18.9807	-18.9807	1.0000
11	-117.9208	-117.9208	1.0000	-44.6135	-44.6135	1.0000
12	0.9832	0.9832	1.0000	1.0000	1.0000	1.0000
13	0.7563	0.7563	1.0000	0.9190	0.9190	1.0000
14	0.6343	0.6343	1.0000	0.8852	0.8852	1.0000
15	0.7537	0.7537	1.0000	0.9006	0.9006	1.0000
16	0.2978	0.2978	1.0000	0.7949	0.7949	1.0000
17	0.3596	0.3596	1.0000	0.7684	0.7684	1.0000
18	-0.7903	-0.7903	1.0000	0.4255	0.4255	1.0000
19	-2916.6509	-2916.6509	1.0000	-484.7065	-484.7065	1.0000
20	0.5446	0.5446	1.0000	0.8761	0.8761	1.0000
21	-176.3011	-176.3011	1.0000	-45.7934	-45.7934	1.0000
22	-60.5415	-60.5415	1.0000	-21.6119	-21.6119	1.0000
23	0.9528	0.9528	1.0000	0.9299	0.9299	1.0000
24	0.9796	0.9796	1.0000	1.0000	1.0000	1.0000
25	0.5345	0.5345	1.0000	0.8412	0.8412	1.0000
26	-185.6622	-185.6622	1.0000	-50.8798	-50.8798	1.0000
27	-5.4868	-5.4868	1.0000	0.7237	0.7237	1.0000
28	-58.6635	-58.6635	1.0000	-20.4906	-20.4906	1.0000
29	-0.4067	-0.4067	1.0000	0.5096	0.5096	1.0000
30	-185.8220	-185.8220	1.0000	-51.3081	-51.3081	1.0000
31	0.9086	0.9086	1.0000	0.9698	0.9698	1.0000
32	-3972.2788	-3972.2788	1.0000	-257.2586	-257.2586	1.0000
33	-3.1746	-3.1746	1.0000	-0.4586	-0.4586	1.0000
34	-50.9663	-50.9663	1.0000	-19.5466	-19.5466	1.0000
35	-3.7143	-3.7143	1.0000	-0.6220	-0.6220	1.0000
36	-1835.3805	-1835.3805	1.0000	-408.4244	-408.4244	1.0000
37	-12.3930	-12.3930	1.0000	-0.4362	-0.4362	1.0000
38	-172.2629	-172.2629	1.0000	-49.0447	-49.0447	1.0000
39	-75.4379	-75.4379	1.0000	-35.2776	-35.2776	1.0000
40	-181.5757	-181.5757	1.0000	-48.8095	-48.8095	1.0000
41	-448.6260	-448.6260	1.0000	-27.5753	-27.5753	1.0000
42	-1594.3276	-1594.3276	1.0000	-298.3149	-298.3149	1.0000
43	-169.8096	-169.8096	1.0000	-41.5154	-41.5154	1.0000
44	-20.0349	-20.0349	1.0000	-5.7278	-5.7278	1.0000
45	-174.1285	-174.1285	1.0000	-42.1946	-42.1946	1.0000
46	-1524.2258	-1524.2258	1.0000	-293.4873	-293.4873	1.0000
47	-11.7818	-11.7818	1.0000	-4.2097	-4.2097	1.0000
48	-186.3890	-186.3890	1.0000	-51.4038	-51.4038	1.0000
49	-1.9114	-1.9114	1.0000	-0.6387	-0.6387	1.0000
50	-1607.2155	-1607.2155	1.0000	-310.2018	-310.2018	1.0000
51	-1.8357	-1.8357	1.0000	-0.4042	-0.4042	1.0000
52	-0.2158	-0.2158	1.0000	0.0992	0.0992	1.0000
53	-10183.4385	-10183.4385	1.0000	-4.4880	-4.4880	1.0000
54	-8534.5771	-8534.5771	1.0000	1.0000	1.0000	1.0000
55	-2.0601	-2.0601	1.0000	-1.4368	-1.4368	1.0000
56	-2.9370	-2.9370	1.0000	0.9653	0.9653	1.0000
57	-2.6250	-2.6250	1.0000	0.9400	0.9400	1.0000
58	-111.0125	-111.0125	1.0000	-17.3144	-17.3144	1.0000
59	-1438.4303	-1438.4303	1.0000	-185.8721	-185.8721	1.0000
60	-1285.3577	-1285.3577	1.0000	-161.1513	-161.1513	1.0000
61	-0.0671	-0.0671	1.0000	0.8597	0.8597	1.0000
62	-1535.2446	-1535.2446	1.0000	-276.2120	-276.2120	1.0000
63	-4.0000	-4.0000	1.0000	-4.0000	-4.0000	1.0000
64	-57.8209	-57.8209	1.0000	-23.7929	-23.7929	1.0000
65	-45.8509	-45.8509	1.0000	-0.4946	-0.4946	1.0000
66	-2.4196	-2.4196	1.0000	-3.1477	-3.1477	1.0000
67	-99.3218	-99.3218	1.0000	-38.9864	-38.9864	1.0000
68	-1.5617	-1.5617	1.0000	-2.0104	-2.0104	1.0000
69	-1.5261	-1.5261	1.0000	0.5798	0.5798	1.0000
70	-83.8234	-83.8234	1.0000	-6.2805	-6.2805	1.0000
71	-1112.0547	-1112.0547	1.0000	-85.1486	-85.1486	1.0000
72	-1131.9274	-1131.9274	1.0000	-84.2329	-84.2329	1.0000
73	-171.3688	-171.3688	1.0000	-17.1642	-17.1642	1.0000

Table S6. (continued)

mol	HOMA(σ(^{13}C))	EN(σ(^{13}C))	GEO(σ(^{13}C))	HOMA(^{1}J(CC))	EN(^{1}J(CC))	GEO(^{1}J(CC))	INICS
1	1.0000	1.0000	1.0000	1.0000	1.0000	1.0000	-146.4
2	-1.2662	-1.0940	0.8278	-0.3320	0.9198	-0.2518	2981.9
3	0.9988	0.9988	1.0000	-140.2904	-44.1701	-95.1203	-196.7
4	0.9397	0.9941	0.9456	0.6041	0.9906	0.6135	-141.1
5	0.1373	0.1373	1.0000	0.7850	0.7850	1.0000	-151.7
6	0.9929	0.9988	0.9941	0.9567	0.9930	0.9637	-142.1
7	0.9927	0.9999	0.9927	0.9511	0.9991	0.9520	-156.4
8	0.0383	0.2079	0.8304	-6688.1614	-4162.0039	-2525.1575	2563.9
9	0.5458	0.5458	1.0000	0.9043	0.9043	1.0000	-157.7
10	0.3087	0.3822	0.9265	0.5895	0.9930	0.5965	25.6
11	0.2444	0.2444	1.0000	-0.0031	-0.0031	1.0000	-236.2
12	0.8652	0.9897	0.8755	0.3414	0.9969	0.3445	-61.0
13	0.9912	0.9993	0.9919	0.8961	0.9997	0.8964	-156.9
14	0.9839	0.9998	0.9841	0.8976	0.9970	0.9005	-118.4
15	-0.3250	0.5937	0.0812	-76.2700	-22.1009	-53.1690	1200.0
16	0.9943	0.9979	0.9964	0.9201	0.9952	0.9248	-202.1
17	0.9926	0.9972	0.9954	0.8064	0.9990	0.8074	-193.0
18	0.9870	0.9870	1.0000	0.9201	0.9952	0.9248	-134.8
19	-0.3545	-0.3545	1.0000	-6.4511	-6.4511	1.0000	-113.9
20	0.0085	0.2367	0.7718	0.5718	0.8862	0.6857	289.1
21	0.0795	0.5276	0.5519	0.3542	0.8771	0.4771	-157.0
22	0.7809	0.9897	0.7912	-3.3402	-2.3741	0.0339	699.0
23	0.3114	0.4637	0.8477	-0.1525	0.8096	0.0379	-67.8
24	0.8374	0.9979	0.8396	-0.1173	0.9243	-0.0416	-30.4
25	0.9948	0.9998	0.9950	0.8108	0.9999	0.8109	-147.0
26	0.5681	0.9115	0.6565	0.4936	0.5475	0.9460	242.0
27	-3.7959	-3.6315	0.8355	0.3279	0.3459	0.9820	101.3
28	0.9514	0.9843	0.9671	0.6717	0.9846	0.6871	-133.4
29	0.9933	0.9969	0.9964	0.8663	0.9889	0.8774	-128.7
30	0.9311	0.9968	0.9342	0.6720	0.9829	0.6891	-233.0
31	-0.0281	0.1581	0.8138	-0.2663	0.4660	0.2677	-102.8
32	-0.9290	0.6134	-0.5423	-12.6099	-12.0405	0.4305	-187.1
33	0.9959	0.9959	1.0000	0.9450	0.9512	0.9938	-89.4
34	0.4426	0.9223	0.5203	-5.5900	-3.3609	-1.2291	707.7
35	0.9902	0.9911	0.9992	0.9428	0.9431	0.9997	21.7
36	-1.6890	-1.5471	0.8581	-2.1034	-1.8021	0.6988	-87.4
37	0.3019	0.7338	0.5681	-85.1116	-25.0629	-59.0487	-167.6
38	-1.4276	-0.7411	0.3135	0.6857	0.8370	0.8487	301.2
39	0.9689	0.9689	1.0000	-0.3868	0.4380	0.1752	40.2
40	0.8404	0.8856	0.9548	-3.9759	0.7641	-3.7399	253.1
41	0.1155	0.7063	0.4092	-1.6223	-1.6223	1.0000	-142.0
42	0.0583	0.4023	0.6559	0.2808	0.8200	0.4608	-229.2
43	-0.0071	0.9867	0.0061	-0.3823	0.6659	-0.0482	-47.9
44	-0.5781	-0.0232	0.4451	-209.4163	-42.6112	-165.8051	nd
45	0.2560	0.9281	0.3279	-0.6043	0.8501	-0.4544	-62.5
46	0.6708	0.6708	1.0000	-0.3027	-0.0995	0.7968	156.1
47	0.9856	0.9856	1.0000	0.9840	0.9840	1.0000	3.0
48	-2.3706	-0.9663	-0.4043	-2.5090	-1.1876	-0.3213	838.6
49	-0.5889	0.3818	0.0293	-1.6940	0.9767	-1.6707	16.9
50	0.7330	0.7362	0.9968	-0.2481	0.7966	-0.0447	213.0
51	-0.6591	0.4124	-0.0715	-1.2269	0.7379	-0.9648	10.4
52	-0.8071	0.3818	-0.1889	-1.0341	0.7229	-0.7570	7.1
53	-1.8028	-0.4524	-0.3504	-10.3924	-5.0918	-4.3006	nd
54	-7.7478	-7.7478	1.0000	-13.8316	-13.8316	1.0000	-122.7
55	-2.5180	-1.3090	-0.2090	-2.6070	-0.9839	-0.6230	1.2
56	-0.2019	0.8048	-0.0067	-42.7509	-5.1935	-36.5574	-35.4
57	-0.4879	0.8998	-0.3877	-51.3232	-7.5686	-42.7546	-5.5
58	-1.8514	-0.5966	-0.2547	-2.4928	-0.7361	-0.7567	-17.8
59	-1.3671	0.0806	-0.4476	-3.4872	-2.1205	-0.3666	22.4
60	-1.5465	0.1712	-0.7177	-3.0349	-2.8630	0.8281	19.0
61	-2.1380	0.1308	-1.2688	-5.4124	0.9825	-5.3949	-55.2
62	0.9234	0.9234	1.0000	-59.0680	-57.7525	-0.3155	253.3
63	-4.0000	-4.0000	1.0000	-4.0000	-4.0000	1.0000	4.5
64	-3.8503	-3.8458	0.9954	-3.6980	-3.6978	0.9998	-3.3
65	-2.8458	0.9795	-2.8253	-216.0299	-95.6233	-119.4067	223.4
66	-3.3339	-3.3268	0.9928	-4.6895	-4.6895	1.0000	-80.1
67	-4.1575	-4.1477	0.9902	-3.4228	-3.4215	0.9987	-6.7
68	-4.7413	-4.7410	0.9997	-3.8587	-3.8586	0.9998	-5.1
69	-1.7847	-0.4822	-0.3024	-34.6738	-0.6280	-33.0458	-27.5
70	-4.1187	-4.1163	0.9976	-4.1084	-4.1078	0.9994	-24.1
71	-4.5384	-4.5384	1.0000	-5.8385	-5.8385	1.0000	40.8
72	-5.4983	-5.4983	1.0000	-84.8396	-84.8396	1.0000	-0.6
73	-3.3097	0.8618	-3.1715	-24.9522	0.7642	-24.7164	55.1

Table S7. Cartesian xyz coordinates for optimized molecules from Figure 1 calculated using B3LYP, ωB97-XD, and TPSSh methods and the aug-cc-pVTZ basis set.

B3LYP			
1	2	3	4
C -0.000000 1.391257 0.000000	C 0.695269 1.204737 -0.000002	C -0.538208 0.288931 0.000048	C -0.744380 0.694840 0.000143
C -1.204867 0.695628 -0.000000	C 1.390801 0.000168 0.000000	C 0.853739 0.289698 0.000498	C -1.912608 1.433527 0.000275
C -1.204867 -0.695628 0.000000	C 0.695559 -1.204570 0.000002	C 1.550502 1.494671 -0.000083	C -3.103375 0.698645 0.000209
C -0.000000 -1.391257 -0.000000	C -0.695270 -1.204737 0.000002	C 0.854860 2.699679 -0.001030	C -3.103375 -0.698642 -0.000024
C 1.204867 -0.695628 0.000000	C -1.390802 -0.000168 0.000000	C -0.537055 2.698913 -0.001479	C -1.912608 -1.433524 -0.000145
C 1.204867 0.695628 -0.000000	C -0.695561 1.204570 -0.000002	C -1.233835 1.493914 -0.000946	C -0.744380 -0.694836 -0.000098
H 0.000000 2.473100 0.000000	H 1.237567 2.143894 -0.000003	H -1.068876 -0.655585 0.000513	H -1.927577 2.515609 0.000524
H -2.141770 1.236553 -0.000000	H 2.475267 0.000298 0.000000	H 1.395005 -0.647965 0.001287	H -4.050870 1.221322 0.000351
H -2.141770 -1.236553 0.000000	H 1.238084 -2.143596 0.000004	H 2.633809 1.481952 0.000302	H -4.050870 -1.221319 -0.000118
H 0.000000 -2.473100 -0.000000	H -1.237569 -2.143894 0.000004	H 1.385570 3.644172 -0.001472	H -1.927576 -2.515606 -0.000393
H 2.141770 -1.236553 0.000000	H -1.238086 2.143596 -0.000003	H -1.078359 3.636555 -0.002244	C 0.771537 -0.790172 -0.000144
H 2.141770 1.236553 -0.000000	H -2.475268 -0.000298 0.000000	H -2.317141 1.506681 -0.001305	H 1.215804 -1.244196 0.886172
			H 1.216201 -1.244131 -0.886300
			C 0.771537 0.790176 0.000118
			H 1.215770 1.244204 -0.886214
			H 1.216234 1.244132 0.886259
5	6	7	8
C 0.002147 1.851739 0.009783	C -0.000000 1.378333 2.491362	C 0.000000 2.827141 0.875520	C -0.896001 1.357785 0.009220
H 0.015151 2.938051 0.016640	C -0.000000 0.707623 1.252721	C 0.000000 3.548296 -0.295011	C 0.503768 1.541737 -0.002963
C 1.289403 1.298810 -0.081213	C -0.000000 1.438695 -0.013537	C -0.000000 1.417526 0.862511	C 1.525752 0.567684 -0.011821
H 2.063248 2.055448 -0.173699	C -0.000000 2.846738 -0.052013	C 0.000000 2.872120 -1.523522	C -1.623910 0.149087 0.015397
C -1.298087 1.328657 0.092310	C -0.000000 -0.707623 1.252721	C -0.000000 0.726396 -0.379482	C 1.401114 -0.837785 -0.010793
H -2.053922 2.102476 0.191343	C -0.000000 0.731072 -1.239184	C -0.000000 0.676717 2.086271	C -1.123691 -1.170664 0.010782
C -1.851748 0.041394 0.005987	C -0.000000 -0.731072 -1.239184	C 0.000000 1.495329 -1.561018	C 0.218921 -1.608716 -0.000788
H -2.938079 0.054372 0.008515	C -0.000000 -1.438695 -0.013537	C -0.000000 -0.726396 -0.379482	H -1.493397 2.265884 0.014556
C 1.812448 -0.002095 -0.009795	C -0.000000 -1.468411 -2.439353	C -0.000000 -0.676717 2.086271	H 0.841122 2.575834 -0.005864
H 2.898760 -0.015060 -0.016660	C 0.000000 1.468411 -2.439353	C -0.000000 -1.495329 -1.561018	H 2.543688 0.948881 -0.020569
C 1.259492 -1.290075 0.070088	C 0.000000 2.845546 -2.448807	C -0.000000 -1.417526 0.862511	H -2.705957 0.247929 0.024879
H 2.016111 -2.064698 0.155985	C 0.000000 3.543495 -1.239922	C 0.000000 -2.827141 0.875520	H 2.333323 -1.396002 -0.018771
C -0.041413 -1.812470 -0.005956	H -0.000000 4.624985 -1.232088	C -0.000000 -2.872120 -1.523522	H -1.875184 -1.956910 0.017154
H -0.054393 -2.898801 -0.008458	H 0.000000 3.379509 -3.389320	C 0.000000 -3.548296 -0.295011	H 0.362230 -2.686847 -0.002172
C -1.329375 -1.258824 -0.081193	C 0.000000 0.697948 3.688726	H -0.000000 3.337436 1.830381	
H -2.104007 -2.014657 -0.173674	C 0.000000 -0.697948 3.688726	H -0.000000 1.225224 3.019606	
	C 0.000000 -1.378333 2.491362	H 0.000000 1.005823 -2.523291	
	H 0.000000 1.245478 4.621407	H -0.000000 -1.225224 3.019606	
	H 0.000000 -1.245478 4.621407	H 0.000000 -3.337436 1.830381	
	C 0.000000 -2.846738 -0.052013	H 0.000000 -4.629534 -0.270764	
	C -0.000000 -2.845546 -2.448807	H -0.000000 -1.005823 -2.523291	
	H 0.000000 -3.379509 -3.389320	H -0.000000 -3.433746 -2.447960	
	C 0.000000 -3.543495 -1.239922	H 0.000000 3.433746 -2.447960	
	H 0.000000 -4.624985 -1.232088	H 0.000000 4.629534 -0.270764	
	H 0.000000 0.954845 -3.387570		
	H -0.000000 -0.954845 -3.387570		
	H 0.000000 -3.411121 0.866865		
	H 0.000000 -2.456301 2.520713		
	H -0.000000 2.456301 2.520713		
	H -0.000000 3.411121 0.866865		
9	10	11	12
C 0.000000 1.201104 0.000000	C 2.528134 -0.000000 0.000003	C -0.000000 1.846416 0.000000	C 3.111381 -0.692101 -0.000125
H -0.000000 2.284214 0.000000	C 1.924834 1.257752 -0.000003	H -0.000000 2.939788 -0.000000	C 3.111381 0.692105 -0.000090
C 1.142316 0.371161 0.000000	C 0.544911 1.592033 0.000008	C -1.305616 1.305616 0.000000	C 1.908869 1.440237 -0.000031
H 2.172417 0.705861 -0.000000	C 1.924834 -1.257752 0.000010	H -2.078746 2.078746 -0.000000	C 0.753354 0.709398 -0.000011
C 0.705990 -0.971714 -0.000000	C -0.504874 0.709337 0.000008	C 1.305616 1.305616 0.000000	C 0.753354 -0.709394 -0.000046
H 1.342627 -1.847968 -0.000000	C 0.544911 -1.592033 0.000007	H 2.078746 2.078746 -0.000000	C 1.908870 -1.440233 -0.000103
C -1.142316 0.371161 0.000000	C -0.504874 -0.709337 -0.000001	C 1.846416 -0.000000 0.000000	H 4.056452 -1.218059 -0.000169
H -2.172417 0.705861 -0.000000	H 3.612004 -0.000000 0.000006	H 2.939788 -0.000000 -0.000000	H 4.056452 1.218064 -0.000109
C -0.705990 -0.971714 -0.000000	H 2.603230 2.104412 -0.000015	C -1.846416 0.000000 0.000000	H 1.929475 2.521138 -0.000004
H -1.342627 -1.847968 0.000000	H 0.313941 2.651483 -0.000014	H -2.939788 -0.000000 -0.000000	H 1.929476 -2.521134 -0.000130
	H 2.603230 -2.104413 0.000026	C -1.305616 -1.305616 0.000000	C -0.753353 0.709398 0.000043
	H 0.313940 -2.651484 0.000040	H -2.078746 -2.078746 -0.000000	C -1.908869 1.440236 0.000106
	C -1.938050 1.118156 -0.000082	C 0.000000 -1.846416 0.000000	C -3.111380 0.692105 0.000131
	H -2.270946 2.150350 -0.000059	H -0.000000 -2.939788 -0.000000	C -3.111380 -0.692101 0.000095
	C -2.759312 0.000000 -0.000009	C 1.305616 -1.305616 0.000000	C -1.908869 -1.440233 0.000032
	H -3.839227 0.000001 0.000004	H 2.078746 -2.078746 -0.000000	C -0.753353 -0.709394 0.000007
	C -1.938051 -1.118156 0.000047		H -1.929475 2.521138 0.000134
	H -2.270947 -2.150349 0.000091		H -4.056451 1.218063 0.000179
			H -4.056451 -1.218060 0.000117
			H -1.929475 -2.521134 0.000005
13	14	15	16
C 2.423919 -0.705992 -0.000001	C 0.736183 -1.245162 0.000000	C 2.429022 -0.705984 -0.000001	C 1.243935 3.521085 -0.000000
C 1.240442 -1.397422 -0.000000	C -0.736184 1.245162 0.000000	C 1.243764 -1.396103 -0.000000	C -0.000000 2.837968 -0.000000
C 0.000005 -0.714123 0.000000	C 1.434748 -0.000000 -0.000000	C 0.000004 -0.714407 0.000000	C -0.000000 1.422168 -0.000000
C -0.000004 0.714122 -0.000000	C -0.736184 -1.245162 0.000000	C -0.000003 0.714406 -0.000000	C 1.231641 0.711084 -0.000000
C 1.240428 1.397434 -0.000001	C -1.434748 -0.000000 0.000000	C 1.243750 1.396115 -0.000001	C 2.457767 1.418989 0.000000
C 2.423911 0.706016 -0.000001	C 0.736183 1.245162 0.000000	C 2.429015 0.706008 -0.000001	C 2.427387 2.837823 -0.000000
H -1.239400 -2.480140 0.000001	C -2.865224 -0.000000 0.000000	H -1.245872 -2.479819 0.000001	C -1.231641 0.711084 0.000000
H 3.364452 -1.240274 -0.000001	C -3.563410 1.228497 0.000000	H 3.371639 -1.239115 -0.000001	C 1.231641 -0.711084 0.000000
H 1.239426 -2.480127 -0.000000	C -2.875984 2.413141 -0.000000	H 1.245898 -2.479806 0.000000	C -0.000000 -1.422168 0.000000
C -1.240428 -1.397435 0.000001	C -1.474679 2.417829 -0.000001	C -1.243750 -1.396116 0.000001	C -1.231641 -0.711084 0.000000
C -1.240442 1.397423 0.000000	C 1.474679 2.417829 0.000001	C -1.243764 1.396103 0.000000	C -0.000000 -2.837968 -0.000000
H 1.239401 2.480139 -0.000001	C 2.875984 2.413141 0.000000	H 1.245873 2.479819 -0.000001	C 1.243935 -3.521085 -0.000000
H 3.364439 1.240307 -0.000002	C 3.563410 1.228497 -0.000000	H 3.371626 1.239148 -0.000002	C 2.427387 -2.837823 0.000000
C -2.423917 0.705993 0.000000	C 2.865224 -0.000000 -0.000000	C -2.429021 0.705985 0.000001	C 2.457767 -1.418989 0.000000
C -2.423910 -0.706017 0.000001	H -4.645520 1.216080 0.000000	C -2.429014 -0.706009 0.000001	C 3.671329 -0.683264 0.000000
H -1.239423 2.480128 -0.000000	H -3.409232 3.354288 -0.000001	H -1.245896 2.479806 -0.000000	C 3.671329 0.683264 0.000000
H -3.364452 1.240272 0.000001	H -0.975331 3.374416 -0.000001	H -3.371638 1.239114 0.000001	H 4.607820 1.226357 0.000000

H -3.364439 -1.240306 0.000001	H 0.975331 3.374416 0.000002<br>H 3.409232 3.354288 0.000001<br>H 4.645519 1.216080 -0.000000<br>C -3.563409 -1.228498 0.000000<br>H -4.645519 -1.216081 0.000000<br>C -1.474678 -2.417829 -0.000000<br>H -0.975330 -3.374416 -0.000001<br>C -2.875983 -2.413141 -0.000000<br>H -3.409231 -3.354289 -0.000000<br>C 3.563409 -1.228498 -0.000000<br>H 4.645519 -1.216082 -0.000001<br>C 1.474678 -2.417829 0.000001<br>H 0.975329 -3.374416 0.000001<br>C 2.875983 -2.413141 0.000000<br>H 3.409231 -3.354289 0.000000	H -3.371626 -1.239147 0.000001	H 4.607820 -1.226358 0.000000<br>H 1.241841 4.603656 -0.000000<br>H 3.365964 3.377303 -0.000000<br>H 1.241841 -4.603656 -0.000000<br>H 3.365964 -3.377303 0.000000<br>C -1.243935 3.521085 -0.000000<br>C -2.427387 2.837823 0.000000<br>C -2.457767 1.418989 0.000000<br>H -1.241841 4.603656 -0.000000<br>H -3.365964 3.377303 0.000000<br>C -3.671329 0.683264 0.000000<br>C -3.671329 -0.683264 0.000000<br>C -2.457767 -1.418989 0.000000<br>H -4.607820 1.226357 0.000000<br>H -4.607820 -1.226358 0.000000<br>C -1.243935 -3.521085 -0.000000<br>H -1.241841 -4.603656 -0.000000<br>C -2.427387 -2.837823 -0.000000<br>H -3.365964 -3.377303 -0.000000
17<br>C 2.470252 1.401900 -0.000202<br>C 1.218766 0.719954 -0.000094<br>C 3.646052 0.710744 -0.000293<br>C -0.000048 1.398706 0.000001<br>C 1.218766 -0.719955 -0.000083<br>C 3.646052 -0.710745 -0.000284<br>C -0.000048 -1.398706 0.000022<br>C -1.218450 0.720024 0.000106<br>C 2.470252 -1.401901 -0.000182<br>C -2.470302 1.401987 0.000204<br>C -1.218451 -0.720024 0.000117<br>C -2.470302 -1.401987 0.000225<br>C -3.645842 0.710925 0.000305<br>C -3.645842 -0.710925 0.000316<br>H 2.470217 2.484518 -0.000210<br>H 0.000142 2.482240 -0.000007<br>H 0.000142 -2.482241 0.000030<br>H 2.470216 -2.484519 -0.000175<br>H -2.469950 2.484611 0.000195<br>H -4.588277 1.241787 0.000378<br>H -2.469950 -2.484611 0.000233<br>H -4.588277 -1.241787 0.000396<br>H 4.588294 -1.241946 -0.000359<br>H 4.588294 1.241945 -0.000375	18<br>C 1.243935 3.521085 -0.000000<br>C -0.000000 2.837968 -0.000000<br>C -0.000000 1.422168 -0.000000<br>C 1.231641 0.711084 -0.000000<br>C 2.457767 1.418989 0.000000<br>C 2.427387 2.837823 -0.000000<br>C -1.231641 0.711084 0.000000<br>C 1.231641 -0.711084 0.000000<br>C -0.000000 -1.422168 0.000000<br>C -1.231641 -0.711084 0.000000<br>C -0.000000 -2.837968 -0.000000<br>C 1.243935 -3.521085 -0.000000<br>C 2.427387 -2.837823 0.000000<br>C 2.457767 -1.418989 0.000000<br>C 3.671329 -0.683264 0.000000<br>C 3.671329 0.683264 0.000000<br>H 4.607820 1.226357 0.000000<br>H 4.607820 -1.226358 0.000000<br>H 1.241841 4.603656 -0.000000<br>H 3.365964 3.377303 -0.000000<br>H 1.241841 -4.603656 -0.000000<br>H 3.365964 -3.377303 -0.000000<br>C -1.243935 3.521085 -0.000000<br>C -2.427387 2.837823 0.000000<br>C -2.457767 1.418989 0.000000<br>H -1.241841 4.603656 -0.000000<br>H -3.365964 3.377303 0.000000<br>C -3.671329 0.683264 0.000000<br>C -3.671329 -0.683264 0.000000<br>C -2.457767 -1.418989 0.000000<br>H -4.607820 1.226357 0.000000<br>H -4.607820 -1.226358 0.000000<br>C -1.243935 -3.521085 -0.000000<br>H -1.241841 -4.603656 -0.000000<br>C -2.427387 -2.837823 -0.000000<br>H -3.365964 -3.377303 -0.000000	19<br>C 0.682705 0.685622 -0.194036<br>C 0.685544 -0.682802 0.194011<br>H 1.381112 1.386402 -0.664296<br>H 1.386838 -1.380647 0.664338<br>C -0.685543 0.682851 0.193883<br>C -0.682709 -0.685675 -0.193881<br>H -1.386806 1.380757 0.664119<br>H -1.381129 -1.386490 -0.664021	20<br>C 2.469104 -0.684444 -0.000001<br>C 1.225045 -1.398953 -0.000000<br>C 0.000004 -0.713146 0.000000<br>C -0.000003 0.713146 -0.000000<br>C 1.225032 1.398965 -0.000001<br>C 2.469097 0.684468 -0.000001<br>H -1.240644 -2.483638 0.000001<br>H 3.397355 -1.241208 -0.000001<br>H 1.240669 -2.483625 0.000000<br>C -1.225030 -1.398966 0.000001<br>C -1.225045 1.398954 0.000000<br>H 1.240644 2.483637 -0.000001<br>H 3.397343 1.241242 -0.000002<br>C -2.469102 0.684444 0.000001<br>C -2.469096 -0.684468 0.000001<br>H -1.240669 2.483625 -0.000000<br>H -3.397354 1.241208 0.000001<br>H -3.397342 -1.241242 0.000002
21<br>C -1.351096 -1.154824 0.001595<br>C -2.182381 -0.003055 -0.000271<br>C -1.363640 1.148522 -0.001092<br>C -0.001796 0.713873 0.000381<br>C 0.001798 -0.713835 -0.000626<br>C 1.351128 1.154828 -0.001640<br>C 2.182383 0.003025 0.000387<br>C 1.363609 -1.148515 0.001201<br>H -1.706642 -2.178163 -0.003834<br>H -3.264672 -0.008189 0.000240<br>H -1.724047 2.170045 0.003984<br>H 1.706766 2.178140 0.003880<br>H 3.264670 0.008123 0.000016<br>H 1.723888 -2.170073 -0.003889	22<br>C 2.513188 -0.000000 0.051361<br>C 1.907890 1.269387 0.000572<br>C 0.565165 1.597879 -0.039752<br>C 1.907890 -1.269387 0.000585<br>C -0.559085 0.731312 -0.000175<br>C 0.565164 -1.597880 -0.039738<br>C -0.559086 -0.731312 -0.000168<br>H 3.597412 0.000000 0.086045<br>H 2.600376 2.108500 -0.014346<br>H 0.332551 2.658264 -0.089223<br>H 2.600375 -2.108500 -0.014329<br>H 0.332551 -2.658264 -0.089213<br>C -1.903919 1.139225 0.010527<br>H -2.238008 2.167846 0.015670<br>C -2.729517 0.000000 0.015588<br>H -3.809870 0.000001 0.033990<br>C -1.903920 -1.139225 0.010497<br>H -2.238009 -2.167845 0.015616	23<br>C 0.758644 0.689582 -0.014038<br>C 0.758645 -0.689578 0.013982<br>C -0.740168 0.719459 -0.001493<br>C -1.900559 1.438089 0.005692<br>C -3.119937 0.692008 0.004012<br>C -3.119937 -0.692005 -0.003785<br>C -1.900559 -1.438085 -0.005554<br>C -0.740168 -0.719455 0.001546<br>H -1.928933 2.521632 0.015754<br>H -4.063767 1.222907 0.006270<br>H -4.063768 -1.222904 -0.005975<br>H -1.928934 -2.521629 -0.015615<br>H 1.537688 1.437244 -0.033405<br>H 1.537691 -1.437240 0.033292	24<br>C 0.762970 0.673402 -0.000017<br>C 0.762970 -0.673398 -0.000039<br>C -0.758637 0.709574 0.000046<br>C -1.900694 1.440229 0.000109<br>C -3.118735 0.686526 0.000133<br>C -3.118735 -0.686523 0.000093<br>C -1.900693 -1.440225 0.000028<br>C -0.758637 -0.709571 0.000008<br>H -1.920815 2.521436 0.000138<br>H -4.061435 1.216832 0.000181<br>H -4.061435 -1.216829 0.000114<br>H -1.920815 -2.521433 0.000002<br>H 1.540315 1.421905 -0.000032<br>H 1.540315 -1.421901 -0.000081
25<br>C 2.470252 1.401900 -0.000202<br>C 1.218766 0.719954 -0.000094<br>C 3.646052 0.710744 -0.000293<br>C -0.000048 1.398706 0.000001<br>C 1.218766 -0.719955 -0.000083<br>C 3.646052 -0.710745 -0.000284<br>C -0.000048 -1.398706 0.000022<br>C -1.218450 0.720024 0.000106<br>C 2.470252 -1.401901 -0.000182<br>C -2.470302 1.401987 0.000204<br>C -1.218451 -0.720024 0.000117<br>C -2.470302 -1.401987 0.000225<br>C -3.645842 0.710925 0.000305<br>C -3.645842 -0.710925 0.000316<br>H 2.470217 2.484518 -0.000210<br>H 0.000142 2.482240 -0.000007<br>H 0.000142 -2.482241 0.000030<br>H 2.470216 -2.484519 -0.000175<br>H -2.469950 2.484611 0.000195	26<br>C 2.513188 -0.000000 0.051361<br>C 1.907890 1.269387 0.000572<br>C 0.565165 1.597879 -0.039752<br>C 1.907890 -1.269387 0.000585<br>C -0.559085 0.731312 -0.000175<br>C 0.565164 -1.597880 -0.039738<br>C -0.559086 -0.731312 -0.000168<br>H 3.597412 0.000000 0.086045<br>H 2.600376 2.108500 -0.014346<br>H 0.332551 2.658264 -0.089223<br>H 2.600375 -2.108500 -0.014329<br>H 0.332551 -2.658264 -0.089213<br>C -1.903919 1.139225 0.010527<br>H -2.238008 2.167846 0.015670<br>C -2.729517 0.000000 0.015588<br>H -3.809870 0.000001 0.033990<br>C -1.903920 -1.139225 0.010497<br>H -2.238009 -2.167845 0.015616	27<br>C -0.093348 1.295180 0.305009<br>C -1.315683 0.785814 -0.112058<br>C -1.207073 -0.598207 -0.101412<br>C 0.055821 -1.301502 -0.285653<br>C 1.278173 -0.792196 0.131357<br>C 1.169585 0.591829 0.120455<br>H -0.076269 2.204253 0.917832<br>H -2.255210 1.314658 -0.000967<br>H -2.078063 -1.198633 0.186912<br>H 0.038813 -2.210815 -0.898138<br>H 2.217683 -1.321083 0.020308<br>H 2.040358 1.192229 -0.168604	28<br>C 2.493237 0.000010 0.000006<br>C 1.903465 -1.261818 0.000137<br>C 0.549761 -1.589977 0.000181<br>C 1.903462 1.261822 -0.000123<br>C -0.551157 -0.747720 0.000101<br>C 0.549743 1.589975 -0.000161<br>C -0.551159 0.747718 -0.000084<br>H 3.577485 0.000009 0.000010<br>H 2.589849 -2.099589 0.000172<br>H 0.321148 -2.651074 0.000151<br>H 2.589833 2.099604 -0.000170<br>H 0.321133 2.651073 -0.000160<br>C -1.894588 -1.145900 -0.000286<br>H -2.238472 -2.168283 -0.000504<br>C -2.698786 -0.000002 0.000159<br>H -3.778719 -0.000010 0.000231<br>C -1.894606 1.145891 0.000089<br>H -2.238488 2.168275 0.000163

H -4.588277 1.241787 0.000378<br>H -2.469950 -2.484611 0.000233<br>H -4.588277 -1.241787 0.000396<br>H 4.588294 -1.241946 -0.000359<br>H 4.588294 1.241945 -0.000375			
29<br>C 0.000000 2.827141 0.875520<br>C 0.000000 3.548296 -0.295011<br>C -0.000000 1.417526 0.862511<br>C 0.000000 2.872120 -1.523522<br>C -0.000000 0.726396 -0.379482<br>C -0.000000 0.676717 2.086271<br>C 0.000000 1.495329 -1.561018<br>C -0.000000 -0.726396 -0.379482<br>C -0.000000 -0.676717 2.086271<br>C -0.000000 -1.495329 -1.561018<br>C -0.000000 -1.417526 0.862511<br>C 0.000000 -2.827141 0.875520<br>C -0.000000 -2.872120 -1.523522<br>C 0.000000 -3.548296 -0.295011<br>H -0.000000 3.337436 1.830381<br>H -0.000000 1.225224 3.019606<br>H 0.000000 1.005823 -2.523291<br>H -0.000000 -1.225224 3.019606<br>H 0.000000 -3.337436 1.830381<br>H 0.000000 -4.629534 -0.270764<br>H -0.000000 -1.005823 -2.523291<br>H -0.000000 -3.433746 -2.447960<br>H 0.000000 3.433746 -2.447960<br>H 0.000000 4.629534 -0.270764	30<br>C 2.493237 0.000010 0.000006<br>C 1.903465 -1.261818 0.000137<br>C 0.549761 -1.589977 0.000181<br>C 1.903462 1.261822 -0.000123<br>C -0.551157 -0.747720 0.000101<br>C 0.549743 1.589975 -0.000161<br>C -0.551159 0.747718 -0.000084<br>H 3.577485 0.000009 0.000010<br>H 2.589849 -2.099589 0.000172<br>H 0.321148 -2.651074 0.000151<br>H 2.589833 2.099604 -0.000170<br>H 0.321133 2.651073 -0.000160<br>C -1.894588 -1.145900 -0.000286<br>H -2.238472 -2.168283 -0.000504<br>C -2.698786 -0.000002 0.000159<br>H -3.778719 -0.000010 0.000231<br>C -1.894606 1.145891 0.000089<br>H -2.238488 2.168275 0.000163	31<br>C 0.685110 0.720853 -0.000008<br>C 0.685111 -0.720850 -0.000042<br>C -0.735883 0.744788 0.000045<br>C -1.892985 1.506516 0.000108<br>C -3.048650 0.726387 0.000129<br>C -3.048650 -0.726383 0.000092<br>C -1.892985 -1.506513 0.000029<br>C -0.735883 -0.744785 0.000007<br>H -1.918321 2.588974 0.000137<br>H -4.017432 1.215221 0.000177<br>H -4.017431 -1.215218 0.000115<br>H -1.918320 -2.588970 0.000002<br>H 1.471129 1.470545 -0.000020<br>H 1.471129 -1.470541 -0.000088	32<br>C 0.038470 0.361323 0.488122<br>C 1.368105 0.221897 -0.000297<br>C 1.368072 1.613872 0.000280<br>C 0.038507 1.474378 -0.488308<br>H 1.966143 -0.568330 -0.427241<br>H 1.966010 2.404127 0.427311
33<br>C -0.000000 1.378333 2.491362<br>C -0.000000 0.707623 1.252721<br>C -0.000000 1.438695 -0.013537<br>C -0.000000 2.846738 -0.052013<br>C -0.000000 -0.707623 1.252721<br>C -0.000000 0.731072 -1.239184<br>C -0.000000 -0.731072 -1.239184<br>C -0.000000 -1.438695 -0.013537<br>C -0.000000 -1.468411 -2.439353<br>C 0.000000 1.468411 -2.439353<br>C 0.000000 2.845546 -2.448807<br>C 0.000000 3.543495 -1.239922<br>H -0.000000 4.624985 -1.232088<br>H 0.000000 3.379509 -3.389320<br>C 0.000000 0.697948 3.688726<br>C 0.000000 -0.697948 3.688726<br>C 0.000000 -1.378333 2.491362<br>H 0.000000 1.245478 4.621407<br>H 0.000000 -1.245478 4.621407<br>C 0.000000 -2.846738 -0.052013<br>C -0.000000 -2.845546 -2.448807<br>H 0.000000 -3.379509 -3.389320<br>C 0.000000 -3.543495 -1.239922<br>H 0.000000 -4.624985 -1.232088<br>H 0.000000 0.954845 -3.387570<br>H -0.000000 -0.954845 -3.387570<br>H 0.000000 -3.411121 0.866865<br>H 0.000000 -2.456301 2.520713<br>H -0.000000 2.456301 2.520713<br>H -0.000000 3.411121 0.866865	34<br>C 1.372420 -0.750460 0.006758<br>C 1.390457 0.641821 0.087081<br>C 0.388219 1.498320 -0.521114<br>C 0.337996 -1.625114 -0.323915<br>C -0.892455 1.208256 -0.829482<br>C -1.066538 -1.256763 -0.308634<br>C -1.617788 -0.034319 -0.452683<br>H 2.324081 -1.234765 0.240969<br>H 2.330154 1.111062 0.353973<br>H 0.740799 2.488971 -0.820489<br>H 0.560056 -2.685678 -0.305155<br>H -1.464880 1.953177 -1.378473<br>H -1.760642 -2.073294 -0.092288<br>H -2.692356 0.059253 -0.309190	35<br>C 0.736183 -1.245162 0.000000<br>C -0.736184 1.245162 0.000000<br>C 1.434748 -0.000000 -0.000000<br>C -0.736184 -1.245162 0.000000<br>C -1.434748 -0.000000 0.000000<br>C 0.736183 1.245162 0.000000<br>C -2.865224 -0.000000 0.000000<br>C -3.563410 1.228497 0.000000<br>C -2.875984 2.413141 -0.000000<br>C -1.474679 2.417829 -0.000001<br>C 1.474679 2.417829 0.000001<br>C 2.875984 2.413141 0.000000<br>C 3.563410 1.228497 -0.000000<br>C 2.865224 -0.000000 -0.000000<br>H -4.645520 1.216080 0.000000<br>H -3.409232 3.354288 -0.000001<br>H -0.975331 3.374416 -0.000001<br>H 0.975331 3.374416 0.000002<br>H 3.409232 3.354288 0.000001<br>H 4.645519 1.216080 -0.000000<br>C -3.563409 -1.228498 0.000000<br>H -4.645519 -1.216081 0.000000<br>C -1.474678 -2.417829 -0.000000<br>H -0.975330 -3.374416 -0.000001<br>C -2.875983 -2.413141 -0.000000<br>H -3.409231 -3.354289 -0.000000<br>C 3.563409 -1.228498 -0.000000<br>H 4.645519 -1.216082 -0.000001<br>C 1.474678 -2.417829 0.000001<br>H 0.975329 -3.374416 0.000001<br>C 2.875983 -2.413141 0.000000<br>H 3.409231 -3.354289 0.000000	36<br>C 0.685110 0.720853 -0.000008<br>C 0.685111 -0.720850 -0.000042<br>C -0.735883 0.744788 0.000045<br>C -1.892985 1.506516 0.000108<br>C -3.048650 0.726387 0.000129<br>C -3.048650 -0.726383 0.000092<br>C -1.892985 -1.506513 0.000029<br>C -0.735883 -0.744785 0.000007<br>H -1.918321 2.588974 0.000137<br>H -4.017432 1.215221 0.000177<br>H -4.017431 -1.215218 0.000115<br>H -1.918320 -2.588970 0.000002<br>H 1.471129 1.470545 -0.000020<br>H 1.471129 -1.470541 -0.000088
37<br>C 0.621515 -1.229426 0.000133<br>C -0.619582 -1.229662 -0.000015<br>C -1.456068 -0.133038 -0.000009<br>C -0.701570 1.055202 0.000007<br>H -2.535758 -0.135364 0.000022<br>H -1.226431 2.002251 0.000010<br>C 1.456079 -0.131092 -0.000046<br>H 2.535790 -0.131803 -0.000020<br>C 0.700041 1.056101 -0.000002<br>H 1.223626 2.003855 0.000046	38<br>C 2.528134 -0.000000 0.000003<br>C 1.924834 1.257752 -0.000003<br>C 0.544911 1.592033 0.000008<br>C 1.924834 -1.257752 0.000010<br>C -0.504874 0.709337 0.000008<br>C 0.544911 -1.592033 0.000007<br>C -0.504874 -0.709337 -0.000001<br>H 3.612004 -0.000000 0.000006<br>H 2.603230 2.104412 -0.000015<br>H 0.313941 2.651483 -0.000014<br>H 2.603230 -2.104413 0.000026<br>H 0.313940 -2.651484 0.000040<br>C -1.938050 1.118156 -0.000082<br>H -2.270946 2.150350 -0.000059<br>C -2.759312 0.000000 -0.000009<br>H -3.839227 0.000001 0.000004<br>C -1.938051 -1.118156 0.000047<br>H -2.270947 -2.150349 0.000091	39<br>C -1.557243 -0.675689 0.375115<br>C -0.659812 -1.564040 -0.375069<br>C 0.659795 1.564052 -0.375080<br>C 0.675680 -1.557256 -0.375088<br>C 1.557269 0.675688 0.375031<br>C 1.564061 -0.659805 0.375031<br>H -2.343803 -1.180673 0.928597<br>H -1.156771 -2.355721 -0.928515<br>H 1.156724 2.355747 -0.928532<br>H 1.180639 -2.343847 -0.928548<br>H 2.343886 1.180660 0.928444<br>H 2.355775 -1.156751 0.928441<br>C -1.564036 0.659802 0.375123<br>H -2.355695 1.156751 0.928608<br>C -0.675697 1.557251 -0.375052<br>H -1.180685 2.343840 -0.928490	40<br>C -1.293952 1.103738 -0.000097<br>C -2.117355 -0.143276 -0.000073<br>C -1.301428 -1.218966 0.000004<br>C 0.078217 -0.710410 0.000004<br>C 0.006432 0.742882 -0.000044<br>C 1.378604 -1.071272 0.000026<br>C 2.202000 0.175717 0.000166<br>C 1.386090 1.251426 -0.000015<br>H -1.706231 2.102431 -0.000136<br>H -3.196082 -0.155705 -0.000114<br>H -1.601131 -2.254497 0.000028<br>H 1.790844 -2.069981 0.000077<br>H 3.280728 0.188182 0.000279<br>H 1.685825 2.286947 -0.000044
41<br>C 0.359829 1.418764 0.000045<br>C -0.786521 0.765701 -0.000055<br>C -1.408577 -0.397753 0.000059<br>C -0.269862 -1.064017 -0.000057<br>C 1.048759 -1.020988 0.000047<br>C 1.056373 0.298294 -0.000039	42<br>C 0.758644 0.689582 -0.014038<br>C 0.758645 -0.689578 0.013982<br>C -0.740168 0.719459 -0.001493<br>C -1.900559 1.438089 0.005692<br>C -3.119937 0.692008 0.004012<br>C -3.119937 -0.692005 -0.003785<br>C -1.900559 -1.438085 -0.005554<br>C -0.740168 -0.719455 0.001546<br>H -1.928933 2.521632 0.015754<br>H -4.063767 1.222907 0.006270<br>H -4.063768 -1.222904 -0.005975<br>H -1.928934 -2.521629 -0.015615	43<br>C -1.429128 1.155997 -0.000115<br>C -2.200330 -0.146955 -0.000073<br>C -1.345841 -1.192856 -0.000059<br>C 0.011383 -0.678121 0.000005<br>C -0.011383 0.678120 -0.000023<br>C 1.429128 -1.155997 0.000136<br>C 2.200330 0.146955 0.000056<br>C 1.345841 1.192856 0.000076<br>H -1.669982 1.766556 -0.876392<br>H -1.670050 1.766631 0.876090<br>H -3.278053 -0.200307 -0.000093<br>H -1.627439 -2.235756 -0.000049	44<br>C -0.401149 -0.088212 -0.006059<br>C 0.860888 -0.026246 -0.005869<br>C 1.520023 1.115374 -0.006554<br>C 0.942693 2.239323 -0.007455<br>C -0.440538 2.318724 -0.007763<br>C -1.161498 1.070019 -0.007022<br>H -1.017186 3.231902 -0.008513<br>H -2.240655 1.112842 -0.007253

	H 1.537688 1.437244 -0.033405<br>H 1.537691 -1.437240 0.033292	H 1.669965 -1.766529 0.876437<br>H 1.670069 -1.766658 -0.876045<br>H 3.278053 0.200306 0.000061<br>H 1.627439 2.235756 0.000075	
45<br>C -0.346033 -0.507287 -1.176657<br>H -0.156286 -0.242403 -2.205159<br>C -1.084231 -1.538618 -0.732649<br>H -1.601891 -2.262307 -1.345104<br>C -0.346050 -0.507261 1.176694<br>H -0.156328 -0.242348 2.205193<br>C -1.084321 -1.538545 0.732697<br>H -1.602041 -2.262185 1.345160<br>C 0.195739 0.251710 0.000014<br>H 1.291236 0.279853 0.000027<br>H -0.133064 1.297098 -0.000005	46<br>C 3.111381 -0.692101 -0.000125<br>C 3.111381 0.692105 -0.000090<br>C 1.908869 1.440237 -0.000031<br>C 0.753354 0.709398 -0.000011<br>C 0.753354 -0.709394 -0.000046<br>C 1.908870 -1.440233 -0.000103<br>H 4.056452 -1.218059 -0.000169<br>H 4.056452 1.218064 -0.000109<br>H 1.929475 2.521138 -0.000004<br>H 1.929476 -2.521134 -0.000130<br>C -0.753353 0.709398 0.000043<br>C -1.908869 1.440236 0.000106<br>C -3.111380 0.692105 0.000131<br>C -3.111380 -0.692101 0.000095<br>C -1.908869 -1.440233 0.000032<br>C -0.753353 -0.709394 0.000007<br>H -1.929475 2.521138 0.000134<br>H -4.056451 1.218063 0.000179<br>H -4.056451 -1.218060 0.000117<br>H -1.929475 -2.521134 0.000005	47<br>C 0.000000 2.833703 0.000000<br>C 0.000000 1.484140 0.000000<br>C -1.285278 0.742062 -0.000371<br>C -2.454072 1.416829 -0.001255<br>C 1.285278 0.742062 0.000371<br>C -1.285278 -0.742062 0.000371<br>C 0.000000 -1.484140 0.000000<br>C 1.285278 -0.742062 -0.000371<br>C 0.000000 -2.833703 0.000000<br>C -2.454072 -1.416829 0.001255<br>C 2.454072 1.416829 0.001255<br>C 2.454072 -1.416829 -0.001255<br>C -3.776376 -0.734093 0.198497<br>H -4.561804 -1.271543 -0.339209<br>H -4.031485 -0.836395 1.262841<br>C -3.776376 0.734093 -0.198497<br>H -4.561804 1.271543 0.339209<br>H -4.031485 0.836395 -1.262841<br>C -2.524656 -2.903375 -0.195234<br>H -3.381208 -3.314546 0.345273<br>H -2.744404 -3.073273 -1.258817<br>C -1.252032 -3.637935 0.197724<br>H -1.180122 -4.585703 -0.342150<br>H -1.288809 -3.912458 1.261513<br>C 1.252032 -3.637935 -0.197724<br>H 1.180122 -4.585703 0.342150<br>H 1.288809 -3.912458 -1.261513<br>C 2.524656 -2.903375 0.195234<br>H 3.381208 -3.314546 -0.345273<br>H 2.744404 -3.073273 1.258817<br>C 3.776376 -0.734093 -0.198497<br>H 4.561804 -1.271543 0.339209<br>H 4.031485 -0.836395 -1.262841<br>C 3.776376 0.734093 0.198497<br>H 4.561804 1.271543 -0.339209<br>H 4.031485 0.836395 1.262841<br>C 2.524656 2.903375 -0.195234<br>H 3.381208 3.314546 0.345273<br>H 2.744404 3.073273 -1.258817<br>C 1.252032 3.637935 0.197724<br>H 1.180122 4.585703 -0.342150<br>H 1.288809 3.912458 1.261513<br>C -1.252032 3.637935 -0.197724<br>H -1.180122 4.585703 0.342150<br>H -1.288809 3.912458 -1.261513<br>C -2.524656 2.903375 0.195234<br>H -3.381208 3.314546 -0.345273<br>H -2.744404 3.073273 1.258817	48<br>C -0.103093 -0.038469 1.155787<br>H -0.155043 0.028982 2.232717<br>C 0.238072 1.176456 0.218932<br>H 0.447008 2.173209 0.579289<br>C 0.215406 0.737147 -1.048949<br>H 0.400881 1.291716 -1.953070<br>C -0.304037 -1.113642 0.378221<br>H -0.555931 -2.117357 0.675687<br>C -0.115120 -0.667995 -0.983326<br>H -0.210385 -1.311311 -1.852292
49<br>C 0.000000 2.833703 0.000000<br>C 0.000000 1.484140 0.000000<br>C -1.285278 0.742062 -0.000371<br>C -2.454072 1.416829 -0.001255<br>C 1.285278 0.742062 0.000371<br>C -1.285278 -0.742062 0.000371<br>C 0.000000 -1.484140 0.000000<br>C 1.285278 -0.742062 -0.000371<br>C 0.000000 -2.833703 0.000000<br>C -2.454072 -1.416829 0.001255<br>C 2.454072 1.416829 0.001255<br>C 2.454072 -1.416829 -0.001255<br>C -3.776376 -0.734093 0.198497<br>H -4.561804 -1.271543 -0.339209<br>H -4.031485 -0.836395 1.262841<br>C -3.776376 0.734093 -0.198497<br>H -4.561804 1.271543 0.339209<br>H -4.031485 0.836395 -1.262841<br>C -2.524656 -2.903375 -0.195234<br>H -3.381208 -3.314546 0.345273<br>H -2.744404 -3.073273 -1.258817<br>C -1.252032 -3.637935 0.197724<br>H -1.180122 -4.585703 -0.342150<br>H -1.288809 -3.912458 1.261513<br>C 1.252032 -3.637935 -0.197724<br>H 1.180122 -4.585703 0.342150<br>H 1.288809 -3.912458 -1.261513<br>C 2.524656 -2.903375 0.195234<br>H 3.381208 -3.314546 -0.345273<br>H 2.744404 -3.073273 1.258817<br>C 3.776376 -0.734093 -0.198497<br>H 4.561804 -1.271543 0.339209<br>H 4.031485 -0.836395 -1.262841<br>C 3.776376 0.734093 0.198497<br>H 4.561804 1.271543 -0.339209<br>H 4.031485 0.836395 1.262841<br>C 2.524656 2.903375 -0.195234<br>H 3.381208 3.314546 0.345273<br>H 2.744404 3.073273 -1.258817<br>C 1.252032 3.637935 0.197724<br>H 1.180122 4.585703 -0.342150	50<br>C 0.762970 0.673402 -0.000017<br>C 0.762970 -0.673398 -0.000039<br>C -0.758637 0.709574 0.000046<br>C -1.900694 1.440229 0.000109<br>C -3.118735 0.686526 0.000133<br>C -3.118735 -0.686523 0.000093<br>C -1.900693 -1.440225 0.000028<br>C -0.758637 -0.709571 0.000008<br>H -1.920815 2.521436 0.000138<br>H -4.061435 1.216832 0.000181<br>H -4.061435 -1.216829 0.000114<br>H -1.920815 -2.521433 0.000002<br>H 1.540315 1.421905 -0.000032<br>H 1.540315 -1.421901 -0.000081	51<br>C -0.832188 -0.139283 0.022147<br>C 1.336434 1.112651 0.022041<br>C 0.672342 2.263009 0.021252<br>C -1.496280 1.011075 0.021358<br>H -1.379675 -1.074915 0.022644<br>H 2.420459 1.118888 0.022459<br>H 1.219830 3.198640 0.021032<br>H -2.580305 1.004839 0.021217<br>C 0.666711 -0.231478 0.022407<br>H 1.002677 -0.813355 0.890106<br>H 1.002926 -0.813933 -0.844816<br>C -0.826556 2.355204 0.020633<br>H -1.162321 2.936731 -0.847385<br>H -1.162973 2.938007 0.887536	52<br>C 0.110570 -1.419466 -0.065084<br>C 0.111580 1.419389 0.065073<br>C 1.252285 0.722630 0.109264<br>C 1.251768 -0.723518 -0.109281<br>H 0.112649 -2.494942 -0.189283<br>H 0.114425 2.494864 0.189267<br>H 2.196927 1.221150 0.285430<br>H 2.196054 -1.222710 -0.285457<br>C -1.189541 0.726271 -0.247405<br>H -2.032937 1.269735 0.180400<br>H -1.334883 0.742730 -1.336402<br>C -1.190060 -0.725422 0.247388<br>H -2.033843 -1.268284 -0.180421<br>H -1.335418 -0.741778 1.336384

H 1.288809 3.912458 1.261513<br>C -1.252032 3.637935 -0.197724<br>H -1.180122 4.585703 0.342150<br>H -1.288809 3.912458 -1.261513<br>C -2.524656 2.903375 0.195234<br>H -3.381208 3.314546 -0.345273<br>H -2.744404 3.073273 1.258817			
53<br>C 0.500874 0.016848 -0.000000<br>C 2.001921 -0.119781 -0.000000<br>C 1.153388 1.126019 0.000000<br>H -0.436854 -0.508015 0.000000<br>H 2.513850 -0.421414 -0.911489<br>H 2.513850 -0.421414 0.911489<br>H 1.157517 2.200616 0.000000	54<br>C 0.363664 -0.081020 -0.000000<br>C 1.868751 -0.080922 -0.000000<br>C 1.116125 1.222483 0.000000<br>H -0.144625 -0.374530 -0.907777<br>H -0.144625 -0.374530 0.907777<br>H 2.377081 -0.374361 -0.907777<br>H 2.377081 -0.374361 0.907777<br>H 1.116094 1.809426 0.907780<br>H 1.116094 1.809426 -0.907780	55<br>H -1.235721 2.040926 -0.056261<br>C -0.709729 1.140766 0.266847<br>C -0.653075 -1.360272 -0.024816<br>C 1.486178 -0.096445 0.245589<br>C 0.661834 -1.353766 0.178366<br>C 0.719556 1.122312 -0.279128<br>C -1.476788 -0.109773 -0.175976<br>H -1.177895 -2.307397 -0.085011<br>H 1.799086 0.075717 1.282361<br>H 1.186135 -2.294946 0.302547<br>H 0.682523 1.079295 -1.371789<br>H -1.788955 -0.006586 -1.222085<br>H -0.672870 1.170235 1.359965<br>H 2.411538 -0.233995 -0.320445<br>H 1.245922 2.041698 -0.016453<br>H -2.402546 -0.208881 0.397374	56<br>C 1.775071 0.406716 -0.000076<br>C 2.379842 1.454189 0.000043<br>C 0.369613 2.685241 -0.001314<br>C -0.296173 1.532066 -0.001151<br>H -0.190972 3.613732 -0.001848<br>H -1.380561 1.553282 -0.001599<br>C 0.331210 0.123372 -0.000379<br>H -0.001622 -0.439772 -0.875950<br>H -0.002083 -0.438982 0.875527<br>C 1.903268 2.846275 -0.000797<br>H 2.223752 3.416545 0.874769<br>H 2.224407 3.415649 -0.876709
57<br>C 0.567705 -1.266528 -0.116476<br>C -0.647400 -1.288844 -0.091060<br>C -1.536348 -0.157938 0.050601<br>C -0.856509 1.006390 0.072501<br>H -2.605435 -0.218812 0.188597<br>H -1.396886 1.935418 0.213541<br>C 1.501627 -0.167765 0.191522<br>H 1.765336 -0.189540 1.252719<br>H 2.429929 -0.151677 -0.376730<br>C 0.637717 1.110586 -0.167263<br>H 0.775250 1.301945 -1.238027<br>H 1.048369 1.977159 0.354331	58<br>C -0.000026 -1.224635 -0.138977<br>C 1.231411 -0.318236 0.104544<br>C -1.231422 -0.318180 0.104537<br>C 0.664482 1.071558 -0.045520<br>C -0.664427 1.071590 -0.045505<br>H -0.000031 -1.558589 -1.177376<br>H -0.000048 -2.114544 0.488789<br>H 2.046062 -0.523846 -0.592525<br>H 1.643592 -0.453706 1.110659<br>H -1.643617 -0.453638 1.110647<br>H -2.046079 -0.523748 -0.592538<br>H 1.284753 1.955951 -0.102628<br>H -1.284656 1.956013 -0.102605	59<br>C 0.003644 0.489838 -0.000028<br>C 1.515054 0.371612 -0.000043<br>C 1.515020 1.942889 -0.000072<br>C 0.003615 1.824597 -0.000261<br>H -0.781674 -0.254376 0.000012<br>H 1.956882 -0.085606 -0.887144<br>H 1.956866 -0.085541 0.887096<br>H 1.956913 2.400059 -0.887161<br>H 1.956727 2.400127 0.887078<br>H -0.781735 2.568777 -0.000390	60<br>C -0.744380 0.694840 0.000143<br>C -1.912608 1.433527 0.000275<br>C -3.103375 0.698645 0.000209<br>C -3.103375 -0.698642 -0.000024<br>C -1.912608 -1.433524 -0.000145<br>C -0.744380 -0.694836 -0.000098<br>H -1.927577 2.515609 0.000524<br>H -4.050870 1.221322 0.000351<br>H -4.050870 -1.221319 -0.000118<br>H -1.927576 -2.515606 -0.000393<br>C 0.771537 -0.790172 -0.000144<br>H 1.215804 -1.244196 0.886172<br>H 1.216201 -1.244131 -0.886300<br>C 0.771537 0.790176 0.000118<br>H 1.215770 1.244204 -0.886214<br>H 1.216234 1.244132 0.886259
61<br>H -0.024950 2.254852 0.168043<br>C 0.093372 1.277122 -0.303216<br>C 0.049860 -1.412664 -0.028170<br>C -1.285382 0.535955 -0.283005<br>H -1.967148 1.030235 0.413634<br>H 0.387085 1.461541 -1.337196<br>H -1.753224 0.603207 -1.266553<br>C 1.273159 0.518151 0.388281<br>H 1.127572 0.495859 1.467962<br>H 2.213497 1.032946 0.188011<br>C -1.139642 -0.906742 0.205581<br>C 1.239395 -0.882213 -0.208518<br>H 1.978149 -1.209829 -0.928000<br>H -1.885907 -1.330726 0.864222	62<br>C -0.035993 0.492368 0.000137<br>C 1.539304 0.492394 -0.000156<br>C 1.539282 1.822115 0.000159<br>C -0.036015 1.822089 -0.000411<br>H -0.798541 -0.271127 0.000346<br>H 2.301877 -0.271076 -0.000230<br>H 2.301830 2.585611 0.000368<br>H -0.798587 2.585559 -0.000752	63<br>H -2.475623 -0.261328 -0.141044<br>C -1.453936 -0.153459 0.229659<br>C 0.859917 -1.182398 0.229699<br>C 0.593985 1.335807 0.229680<br>C 1.453936 0.153459 -0.229659<br>C -0.859917 1.182398 -0.229699<br>C -0.593985 -1.335807 -0.229680<br>H 0.896244 -1.232357 1.323179<br>H 0.619064 1.392232 1.323155<br>H 1.515444 0.159926 -1.323142<br>H -0.896244 1.232357 -1.323179<br>H -0.619064 -1.392232 -1.323155<br>H -1.515444 -0.159926 1.323142<br>H 1.464097 -2.013340 -0.140976<br>H 1.011482 2.274540 -0.140972<br>H 2.475623 0.261328 0.141044<br>H -1.464097 2.013340 0.140976<br>H -1.011482 -2.274540 0.140972	64<br>C 0.310084 -1.517039 -0.403440<br>C 1.540928 -0.757600 0.110646<br>C 1.540902 0.757647 -0.110651<br>C -0.964830 -1.242531 0.408724<br>C 0.310037 1.517048 0.403442<br>C -1.773887 -0.000030 -0.000004<br>C -0.964872 1.242505 -0.408717<br>H 0.142444 -1.290636 -1.461263<br>H 1.640462 -0.960924 1.182299<br>H 1.640423 0.960975 -1.182305<br>H 0.142409 1.290639 1.461266<br>H -0.681814 -1.160713 1.461944<br>H -2.433627 0.260836 0.831924<br>H 0.530984 -2.585919 -0.355339<br>H 2.437839 -1.170734 -0.358981<br>H 2.437803 1.170811 0.358970<br>H -1.628417 -2.108361 0.350696<br>H 0.530905 2.585935 0.355342<br>H -2.433599 -0.260924 -0.831945<br>H -1.628488 2.108313 -0.350673<br>H -0.681859 1.160713 -1.461939
65<br>C -0.605294 -1.135587 -0.000036<br>C 0.604741 -1.135604 0.000050<br>C 0.605296 1.135645 0.000006<br>C -0.604738 1.135646 0.000007<br>C -1.609329 0.000287 0.000026<br>H -2.253375 0.000462 -0.878637<br>H -2.253261 0.000425 0.878774<br>C 1.609325 -0.000223 -0.000034<br>H 2.253387 -0.000369 0.878618<br>H 2.253244 -0.000406 -0.878792	66<br>C 0.000000 -0.000000 1.774891<br>H 0.621923 0.621923 2.424809<br>H -0.621923 -0.621923 2.424809<br>C 0.889546 -0.889546 0.889546<br>H 1.520387 -1.520387 1.520387<br>C -0.889546 0.889546 0.889546<br>H -1.520387 1.520387 1.520387<br>C 0.000000 1.774891 0.000000<br>H 0.621923 2.424809 0.621923<br>H -0.621923 2.424809 -0.621923<br>C 1.774891 -0.000000 -0.000000<br>H 2.424809 -0.621923 -0.621923<br>H 2.424809 0.621923 0.621923<br>C 0.889546 0.889546 -0.889546<br>H 1.520387 1.520387 -1.520387<br>C -0.000000 0.000000 -1.774891<br>H -0.621923 0.621923 -2.424809<br>H 0.621923 -0.621923 -2.424809<br>C -0.000000 -1.774891 0.000000<br>H -0.621923 -2.424809 0.621923<br>H 0.621923 -2.424809 -0.621923<br>C -1.774891 0.000000 -0.000000<br>H -2.424809 -0.621923 0.621923<br>H -2.424809 0.621923 -0.621923<br>C -0.889546 -0.889546 -0.889546<br>H -1.520387 -1.520387 -1.520387	67<br>C -0.633260 -0.237836 1.730380<br>C -0.257724 1.202575 1.342164<br>C -0.123313 -1.268012 -1.101376<br>C -0.646921 1.683681 -0.071402<br>C 0.017777 -0.007466 -1.967728<br>C 0.292789 1.312286 -1.231355<br>H -1.590461 -0.484581 1.263618<br>H 0.815048 1.361965 1.493803<br>H -1.666074 1.352104 -0.296289<br>H -1.116340 -1.278772 -0.649958<br>H -0.892697 0.105752 -2.563607<br>H -0.824496 -0.267436 2.806015<br>H -0.756853 1.868969 2.049546<br>H -0.098856 -2.137284 -1.761232<br>H -0.694911 2.774363 -0.030071<br>H 0.829842 -0.159464 -2.685362<br>H 0.248227 2.106593 -1.980702<br>H 1.321627 1.325680 -0.862162<br>C 0.394296 -1.340282 1.432796<br>H -0.072189 -2.299643 1.676861<br>H 1.236195 -1.231311 2.122857<br>C 0.946681 -1.405557 0.000951<br>H 1.701467 -0.627884 -0.123651<br>H 1.485096 -2.348448 -0.111769	68<br>C -1.523123 -0.000013 -0.000018<br>C 0.661086 1.221650 0.384893<br>C 0.661104 -1.221647 -0.384879<br>C 1.523122 0.000012 -0.000004<br>C -0.661089 -1.221664 0.384840<br>C -0.661102 1.221664 -0.384835<br>H -2.177742 0.258424 0.835183<br>H -2.177687 -0.258472 -0.835255<br>H -1.214986 2.144538 -0.205342<br>H 0.443838 -1.200087 -1.456475<br>H 1.214957 2.144543 0.205456<br>H -0.443831 1.200150 -1.456430<br>H 0.443817 1.200066 1.456487<br>H 2.177730 -0.258459 0.835194<br>H 2.177697 0.258502 -0.835223<br>H -1.214959 -2.144551 0.205366<br>H -0.443828 -1.200121 1.456437<br>H 1.214986 -2.144529 -0.205423
69<br>C 0.604872 -1.293957 -0.024560<br>C -0.604903 -1.293957 0.024636<br>C -1.585377 -0.196877 0.116074<br>H -1.973727 -0.102687 1.132253	70<br>C 0.159422 1.278725 0.128218<br>C -1.137191 0.539811 -0.243205<br>C -0.887729 -0.887490 0.254812<br>C 0.569729 -1.149620 -0.152682	71<br>C 1.028686 0.336360 0.124502<br>C 0.336351 -1.028692 -0.124482<br>C -0.336355 1.028678 -0.124587<br>C -1.028683 -0.336346 0.124566	72<br>C 1.079861 -0.000343 0.136443<br>H 1.373920 0.001028 1.187152<br>H 1.985072 -0.001681 -0.476397<br>C 0.000856 1.079985 -0.135455

H -2.442323 -0.289227 -0.551434<br>C 1.585387 -0.196922 -0.116022<br>H 1.973743 -0.102773 -1.132202<br>H 2.442326 -0.289275 0.551495<br>C 0.717158 1.053922 0.282881<br>H 1.232516 1.957547 -0.051409<br>H 0.670600 1.095126 1.373437<br>C -0.717116 1.053927 -0.282877<br>H -0.670555 1.095089 -1.373435<br>H -1.232450 1.957578 0.051377	C 1.286384 0.215775 0.012741<br>H 0.339756 2.150003 -0.501119<br>H 0.090137 1.639226 1.156401<br>H -1.267817 0.529428 -1.329091<br>H -2.026689 1.000529 0.187318<br>H -0.986345 -0.919040 1.343857<br>H -1.583812 -1.618534 -0.157482<br>H 1.036870 -1.942514 0.431254<br>H 0.600525 -1.463280 -1.198400<br>H 1.916312 0.223821 0.902410<br>H 1.941637 0.417920 -0.834485	H 1.347100 0.440534 1.162455<br>H 1.862281 0.608889 -0.521779<br>H 0.440455 -1.347150 -1.162428<br>H 0.608925 -1.862259 0.521817<br>H -0.608912 1.862317 0.521626<br>H -0.440484 1.347021 -1.162566<br>H -1.862295 -0.608947 -0.521664<br>H -1.347070 -0.440405 1.162538	H 0.003481 1.375003 -1.185893<br>H -0.000142 1.984637 0.478211<br>C -1.080493 0.000730 0.131347<br>H -1.379503 0.002395 1.180657<br>H -1.982803 0.000290 -0.485757<br>C -0.000225 -1.080372 -0.132335<br>H 0.002103 -1.378425 -1.181916<br>H -0.002127 -1.983246 0.483942
73<br>C 0.000374 1.116092 -0.118301<br>C -1.270517 0.191518 0.107470<br>C 1.270696 0.190727 0.107487<br>C -0.608256 -1.150341 -0.060193<br>C 0.607424 -1.150553 -0.060195<br>H 0.000497 1.451478 -1.155873<br>H 0.000659 2.011912 0.506495<br>H -2.066143 0.405345 -0.604027<br>H -1.687202 0.292413 1.110406<br>H 1.687450 0.291365 1.110421<br>H 2.066448 0.404024 -0.604029			
ωB97-XD			
1<br>C 0.000000 1.387133 0.000000<br>C -1.201397 0.693588 -0.000000<br>C -1.201397 -0.693588 0.000000<br>C 0.000000 -1.387133 -0.000000<br>C 1.201397 -0.693588 0.000000<br>C 1.201397 0.693588 -0.000000<br>H -0.000000 2.468751 -0.000000<br>H -2.138060 1.234485 -0.000000<br>H -2.138060 -1.234485 -0.000000<br>H 0.000000 -2.468751 -0.000000<br>H 2.138060 -1.234485 0.000000<br>H 2.138060 1.234485 -0.000000	2<br>C 0.689868 1.198392 0.068757<br>C 1.378290 -0.004214 0.136984<br>C 0.690299 -1.206773 0.068553<br>C -0.690010 -1.206940 -0.068547<br>C -1.378290 -0.004547 -0.136982<br>C -0.690158 1.198226 -0.068759<br>H 1.224636 2.137099 0.121820<br>H 2.455157 -0.003891 0.243750<br>H 1.224995 -2.145532 0.121603<br>H -1.224480 -2.145828 -0.121594<br>H -1.225152 2.136803 -0.121825<br>H -2.455158 -0.004483 -0.243748	3<br>C -0.535748 0.292718 0.000058<br>C 0.851806 0.292993 0.000494<br>C 1.545890 1.494469 -0.000055<br>C 0.852396 2.695894 -0.001044<br>C -0.535117 2.695621 -0.001486<br>C -1.229223 1.494111 -0.000942<br>H -1.066917 -0.650544 0.000499<br>H 1.392543 -0.643558 0.001267<br>H 2.628352 1.482701 0.000305<br>H 1.383615 3.639129 -0.001473<br>H -1.075903 3.632145 -0.002245<br>H -2.311684 1.505936 -0.001290	4<br>C -0.745194 0.692433 0.000120<br>C -1.909808 1.429915 0.000260<br>C -3.096109 0.696490 0.000195<br>C -3.096109 -0.696487 -0.000010<br>C -1.909808 -1.429912 -0.000140<br>C -0.745194 -0.692429 -0.000070<br>H -1.925500 2.511652 0.000456<br>H -4.043807 1.218494 0.000313<br>H -4.043806 -1.218491 -0.000074<br>H -1.925499 -2.511649 -0.000333<br>C 0.767200 -0.786270 -0.000133<br>H 1.208919 -1.239382 0.886644<br>H 1.209030 -1.239243 -0.886925<br>C 0.767200 0.786274 0.000112<br>H 1.208879 1.239387 -0.886684<br>H 1.209069 1.239246 0.886885
5<br>C 0.002108 1.842218 0.010450<br>H 0.015250 2.928708 0.018061<br>C 1.280064 1.290224 -0.133077<br>H 2.048927 2.042701 -0.282315<br>C -1.289062 1.319302 0.144135<br>H -2.040228 2.088149 0.299925<br>C -1.842262 0.041365 0.005199<br>H -2.928777 0.054495 0.006894<br>C 1.802959 -0.002055 -0.010474<br>H 2.889450 -0.015150 -0.018106<br>C 1.250914 -1.281183 0.122003<br>H 2.003349 -2.051328 0.264705<br>C -0.041372 -1.802962 -0.005192<br>H -0.054499 -2.889477 -0.006885<br>C -1.320476 -1.249780 -0.133050<br>H -2.090611 -2.000960 -0.282272	6<br>C -0.000000 1.374209 2.483994<br>C 0.000000 0.702751 1.250925<br>C 0.000000 1.434815 -0.016920<br>C 0.000000 2.838425 -0.051806<br>C 0.000000 -0.702751 1.250925<br>C 0.000000 0.732071 -1.234211<br>C 0.000000 -0.732071 -1.234211<br>C 0.000000 -1.434815 -0.016920<br>C 0.000000 -1.464367 -2.432239<br>C 0.000000 1.464367 -2.432239<br>C -0.000000 2.837459 -2.441768<br>C 0.000000 3.533386 -1.236094<br>H 0.000000 4.614643 -1.227820<br>H -0.000000 3.371171 -3.382168<br>C -0.000000 0.696056 3.678027<br>C 0.000000 -0.696056 3.678027<br>C 0.000000 -1.374209 2.483994<br>H -0.000000 1.243871 4.610280<br>H 0.000000 -1.243871 4.610280<br>C -0.000000 -2.838425 -0.051806<br>C -0.000000 -2.837459 -2.441768<br>H 0.000000 -3.371171 -3.382168<br>C -0.000000 -3.533386 -1.236094<br>H -0.000000 -4.614643 -1.227820<br>H 0.000000 0.950665 -3.380708<br>H 0.000000 -0.950665 -3.380708<br>H -0.000000 -3.402916 0.867380<br>H 0.000000 -2.452484 2.513126<br>H -0.000000 2.452484 2.513126<br>H -0.000000 3.402916 0.867380	7<br>C 0.000000 2.819086 0.871687<br>C 0.000000 3.537247 -0.295171<br>C -0.000000 1.412900 0.857372<br>C -0.000000 2.861752 -1.520619<br>C 0.000000 0.726489 -0.375034<br>C 0.000000 0.673037 2.083664<br>C 0.000000 1.489931 -1.556445<br>C 0.000000 -0.726489 -0.375034<br>C -0.000000 -0.673037 2.083664<br>C -0.000000 -1.489931 -1.556445<br>C 0.000000 -1.412900 0.857372<br>C -0.000000 -2.819086 0.871687<br>C 0.000000 -2.861752 -1.520619<br>C -0.000000 -3.537247 -0.295171<br>H 0.000000 3.328871 1.826841<br>H 0.000000 1.223221 3.015964<br>H -0.000000 0.998790 -2.518260<br>H -0.000000 -1.223221 3.015964<br>H -0.000000 -3.328871 1.826841<br>H -0.000000 -4.618284 -0.271340<br>H 0.000000 -0.998790 -2.518260<br>H 0.000000 -3.422385 -2.445418<br>H -0.000000 3.422385 -2.445418<br>H 0.000000 4.618284 -0.271340	8<br>C 0.007492 1.589946 0.425355<br>C 1.274288 0.984175 0.357433<br>C 1.584095 -0.374761 0.168928<br>C -1.257555 0.983989 0.321213<br>C 0.709559 -1.465662 0.001843<br>C -1.573321 -0.374007 0.124092<br>C -0.697923 -1.467112 -0.018474<br>H 0.009698 2.666507 0.578181<br>H 2.122205 1.656378 0.464850<br>H 2.644022 -0.616446 0.149593<br>H -2.108391 1.655847 0.404199<br>H 1.182387 -2.435679 -0.128891<br>H -2.633055 -0.611193 0.075527<br>H -1.168653 -2.435519 -0.161981
9<br>C -0.000000 1.197246 0.000000<br>H 0.000000 2.280474 0.000000<br>C 1.138622 0.369991 -0.000000<br>H 2.168846 0.704729 -0.000000<br>C 0.703700 -0.968621 -0.000000<br>H 1.340413 -1.844959 -0.000000<br>C -1.138622 0.369991 -0.000000<br>H -2.168846 0.704729 -0.000000<br>C -0.703700 -0.968621 0.000000<br>H -1.340413 -1.844959 0.000000	10<br>C 2.518852 -0.000000 0.000008<br>C 1.918672 1.254063 -0.000005<br>C 0.544537 1.588674 -0.000008<br>C 1.918672 -1.254064 0.000017<br>C -0.497437 0.706006 -0.000007<br>C 0.544537 -1.588674 0.000012<br>C -0.497437 -0.706006 -0.000004<br>H 3.602462 -0.000000 0.000015<br>H 2.598551 2.099421 -0.000014<br>H 0.312395 2.647814 -0.000029<br>H 2.598551 -2.099421 0.000036<br>H 0.312395 -2.647814 0.000037<br>C -1.933494 1.111722 -0.000069<br>H -2.266525 2.144538 -0.000048<br>C -2.753812 0.000000 0.000001<br>H -3.833210 0.000001 0.000028<br>C -1.933494 -1.111722 0.000026<br>H -2.266526 -2.144538 0.000070	11<br>C 0.000000 1.841361 0.000000<br>H 0.000000 2.934860 0.000000<br>C -1.302040 1.302040 0.000000<br>H -2.075260 2.075260 -0.000000<br>C 1.302040 1.302040 0.000000<br>H 2.075260 2.075260 -0.000000<br>C 1.841361 -0.000000 -0.000000<br>H 2.934860 -0.000000 -0.000000<br>C -1.841361 -0.000000 -0.000000<br>H -2.934860 -0.000000 0.000000<br>C -1.302040 -1.302040 -0.000000<br>H -2.075260 -2.075260 0.000000<br>C 0.000000 -1.841361 0.000000<br>H 0.000000 -2.934860 0.000000<br>C 1.302040 -1.302040 -0.000000<br>H 2.075260 -2.075260 0.000000	12<br>C 3.101773 -0.689642 -0.000124<br>C 3.101773 0.689647 -0.000090<br>C 1.903530 1.436127 -0.000031<br>C 0.753172 0.705979 -0.000011<br>C 0.753172 -0.705976 -0.000046<br>C 1.903530 -1.436123 -0.000103<br>H 4.047191 -1.214752 -0.000169<br>H 4.047191 1.214757 -0.000108<br>H 1.923849 2.516703 -0.000004<br>H 1.923850 -2.516699 -0.000130<br>C -0.753172 0.705979 0.000043<br>C -1.903530 1.436126 0.000105<br>C -3.101772 0.689646 0.000130<br>C -3.101772 -0.689643 0.000095<br>C -1.903529 -1.436123 0.000032<br>C -0.753172 -0.705976 0.000007<br>H -1.923849 2.516703 0.000134<br>H -4.047191 1.214756 0.000179<br>H -4.047191 -1.214753 0.000117<br>H -1.923849 -2.516700 0.000005
13	14	15	16

C 2.416900 -0.704944 -0.000001<br>C 1.238253 -1.392951 -0.000000<br>C 0.000005 -0.708954 0.000000<br>C -0.000004 0.708953 -0.000000<br>C 1.238238 1.392963 -0.000001<br>C 2.416892 0.704968 -0.000001<br>H -1.236314 -2.475596 0.000001<br>H 3.356896 -1.239672 -0.000001<br>H 1.236340 -2.475583 -0.000000<br>C -1.238239 -1.392964 0.000001<br>C -1.238252 1.392951 0.000000<br>H 1.236315 2.475595 -0.000001<br>H 3.356883 1.239705 -0.000001<br>C -2.416899 0.704945 0.000000<br>C -2.416891 -0.704969 0.000001<br>H -1.236338 2.475583 -0.000000<br>H -3.356897 1.239670 0.000000<br>H -3.356883 -1.239704 0.000001	C 0.737532 -1.242375 -0.033223<br>C -0.737677 1.242488 -0.028674<br>C 1.434394 0.000075 -0.021536<br>C -0.737575 -1.242390 -0.000380<br>C -1.434462 0.000045 -0.012392<br>C 0.737587 1.242503 -0.004926<br>C -2.854360 0.000035 -0.008129<br>C -3.553110 1.226150 -0.036975<br>C -2.869783 2.404784 -0.069497<br>C -1.468323 2.408471 -0.064565<br>C 1.468213 2.408493 0.031116<br>C 2.869675 2.404838 0.035574<br>C 3.553019 1.226229 0.002485<br>C 2.854291 0.000100 -0.026247<br>H -4.635185 1.211873 -0.035134<br>H -3.402289 3.345576 -0.097058<br>H -0.968703 3.364875 -0.091083<br>H 0.968576 3.364874 0.058137<br>H 3.402169 3.345636 0.063168<br>H 4.635094 1.211983 0.000137<br>C -3.552904 -1.226090 0.024886<br>H -4.634971 -1.211835 0.029522<br>C -1.467969 -2.408387 0.039703<br>H -0.968206 -3.364803 0.062918<br>C -2.869375 -2.404718 0.053140<br>H -3.401691 -3.345522 0.083729<br>C 3.552855 -1.226010 -0.059378<br>H 4.634920 -1.211724 -0.064520<br>C 1.467946 -2.408365 -0.073155<br>H 0.968204 -3.364804 -0.095868<br>C 2.869347 -2.404663 -0.087066<br>H 3.401675 -3.345462 -0.117622	C 2.419965 -0.705113 -0.000001<br>C 1.240458 -1.392156 -0.000000<br>C 0.000004 -0.709080 0.000000<br>C -0.000003 0.709080 -0.000000<br>C 1.240445 1.392168 -0.000001<br>C 2.419958 0.705137 -0.000001<br>H -1.240799 -2.475650 0.000001<br>H 3.362388 -1.235184 -0.000001<br>H 1.240825 -2.475637 0.000000<br>C -1.240444 -1.392168 0.000001<br>C -1.240458 1.392156 0.000000<br>H 1.240800 2.475649 -0.000001<br>H 3.362376 1.235217 -0.000002<br>C -2.419964 0.705113 0.000001<br>C -2.419957 -0.705137 0.000001<br>H -1.240824 2.475637 -0.000000<br>H -3.362388 1.235184 0.000001<br>H -3.362375 -1.235217 0.000001	C 1.241853 3.510474 0.000000<br>C -0.000000 2.825832 0.000000<br>C -0.000000 1.420483 -0.000000<br>C 1.230220 0.710252 -0.000000<br>C 2.447335 1.412931 -0.000000<br>C 2.419324 2.830718 -0.000000<br>C -1.230220 0.710252 -0.000000<br>C 1.230220 -0.710252 -0.000000<br>C -0.000000 -1.420483 -0.000000<br>C -1.230220 -0.710252 -0.000000<br>C -0.000000 -2.825832 0.000000<br>C 1.241853 -3.510474 0.000000<br>C 2.419324 -2.830718 -0.000000<br>C 2.447335 -1.412931 -0.000000<br>C 3.661189 -0.679798 0.000000<br>C 3.661189 0.679798 -0.000000<br>H 4.597130 1.223686 -0.000000<br>H 4.597130 -1.223686 0.000000<br>H 1.238852 4.592959 0.000000<br>H 3.358244 3.369445 -0.000000<br>H 1.238852 -4.592959 0.000000<br>H 3.358244 -3.369445 0.000000<br>C -1.241853 3.510474 0.000000<br>C -2.419324 2.830718 -0.000000<br>C -2.447335 1.412931 -0.000000<br>H -1.238852 4.592959 0.000000<br>H -3.358244 3.369445 0.000000<br>C -3.661190 0.679798 -0.000000<br>C -3.661190 -0.679798 -0.000000<br>C -2.447335 -1.412931 -0.000000<br>H -4.597130 1.223686 -0.000000<br>H -4.597130 -1.223686 -0.000000<br>C -1.241853 -3.510474 0.000000<br>H -1.238852 -4.592959 0.000000<br>C -2.419324 -2.830718 0.000000<br>H -3.358244 -3.369445 0.000000
17	18	19	20
C 2.465729 1.398410 -0.000202<br>C 1.214084 0.714764 -0.000094<br>C 3.635139 0.710899 -0.000293<br>C 0.000025 1.393362 0.000001<br>C 1.214084 -0.714764 -0.000084<br>C 3.635139 -0.710900 -0.000283<br>C 0.000025 -1.393363 0.000021<br>C -1.213860 0.714800 0.000105<br>C 2.465728 -1.398410 -0.000181<br>C -2.465659 1.398452 0.000203<br>C -1.213860 -0.714800 0.000115<br>C -2.465659 -1.398452 0.000224<br>C -3.634968 0.710973 0.000304<br>C -3.634968 -0.710973 0.000315<br>H 2.464181 2.480983 -0.000210<br>H 0.000089 2.477063 -0.000007<br>H 0.000089 -2.477064 0.000029<br>H 2.464180 -2.480984 -0.000173<br>H -2.464008 2.481027 0.000195<br>H -4.577221 1.241761 0.000378<br>H -2.464008 -2.481027 0.000232<br>H -4.577221 -1.241761 0.000397<br>H 4.577322 -1.241809 -0.000356<br>H 4.577322 1.241808 -0.000374	C 1.241853 3.510474 0.000000<br>C -0.000000 2.825832 0.000000<br>C -0.000000 1.420483 -0.000000<br>C 1.230220 0.710252 -0.000000<br>C 2.447335 1.412931 -0.000000<br>C 2.419324 2.830718 -0.000000<br>C -1.230220 0.710252 -0.000000<br>C 1.230220 -0.710252 -0.000000<br>C -0.000000 -1.420483 -0.000000<br>C -1.230220 -0.710252 -0.000000<br>C -0.000000 -2.825832 0.000000<br>C 1.241853 -3.510474 0.000000<br>C 2.419324 -2.830718 0.000000<br>C 2.447335 -1.412931 -0.000000<br>C 3.661189 -0.679798 0.000000<br>C 3.661189 0.679798 -0.000000<br>H 4.597130 1.223686 -0.000000<br>H 4.597130 -1.223686 0.000000<br>H 1.238852 4.592959 0.000000<br>H 3.358244 3.369445 -0.000000<br>H 1.238852 -4.592959 0.000000<br>H 3.358244 -3.369445 0.000000<br>C -1.241853 3.510474 0.000000<br>C -2.419324 2.830718 -0.000000<br>C -2.447335 1.412931 -0.000000<br>H -1.238852 4.592959 0.000000<br>H -3.358244 3.369445 -0.000000<br>C -3.661190 0.679798 -0.000000<br>C -3.661190 -0.679798 -0.000000<br>C -2.447335 -1.412931 -0.000000<br>H -4.597130 1.223686 -0.000000<br>H -4.597130 -1.223686 -0.000000<br>C -1.241853 -3.510474 0.000000<br>H -1.238852 -4.592959 0.000000<br>C -2.419324 -2.830718 0.000000<br>H -3.358244 -3.369445 0.000000	C 0.689488 0.666322 -0.205835<br>C 0.666295 -0.689514 0.205823<br>H 1.397042 1.349718 -0.686477<br>H 1.349996 -1.396799 0.686427<br>C -0.666289 0.689553 0.205707<br>C -0.689492 -0.666363 -0.205688<br>H -1.349980 1.396921 0.686199<br>H -1.397068 -1.349832 -0.686187	C 2.461249 -0.680539 -0.000001<br>C 1.217632 -1.395988 -0.000000<br>C 0.000004 -0.711769 0.000000<br>C -0.000003 0.711769 -0.000000<br>C 1.217618 1.396000 -0.000001<br>C 2.461242 0.680563 -0.000001<br>H -1.233065 -2.480971 0.000001<br>H 3.389395 -1.236916 -0.000001<br>H 1.233091 -2.480958 0.000000<br>C -1.217617 -1.396000 0.000001<br>C -1.217631 1.395988 0.000000<br>H 1.233066 2.480970 -0.000001<br>H 3.389382 1.236949 -0.000002<br>C -2.461248 0.680539 0.000001<br>C -2.461241 -0.680563 0.000001<br>H -1.233090 2.480958 -0.000000<br>H -3.389394 1.236915 0.000001<br>H -3.389381 -1.236949 0.000002
21	22	23	24
C -1.360540 -1.160800 0.000111<br>C -2.172727 0.000019 0.000049<br>C -1.360520 1.160822 0.000079<br>C 0.000005 0.717474 -0.000020<br>C -0.000008 -0.717475 0.000019<br>C 1.360538 1.160800 -0.000121<br>C 2.172725 -0.000020 -0.000098<br>C 1.360518 -1.160825 -0.000031<br>H -1.724975 -2.184304 0.000166<br>H -3.261546 0.000029 0.000052<br>H -1.724937 2.184333 0.000093<br>H 1.724973 2.184304 -0.000191<br>H 3.261545 -0.000030 -0.000141<br>H 1.724934 -2.184336 -0.000006	C 2.482530 0.000475 0.311887<br>C 1.884749 1.264089 0.141904<br>C 0.558009 1.591527 0.006893<br>C 1.872295 -1.267445 0.256735<br>C -0.578611 0.728217 0.067515<br>C 0.542324 -1.592623 0.151272<br>C -0.585685 -0.716249 0.133118<br>H 3.563423 -0.001359 0.389492<br>H 2.582146 2.098818 0.109285<br>H 0.333775 2.647172 -0.124141<br>H 2.561428 -2.108499 0.300276<br>H 0.307646 -2.653543 0.116472<br>C -1.916045 1.145924 0.017341<br>H -2.240617 2.177111 -0.036699<br>C -2.747491 0.014260 0.048820<br>H -3.828334 0.019037 0.036447<br>C -1.927239 -1.123589 0.120915<br>H -2.261870 -2.152210 0.160805	C 0.758432 0.672883 0.011522<br>C 0.752996 -0.681559 -0.074387<br>C -0.749773 0.710401 0.013385<br>C -1.898194 1.429901 0.059099<br>C -3.115968 0.685156 0.037488<br>C -3.116196 -0.687524 -0.027579<br>C -1.902158 -1.436156 -0.074284<br>C -0.755238 -0.712957 -0.055060<br>H -1.917993 2.512296 0.103063<br>H -4.059825 1.211874 0.073918<br>H -4.060390 -1.213643 -0.053643<br>H -1.932607 -2.516472 -0.123340<br>H 1.536959 1.416096 0.078015<br>H 1.530036 -1.426754 -0.135921	C 0.756711 0.670285 -0.000015<br>C 0.756711 -0.670281 -0.000041<br>C -0.761855 0.707604 0.000046<br>C -1.898683 1.436999 0.000108<br>C -3.112632 0.683989 0.000132<br>C -3.112632 -0.683986 0.000094<br>C -1.898683 -1.436996 0.000029<br>C -0.761855 -0.707600 0.000008<br>H -1.918679 2.517858 0.000136<br>H -4.056021 1.212812 0.000180<br>H -4.056021 -1.212809 0.000114<br>H -1.918679 -2.517854 0.000003<br>H 1.534128 1.418504 -0.000028<br>H 1.534128 -1.418500 -0.000084
25	26	27	28
C 2.465729 1.398410 -0.000202<br>C 1.214084 0.714764 -0.000094	C 2.482530 0.000475 0.311887<br>C 1.884749 1.264089 0.141904	C -0.099924 1.283069 0.306312<br>C -1.317595 0.789294 -0.123195	C 2.484739 0.000046 -0.000010<br>C 1.897805 -1.258189 -0.000069

C 3.635139 0.710899 -0.000293<br>C 0.000025 1.393362 0.000001<br>C 1.214084 -0.714764 -0.000084<br>C 3.635139 -0.710900 -0.000283<br>C 0.000025 -1.393363 0.000021<br>C -1.213860 0.714800 0.000105<br>C 2.465728 -1.398410 -0.000181<br>C -2.465659 1.398452 0.000203<br>C -1.213860 -0.714800 0.000115<br>C -2.465659 -1.398452 0.000224<br>C -3.634968 0.710973 0.000304<br>C -3.634968 -0.710973 0.000315<br>H 2.464181 2.480983 -0.000210<br>H 0.000089 2.477063 -0.000007<br>H 0.000089 -2.477064 0.000029<br>H 2.464180 -2.480984 -0.000173<br>H -2.464008 2.481027 0.000195<br>H -4.577221 1.241761 0.000378<br>H -2.464008 -2.481027 0.000232<br>H -4.577221 -1.241761 0.000397<br>H 4.577322 -1.241809 -0.000356<br>H 4.577322 1.241808 -0.000374	C 0.558009 1.591527 0.006893<br>C 1.872295 -1.267445 0.256735<br>C -0.578611 0.728217 0.067515<br>C 0.542324 -1.592623 0.151272<br>C -0.585685 -0.716249 0.133118<br>H 3.563423 -0.001359 0.389492<br>H 2.582146 2.098818 0.109285<br>H 0.333775 2.647172 -0.124141<br>H 2.561428 -2.108499 0.300276<br>H 0.307646 -2.653543 0.116472<br>C -1.916045 1.145924 0.017341<br>H -2.240617 2.177111 -0.036699<br>C -2.747491 0.014260 0.048820<br>H -3.828334 0.019037 0.036447<br>C -1.927239 -1.123589 0.120915<br>H -2.261870 -2.152210 0.160805	C -1.200465 -0.587869 -0.095165<br>C 0.062393 -1.289480 -0.287123<br>C 1.280060 -0.795707 0.142400<br>C 1.162913 0.581456 0.114320<br>H -0.076573 2.186861 0.925105<br>H -2.256756 1.317344 -0.009486<br>H -2.064771 -1.193052 0.199789<br>H 0.039066 -2.193260 -0.905933<br>H 2.219223 -1.323753 0.028699<br>H 2.027216 1.186624 -0.180682	C 0.549131 -1.585058 -0.000086<br>C 1.897771 1.258232 0.000051<br>C -0.549687 -0.744288 -0.000049<br>C 0.549057 1.585064 0.000072<br>C -0.549711 0.744275 0.000041<br>H 3.568839 0.000059 -0.000019<br>H 2.584409 -2.095469 -0.000075<br>H 0.319758 -2.645876 -0.000035<br>H 2.584330 2.095549 0.000061<br>H 0.319669 2.645880 0.000044<br>C -1.888438 -1.142627 0.000241<br>H -2.232357 -2.165019 0.000428<br>C -2.688680 -0.000034 -0.000119<br>H -3.768697 -0.000062 -0.000178<br>C -1.888504 1.142568 -0.000083<br>H -2.232450 2.164951 -0.000157
29<br>C 0.000000 2.819086 0.871687<br>C 0.000000 3.537247 -0.295171<br>C -0.000000 1.412900 0.857372<br>C -0.000000 2.861752 -1.520619<br>C 0.000000 0.726489 -0.375034<br>C 0.000000 0.673037 2.083664<br>C 0.000000 1.489931 -1.556445<br>C 0.000000 -0.726489 -0.375034<br>C -0.000000 -0.673037 2.083664<br>C -0.000000 -1.489931 -1.556445<br>C 0.000000 -1.412900 0.857372<br>C -0.000000 -2.819086 0.871687<br>C 0.000000 -2.861752 -1.520619<br>C -0.000000 -3.537247 -0.295171<br>H 0.000000 3.328871 1.826841<br>H 0.000000 1.223221 3.015964<br>H -0.000000 0.998790 -2.518260<br>H -0.000000 -1.223221 3.015964<br>H -0.000000 -3.328871 1.826841<br>H -0.000000 -4.618284 -0.271340<br>H 0.000000 -0.998790 -2.518260<br>H 0.000000 -3.422385 -2.445418<br>H -0.000000 3.422385 -2.445418<br>H 0.000000 4.618284 -0.271340	30<br>C 2.484739 0.000046 -0.000010<br>C 1.897805 -1.258189 -0.000069<br>C 0.549131 -1.585058 -0.000086<br>C 1.897771 1.258232 0.000051<br>C -0.549687 -0.744288 -0.000049<br>C 0.549057 1.585064 0.000072<br>C -0.549711 0.744275 0.000041<br>H 3.568839 0.000059 -0.000019<br>H 2.584409 -2.095469 -0.000075<br>H 0.319758 -2.645876 -0.000035<br>H 2.584330 2.095549 0.000061<br>H 0.319669 2.645880 0.000044<br>C -1.888438 -1.142627 0.000241<br>H -2.232357 -2.165019 0.000428<br>C -2.688680 -0.000034 -0.000119<br>H -3.768697 -0.000062 -0.000178<br>C -1.888504 1.142568 -0.000083<br>H -2.232450 2.164951 -0.000157	31<br>C 0.677658 0.716855 -0.000008<br>C 0.677658 -0.716851 -0.000042<br>C -0.739269 0.741428 0.000045<br>C -1.892353 1.504627 0.000107<br>C -3.039606 0.724992 0.000129<br>C -3.039606 -0.724989 0.000092<br>C -1.892353 -1.504623 0.000029<br>C -0.739269 -0.741424 0.000007<br>H -1.917855 2.586479 0.000136<br>H -4.009356 1.211740 0.000177<br>H -4.009355 -1.211737 0.000115<br>H -1.917854 -2.586476 0.000002<br>H 1.463749 1.466393 -0.000019<br>H 1.463750 -1.466389 -0.000088	32<br>C 0.040167 0.359410 0.491476<br>C 1.359844 0.225046 -0.012586<br>C 1.359806 1.610720 0.012580<br>C 0.040207 1.476289 -0.491666<br>H 1.972706 -0.562185 -0.420627<br>H 1.972577 2.397986 0.420690
33<br>C -0.000000 1.374209 2.483994<br>C 0.000000 0.702751 1.250925<br>C 0.000000 1.434815 -0.016920<br>C 0.000000 2.838425 -0.051806<br>C 0.000000 -0.702751 1.250925<br>C 0.000000 0.732071 -1.234211<br>C 0.000000 -0.732071 -1.234211<br>C 0.000000 -1.434815 -0.016920<br>C 0.000000 -1.464367 -2.432239<br>C 0.000000 1.464367 -2.432239<br>C -0.000000 2.837459 -2.441768<br>C 0.000000 3.533386 -1.236094<br>H 0.000000 4.614643 -1.227820<br>H -0.000000 3.371171 -3.382168<br>C -0.000000 0.696056 3.678027<br>C 0.000000 -0.696056 3.678027<br>C 0.000000 -1.374209 2.483994<br>H -0.000000 1.243871 4.610280<br>H 0.000000 -1.243871 4.610280<br>C -0.000000 -2.838425 -0.051806<br>C -0.000000 -2.837459 -2.441768<br>H 0.000000 -3.371171 -3.382168<br>C -0.000000 -3.533386 -1.236094<br>H -0.000000 -4.614643 -1.227820<br>H 0.000000 0.950665 -3.380708<br>H 0.000000 -0.950665 -3.380708<br>H -0.000000 -3.402916 0.867380<br>H 0.000000 -2.452484 2.513126<br>H -0.000000 2.452484 2.513126<br>H -0.000000 3.402916 0.867380	34<br>C 1.367617 -0.747964 0.005606<br>C 1.389268 0.639907 0.089199<br>C 0.385282 1.495677 -0.518235<br>C 0.339607 -1.623406 -0.325703<br>C -0.890233 1.208716 -0.825422<br>C -1.064956 -1.253422 -0.311774<br>C -1.615540 -0.037057 -0.455371<br>H 2.318780 -1.232043 0.239535<br>H 2.334401 1.107034 0.340581<br>H 0.736831 2.485426 -0.817257<br>H 0.560388 -2.683928 -0.290633<br>H -1.463018 1.955647 -1.369706<br>H -1.758782 -2.068799 -0.096477<br>H -2.690124 0.054679 -0.316984	35<br>C 0.737589 -1.242446 -0.014287<br>C -0.737590 1.242446 -0.014287<br>C 1.434425 -0.000000 0.000000<br>C -0.737590 -1.242446 0.014287<br>C -1.434425 -0.000000 -0.000000<br>C 0.737589 1.242446 0.014287<br>C -2.854283 -0.000000 -0.000000<br>C -3.552944 1.226140 -0.031323<br>C -2.869521 2.404750 -0.062029<br>C -1.468039 2.408420 -0.052674<br>C 1.468038 2.408420 0.052675<br>C 2.869520 2.404750 0.062030<br>C 3.552944 1.226140 0.031323<br>C 2.854283 -0.000000 0.000000<br>H -4.635019 1.211825 -0.032811<br>H -3.401867 3.345570 -0.091498<br>H -0.968280 3.364808 -0.077741<br>H 0.968279 3.364808 0.077741<br>H 3.401867 3.345570 0.091498<br>H 4.635018 1.211825 0.032811<br>C -3.552944 -1.226141 0.031323<br>H -4.635019 -1.211825 0.032811<br>C -1.468038 -2.408421 0.052674<br>H -0.968279 -3.364809 0.077741<br>C -2.869521 -2.404750 0.062029<br>H -3.401867 -3.345571 0.091498<br>C 3.552944 -1.226141 -0.031323<br>H 4.635018 -1.211825 -0.032811<br>C 1.468038 -2.408421 -0.052674<br>H 0.968279 -3.364809 -0.077741<br>C 2.869520 -2.404750 -0.062029<br>H 3.401867 -3.345571 -0.091498	36<br>C 0.677658 0.716855 -0.000008<br>C 0.677658 -0.716851 -0.000042<br>C -0.739269 0.741428 0.000045<br>C -1.892353 1.504627 0.000107<br>C -3.039606 0.724992 0.000129<br>C -3.039606 -0.724989 0.000092<br>C -1.892353 -1.504623 0.000029<br>C -0.739269 -0.741424 0.000007<br>H -1.917855 2.586479 0.000136<br>H -4.009356 1.211740 0.000177<br>H -4.009355 -1.211737 0.000115<br>H -1.917854 -2.586476 0.000002<br>H 1.463749 1.466393 -0.000019<br>H 1.463750 -1.466389 -0.000088
37<br>C 0.619107 -1.224329 0.000129<br>C -0.617155 -1.224516 -0.000022<br>C -1.453783 -0.131452 -0.000006<br>C -0.700434 1.050695 0.000008<br>H -2.533124 -0.133832 0.000022<br>H -1.223135 1.998530 0.000015<br>C 1.453778 -0.129523 -0.000036<br>H 2.533144 -0.130266 -0.000017<br>C 0.698906 1.051592 -0.000003<br>H 1.220336 2.000125 0.000036	38<br>C 2.518852 -0.000000 0.000008<br>C 1.918672 1.254063 -0.000005<br>C 0.544537 1.588674 -0.000008<br>C 1.918672 -1.254064 0.000017<br>C -0.497437 0.706006 -0.000007<br>C 0.544537 -1.588674 0.000012<br>C -0.497437 -0.706006 -0.000004<br>H 3.602462 -0.000000 0.000015<br>H 2.598551 2.099421 -0.000014<br>H 0.312395 2.647814 -0.000029<br>H 2.598551 -2.099421 0.000036<br>H 0.312395 -2.647814 0.000037<br>C -1.933494 1.111722 -0.000069<br>H -2.266525 2.144538 -0.000048	39<br>C -1.545198 -0.673036 0.386472<br>C -0.657267 -1.552001 -0.386392<br>C 0.657254 1.552005 -0.386418<br>C 0.673029 -1.545213 -0.386453<br>C 1.545232 0.673035 0.386385<br>C 1.552019 -0.657262 0.386357<br>H -2.315144 -1.181407 0.958508<br>H -1.157823 -2.327084 -0.958385<br>H 1.157776 2.327092 -0.958435<br>H 1.181381 -2.315174 -0.958485<br>H 2.315213 1.181400 0.958377<br>H 2.327122 -1.157802 0.958337<br>C -1.551986 0.657259 0.386456<br>H -2.327053 1.157796 0.958486	40<br>C -1.287430 1.101692 -0.000088<br>C -2.108350 -0.145385 -0.000079<br>C -1.299933 -1.218100 -0.000007<br>C 0.079606 -0.710187 0.000015<br>C 0.005045 0.742659 -0.000039<br>C 1.372082 -1.069227 0.000060<br>C 2.192996 0.177828 0.000148<br>C 1.384594 1.250560 -0.000008<br>H -1.700540 2.099836 -0.000124<br>H -3.187245 -0.157894 -0.000125<br>H -1.601814 -2.252663 0.000008<br>H 1.785152 -2.067387 0.000105<br>H 3.271892 0.190371 0.000237<br>H 1.686506 2.285112 -0.000041

	C -2.753812 0.000000 0.000001<br>H -3.833210 0.000001 0.000028<br>C -1.933494 -1.111722 0.000026<br>H -2.266526 -2.144538 0.000070	C -0.673042 1.545217 -0.386401<br>H -1.181427 2.315183 -0.958397	
41<br>C 0.361192 1.424124 0.000111<br>C -0.773073 0.752626 -0.000109<br>C -1.413938 -0.399264 0.000108<br>C -0.265282 -1.045902 -0.000109<br>C 1.052708 -1.024846 0.000111<br>C 1.038393 0.293264 -0.000111	42<br>C 0.758432 0.672883 0.011522<br>C 0.752996 -0.681559 -0.074387<br>C -0.749773 0.710401 0.013385<br>C -1.898194 1.429901 0.059099<br>C -3.115968 0.685156 0.037488<br>C -3.116196 -0.687524 -0.027579<br>C -1.902158 -1.436156 -0.074284<br>C -0.755238 -0.712957 -0.055060<br>H -1.917993 2.512296 0.103063<br>H -4.059825 1.211874 0.073918<br>H -4.060390 -1.213643 -0.053643<br>H -1.932607 -2.516472 -0.123340<br>H 1.536959 1.416096 0.078015<br>H 1.530036 -1.426754 -0.135921	43<br>C -1.425879 1.152618 -0.000112<br>C -2.192923 -0.146684 -0.000076<br>C -1.345294 -1.190051 -0.000051<br>C 0.012736 -0.674341 0.000008<br>C -0.012736 0.674341 -0.000026<br>C 1.425880 -1.152618 0.000129<br>C 2.192923 0.146685 0.000066<br>C 1.345294 1.190051 0.000065<br>H -1.666539 1.760286 -0.876990<br>H -1.666605 1.760357 0.876698<br>H -3.270704 -0.200272 -0.000101<br>H -1.629019 -2.232073 -0.000039<br>H 1.666520 -1.760263 0.877030<br>H 1.666625 -1.760380 -0.876658<br>H 3.270704 0.200272 0.000082<br>H 1.629019 2.232073 0.000060	44<br>C -0.321590 0.072535 0.208994<br>C 0.913191 -0.217852 0.238683<br>C 1.310016 1.055522 0.139063<br>C 0.974603 2.277583 0.039701<br>C -0.444194 2.317617 0.029020<br>C -1.195139 1.118529 0.120922<br>H -1.001828 3.243907 -0.047851<br>H -2.269578 1.075237 0.118813
45<br>C -0.346504 -0.507627 -1.172281<br>H -0.157061 -0.243208 -2.200979<br>C -1.082235 -1.535880 -0.732080<br>H -1.599014 -2.258517 -1.346065<br>C -0.346526 -0.507598 1.172318<br>H -0.157112 -0.243147 2.201013<br>C -1.082315 -1.535814 0.732128<br>H -1.599148 -2.258405 1.346122<br>C 0.194422 0.249813 0.000014<br>H 1.288691 0.274908 0.000028<br>H -0.136468 1.293179 -0.000006	46<br>C 3.101773 -0.689642 -0.000124<br>C 3.101773 0.689647 -0.000090<br>C 1.903530 1.436127 -0.000031<br>C 0.753172 0.705979 -0.000011<br>C 0.753172 -0.705976 -0.000046<br>C 1.903530 -1.436123 -0.000103<br>H 4.047191 -1.214752 -0.000169<br>H 4.047191 1.214757 -0.000108<br>H 1.923849 2.516703 -0.000004<br>H 1.923850 -2.516699 -0.000130<br>C -0.753172 0.705979 0.000043<br>C -1.903530 1.436126 0.000105<br>C -3.101772 0.689646 0.000130<br>C -3.101772 -0.689643 0.000095<br>C -1.903529 -1.436123 0.000032<br>C -0.753172 -0.705976 0.000007<br>H -1.923849 2.516703 0.000134<br>H -4.047191 1.214756 0.000179<br>H -4.047191 -1.214753 0.000117<br>H -1.923849 -2.516700 0.000005	47<br>C 0.000000 2.824858 -0.000000<br>C -0.000000 1.482693 -0.000000<br>C -1.284220 0.741377 -0.000101<br>C -2.446529 1.412508 -0.000255<br>C 1.284220 0.741377 0.000101<br>C -1.284220 -0.741377 0.000101<br>C -0.000000 -1.482693 -0.000000<br>C 1.284220 -0.741377 -0.000101<br>C -0.000000 -2.824858 -0.000000<br>C -2.446529 -1.412508 0.000255<br>C 2.446529 1.412508 0.000255<br>C 2.446529 -1.412508 -0.000255<br>C -3.769023 -0.733763 0.191295<br>H -4.545228 -1.268621 -0.360802<br>H -4.032513 -0.847949 1.250731<br>C -3.769023 0.733763 -0.191295<br>H -4.545228 1.268621 0.360802<br>H -4.032513 0.847949 -1.250731<br>C -2.520061 -2.897223 -0.190536<br>H -3.371037 -3.301891 0.362167<br>H -2.751344 -3.068496 -1.249798<br>C -1.248919 -3.630881 0.191441<br>H -1.173951 -4.570421 -0.360859<br>H -1.281269 -3.916360 1.250837<br>C 1.248919 -3.630881 -0.191441<br>H 1.173951 -4.570421 0.360859<br>H 1.281269 -3.916360 -1.250837<br>C 2.520061 -2.897223 0.190536<br>H 3.371037 -3.301891 -0.362167<br>H 2.751344 -3.068496 1.249798<br>C 3.769023 -0.733763 -0.191295<br>H 4.545228 -1.268621 0.360802<br>H 4.032513 -0.847949 -1.250731<br>C 3.769023 0.733763 0.191295<br>H 4.545228 1.268621 -0.360802<br>H 4.032513 0.847949 1.250731<br>C 2.520061 2.897223 -0.190536<br>H 3.371037 3.301891 0.362167<br>H 2.751344 3.068496 -1.249798<br>C 1.248919 3.630881 0.191441<br>H 1.173951 4.570421 -0.360859<br>H 1.281269 3.916360 1.250837<br>C -1.248919 3.630881 -0.191441<br>H -1.173951 4.570421 0.360859<br>H -1.281269 3.916360 -1.250837<br>C -2.520061 2.897223 0.190536<br>H -3.371037 3.301891 -0.362167<br>H -2.751344 3.068496 1.249798	48<br>C -0.102560 -0.037878 1.150082<br>H -0.154042 0.030657 2.226935<br>C 0.236834 1.170880 0.217964<br>H 0.445506 2.167265 0.579406<br>C 0.215032 0.736279 -1.046715<br>H 0.400459 1.290744 -1.950340<br>C -0.303613 -1.111222 0.377987<br>H -0.555439 -2.114434 0.675453<br>C -0.114653 -0.665006 -0.979309<br>H -0.209766 -1.308547 -1.848468
49<br>C 0.000000 2.824858 -0.000000<br>C -0.000000 1.482693 -0.000000<br>C -1.284220 0.741377 -0.000101<br>C -2.446529 1.412508 -0.000255<br>C 1.284220 0.741377 0.000101<br>C -1.284220 -0.741377 0.000101<br>C -0.000000 -1.482693 -0.000000<br>C 1.284220 -0.741377 -0.000101<br>C -0.000000 -2.824858 -0.000000<br>C -2.446529 -1.412508 0.000255<br>C 2.446529 1.412508 0.000255<br>C 2.446529 -1.412508 -0.000255<br>C -3.769023 -0.733763 0.191295<br>H -4.545228 -1.268621 -0.360802<br>H -4.032513 -0.847949 1.250731<br>C -3.769023 0.733763 -0.191295<br>H -4.545228 1.268621 0.360802<br>H -4.032513 0.847949 -1.250731<br>C -2.520061 -2.897223 -0.190536<br>H -3.371037 -3.301891 0.362167<br>H -2.751344 -3.068496 -1.249798<br>C -1.248919 -3.630881 0.191441<br>H -1.173951 -4.570421 -0.360859<br>H -1.281269 -3.916360 1.250837	50<br>C 0.756711 0.670285 -0.000015<br>C 0.756711 -0.670281 -0.000041<br>C -0.761855 0.707604 0.000046<br>C -1.898683 1.436999 0.000108<br>C -3.112632 0.683989 0.000132<br>C -3.112632 -0.683986 0.000094<br>C -1.898683 -1.436996 0.000029<br>C -0.761855 -0.707600 0.000008<br>H -1.918679 2.517858 0.000136<br>H -4.056021 1.212812 0.000180<br>H -4.056021 -1.212809 0.000114<br>H -1.918679 -2.517854 0.000003<br>H 1.534128 1.418504 -0.000028<br>H 1.534128 -1.418500 -0.000084	51<br>C -0.830111 -0.135990 0.022146<br>C 1.332586 1.112523 0.022040<br>C 0.670265 2.259716 0.021253<br>C -1.492432 1.011203 0.021358<br>H -1.379231 -1.070178 0.022646<br>H 2.416170 1.120916 0.022461<br>H 1.219387 3.193903 0.021036<br>H -2.576016 1.002811 0.021221<br>C 0.665513 -0.229356 0.022404<br>H 0.999564 -0.808043 0.890662<br>H 0.999810 -0.808616 -0.845384<br>C -0.825358 2.353082 0.020632<br>H -1.159206 2.931416 -0.847945<br>H -1.159859 2.932693 0.888100	52<br>C 0.109973 -1.414540 -0.063644<br>C 0.110981 1.414464 0.063625<br>C 1.248807 0.722880 0.110543<br>C 1.248292 -0.723767 -0.110562<br>H 0.111359 -2.489433 -0.191423<br>H 0.113131 2.489356 0.191405<br>H 2.191361 1.222735 0.291315<br>H 2.190489 -1.224293 -0.291333<br>C -1.187141 0.722700 -0.249050<br>H -2.028680 1.267560 0.178438<br>H -1.330615 0.736959 -1.336991<br>C -1.187656 -0.721851 0.249033<br>H -2.029583 -1.266111 -0.178456<br>H -1.331140 -0.736010 1.336974

C 1.248919 -3.630881 -0.191441<br>H 1.173951 -4.570421 0.360859<br>H 1.281269 -3.916360 -1.250837<br>C 2.520061 -2.897223 0.190536<br>H 3.371037 -3.301891 -0.362167<br>H 2.751344 -3.068496 1.249798<br>C 3.769023 -0.733763 -0.191295<br>H 4.545228 -1.268621 0.360802<br>H 4.032513 -0.847949 -1.250731<br>C 3.769023 0.733763 0.191295<br>H 4.545228 1.268621 -0.360802<br>H 4.032513 0.847949 1.250731<br>C 2.520061 2.897223 -0.190536<br>H 3.371037 3.301891 0.362167<br>H 2.751344 3.068496 -1.249798<br>C 1.248919 3.630881 0.191441<br>H 1.173951 4.570421 -0.360859<br>H 1.281269 3.916360 1.250837<br>C -1.248919 3.630881 -0.191441<br>H -1.173951 4.570421 0.360859<br>H -1.281269 3.916360 -1.250837<br>C -2.520061 2.897223 0.190536<br>H -3.371037 3.301891 -0.362167<br>H -2.751344 3.068496 1.249798			
53<br>C 0.502655 0.017460 -0.000000<br>C 2.000660 -0.119362 -0.000000<br>C 1.153873 1.123907 -0.000000<br>H -0.434173 -0.506818 0.000000<br>H 2.511760 -0.419880 -0.911580<br>H 2.511760 -0.419880 0.911580<br>H 1.158011 2.197432 0.000000	54<br>C 0.366202 -0.079567 -0.000000<br>C 1.866212 -0.079470 0.000000<br>C 1.116125 1.219532 0.000000<br>H -0.140241 -0.371973 -0.908349<br>H -0.140241 -0.371973 0.908349<br>H 2.372696 -0.371804 -0.908349<br>H 2.372696 -0.371804 0.908349<br>H 1.116095 1.804335 0.908358<br>H 1.116095 1.804335 -0.908358	55<br>H -1.232103 2.036808 -0.055496<br>C -0.707240 1.136816 0.267022<br>C -0.651196 -1.356226 -0.025051<br>C 1.482344 -0.094271 0.245775<br>C 0.659932 -1.349638 0.178777<br>C 0.716978 1.118442 -0.279111<br>C -1.472881 -0.107723 -0.176522<br>H -1.174540 -2.303690 -0.084766<br>H 1.792323 0.077998 1.282118<br>H 1.182700 -2.291171 0.302897<br>H 0.678978 1.073961 -1.371205<br>H -1.781707 -0.004537 -1.222341<br>H -0.669404 1.164732 1.359674<br>H 2.405940 -0.232696 -0.320577<br>H 1.242187 2.037618 -0.016954<br>H -2.397118 -0.207535 0.396845	56<br>C 1.771282 0.411333 -0.000048<br>C 2.373961 1.455110 0.000010<br>C 0.373446 2.681432 -0.001310<br>C -0.290934 1.530672 -0.001149<br>H -0.192638 3.606098 -0.001858<br>H -1.374755 1.558578 -0.001586<br>C 0.330164 0.123876 -0.000371<br>H -0.000117 -0.436348 -0.876881<br>H -0.000614 -0.435574 0.876447<br>C 1.902342 2.846891 -0.000805<br>H 2.221483 3.413551 0.875691<br>H 2.222133 3.412691 -0.877623
57<br>C 0.562962 -1.259455 -0.100784<br>C -0.646956 -1.289518 -0.086132<br>C -1.537155 -0.156365 0.050032<br>C -0.854956 1.000059 0.070611<br>H -2.607052 -0.213988 0.179027<br>H -1.393852 1.931258 0.200907<br>C 1.503063 -0.164762 0.189958<br>H 1.780946 -0.183916 1.246116<br>H 2.420764 -0.155526 -0.393900<br>C 0.638328 1.105341 -0.160873<br>H 0.776106 1.307129 -1.228715<br>H 1.041154 1.970137 0.368009	58<br>C -0.000026 -1.220249 -0.137713<br>C 1.227509 -0.318951 0.104351<br>C -1.227520 -0.318894 0.104346<br>C 0.662630 1.068408 -0.044863<br>C -0.662576 1.068440 -0.044856<br>H -0.000032 -1.555117 -1.175397<br>H -0.000048 -2.109050 0.490686<br>H 2.039014 -0.524161 -0.594961<br>H 1.639565 -0.453386 1.109297<br>H -1.639588 -0.453315 1.109289<br>H -2.039032 -0.524064 -0.594971<br>H 1.281918 1.953134 -0.101859<br>H -1.281822 1.953195 -0.101848	59<br>C 0.006536 0.491717 -0.000049<br>C 1.514025 0.375640 -0.000035<br>C 1.513991 1.938861 -0.000080<br>C 0.006507 1.822719 -0.000241<br>H -0.778043 -0.252603 -0.000022<br>H 1.954147 -0.080201 -0.887403<br>H 1.954106 -0.080124 0.887390<br>H 1.954153 2.394642 -0.887456<br>H 1.953993 2.394722 0.887337<br>H -0.778104 2.567005 -0.000355	60<br>C -0.745194 0.692433 0.000120<br>C -1.909808 1.429915 0.000260<br>C -3.096109 0.696490 0.000195<br>C -3.096109 -0.696487 -0.000010<br>C -1.909808 -1.429912 -0.000140<br>C -0.745194 -0.692429 -0.000070<br>H -1.925500 2.511652 0.000456<br>H -4.043807 1.218494 0.000313<br>H -4.043806 -1.218491 -0.000074<br>H -1.925499 -2.511649 -0.000333<br>C 0.767200 -0.786270 -0.000133<br>H 1.208919 -1.239382 0.886644<br>H 1.209030 -1.239243 -0.886925<br>C 0.767200 0.786274 0.000112<br>H 1.208879 1.239387 -0.886684<br>H 1.209069 1.239246 0.886885
61<br>H -0.028346 2.247964 0.178268<br>C 0.092223 1.273858 -0.297909<br>C 0.049243 -1.402467 -0.025855<br>C -1.280968 0.533599 -0.286315<br>H -1.969485 1.033020 0.398235<br>H 0.387031 1.465626 -1.330035<br>H -1.737253 0.590110 -1.274892<br>C 1.268528 0.516523 0.386426<br>H 1.126184 0.487695 1.465777<br>H 2.206684 1.033398 0.185620<br>C -1.137699 -0.901381 0.212133<br>C 1.236809 -0.878910 -0.213198<br>H 1.970117 -1.208753 -0.935818<br>H -1.877235 -1.322586 0.878640	62<br>C -0.032223 0.494209 -0.000078<br>C 1.535534 0.494235 0.000059<br>C 1.535512 1.820274 -0.000057<br>C -0.032245 1.820248 -0.000193<br>H -0.794436 -0.269001 -0.000078<br>H 2.297772 -0.268950 0.000193<br>H 2.297725 2.583484 -0.000057<br>H -0.794483 2.583433 -0.000328	63<br>H -2.469594 -0.260662 -0.140808<br>C -1.448784 -0.152919 0.229538<br>C 0.856853 -1.178206 0.229533<br>C 0.591918 1.331103 0.229572<br>C 1.448784 0.152919 -0.229538<br>C -0.856853 1.178206 -0.229533<br>C -0.591918 -1.331103 -0.229572<br>H 0.891812 -1.226313 1.322580<br>H 0.616117 1.385473 1.322611<br>H 1.508036 0.159182 -1.322580<br>H -0.891812 1.226313 -1.322580<br>H -0.616117 -1.385473 -1.322611<br>H -1.508036 -0.159182 1.322580<br>H 1.460572 -2.008425 -0.140680<br>H 1.008997 2.269042 -0.140730<br>H 2.469594 0.260662 0.140808<br>H -1.460572 2.008425 0.140680<br>H -1.008997 -2.269042 0.140730	64<br>C 0.306959 -1.508826 -0.404699<br>C 1.535946 -0.755560 0.108433<br>C 1.535920 0.755608 -0.108438<br>C -0.960082 -1.235270 0.411164<br>C 0.306912 1.508836 0.404700<br>C -1.769055 -0.000030 -0.000004<br>C -0.960124 1.235244 -0.411158<br>H 0.136364 -1.276685 -1.460454<br>H 1.635145 -0.961312 1.179077<br>H 1.635107 0.961362 -1.179083<br>H 0.136330 1.276690 1.460456<br>H -0.670669 -1.145958 1.461757<br>H -2.427477 0.263986 0.830981<br>H 0.526419 -2.577281 -0.361391<br>H 2.431042 -1.167510 -0.363438<br>H 2.431005 1.167587 0.363428<br>H -1.622012 -2.101722 0.361188<br>H 0.526340 2.577296 0.361391<br>H -2.427450 -0.264075 -0.831001<br>H -1.622082 2.101673 -0.361166<br>H -0.670712 1.145957 -1.461752
65<br>C -0.603445 -1.128990 -0.000039<br>C 0.602898 -1.129010 0.000017<br>C 0.603446 1.129049 -0.000015<br>C -0.602896 1.129057 -0.000006<br>C -1.611345 0.000288 0.000035<br>H -2.251130 0.000456 -0.880130<br>H -2.251001 0.000428 0.880295<br>C 1.611341 -0.000230 -0.000035<br>H 2.251112 -0.000372 0.880142<br>H 2.251014 -0.000400 -0.880282	66<br>C -0.000000 -0.000000 1.769304<br>H 0.622018 0.622018 2.418319<br>H -0.622018 -0.622018 2.418319<br>C 0.886587 -0.886587 0.886587<br>H 1.516840 -1.516840 1.516840<br>C -0.886587 0.886587 0.886587<br>H -1.516840 1.516840 1.516840<br>C 0.000000 1.769304 0.000000<br>H 0.622018 2.418319 0.622018<br>H -0.622018 2.418319 -0.622018<br>C 1.769304 0.000000 -0.000000<br>H 2.418319 -0.622018 -0.622018<br>H 2.418319 0.622018 0.622018<br>C 0.886587 0.886587 -0.886587	67<br>C -0.625603 -0.195226 1.739600<br>C -0.221696 1.219335 1.305746<br>C -0.288674 -1.261742 -1.089197<br>C -0.676060 1.689156 -0.087054<br>C -0.145231 -0.029512 -1.985390<br>C 0.193819 1.289398 -1.285287<br>H -1.614466 -0.414973 1.329084<br>H 0.863298 1.339524 1.389981<br>H -1.710252 1.371182 -0.254629<br>H -1.256213 -1.221877 -0.586526<br>H -1.075118 0.098921 -2.545915<br>H -0.758575 -0.200803 2.823519<br>H -0.652294 1.914932 2.028665<br>H -0.331981 -2.144392 -1.728700	68<br>C -1.517639 -0.000013 -0.000017<br>C 0.658115 1.216498 0.385271<br>C 0.658132 -1.216495 -0.385258<br>C 1.517638 0.000013 -0.000004<br>C -0.658118 -1.216513 0.385219<br>C -0.658130 1.216511 -0.385214<br>H -2.171117 0.260020 0.834649<br>H -2.171062 -0.260069 -0.834720<br>H -1.211411 2.139021 -0.207471<br>H 0.438276 -1.192183 -1.455801<br>H 1.211382 2.139026 0.207584<br>H -0.438269 1.192246 -1.455757<br>H 0.438256 1.192164 1.455813<br>H 2.171105 -0.260055 0.834660

H 1.516840 1.516840 -1.516840
C 0.000000 -0.000000 -1.769304
H -0.622018 0.622018 -2.418319
H 0.622018 -0.622018 -2.418319
C -0.000000 -1.769304 0.000000
H -0.622018 -2.418319 0.622018
H 0.622018 -2.418319 -0.622018
C -1.769304 0.000000 -0.000000
H -2.418319 -0.622018 0.622018
H -2.418319 0.622018 -0.622018
C -0.886587 -0.886587 -0.886587
H -1.516840 -1.516840 -1.516840

H -0.704269 2.780131 -0.057622
H 0.632679 -0.218523 -2.730407
H 0.125153 2.074964 -2.040766
H 1.240073 1.287380 -0.968994
C 0.345007 -1.331780 1.406495
H -0.142654 -2.273047 1.675130
H 1.219991 -1.248789 2.056752
C 0.827419 -1.415302 -0.043636
H 1.588824 -0.651134 -0.207122
H 1.341967 -2.367877 -0.176818

H 2.171073 0.260099 -0.834688
H -1.211385 -2.139034 0.207494
H -0.438267 -1.192218 1.455763
H 1.211411 -2.139012 -0.207552

69

C 0.602751 -1.287514 -0.025726
C -0.602776 -1.287508 0.025816
C -1.584254 -0.192334 0.117020
H -1.969063 -0.098793 1.133442
H -2.438936 -0.287154 -0.551135
C 1.584264 -0.192379 -0.116970
H 1.969069 -0.098884 -1.133399
H 2.438946 -0.287200 0.551184
C 0.715353 1.048017 0.282566
H 1.225301 1.955285 -0.047493
H 0.667438 1.086335 1.373012
C -0.715311 1.048025 -0.282559
H -0.667394 1.086301 -1.373007
H -1.225236 1.955318 0.047465

70

C 0.162236 1.292816 0.116425
C -1.148869 0.554787 -0.207952
C -0.863823 -0.900521 0.161125
C 0.574265 -1.095626 -0.315037
C 1.276440 0.216399 0.070993
H 0.348668 2.106489 -0.583207
H 0.108589 1.738753 1.109739
H -1.360985 0.619169 -1.277559
H -2.007205 0.971542 0.317780
H -0.917272 -1.028604 1.245720
H -1.565044 -1.602886 -0.288720
H 1.055844 -1.973287 0.114831
H 0.580243 -1.220219 -1.400463
H 1.741770 0.115256 1.051949
H 2.070325 0.473310 -0.628890

71

C 1.024185 0.334824 0.127426
C 0.334815 -1.024191 -0.127405
C -0.334818 1.024176 -0.127510
C -1.024182 -0.334810 0.127489
H 1.332771 0.435842 1.167764
H 1.860615 0.608385 -0.513221
H 0.435762 -1.332821 -1.167737
H 0.608420 -1.860593 0.513259
H -0.608407 1.860649 0.513068
H -0.435792 1.332692 -1.167874
H -1.860627 -0.608442 -0.513106
H -1.332741 -0.435712 1.167846

72

C 1.073897 -0.000335 0.140453
H 1.355400 0.001038 1.193598
H 1.982564 -0.001665 -0.464561
C 0.000861 1.074024 -0.139475
H 0.003475 1.356487 -1.192361
H -0.000115 1.982136 0.466372
C -1.074543 0.000732 0.135407
H -1.361009 0.002389 1.187214
H -1.980334 0.000308 -0.473907
C -0.000213 -1.074420 -0.136386
H 0.002116 -1.359927 -1.188451
H -0.002100 -1.980769 0.472095

73

C 0.039776 1.169286 -0.041207
C -1.188682 0.155133 -0.019172
C 1.291280 0.241377 0.031202
C -0.740475 -1.418863 0.081671
C 0.398097 -0.908553 0.078987
H 0.038558 1.761881 -0.951801
H 0.000334 1.843115 0.810069
H -1.774216 0.231095 -0.929780
H -1.824661 0.331590 0.842289
H 1.893881 0.382493 0.924964
H 1.923808 0.283629 -0.852050

TPSSh

1

C -0.000000 1.392012 -0.000000
C -1.205535 0.696007 0.000000
C -1.205535 -0.696007 -0.000000
C -0.000000 -1.392012 0.000000
C 1.205535 -0.696007 -0.000000
C 1.205535 0.696007 0.000000
H 0.000000 2.475648 0.000000
H -2.143990 1.237827 -0.000000
H -2.143990 -1.237827 0.000000
H 0.000000 -2.475648 -0.000000
H 2.143990 -1.237827 0.000000
H 2.143990 1.237827 -0.000000

2

C 0.695849 1.205826 -0.000002
C 1.391916 0.000168 0.000000
C 0.696140 -1.205659 0.000002
C -0.695851 -1.205826 0.000002
C -1.391917 -0.000168 0.000000
C -0.696141 1.205659 -0.000002
H 1.239001 2.145896 -0.000003
H 2.477604 0.000299 0.000000
H 1.239518 -2.145597 0.000004
H -1.239002 -2.145896 0.000004
H -1.239519 2.145597 -0.000003
H -2.477605 -0.000299 0.000000

3

C -0.538372 0.288075 0.000067
C 0.854643 0.288100 0.000504
C 1.551243 1.494433 -0.000031
C 0.855033 2.700532 -0.001051
C -0.537967 2.700507 -0.001489
C -1.234575 1.494161 -0.000935
H -1.069963 -0.658034 0.000489
H 1.396707 -0.650879 0.001259
H 2.636393 1.481627 0.000308
H 1.386645 3.646630 -0.001486
H -1.080050 3.639475 -0.002259
H -2.319725 1.506990 -0.001288

4

C -0.743561 0.695708 0.000187
C -1.913078 1.436632 0.000434
C -3.103189 0.699299 0.000276
C -3.103189 -0.699296 -0.000088
C -1.913077 -1.436629 -0.000322
C -0.743561 -0.695704 -0.000144
H -1.930646 2.520812 0.000682
H -4.053020 1.221792 0.000428
H -4.053019 -1.221789 -0.000178
H -1.930645 -2.520809 -0.000566
C 0.772351 -0.789058 0.000008
H 1.217773 -1.242612 0.888062
H 1.218101 -1.242772 -0.887787
C 0.772351 0.789062 -0.000029
H 1.217738 1.242619 -0.888099
H 1.218135 1.242774 0.887750

5

C 0.002177 1.856456 0.009497
H 0.015175 2.944197 0.016008
C 1.293304 1.302487 -0.067249
H 2.069220 2.060949 -0.143379
C -1.301886 1.332558 0.078376
H -2.059683 2.108453 0.161078
C -1.856468 0.041426 0.006309
H -2.944224 0.054399 0.009200
C 1.817170 -0.002129 -0.009516
H 2.904911 -0.015091 -0.016032
C 1.263173 -1.293856 0.056090
H 2.021614 -2.070411 0.125633
C -0.041446 -1.817186 -0.006286
H -0.054424 -2.904942 -0.009153
C -1.333158 -1.262621 -0.067228
H -2.109722 -2.020421 -0.143350

6

C 0.000000 1.380431 2.490417
C -0.000000 0.708109 1.252365
C -0.000000 1.438623 -0.012933
C -0.000000 2.846955 -0.049712
C -0.000000 -0.708109 1.252365
C -0.000000 0.730510 -1.239436
C -0.000000 -0.730510 -1.239436
C 0.000000 -1.438623 -0.012933
C -0.000000 -1.466529 -2.440705
C 0.000000 1.466529 -2.440705
C 0.000000 2.845761 -2.449821
C -0.000000 3.544463 -1.239619
H -0.000000 4.627828 -1.231829
H 0.000000 3.380701 -3.391933
C 0.000000 0.698703 3.689434
C -0.000000 -0.698703 3.689434
C 0.000000 -1.380431 2.490417
H 0.000000 1.247130 4.623759
H -0.000000 -1.247130 4.623759
C 0.000000 -2.846955 -0.049712
C -0.000000 -2.845761 -2.449821
H -0.000000 -3.380701 -3.391933
C 0.000000 -3.544463 -1.239619
H 0.000000 -4.627828 -1.231829
H 0.000000 0.947861 -3.389098
H -0.000000 -0.947861 -3.389098
H 0.000000 -3.408932 0.873678
H 0.000000 -2.461105 2.515433
H 0.000000 2.461105 2.515433
H -0.000000 3.408932 0.873678

7

C 0.000000 2.828864 0.876456
C -0.000000 3.549440 -0.296887
C 0.000000 1.418199 0.864177
C 0.000000 2.871063 -1.525849
C 0.000000 0.726274 -0.377569
C -0.000000 0.678141 2.089311
C -0.000000 1.492203 -1.561685
C 0.000000 -0.726274 -0.377569
C -0.000000 -0.678141 2.089311
C -0.000000 -1.492203 -1.561685
C 0.000000 -1.418199 0.864177
C 0.000000 -2.828864 0.876456
C -0.000000 -2.871063 -1.525849
C 0.000000 -3.549440 -0.296887
H 0.000000 3.339242 1.833551
H -0.000000 1.230677 3.022589
H 0.000000 0.997007 -2.523863
H -0.000000 -1.230677 3.022589
H 0.000000 -3.339242 1.833551
H 0.000000 -4.632664 -0.274439
H -0.000000 -0.997007 -2.523863
H -0.000000 -3.432713 -2.452550
H 0.000000 3.432713 -2.452550
H -0.000000 4.632664 -0.274439

8

C -0.887264 1.358462 0.006254
C 0.497805 1.548356 -0.000397
C 1.521172 0.557589 -0.010354
C -1.618287 0.148572 0.015807
C 1.398059 -0.826733 -0.013130
C -1.115167 -1.173987 0.013852
C 0.210881 -1.613470 -0.002012
H -1.487278 2.266437 0.006565
H 0.837008 2.581033 -0.002919
H 2.538821 0.942361 -0.014120
H -2.701052 0.247277 0.025976
H 2.328555 -1.389194 -0.025886
H -1.869981 -1.958511 0.021055
H 0.358507 -2.690297 -0.002445

9

C -0.000000 1.201677 0.000000
H -0.000000 2.287169 0.000000
C 1.142861 0.371337 0.000000
H 2.175226 0.706770 -0.000000

10

C 2.531271 -0.000000 -0.000000
C 1.927280 1.260029 -0.000035
C 0.545808 1.594685 -0.000036
C 1.927280 -1.260030 0.000043

11

C -0.011309 1.838239 0.002687
H -0.004298 2.929453 -0.001670
C -1.289976 1.307378 0.002384
H -2.067262 2.073499 -0.000553

12

C 3.111149 -0.693110 -0.000125
C 3.111149 0.693114 -0.000090
C 1.909681 1.443004 -0.000030
C 0.752375 0.709747 -0.000010

C 0.706327 -0.972173 -0.000000<br>H 1.344364 -1.850356 0.000000<br>C -1.142861 0.371337 -0.000000<br>H -2.175226 0.706770 -0.000000<br>C -0.706327 -0.972173 -0.000000<br>H -1.344364 -1.850356 0.000000	C -0.507227 0.709944 -0.000007<br>C 0.545807 -1.594685 0.000061<br>C -0.507227 -0.709944 0.000040<br>H 3.616753 -0.000001 -0.000012<br>H 2.606951 2.107781 -0.000067<br>H 0.315898 2.656140 -0.000037<br>H 2.606950 -2.107782 0.000056<br>H 0.315897 -2.656140 0.000068<br>C -1.940533 1.119417 0.000054<br>H -2.273390 2.153566 0.000022<br>C -2.764152 0.000000 -0.000018<br>H -3.845758 0.000000 -0.000060<br>C -1.940533 -1.119417 -0.000004<br>H -2.273389 -2.153566 -0.000002	C 1.318329 1.295584 0.001948<br>H 2.081551 2.074053 -0.002212<br>C 1.845833 0.017117 -0.002465<br>H 2.936237 0.005417 0.002916<br>C -1.845826 -0.017112 0.002463<br>H -2.936224 -0.005388 -0.002904<br>C -1.318335 -1.295596 -0.001944<br>H -2.081541 -2.074086 0.002203<br>C 0.011306 -1.838249 -0.002684<br>H 0.004266 -2.929469 0.001660<br>C 1.289973 -1.307370 -0.002384<br>H 2.067277 -2.073469 0.000552	C 0.752375 -0.709743 -0.000046<br>C 1.909681 -1.443000 -0.000104<br>H 4.058817 -1.218659 -0.000171<br>H 4.058817 1.218664 -0.000109<br>H 1.931948 2.526027 -0.000003<br>H 1.931948 -2.526022 -0.000131<br>C -0.752374 0.709747 0.000043<br>C -1.909681 1.443004 0.000104<br>C -3.111148 0.693114 0.000130<br>C -3.111148 -0.693110 0.000096<br>C -1.909680 -1.443000 0.000032<br>C -0.752374 -0.709743 0.000008<br>H -1.931947 2.526026 0.000133<br>H -4.058817 1.218663 0.000178<br>H -4.058816 -1.218660 0.000118<br>H -1.931947 -2.526023 0.000006
13	14	15	16
C 2.426101 -0.706480 -0.000001<br>C 1.240848 -1.398791 -0.000000<br>C 0.000004 -0.714237 0.000000<br>C -0.000003 0.714237 -0.000000<br>C 1.240833 1.398803 -0.000001<br>C 2.426093 0.706504 -0.000001<br>H -1.238140 -2.483476 0.000001<br>H 3.368523 -1.241356 -0.000001<br>H 1.238165 -2.483464 0.000000<br>C -1.240833 -1.398803 0.000001<br>C -1.240847 1.398790 -0.000000<br>H 1.238140 2.483476 -0.000001<br>H 3.368510 1.241389 -0.000001<br>C -2.426100 0.706480 0.000000<br>C -2.426093 -0.706505 0.000001<br>H -1.238164 2.483463 -0.000000<br>H -3.368522 1.241355 0.000001<br>H -3.368510 -1.241389 0.000001	C 0.735120 -1.244514 0.000000<br>C -0.735121 1.244514 -0.000000<br>C 1.434253 -0.000000 0.000000<br>C -0.735120 -1.244514 0.000000<br>C -1.434253 -0.000000 0.000000<br>C 0.735120 1.244514 0.000000<br>C -2.865202 -0.000000 -0.000000<br>C -3.563621 1.229318 -0.000000<br>C -2.874739 2.416403 -0.000000<br>C -1.472716 2.420396 -0.000000<br>C 1.472716 2.420396 0.000000<br>C 2.874739 2.416404 0.000000<br>C 3.563621 1.229318 0.000000<br>C 2.865202 -0.000000 0.000000<br>H -4.647636 1.215070 -0.000000<br>H -3.408348 3.359484 -0.000000<br>H -0.967792 3.376923 -0.000000<br>H 0.967791 3.376923 0.000001<br>H 3.408347 3.359484 0.000001<br>H 4.647635 1.215070 0.000000<br>C -3.563621 -1.229319 -0.000000<br>H -4.647636 -1.215071 -0.000000<br>C -1.472716 -2.420397 -0.000000<br>H -0.967791 -3.376923 -0.000000<br>C -2.874739 -2.416404 -0.000000<br>H -3.408347 -3.359485 -0.000000<br>C 3.563621 -1.229319 0.000000<br>H 4.647635 -1.215071 0.000000<br>C 1.472716 -2.420397 0.000000<br>H 0.967791 -3.376923 0.000000<br>C 2.874739 -2.416404 0.000000<br>H 3.408347 -3.359485 0.000000	C 2.436989 -0.704141 0.009477<br>C 1.244576 -1.396724 -0.005019<br>C 0.000000 -0.716378 -0.000345<br>C 0.000000 0.716378 -0.000345<br>C 1.244561 1.396734 -0.005111<br>C 2.436988 0.704170 0.009399<br>H -1.245432 -2.482653 -0.016780<br>H 3.380427 -1.238679 0.012573<br>H 1.245464 -2.482641 -0.016792<br>C -1.244560 -1.396734 -0.005109<br>C -1.244575 1.396724 -0.005019<br>H 1.245433 2.482653 -0.016782<br>H 3.380423 1.238710 0.012593<br>C -2.436988 0.704141 0.009479<br>C -2.436987 -0.704170 0.009401<br>H -1.245464 2.482640 -0.016792<br>H -3.380426 1.238679 0.012575<br>H -3.380422 -1.238711 0.012596	C 1.244179 3.523929 0.000000<br>C -0.000000 2.840072 -0.000000<br>C -0.000000 1.422545 -0.000000<br>C 1.231985 0.711270 0.000000<br>C 2.459622 1.420040 0.000000<br>C 2.429762 2.839444 0.000000<br>C -1.231985 0.711270 0.000000<br>C 1.231985 -0.711270 -0.000000<br>C -0.000000 -1.422545 -0.000000<br>C -1.231985 -0.711270 -0.000000<br>C -0.000000 -2.840072 -0.000000<br>C 1.244179 -3.523929 -0.000000<br>C 2.429762 -2.839444 -0.000000<br>C 2.459622 -1.420040 -0.000000<br>C 3.673948 -0.684490 0.000000<br>C 3.673948 0.684490 0.000000<br>H 4.611706 1.229290 0.000000<br>H 4.611706 -1.229290 0.000000<br>H 1.241249 4.608452 -0.000000<br>H 3.370445 3.379176 0.000000<br>H 1.241248 -4.608452 -0.000000<br>H 3.370445 -3.379176 -0.000000<br>C -1.244179 3.523929 -0.000000<br>C -2.429762 2.839444 -0.000000<br>C -2.459622 1.420040 -0.000000<br>H -1.241248 4.608452 -0.000000<br>H -3.370445 3.379176 -0.000000<br>C -3.673948 0.684490 0.000000<br>C -3.673948 -0.684490 0.000000<br>C -2.459622 -1.420040 0.000000<br>H -4.611706 1.229290 0.000000<br>H -4.611706 -1.229290 0.000000<br>C -1.244179 -3.523929 -0.000000<br>H -1.241249 -4.608452 -0.000000<br>C -2.429762 -2.839444 0.000000<br>H -3.370445 -3.379176 0.000000
17	18	19	20
C 2.471321 1.403047 -0.000203<br>C 1.220181 0.720357 -0.000095<br>C 3.649382 0.710790 -0.000294<br>C 0.000068 1.400249 0.000001<br>C 1.220182 -0.720359 -0.000084<br>C 3.649383 -0.710791 -0.000284<br>C 0.000068 -1.400250 0.000021<br>C -1.220036 0.720359 0.000106<br>C 2.471322 -1.403048 -0.000182<br>C -2.471183 1.403049 0.000204<br>C -1.220037 -0.720360 0.000116<br>C -2.471185 -1.403049 0.000224<br>C -3.649239 0.710794 0.000306<br>C -3.649240 -0.710794 0.000316<br>H 2.469726 2.487592 -0.000210<br>H 0.000073 2.485779 -0.000007<br>H 0.000073 -2.485779 0.000029<br>H 2.469727 -2.487593 -0.000174<br>H -2.469580 2.487594 0.000195<br>H -4.593292 1.242664 0.000379<br>H -2.469582 -2.487594 0.000232<br>H -4.593293 -1.242662 0.000398<br>H 4.593432 -1.242667 -0.000357<br>H 4.593431 1.242668 -0.000376	C 1.244179 3.523929 0.000000<br>C -0.000000 2.840072 -0.000000<br>C -0.000000 1.422545 -0.000000<br>C 1.231985 0.711270 0.000000<br>C 2.459622 1.420040 0.000000<br>C 2.429762 2.839444 0.000000<br>C -1.231985 0.711270 -0.000000<br>C 1.231985 -0.711270 -0.000000<br>C -0.000000 -1.422545 -0.000000<br>C -1.231985 -0.711270 -0.000000<br>C -0.000000 -2.840072 -0.000000<br>C 1.244179 -3.523929 -0.000000<br>C 2.429762 -2.839444 -0.000000<br>C 2.459622 -1.420040 -0.000000<br>C 3.673948 -0.684490 0.000000<br>C 3.673948 0.684490 0.000000<br>H 4.611706 1.229290 0.000000<br>H 4.611706 -1.229290 0.000000<br>H 1.241249 4.608452 -0.000000<br>H 3.370445 3.379176 0.000000<br>H 1.241248 -4.608452 -0.000000<br>H 3.370445 -3.379176 -0.000000<br>C -1.244179 3.523929 -0.000000<br>C -2.429762 2.839444 -0.000000<br>C -2.459622 1.420040 -0.000000<br>H -1.241248 4.608452 -0.000000<br>H -3.370445 3.379176 -0.000000<br>C -3.673948 0.684490 0.000000<br>C -3.673948 -0.684490 0.000000<br>C -2.459622 -1.420040 0.000000<br>H -4.611706 1.229290 0.000000<br>H -4.611706 -1.229290 0.000000<br>C -1.244179 -3.523929 -0.000000<br>H -1.241249 -4.608452 -0.000000<br>C -2.429762 -2.839444 0.000000<br>H -3.370445 -3.379176 0.000000	C -0.702005 0.658460 -0.205362<br>C 0.658522 0.701918 0.205497<br>H -1.417373 1.329688 -0.696938<br>H 1.329582 1.417494 0.697000<br>C -0.658573 -0.702041 0.205387<br>C 0.702018 -0.658591 -0.205220<br>H -1.329706 -1.417605 0.696761<br>H 1.417462 -1.329824 -0.696634	C 2.472300 -0.686089 -0.000001<br>C 1.227323 -1.399900 -0.000000<br>C 0.000004 -0.712577 0.000000<br>C -0.000003 0.712577 -0.000000<br>C 1.227309 1.399913 -0.000001<br>C 2.472293 0.686113 -0.000001<br>H -1.241593 -2.486477 0.000001<br>H 3.401619 -1.244426 -0.000001<br>H 1.241617 -2.486463 0.000000<br>C -1.227307 -1.399913 0.000001<br>C -1.227322 1.399901 0.000000<br>H 1.241593 2.486475 -0.000001<br>H 3.401607 1.244459 -0.000002<br>C -2.472298 0.686089 0.000001<br>C -2.472291 -0.686113 0.000001<br>H -1.241618 2.486464 -0.000000<br>H -3.401619 1.244425 0.000001<br>H -3.401606 -1.244460 0.000002
21	22	23	24
C -1.357998 -1.150839 0.001779<br>C -2.184210 0.000012 0.000101<br>C -1.357967 1.150858 -0.001670<br>C 0.000005 0.713477 0.000619	C 2.514890 -0.000000 0.044833<br>C 1.910323 1.269699 0.001009<br>C 0.564350 1.598168 -0.033578<br>C 1.910323 -1.269699 0.001003	C 0.758947 0.689290 -0.014296<br>C 0.758948 -0.689287 0.014240<br>C -0.739493 0.719052 -0.001306<br>C -1.900912 1.439855 0.005948	C 0.762540 0.674545 -0.000016<br>C 0.762540 -0.674541 -0.000041<br>C -0.756869 0.709425 0.000045<br>C -1.900660 1.442765 0.000108

C -0.000007 -0.713477 -0.000629<br>C 1.357996 1.150838 -0.001762<br>C 2.184208 -0.000014 -0.000089<br>C 1.357964 -1.150860 0.001622<br>H -1.716973 -2.175394 -0.000624<br>H -3.268397 0.000027 0.000150<br>H -1.716928 2.175419 0.000766<br>H 1.716974 2.175393 0.000650<br>H 3.268395 -0.000028 -0.000126<br>H 1.716924 -2.175421 -0.000827	C -0.559153 0.732416 0.002356<br>C 0.564350 -1.598168 -0.033586<br>C -0.559153 -0.732415 0.002352<br>H 3.601143 -0.000001 0.072567<br>H 2.603893 2.109507 -0.013350<br>H 0.332592 2.660885 -0.080868<br>H 2.603892 -2.109508 -0.013359<br>H 0.332591 -2.660884 -0.080878<br>C -1.905421 1.140002 0.008719<br>H -2.242375 2.169821 0.014094<br>C -2.730373 0.000000 0.011815<br>H -3.812927 0.000000 0.027552<br>C -1.905421 -1.140001 0.008724<br>H -2.242376 -2.169820 0.014103	C -3.119647 0.692281 0.004418<br>C -3.119647 -0.692278 -0.004191<br>C -1.900912 -1.439852 -0.005810<br>C -0.739493 -0.719048 0.001359<br>H -1.930509 2.525509 0.016217<br>H -4.065650 1.222530 0.006280<br>H -4.065650 -1.222527 -0.005984<br>H -1.930510 -2.525505 -0.016077<br>H 1.540232 1.437599 -0.034603<br>H 1.540234 -1.437595 0.034490	C -3.117179 0.687515 0.000132<br>C -3.117179 -0.687512 0.000094<br>C -1.900660 -1.442762 0.000029<br>C -0.756869 -0.709421 0.000008<br>H -1.922758 2.525867 0.000137<br>H -4.062459 1.217179 0.000181<br>H -4.062459 -1.217175 0.000114<br>H -1.922758 -2.525864 0.000003<br>H 1.540354 1.425508 -0.000029<br>H 1.540354 -1.425504 -0.000084
25	26	27	28
C 2.471321 1.403047 -0.000203<br>C 1.220181 0.720357 -0.000095<br>C 3.649382 0.710790 -0.000294<br>C 0.000068 1.400249 0.000001<br>C 1.220182 -0.720359 -0.000084<br>C 3.649383 -0.710791 -0.000284<br>C 0.000068 -1.400250 0.000021<br>C -1.220036 0.720359 0.000106<br>C 2.471322 -1.403048 -0.000182<br>C -2.471183 1.403049 0.000204<br>C -1.220037 -0.720360 0.000116<br>C -2.471185 -1.403049 0.000224<br>C -3.649239 0.710794 0.000306<br>C -3.649240 -0.710794 0.000316<br>H 2.469726 2.487592 -0.000210<br>H 0.000073 2.485779 -0.000007<br>H 0.000073 -2.485779 0.000029<br>H 2.469727 -2.487593 -0.000174<br>H -2.469580 2.487594 0.000195<br>H -4.593292 1.242664 0.000379<br>H -2.469582 -2.487594 0.000232<br>H -4.593293 -1.242662 0.000398<br>H 4.593432 -1.242667 -0.000357<br>H 4.593431 1.242668 -0.000376	C 2.514890 -0.000000 0.044833<br>C 1.910323 1.269699 0.001009<br>C 0.564350 1.598168 -0.033578<br>C 1.910323 -1.269699 0.001003<br>C -0.559153 0.732416 0.002356<br>C 0.564350 -1.598168 -0.033586<br>C -0.559153 -0.732415 0.002352<br>H 3.601143 -0.000001 0.072567<br>H 2.603893 2.109507 -0.013350<br>H 0.332592 2.660885 -0.080868<br>H 2.603892 -2.109508 -0.013359<br>H 0.332591 -2.660884 -0.080878<br>C -1.905421 1.140002 0.008719<br>H -2.242375 2.169821 0.014094<br>C -2.730373 0.000000 0.011815<br>H -3.812927 0.000000 0.027552<br>C -1.905421 -1.140001 0.008724<br>H -2.242376 -2.169820 0.014103	C -0.094948 1.288117 0.305145<br>C -1.321766 0.791734 -0.123068<br>C -1.201990 -0.593886 -0.098701<br>C 0.057414 -1.294532 -0.285952<br>C 1.284231 -0.798147 0.142268<br>C 1.164437 0.587471 0.117850<br>H -0.077870 2.192008 0.930199<br>H -2.262747 1.321155 -0.010893<br>H -2.070665 -1.195763 0.203198<br>H 0.040361 -2.198404 -0.911033<br>H 2.225215 -1.327563 0.030101<br>H 2.033115 1.189338 -0.184072	C 2.496610 0.000043 0.000011<br>C 1.905915 -1.263026 0.000143<br>C 0.550415 -1.591060 0.000191<br>C 1.905876 1.263082 -0.000116<br>C -0.552243 -0.747213 0.000111<br>C 0.550354 1.591073 -0.000153<br>C -0.552267 0.747194 -0.000075<br>H 3.582618 0.000059 0.000015<br>H 2.593259 -2.102308 0.000175<br>H 0.322393 -2.654075 0.000160<br>H 2.593184 2.102394 -0.000160<br>H 0.322306 2.654082 -0.000147<br>C -1.896462 -1.147013 -0.000266<br>H -2.241613 -2.171001 -0.000483<br>C -2.701582 -0.000042 0.000155<br>H -3.783581 -0.000065 0.000219<br>C -1.896510 1.146949 0.000102<br>H -2.241689 2.170927 0.000176
29	30	31	32
C 0.000000 2.828864 0.876456<br>C -0.000000 3.549440 -0.296887<br>C 0.000000 1.418199 0.864177<br>C 0.000000 2.871063 -1.525849<br>C 0.000000 0.726274 -0.377569<br>C -0.000000 0.678141 2.089311<br>C -0.000000 1.492203 -1.561685<br>C 0.000000 -0.726274 -0.377569<br>C -0.000000 -0.678141 2.089311<br>C -0.000000 -1.492203 -1.561685<br>C 0.000000 -1.418199 0.864177<br>C 0.000000 -2.828864 0.876456<br>C -0.000000 -2.871063 -1.525849<br>C 0.000000 -3.549440 -0.296887<br>H 0.000000 3.339242 1.833551<br>H -0.000000 1.230677 3.022589<br>H 0.000000 0.997007 -2.523863<br>H -0.000000 -1.230677 3.022589<br>H 0.000000 -3.339242 1.833551<br>H 0.000000 -4.632664 -0.274439<br>H -0.000000 -0.997007 -2.523863<br>H -0.000000 -3.432713 -2.452550<br>H 0.000000 3.432713 -2.452550<br>H -0.000000 4.632664 -0.274439	C 2.496610 0.000043 0.000011<br>C 1.905915 -1.263026 0.000143<br>C 0.550415 -1.591060 0.000191<br>C 1.905876 1.263082 -0.000116<br>C -0.552243 -0.747213 0.000111<br>C 0.550354 1.591073 -0.000153<br>C -0.552267 0.747194 -0.000075<br>H 3.582618 0.000059 0.000015<br>H 2.593259 -2.102308 0.000175<br>H 0.322393 -2.654075 0.000160<br>H 2.593184 2.102394 -0.000160<br>H 0.322306 2.654082 -0.000147<br>C -1.896462 -1.147013 -0.000266<br>H -2.241613 -2.171001 -0.000483<br>C -2.701582 -0.000042 0.000155<br>H -3.783581 -0.000065 0.000219<br>C -1.896510 1.146949 0.000102<br>H -2.241689 2.170927 0.000176	C 0.686949 0.721397 -0.000008<br>C 0.686949 -0.721393 -0.000043<br>C -0.735571 0.745057 0.000045<br>C -1.893122 1.510177 0.000107<br>C -3.049185 0.726985 0.000130<br>C -3.049185 -0.726982 0.000092<br>C -1.893122 -1.510174 0.000029<br>C -0.735571 -0.745054 0.000007<br>H -1.919848 2.594346 0.000137<br>H -4.020478 1.214830 0.000177<br>H -4.020478 -1.214827 0.000116<br>H -1.919848 -2.594343 0.000002<br>H 1.474224 1.472228 -0.000019<br>H 1.474225 -1.472224 -0.000088	C 0.045193 0.375585 0.502584<br>C 1.372660 0.220985 0.003325<br>C 1.372627 1.614784 -0.003340<br>C 0.045233 1.460116 -0.502769<br>H 1.954864 -0.572631 -0.441887<br>H 1.954730 2.408427 0.441954
33	34	35	36
C 0.000000 1.380431 2.490417<br>C -0.000000 0.708109 1.252365<br>C -0.000000 1.438623 -0.012933<br>C -0.000000 2.846955 -0.049712<br>C -0.000000 -0.708109 1.252365<br>C -0.000000 0.730510 -1.239436<br>C -0.000000 -0.730510 -1.239436<br>C 0.000000 -1.438623 -0.012933<br>C -0.000000 -1.466529 -2.440705<br>C 0.000000 1.466529 -2.440705<br>C 0.000000 2.845761 -2.449821<br>C -0.000000 3.544463 -1.239619<br>H -0.000000 4.627828 -1.231829<br>H 0.000000 3.380701 -3.391933<br>C 0.000000 0.698703 3.689434<br>C -0.000000 -0.698703 3.689434<br>C 0.000000 -1.380431 2.490417<br>H 0.000000 1.247130 4.623759<br>H -0.000000 -1.247130 4.623759<br>C 0.000000 -2.846955 -0.049712<br>C -0.000000 -2.845761 -2.449821<br>H -0.000000 -3.380701 -3.391933<br>C 0.000000 -3.544463 -1.239619<br>H 0.000000 -4.627828 -1.231829<br>H 0.000000 0.947861 -3.389098<br>H -0.000000 -0.947861 -3.389098<br>H 0.000000 -3.408932 0.873678<br>H 0.000000 -2.461105 2.515433<br>H 0.000000 2.461105 2.515433<br>H -0.000000 3.408932 0.873678	C -0.001344 1.586622 -0.000080<br>C 1.241239 1.003073 -0.254602<br>C 1.561569 -0.377605 0.060621<br>C -1.243018 1.000855 0.254661<br>C 0.724197 -1.432149 0.172564<br>C -1.560875 -0.380266 -0.060360<br>C -0.721664 -1.433395 -0.172904<br>H -0.002368 2.681888 -0.000380<br>H 2.081618 1.673420 -0.408512<br>H 2.612967 -0.572153 0.299069<br>H -2.084583 1.669808 0.408119<br>H 1.140373 -2.376759 0.524018<br>H -2.612091 -0.576771 -0.298022<br>H -1.136333 -2.378764 -0.524029	C 0.735120 -1.244514 0.000000<br>C -0.735121 1.244514 -0.000000<br>C 1.434253 -0.000000 0.000000<br>C -0.735120 -1.244514 0.000000<br>C -1.434253 -0.000000 0.000000<br>C 0.735120 1.244514 0.000000<br>C -2.865202 -0.000000 -0.000000<br>C -3.563621 1.229318 -0.000000<br>C -2.874739 2.416403 -0.000000<br>C -1.472716 2.420396 -0.000000<br>C 1.472716 2.420396 0.000000<br>C 2.874739 2.416404 0.000000<br>C 3.563621 1.229318 0.000000<br>C 2.865202 -0.000000 0.000000<br>H -4.647636 1.215070 -0.000000<br>H -3.408348 3.359484 -0.000000<br>H -0.967792 3.376923 -0.000000<br>H 0.967791 3.376923 0.000001<br>H 3.408347 3.359484 0.000001<br>H 4.647635 1.215070 0.000000<br>C -3.563621 -1.229319 -0.000000<br>H -4.647636 -1.215071 -0.000000<br>C -1.472716 -2.420397 -0.000000<br>H -0.967791 -3.376923 -0.000000<br>C -2.874739 -2.416404 -0.000000<br>H -3.408347 -3.359485 -0.000000<br>C 3.563621 -1.229319 0.000000<br>H 4.647635 -1.215071 0.000000<br>C 1.472716 -2.420397 0.000000<br>H 0.967791 -3.376923 0.000000	C 0.686949 0.721397 -0.000008<br>C 0.686949 -0.721393 -0.000043<br>C -0.735571 0.745057 0.000045<br>C -1.893122 1.510177 0.000107<br>C -3.049185 0.726985 0.000130<br>C -3.049185 -0.726982 0.000092<br>C -1.893122 -1.510174 0.000029<br>C -0.735571 -0.745054 0.000007<br>H -1.919848 2.594346 0.000137<br>H -4.020478 1.214830 0.000177<br>H -4.020478 -1.214827 0.000116<br>H -1.919848 -2.594343 0.000002<br>H 1.474224 1.472228 -0.000019<br>H 1.474225 -1.472224 -0.000088

		C 2.874739 -2.416404 0.000000<br>H 3.408347 -3.359485 0.000000	
37<br>C 0.623398 -1.227459 0.000137<br>C -0.621444 -1.227657 -0.000022<br>C -1.461663 -0.133516 -0.000003<br>C -0.701467 1.052922 0.000006<br>H -2.543097 -0.135686 0.000025<br>H -1.224748 2.003342 0.000006<br>C 1.461664 -0.131566 -0.000050<br>H 2.543120 -0.132116 -0.000018<br>C 0.699940 1.053819 -0.000004<br>H 1.221939 2.004942 0.000050	38<br>C 2.531271 -0.000000 -0.000000<br>C 1.927280 1.260029 -0.000035<br>C 0.545808 1.594685 -0.000036<br>C 1.927280 -1.260030 0.000043<br>C -0.507227 0.709944 -0.000007<br>C 0.545807 -1.594685 0.000061<br>C -0.507227 -0.709944 0.000040<br>H 3.616753 -0.000001 -0.000012<br>H 2.606951 2.107781 -0.000067<br>H 0.315898 2.656140 -0.000037<br>H 2.606950 -2.107782 0.000056<br>H 0.315897 -2.656140 0.000068<br>C -1.940533 1.119417 0.000054<br>H -2.273390 2.153566 0.000022<br>C -2.764152 0.000000 -0.000018<br>H -3.845758 0.000000 -0.000060<br>C -1.940533 -1.119417 -0.000004<br>H -2.273389 -2.153566 -0.000002	39<br>C -1.555391 -0.677118 0.378695<br>C -0.661260 -1.562207 -0.378653<br>C 0.661245 1.562214 -0.378672<br>C 0.677110 -1.555406 -0.378672<br>C 1.555421 0.677117 0.378608<br>C 1.562226 -0.661254 0.378616<br>H -2.342355 -1.182750 0.934769<br>H -1.158863 -2.354291 -0.934699<br>H 1.158817 2.354307 -0.934733<br>H 1.182722 -2.342391 -0.934733<br>H 2.342431 1.182739 0.934627<br>H 2.354337 -1.158841 0.934638<br>C -1.562196 0.661251 0.378712<br>H -2.354263 1.158839 0.934796<br>C -0.677125 1.555406 -0.378628<br>H -1.182767 2.342393 -0.934659	40<br>C -1.297338 1.102350 -0.000104<br>C -2.120008 -0.140888 -0.000107<br>C -1.301749 -1.218794 -0.000083<br>C 0.076524 -0.708532 0.000058<br>C 0.008127 0.740993 0.000003<br>C 1.381989 -1.069890 0.000260<br>C 2.204660 0.173346 -0.000033<br>C 1.386401 1.251253 0.000128<br>H -1.711025 2.102865 -0.000155<br>H -3.200656 -0.152928 -0.000149<br>H -1.601542 -2.256447 -0.000080<br>H 1.795670 -2.070408 0.000322<br>H 3.285308 0.185391 -0.000121<br>H 1.686198 2.288904 0.000122
41<br>C 0.364321 1.436477 0.000057<br>C -0.768430 0.748090 -0.000065<br>C -1.426165 -0.402719 0.000067<br>C -0.263655 -1.039541 -0.000067<br>C 1.061856 -1.033739 0.000059<br>C 1.032074 0.291433 -0.000051	42<br>C 0.758947 0.689290 -0.014296<br>C 0.758948 -0.689287 0.014240<br>C -0.739493 0.719052 -0.001306<br>C -1.900912 1.439855 0.005948<br>C -3.119647 0.692281 0.004418<br>C -3.119647 -0.692278 -0.004191<br>C -1.900912 -1.439852 -0.005810<br>C -0.739493 -0.719048 0.001359<br>H -1.930509 2.525509 0.016217<br>H -4.065650 1.222530 0.006280<br>H -4.065650 -1.222527 -0.005984<br>H -1.930510 -2.525505 -0.016077<br>H 1.540232 1.437599 -0.034603<br>H 1.540234 -1.437595 0.034490	43<br>C -1.430358 1.156430 -0.000115<br>C -2.200453 -0.145552 -0.000074<br>C -1.345469 -1.194228 -0.000059<br>C 0.011836 -0.679647 0.000006<br>C -0.011836 0.679646 -0.000023<br>C 1.430358 -1.156430 0.000136<br>C 2.200453 0.145553 0.000058<br>C 1.345469 1.194228 0.000075<br>H -1.672519 1.767873 -0.877569<br>H -1.672587 1.767948 0.877266<br>H -3.280380 -0.198335 -0.000092<br>H -1.628685 -2.238750 -0.000049<br>H 1.672501 -1.767847 0.877613<br>H 1.672606 -1.767974 -0.877222<br>H 3.280380 0.198335 0.000062<br>H 1.628684 2.238750 0.000073	44<br>C -0.399171 -0.037446 0.217424<br>C 0.869794 -0.114727 0.230222<br>C 1.405625 1.083962 0.137251<br>C 0.975538 2.284160 0.039165<br>C -0.425915 2.304999 0.030184<br>C -1.186798 1.083290 0.123777<br>H -1.008312 3.217880 -0.045789<br>H -2.265280 1.120960 0.115110
45<br>C -0.345282 -0.506452 -1.176513<br>H -0.154565 -0.240306 -2.206661<br>C -1.085192 -1.539893 -0.732165<br>H -1.603901 -2.264963 -1.345568<br>C -0.345294 -0.506430 1.176549<br>H -0.154597 -0.240259 2.206694<br>C -1.085292 -1.539812 0.732213<br>H -1.604068 -2.264828 1.345625<br>C 0.195465 0.251419 0.000014<br>H 1.292715 0.280526 0.000025<br>H -0.133260 1.298703 -0.000004	46<br>C 3.111149 -0.693110 -0.000125<br>C 3.111149 0.693114 -0.000090<br>C 1.909681 1.443004 -0.000030<br>C 0.752375 0.709747 -0.000010<br>C 0.752375 -0.709743 -0.000046<br>C 1.909681 -1.443000 -0.000104<br>H 4.058817 -1.218659 -0.000171<br>H 4.058817 1.218664 -0.000109<br>H 1.931948 2.526027 -0.000003<br>H 1.931948 -2.526022 -0.000131<br>C -0.752374 0.709747 0.000043<br>C -1.909681 1.443004 0.000104<br>C -3.111148 0.693114 0.000130<br>C -3.111148 -0.693110 0.000096<br>C -1.909680 -1.443000 0.000032<br>C -0.752374 -0.709743 0.000008<br>H -1.931947 2.526026 0.000133<br>H -4.058817 1.218663 0.000178<br>H -4.058816 -1.218660 0.000118<br>H -1.931947 -2.526023 0.000006	47<br>C -0.000000 2.835304 -0.000000<br>C -0.000000 1.482660 -0.000000<br>C -1.284019 0.741321 -0.000496<br>C -2.455482 1.417633 -0.001683<br>C 1.284019 0.741321 0.000496<br>C -1.284019 -0.741321 0.000496<br>C -0.000000 -1.482660 -0.000000<br>C 1.284019 -0.741321 -0.000496<br>C 0.000000 -2.835304 -0.000000<br>C -2.455482 -1.417633 0.001683<br>C 2.455482 1.417633 0.001683<br>C 2.455482 -1.417633 -0.001683<br>C -3.779602 -0.735416 0.196514<br>H -4.561833 -1.273949 -0.348655<br>H -4.041482 -0.840885 1.260644<br>C -3.779602 0.735416 -0.196514<br>H -4.561833 1.273949 0.348655<br>H -4.041482 0.840885 -1.260644<br>C -2.527643 -2.905494 -0.192089<br>H -3.382949 -3.313258 0.356828<br>H -2.754701 -3.079774 -1.255167<br>C -1.252352 -3.641492 0.195571<br>H -1.178234 -4.586689 -0.352376<br>H -1.289032 -3.924134 1.258977<br>C 1.252352 -3.641492 -0.195571<br>H 1.178234 -4.586689 0.352376<br>H 1.289032 -3.924134 -1.258977<br>C 2.527643 -2.905494 0.192089<br>H 3.382949 -3.313258 -0.356828<br>H 2.754701 -3.079774 1.255167<br>C 3.779602 -0.735416 -0.196514<br>H 4.561833 -1.273949 0.348655<br>H 4.041482 -0.840885 -1.260644<br>C 3.779602 0.735416 0.196514<br>H 4.561833 1.273949 -0.348655<br>H 4.041482 0.840885 1.260644<br>C 2.527643 2.905494 -0.192089<br>H 3.382949 3.313258 0.356828<br>H 2.754701 3.079774 -1.255167<br>C 1.252352 3.641492 0.195571<br>H 1.178234 4.586689 -0.352376<br>H 1.289032 3.924134 1.258977<br>C -1.252352 3.641492 -0.195571<br>H -1.178234 4.586689 0.352376<br>H -1.289032 3.924134 -1.258977<br>C -2.527643 2.905494 0.192089<br>H -3.382949 3.313258 -0.356828<br>H -2.754701 3.079774 1.255167	48<br>C -0.103088 -0.038269 1.156645<br>H -0.154581 0.031694 2.235257<br>C 0.238216 1.177210 0.219366<br>H 0.447170 2.174869 0.582638<br>C 0.215800 0.738243 -1.050970<br>H 0.401361 1.292663 -1.957017<br>C -0.304573 -1.115853 0.378745<br>H -0.556844 -2.121342 0.675545<br>C -0.115125 -0.667976 -0.983301<br>H -0.210579 -1.312501 -1.853913
49<br>C -0.000000 2.835304 -0.000000<br>C -0.000000 1.482660 -0.000000<br>C -1.284019 0.741321 -0.000496<br>C -2.455482 1.417633 -0.001683<br>C 1.284019 0.741321 0.000496<br>C -1.284019 -0.741321 0.000496<br>C -0.000000 -1.482660 -0.000000	50<br>C 0.762540 0.674545 -0.000016<br>C 0.762540 -0.674541 -0.000041<br>C -0.756869 0.709425 0.000045<br>C -1.900660 1.442765 0.000108<br>C -3.117179 0.687515 0.000132<br>C -3.117179 -0.687512 0.000094<br>C -1.900660 -1.442762 0.000029	51<br>C -0.829928 -0.139430 0.022149<br>C 1.335435 1.110615 0.022043<br>C 0.670082 2.263155 0.021252<br>C -1.495281 1.013111 0.021357<br>H -1.380100 -1.075783 0.022653<br>H 2.421428 1.118831 0.022468<br>H 1.220255 3.199508 0.021039	52<br>C 0.109796 -1.419226 -0.064661<br>C 0.110807 1.419151 0.064641<br>C 1.254336 0.721924 0.110398<br>C 1.253821 -0.722814 -0.110418<br>H 0.111356 -2.496605 -0.190244<br>H 0.113134 2.496528 0.190227<br>H 2.199873 1.222175 0.288502

C 1.284019 -0.741321 -0.000496<br>C 0.000000 -2.835304 -0.000000<br>C -2.455482 -1.417633 0.001683<br>C 2.455482 1.417633 0.001683<br>C 2.455482 -1.417633 -0.001683<br>C -3.779602 -0.735416 0.196514<br>H -4.561833 -1.273949 -0.348655<br>H -4.041482 -0.840885 1.260644<br>C -3.779602 0.735416 -0.196514<br>H -4.561833 1.273949 0.348655<br>H -4.041482 0.840885 -1.260644<br>C -2.527643 -2.905494 -0.192089<br>H -3.382949 -3.313258 0.356828<br>H -2.754701 -3.079774 -1.255167<br>C -1.252352 -3.641492 0.195571<br>H -1.178234 -4.586689 -0.352376<br>H -1.289032 -3.924134 1.258977<br>C 1.252352 -3.641492 -0.195571<br>H 1.178234 -4.586689 0.352376<br>H 1.289032 -3.924134 -1.258977<br>C 2.527643 -2.905494 0.192089<br>H 3.382949 -3.313258 -0.356828<br>H 2.754701 -3.079774 1.255167<br>C 3.779602 -0.735416 -0.196514<br>H 4.561833 -1.273949 0.348655<br>H 4.041482 -0.840885 -1.260644<br>C 3.779602 0.735416 0.196514<br>H 4.561833 1.273949 -0.348655<br>H 4.041482 0.840885 1.260644<br>C 2.527643 2.905494 -0.192089<br>H 3.382949 3.313258 0.356828<br>H 2.754701 3.079774 -1.255167<br>C 1.252352 3.641492 0.195571<br>H 1.178234 4.586689 -0.352376<br>H 1.289032 3.924134 1.258977<br>C -1.252352 3.641492 -0.195571<br>H -1.178234 4.586689 0.352376<br>H -1.289032 3.924134 -1.258977<br>C -2.527643 2.905494 0.192089<br>H -3.382949 3.313258 -0.356828<br>H -2.754701 3.079774 1.255167	C -0.756869 -0.709421 0.000008<br>H -1.922758 2.525867 0.000137<br>H -4.062459 1.217179 0.000181<br>H -4.062459 -1.217175 0.000114<br>H -1.922758 -2.525864 0.000003<br>H 1.540354 1.425508 -0.000029<br>H 1.540354 -1.425504 -0.000084	H -2.581273 1.004896 0.021224<br>C 0.668211 -0.234100 0.022404<br>H 1.004594 -0.816673 0.891745<br>H 1.004840 -0.817248 -0.846464<br>C -0.828056 2.357826 0.020627<br>H -1.164235 2.940045 -0.849038<br>H -1.164890 2.941326 0.889171	H 2.199001 -1.223739 -0.288521<br>C -1.189737 0.725579 -0.249065<br>H -2.033684 1.270294 0.180510<br>H -1.336878 0.741061 -1.339457<br>C -1.190254 -0.724729 0.249047<br>H -2.034588 -1.268842 -0.180528<br>H -1.337407 -0.740108 1.339440
53	54	55	56
C 0.500403 0.014755 -0.000000<br>C 1.999992 -0.118935 -0.000000<br>C 1.155134 1.127204 0.000000<br>H -0.437695 -0.512577 0.000000<br>H 2.512698 -0.420460 -0.912917<br>H 2.512698 -0.420460 0.912917<br>H 1.161316 2.203331 -0.000000	C 0.363922 -0.080871 0.000000<br>C 1.868492 -0.080773 0.000000<br>C 1.116125 1.222182 -0.000000<br>H -0.144108 -0.374230 -0.909499<br>H -0.144108 -0.374230 0.909499<br>H 2.376563 -0.374060 -0.909499<br>H 2.376563 -0.374060 0.909499<br>H 1.116095 1.808827 0.909497<br>H 1.116095 1.808827 -0.909497	H -1.234925 2.041968 -0.055515<br>C -0.709492 1.139369 0.267706<br>C -0.654161 -1.358507 -0.027821<br>C 1.487845 -0.095485 0.247608<br>C 0.662593 -1.351921 0.181490<br>C 0.719549 1.120749 -0.279905<br>C -1.478480 -0.108752 -0.178109<br>H -1.178359 -2.308217 -0.089210<br>H 1.804454 0.079238 1.284588<br>H 1.186134 -2.295691 0.306904<br>H 0.682817 1.075007 -1.373864<br>H -1.794268 -0.003007 -1.224616<br>H -0.672931 1.166299 1.362299<br>H 2.412548 -0.235163 -0.322413<br>H 1.245521 2.042517 -0.017365<br>H -2.403652 -0.209515 0.399307	C 1.768620 0.408156 -0.000048<br>C 2.375349 1.459055 0.000009<br>C 0.375376 2.683556 -0.001313<br>C -0.291825 1.527918 -0.001146<br>H -0.193295 3.609953 -0.001859<br>H -1.378445 1.557171 -0.001589<br>C 0.327286 0.116959 -0.000368<br>H -0.005079 -0.446564 -0.878021<br>H -0.005578 -0.445793 0.877593<br>C 1.906871 2.852879 -0.000808<br>H 2.227910 3.422940 0.876840<br>H 2.228564 3.422083 -0.878774
57	58	59	60
C 0.569013 -1.255151 -0.128030<br>C -0.650481 -1.286439 -0.094261<br>C -1.541722 -0.158797 0.049238<br>C -0.855480 1.004974 0.074670<br>H -2.612179 -0.220021 0.190662<br>H -1.392556 1.937416 0.222887<br>C 1.506394 -0.165745 0.195861<br>H 1.763643 -0.194827 1.260072<br>H 2.437368 -0.143395 -0.370993<br>C 0.637243 1.106430 -0.168376<br>H 0.774134 1.285863 -1.243072<br>H 1.047974 1.980085 0.345598	C -0.000026 -1.222572 -0.137004<br>C 1.232475 -0.318296 0.104531<br>C -1.232488 -0.318240 0.104520<br>C 0.665939 1.071133 -0.045061<br>C -0.665884 1.071166 -0.045037<br>H -0.000031 -1.557091 -1.176840<br>H -0.000049 -2.113213 0.492836<br>H 2.044963 -0.526086 -0.597227<br>H 1.648673 -0.455057 1.110710<br>H -1.648700 -0.454993 1.110694<br>H -2.044980 -0.525988 -0.597245<br>H 1.287000 1.957580 -0.101707<br>H -1.286901 1.957644 -0.101670	C 0.004021 0.488508 -0.000036<br>C 1.513986 0.373108 -0.000040<br>C 1.513952 1.941392 -0.000075<br>C 0.003992 1.825927 -0.000252<br>H -0.781572 -0.258630 -0.000003<br>H 1.957181 -0.083765 -0.888645<br>H 1.957155 -0.083695 0.888611<br>H 1.957202 2.398213 -0.888677<br>H 1.957027 2.398286 0.888579<br>H -0.781633 2.573031 -0.000376	C -0.743561 0.695708 0.000187<br>C -1.913078 1.436632 0.000434<br>C -3.103189 0.699299 0.000276<br>C -3.103189 -0.699296 -0.000088<br>C -1.913077 -1.436629 -0.000322<br>C -0.743561 -0.695704 -0.000144<br>H -1.930646 2.520812 0.000682<br>H -4.053020 1.221792 0.000428<br>H -4.053019 -1.221789 -0.000178<br>H -1.930645 -2.520809 -0.000566<br>C 0.772351 -0.789058 0.000008<br>H 1.217773 -1.242612 0.888062<br>H 1.218101 -1.242772 -0.887787<br>C 0.772351 0.789062 -0.000029<br>H 1.217738 1.242619 -0.888099<br>H 1.218135 1.242774 0.887750
61	62	63	64
H -0.028401 2.251818 0.171613<br>C 0.093692 1.273875 -0.303107<br>C 0.051207 -1.408932 -0.024638<br>C -1.284144 0.533811 -0.286247<br>H -1.971022 1.036135 0.402341<br>H 0.390572 1.460269 -1.337488<br>H -1.744403 0.591202 -1.275840<br>C 1.272247 0.516246 0.390146<br>H 1.119321 0.480885 1.469626<br>H 2.212581 1.037815 0.197286<br>C -1.138742 -0.902107 0.219883<br>C 1.240711 -0.877375 -0.220559<br>H 1.977239 -1.203532 -0.945394<br>H -1.885024 -1.322414 0.883453	C -0.035153 0.491394 0.000059<br>C 1.538464 0.491420 -0.000079<br>C 1.538442 1.823089 0.000081<br>C -0.035175 1.823063 -0.000333<br>H -0.797228 -0.275203 0.000193<br>H 2.300565 -0.275152 -0.000076<br>H 2.300518 2.589686 0.000214<br>H -0.797275 2.589635 -0.000599	H -2.476659 -0.261435 -0.140348<br>C -1.453258 -0.153382 0.230892<br>C 0.859551 -1.181838 0.230935<br>C 0.593672 1.335161 0.230902<br>C 1.453258 0.153382 -0.230892<br>C -0.859551 1.181838 -0.230935<br>C -0.593672 -1.335161 -0.230902<br>H 0.894978 -1.230559 1.325998<br>H 0.618130 1.390160 1.325961<br>H 1.513245 0.159662 -1.325958<br>H -0.894978 1.230559 -1.325998<br>H -0.618130 -1.390160 -1.325961<br>H -1.513245 -0.159662 1.325958<br>H 1.464699 -2.014206 -0.140282<br>H 1.011907 2.275461 -0.140267<br>H 2.476659 0.261435 0.140348<br>H -1.464699 2.014206 0.140282<br>H -1.011907 -2.275461 0.140267	C 0.308614 -1.516372 -0.407712<br>C 1.539435 -0.758175 0.109100<br>C 1.539409 0.758222 -0.109106<br>C -0.962842 -1.240678 0.410361<br>C 0.308566 1.516380 0.407714<br>C -1.774461 -0.000030 -0.000005<br>C -0.962885 1.240653 -0.410353<br>H 0.139493 -1.290498 -1.467139<br>H 1.635829 -0.964790 1.182102<br>H 1.635789 0.964841 -1.182109<br>H 0.139458 1.290498 1.467141<br>H -0.672265 -1.153988 1.463183<br>H -2.434374 0.262931 0.833232<br>H 0.529626 -2.586966 -0.357422<br>H 2.438046 -1.170303 -0.362089<br>H 2.438009 1.170380 0.362077<br>H -1.627221 -2.108463 0.357287<br>H 0.529546 2.586980 0.357426<br>H -2.434344 -0.263020 -0.833257

			H -1.627293 2.108416 -0.357261<br>H -0.672310 1.153992 -1.463179
65<br>C -0.657348 -1.214532 -0.171273<br>C 0.548634 -0.970244 -0.140546<br>C 0.690946 1.239261 0.149069<br>C -0.515061 0.995230 0.118259<br>C -1.607353 -0.038089 -0.016078<br>H -2.244916 0.115166 -0.887433<br>H -2.243129 -0.114796 0.866614<br>C 1.641018 0.062864 -0.006032<br>H 2.278405 -0.090432 0.865449<br>H 2.276964 0.139619 -0.888596	66<br>C 0.000000 -0.000000 1.773747<br>H 0.623565 0.623565 2.423985<br>H -0.623565 -0.623565 2.423985<br>C 0.888612 -0.888612 0.888612<br>H 1.520547 -1.520547 1.520547<br>C -0.888612 0.888612 0.888612<br>H -1.520547 1.520547 1.520547<br>C -0.000000 1.773747 -0.000000<br>H 0.623565 2.423985 0.623565<br>H -0.623565 2.423985 -0.623565<br>C 1.773747 -0.000000 -0.000000<br>H 2.423985 -0.623565 -0.623565<br>H 2.423985 0.623565 0.623565<br>C 0.888612 0.888612 -0.888612<br>H 1.520547 1.520547 -1.520547<br>C -0.000000 0.000000 -1.773747<br>H -0.623565 0.623565 -2.423985<br>H 0.623565 -0.623565 -2.423985<br>C 0.000000 -1.773747 -0.000000<br>H -0.623565 -2.423985 0.623565<br>H 0.623565 -2.423985 -0.623565<br>C -1.773747 -0.000000 0.000000<br>H -2.423985 -0.623565 0.623565<br>H -2.423985 0.623565 -0.623565<br>C -0.888612 -0.888612 -0.888612<br>H -1.520547 -1.520547 -1.520547	67<br>C -0.632560 -0.237551 1.728773<br>C -0.254248 1.203464 1.339546<br>C -0.132953 -1.266784 -1.093252<br>C -0.643090 1.682570 -0.075542<br>C 0.016598 -0.014500 -1.970109<br>C 0.297967 1.305234 -1.234522<br>H -1.590350 -0.483971 1.257894<br>H 0.819797 1.365311 1.492352<br>H -1.665737 1.354653 -0.298578<br>H -1.122949 -1.259889 -0.629179<br>H -0.893565 0.101622 -2.568809<br>H -0.825247 -0.264738 2.805955<br>H -0.757959 1.870978 2.045441<br>H -0.121854 -2.144596 -1.744941<br>H -0.684222 2.775390 -0.035671<br>H 0.831246 -0.174405 -2.685533<br>H 0.257351 2.100559 -1.985489<br>H 1.327855 1.312364 -0.861575<br>C 0.395024 -1.341829 1.434242<br>H -0.074279 -2.302400 1.675326<br>H 1.237429 -1.234508 2.126467<br>C 0.946148 -1.399373 0.001539<br>H 1.691208 -0.609152 -0.124770<br>H 1.493341 -2.338460 -0.117239	68<br>C -1.522752 -0.000013 -0.000020<br>C 0.658592 1.218055 0.389584<br>C 0.658610 -1.218055 -0.389569<br>C 1.522752 0.000012 -0.000007<br>C -0.658595 -1.218069 0.389533<br>C -0.658608 1.218071 -0.389525<br>H -2.177601 0.263226 0.835703<br>H -2.177537 -0.263277 -0.835786<br>H -1.213515 2.143803 -0.217191<br>H 0.434900 -1.188118 -1.461299<br>H 1.213486 2.143807 0.217314<br>H -0.434893 1.188182 -1.461255<br>H 0.434879 1.188088 1.461313<br>H 2.177589 -0.263261 0.835715<br>H 2.177549 0.263306 -0.835754<br>H -1.213488 -2.143815 0.217225<br>H -0.434889 -1.188142 1.461264<br>H 1.213516 -2.143794 -0.217273
69<br>C 0.607092 -1.286927 -0.022402<br>C -0.607126 -1.286941 0.022475<br>C -1.592392 -0.194737 0.117610<br>H -1.980906 -0.103331 1.135619<br>H -2.448781 -0.286203 -0.553377<br>C 1.592405 -0.194784 -0.117561<br>H 1.980941 -0.103416 -1.135565<br>H 2.448775 -0.286257 0.553449<br>C 0.717208 1.049336 0.282785<br>H 1.227771 1.958309 -0.051669<br>H 0.672804 1.087409 1.374964<br>C -0.717170 1.049341 -0.282783<br>H -0.672763 1.087373 -1.374964<br>H -1.227708 1.958342 0.051634	70<br>C 0.158793 1.277942 0.129377<br>C -1.136861 0.539084 -0.245228<br>C -0.887611 -0.886933 0.257131<br>C 0.568770 -1.149040 -0.154143<br>C 1.285600 0.215569 0.013054<br>H 0.340969 2.150962 -0.500042<br>H 0.088503 1.636470 1.159828<br>H -1.263135 0.526028 -1.333002<br>H -2.028498 1.001894 0.183062<br>H -0.982738 -0.914633 1.347960<br>H -1.585183 -1.620428 -0.152902<br>H 1.038458 -1.943585 0.428789<br>H 0.597429 -1.459970 -1.202327<br>H 1.915503 0.222521 0.904784<br>H 1.941189 0.418879 -0.835791	71<br>C 1.024698 0.335058 0.137774<br>C 0.335049 -1.024704 -0.137753<br>C -0.335052 1.024689 -0.137858<br>C -1.024695 -0.335043 0.137837<br>H 1.309131 0.428117 1.188080<br>H 1.878101 0.614065 -0.482286<br>H 0.428036 -1.309181 -1.188053<br>H 0.614098 -1.878081 0.482325<br>H -0.614085 1.878134 0.482132<br>H -0.428066 1.309049 -1.188187<br>H -1.878113 -0.614118 -0.482170<br>H -1.309100 -0.427985 1.188161	72<br>C 1.075070 -0.000322 0.149439<br>H 1.332508 0.001083 1.211083<br>H 2.000532 -0.001629 -0.434543<br>C 0.000884 1.075206 -0.148456<br>H 0.003516 1.333612 -1.209862<br>H -0.000037 2.000135 0.436371<br>C -1.075762 0.000747 0.144365<br>H -1.338202 0.002409 1.204783<br>H -1.998461 0.000358 -0.443975<br>C -0.000192 -1.075630 -0.145349<br>H 0.002180 -1.337103 -1.206005<br>H -0.002037 -1.998865 0.442147
73<br>C -0.038779 1.174506 -0.042313<br>C -1.290416 0.240146 0.049195<br>C 1.198881 0.156849 0.005659<br>C -0.387213 -0.905202 0.110963<br>C 0.758030 -1.423974 0.123713<br>H -0.037341 1.742932 -0.970629<br>H -0.002269 1.869744 0.794474<br>H -1.929734 0.267283 -0.833076<br>H -1.892818 0.392043 0.944717<br>H 1.824000 0.344492 0.875102<br>H 1.790753 0.220741 -0.904020			
